# The Expression of *IbMYB1* Is Essential to Maintain the Purple Color of Leaf and Storage Root in Sweet Potato [*Ipomoea batatas* (L.) Lam]

**DOI:** 10.3389/fpls.2021.688707

**Published:** 2021-09-23

**Authors:** Daowei Zhang, Yongjun Tan, Fang Dong, Ya Zhang, Yanlan Huang, Yizhou Zhou, ZhiJian Zhao, Qin Yin, Xuehua Xie, Xiewang Gao, Chaofan Zhang, Naimei Tu

**Affiliations:** ^1^College of Agronomy, Hunan Agricultural University, Changsha, China; ^2^Crop Research Institute, Hunan Academy of Agricultural Sciences, Changsha, China; ^3^Department of Biology, School of Life Sciences, Southern University of Science and Technology, Shenzhen, China; ^4^Dryland Crop Research Institute, Shao Yang Academy of Agriculture Science, Shaoyang, China

**Keywords:** purple-fleshed sweetpotato, anthocyanin biosynthesis, *R2R3-MYB*, transcriptome, multiple members, early response gene

## Abstract

*IbMYB1* was one of the major anthocyanin biosynthesis regulatory genes that has been identified and utilized in purple-fleshed sweet potato breeding. At least three members of this gene, namely, *IbMYB1-1*, *-2a*, and *-2b*, have been reported. We found that *IbMYB1-2a* and *-2b* are not necessary for anthocyanin accumulation in a variety of cultivated species (hexaploid) with purple shoots or purplish rings/spots of flesh. Transcriptomic and quantitative reverse transcription PCR (RT-qPCR) analyses revealed that persistent and vigorous expression of *IbMYB1* is essential to maintain the purple color of leaves and storage roots in this type of cultivated species, which did not contain *IbMYB1-2* gene members. Compared with *IbbHLH2*, *IbMYB1* is an early response gene of anthocyanin biosynthesis in sweet potato. It cannot exclude the possibility that other *MYBs* participate in this gene regulation networks. Twenty-two *MYB-like* genes were identified from 156 *MYBs* to be highly positively or negatively correlated with the anthocyanin content in leaves or flesh. Even so, the *IbMYB1* was most coordinately expressed with anthocyanin biosynthesis genes. Differences in flanking and coding sequences confirm that *IbMYB2s*, the highest similarity genes of *IbMYB1*, are not the members of *IbMYB1*. This phenomenon indicates that there may be more members of *IbMYB1* in sweet potato, and the genetic complementation of these members is involved in the regulation of anthocyanin biosynthesis. The 3′ flanking sequence of *IbMYB1-1* is homologous to the retrotransposon sequence of *TNT1-94*. Transposon movement is involved in the formation of multiple members of *IbMYB1*. This study provides critical insights into the expression patterns of *IbMYB1*, which are involved in the regulation of anthocyanin biosynthesis in the leaf and storage root. Notably, our study also emphasized the presence of a multiple member of *IbMYB1* for genetic improvement.

## Introduction

Anthocyanins are important secondary metabolites responsible for the development of colors, such as red and blue, in plants. These pigments are widely distributed in organs, such as the roots, stems, leaves, flowers, and fruits, and they are involved in responses to environmental and developmental cues (Albert et al., 2014). In sweet potato [*Ipomoea batatas* (L.) Lam], the color of the storage root is highly variable, ranging from white to deep purple, occasionally with purplish stain, and the colors of stems and leaves can also range from purple to green, or both colors on the same plant, depending on their age (Bradshaw, 2010). Colorful sweet potato vines have long been used as a classic “spiller” in containers, baskets, and along beds and borders. Their vigor, growth rate, and impactful garden color have long been appreciated (Armitage and Garner, 2001). The morphological diversity of anthocyanin accumulation has been widely utilized in ornamental horticulture and by pigment production industries (Bradshaw, 2010; Tanaka et al., 2017).

Gaining insights into the genetic factors affecting natural pigment variation in crops is crucial for breeders to alter the levels of anthocyanins during crop breeding (Tanaka et al., 2017; Appelhagen et al., 2018). Genetic mutations related to the anthocyanin biosynthesis pathway, in either structural or regulatory genes, often result in valuable unique color varieties. The *R2R3-MYB* transcription factors (TFs), basic helix-loop-helix (bHLH), and TTG1 (WD-Repeats) form a ternary MYB-bHLH-WDR (MBW) complex, which regulates the expression of structural genes involved in anthocyanin biosynthesis (Ramsay and Glover, 2005; Dubos et al., 2008; Albert et al., 2014; Xu et al., 2015). This MBW complex represents a central mode of gene regulation in the anthocyanin pathway (Feller et al., 2011). Most of the enzyme-coding genes of the anthocyanin pathway are positively regulated by the MBW complex (Carey et al., 2004). The change in coordinately expressed enzyme-coding genes is most likely caused by the variation of TFs in the MBW complex. It provides a common model for understanding the genetic basis of color mutants (Zhu et al., 2015; Sun et al., 2018). The co-expression network analysis (WGCNA) (Langfelder and Horvath, 2008) and the knowledge-based identification of pathway enzymes (KIPEs) (Pucker et al., 2020) based on the genome-wide and transcriptome-wide analyses have been applied to identify the tissue-specific regulation of anthocyanin biosynthesis (Tombuloglu et al., 2013; Liu et al., 2018, 2020; Iorizzo et al., 2020; Qin et al., 2020; Zhang et al., 2020; Chen et al., 2021; Parra-Galindo et al., 2021).

The MYBs include highly conserved MYB repeats (1R, R2R3, 3R, and 4R) in the N-terminus and have diverse C-terminal sequences that provide the protein with a wide range of functions (Stracke et al., 2001; Zimmermann et al., 2004; Du et al., 2012; Stracke et al., 2014; Liu et al., 2015). Gene duplication, segmental duplication, allelic variation, and genome doubling make the genetic mechanism of MYBs in polyploid crops more complex than that in diploid crops, and the number of *MYB* genes is redoubled in polyploid crops (Feller et al., 2011; Tombuloglu et al., 2013; Strygina and Khlestkina, 2019; Chen-Kun Jiang, 2020; Li C. et al., 2020; Li Y. et al., 2020; Liu et al., 2020). *IbMYB1*, *IbMYB2s*, and *IbMYB2* belong to the different subgroups of *R2R3-MYBs* that are involved in anthocyanin biosynthesis (Mano et al., 2007; Deng et al., 2020). *IbMYB1* was classified into *IbMYB1-1* and *IbMYB1-2* members. *IbMYB1-1* was considered to be a pseudogene due to variations in its promoter sequence (Tanaka et al., 2011). *IbMYB1-2* was further classified into two isoforms, namely, *IbMYB1-2a* and *IbMYB1-2b*. At least four isoforms of *IbMYB2s* were cloned and named *IbMYB2-1* to *IbMYB2-4*. The transcript sequences and amino acid sequences of *IbMYB1* and *IbMYB2*s were similar, but the biological function of *IbMYB2s* is currently unclear (Mano et al., 2007).

*R2R3-MYBs* such as *IbMYB1* can independently initiate the transcription of anthocyanin biosynthesis genes (Stracke et al., 2007; Dubos et al., 2008; Dong, 2014; Dong et al., 2014) or can interact with other TFs to form the MBW complex to activate anthocyanin biosynthesis in sweet potato and other plants (Dubos et al., 2008; Davies et al., 2012; Zhu et al., 2015; Deng et al., 2020; Yan et al., 2021). It has been confirmed that *IbMYB1-2* plays a vital regulatory role in anthocyanin accumulation in storage roots (Tanaka et al., 2011), but it is not necessary in all varieties for the expression of *IbMYB1* (Kim et al., 2010; Li et al., 2019; Zhang et al., 2020). A major locus of chromosome 5, where *IbMYB1* genes are located, has been shown to have a large effect on the color variation of shoots (Chen et al., 2021). We speculated that more *IbMYB1* members with genetic complementation may be involved in the regulation of anthocyanin biosynthesis in cultivated species. A variety of cultivars, which present a typical color variant of purple leaf in the shoots or purplish rings/spots in the storage root, were used in this study to identify the genetic mechanism of multiple members of *IbMYB1* related to the diversity of anthocyanin accumulation by the gene expression analysis.

## Materials and Methods

### Plant Materials

Nine sweet potato cultivar species, namely, “Zhezi No.1,” “Purple leaf,” “D7^∗^CIP1,” “Zibai,” “Xiangshu99,” “Huazi,” “Yidianhong,” “XCS No.2,” and “19-Z1-1,” were used in this study. “Zhezi No.1” was obtained from the Zhejiang Academy of Agricultural Sciences, China. “Purple leaf” is a traditional crop cultivar (i.e., landrace) from the field gene bank of the Crop Research Institute, Hunan Academy of Agricultural Sciences, China. “D7^∗^CIP1” and “19-Z1-1” are hybrid strains from the Hunan Academy of Agricultural Sciences. “Zibai” is a cultivar named “Aozhou Zibai” in the market of China. “Xiangshu99” is a bud mutation material from “Zhezi No.1.” “Huazi” is a landrace from the Hunan Academy of Agricultural Sciences. “Yidianhong” is a cultivar in the market of China. “XCS No.2” is a cultivar from the Hunan Academy of Agricultural Sciences.

All cultivars were transplanted on May 15^th^, 2018, May 10^th^, 2019, and May 20^th^, 2020, at the experimental station located at the Crop Research Institute, Hunan Academy of Agricultural Sciences, Changsha, China (28.2021° N, 113.0968° E). The characteristics of the plant samples were recorded before sampling on August 22^nd^, 2018, August 19^th^, 2019, and August 28^th^, 2020. Plants were subjected to natural light conditions and temperatures ranging from 18°C to 35°C in the same field. The sampled plants grew healthily, without any obvious disease or insect pests. All samples for DNA or RNA extraction were collected, immediately frozen in liquid nitrogen, and stored at −80°C for subsequent studies.

The apical tip leaf was collected as the top leaf, and the seventh leaf from the top leaf along each vine was sampled as the mature leaf. Six to seven nodes of the stem along each vine from the top leaf were sampled as stems. The leaves of “Yidianhong” and “19-Z1-1” were sampled based on the development stages. Three leaves from the same development stage were combined as one sample. These samples were named T1–T7 based on the development stage from the top leaf to the seventh leaf. The storage root samples were peeled before freezing in liquid nitrogen, and the storage roots of “Yidianhong” were divided into two parts based on color. Samples from three individual plants of the same variety at the same stage were ground into a fine powder in liquid nitrogen and combined as one sample.

### Pigment Observation and Measurement

The colors of leaves, stems, and storage roots were observed and recorded from healthy plants. The colors of leaves included purple, lilac (i.e., light purple), and green; stem color included purple and green; and flesh color was allocated as either purple, speckle purple (i.e., purple with purple rings and spots), or non-purple. The anthocyanin content was determined according to the previously described method (Drumm-Herrel and Mohr, 1982). Five samples were tested as biological replicates. Data Processing System v9.50 and Tukey’s method for multiple comparisons were used for the statistical analysis.

### Genotype Analysis of Major Genes Related to Anthocyanin Biosynthesis

Specific primers for *IbMYB1*, *IbbHLH1*, *IbbHLH2*, *IbWDR*, and *IbANS*, which are listed in Supplementary Table 1, were used for the genotype analysis following a previous study (Mano et al., 2007; Tanaka et al., 2011). The primer specificity of IbMYB1sF/IbMYB1sR and IbMYB2sF/IbMYB2sR was verified by the high-resolution melting and amplification efficiency of qPCR (Supplementary Figure 1). Primers for the genotype analysis, such as MYB2s-14R and MYB2s-23R, were synthesized according to the specificity of *IbMYB2s* sequences using an amplification refractory mutation system (Newton et al., 1989). The annealing temperature for all reactions was 54°C, the extension time was or 30 s – 6 min according to the length of the amplified fragment, and the number of cycles was 30. A 1.2% agarose gel was used for the genotype analysis.

### Construction of RNA-Seq *Library*, *Processing* of *Short R*eads, and *Assembling* of *Transcripts*

RNA sequencing was performed by Meiji Biomedical Technology Co., Ltd. (Shanghai, China). The RNA sequencing (RNA-Seq) library was prepared following the protocol of the manufacturer using the TruSeq RNA Sample Preparation Kit from Illumina (San Diego, CA, United States) using 1 μg of total RNA. In brief, the synthesized cDNA was subjected to end-repair, phosphorylation, and “A” base addition. Fragments with a length of approximately 300 bp were selected and linked to sequencing adapters. After quantitation using a Qubit Flex fluorometer, the paired-end RNA-Seq library was sequenced on an Illumina NovaSeq 6000 sequencer (2 × 150 bp read length).

Transcripts were assembled using Trinity software (v2.8.5) in *de novo* mode with parameters “–seqType fq –max_memory 250G –CPU 60” (Grabherr et al., 2011). Raw paired-end reads were trimmed using Trim Galore (v0.6.4_dev),^1^ which is a wrapper tool around Cutadapt^2^ (Martin, 2011) and FastQC^3^. The functional annotations of all transcripts were accomplished using the Trinotate pipeline^4^. Only *IbMYB2* transcript sequences could be retrieved from ipoBat4.transcript.fa of *I. batatas* Genome Browser,^5^ so we updated the transcript sequences of *IbMYB1* based on the *de novo* assembled transcripts and the Sanger sequencing results. All *IbMYB1* and *IbMYB2s* transcripts were verified by Sanger sequencing.

### Identification of Enzyme-Coding Genes and MBW Complex TF Genes

The amino acid sequences of MYBs, bHLH, and WDR from *Ipomoea nil*, *Ipomoea triloba*, *Ipomoea trifida*, and *Arabidopsis thaliana* were obtained by a BLAST search at http://sweetpotato.plantbiology.msu.edu/. The knowledge-based identification of pathway enzymes (KIPEs) (Pucker et al., 2020) based on peptide sequences was used to identify genes related to anthocyanin synthesis. The amino acid sequences used for gene identification are listed in Supplementary Table 2. The amino acid sequences of MYBs in Supplementary Table 3 were used for alignment, and an unrooted phylogenetic tree was constructed using CLUSTALW 2.1^6^ (Huerta-Cepas et al., 2016) with default parameters of FastTree (Price et al., 2009). To investigate the alternative splicing in the *IbMYB2s*, all reads generated in the RNA-Seq were aligned to the reference genome sequence of ‘‘Taizhong No.6’’ using HISAT2 v2.2.1^7^ with default parameters and visualized in IGV v2.8.2^8^.

### Differentially Expressed Gene Analysis in Transcriptome Sequencing

The abundance of each transcript in the RNA-Seq sample was quantified using Salmon v1.2.1 (Patro et al., 2017) with the updated reference transcripts of ipoBat4.transcript.fa. The expression levels of the transcripts were then compared between different groups (purple leaf vs. non-purple leaf, purple flesh vs. non-purple flesh, purple flesh, and leaf vs. non-purple flesh and leaf) in the R package DESeq2 (v1.26.0) with the calculated read number in the previous step (Love et al., 2014). Transcripts with *Q*-values less than 0.05 and fold change (FC) higher than 2 (| log_2_FC| > 1) were declared as significant differentially expressed genes (DEGs). A heatmap of some representative transcripts was generated using the R package “pheatmap (v1.0.12)” (Kolde, 2015), and the logarithm of the value of transcripts per million (FPKM) based on 2 was used in the pheatmap. Two varieties with the same shoot characteristics were assumed as pseudoreplicates, and the three storage root samples from the same varieties were tested as biological replicates for transcriptome sequencing.

Pearson’s correlation coefficient between gene expression and anthocyanin content in leaves and storage roots was calculated using the R package “psych 2.1.3” (Revelle, 2013). The value of the correlation coefficient (>0.7 or < −0.7) and the gene expression value of FPKM (>1.0) were set to filter the genes that were highly correlated with anthocyanin accumulation. The co-expression analysis identified numerous potential interactive regulators of anthocyanin biosynthesis, such as 22 MYBs, 2 bHLHs, 2 WD-repeats, and 31 biosynthesis genes. Spearman’s correlation coefficients among the main genes related to anthocyanin accumulation were calculated in the leaves and storage roots using the R package “psych 2.1.3.”

### Gene Expression in Different Color Tissues by Quantitative Reverse Transcription PCR Analysis

Total RNA was extracted from samples that were ground into powder under liquid nitrogen using the TRIzol method (Tiangen Biotech Co., Ltd., Beijing, China) and then examined by electrophoresis. First-strand cDNA was synthesized using HiScript III RT SuperMix for qPCR with gDNA wiper (Vazyme Biotech Co., Ltd., Nanjing, China). The quantitative reverse transcription PCR (RT-qPCR) was performed to determine the transcript levels of genes using AceQ SYBR qPCR Master Mix (Vazyme Biotech Co., Ltd.) and a real-time PCR system (LightCycler 960, Roche Diagnostics, Basel, Switzerland). The genes for the RT-qPCR amplification were confirmed to be closely related to the regulation of anthocyanin synthesis (Mano et al., 2007; Li et al., 2019; Deng et al., 2020). The primers for these genes were synthesized according to a previous study (Deng et al., 2020) and are listed in Supplementary Table 1. All primer combinations span an intron, or the primers span an exon-exon junction from the reference genome sequence (Yang et al., 2017).

The relative mRNA levels of genes related to anthocyanin synthesis in this study were calculated by normalizing the quantification (Pfaffl, 2001) to the geometric mean of three housekeeping genes, namely, *18S rRNA*, *TUA*, and *ACT* (GuoLiang et al., 2020). Three samples were tested as biological replicates for RT-qPCR. GraphPad Prism 8.3.0 and one-way ANOVA were used for the statistical analysis.

The RT-qPCR was performed for the validation of the transcriptome data (Supplementary Figure 2). The same samples were used for RT-qPCR and RNA-seq analysis, three biological replicates for each group and three repetitions for each sample. The expression level of the reference gene across 15 RNA-Seq samples was calculated for Pearson’s correlation analysis. The expression levels of four anthocyanin biosynthesis genes (i.e., *IbCHS-D*, *IbCHI*, *IbDFR-A*, and *IbANS*) from the same tissues (i.e., six leaf samples and nine flesh samples) were calculated for the Pearson’s correlation analysis between RT-qPCR and RNA-Seq. The values of log_2_FPKMs were used in all the analyses.

### Gene Cloning of *IbMYB1* and *IbMYB2s* From Cultivated Species

*IbMYB1* and *IbMYB2s* genes were cloned from the cDNA of leaves using the FastKing RT SuperMix (Tiangen Biotech Co., Ltd., Beijing, China) for DNA removal. The primers for *IbMYB1* and *IbMYB2s* cloning are listed in Supplementary Table 1. The PCR products were recovered for T-vector cloning and Sanger sequencing.

The DNA was extracted from the leaves using a modified hexadecyltrimethylammonium bromide (CTAB) DNA extraction procedure (Gawel and Jarret, 1991). The flanking sequences of *IbMYB1* and *IbMYB2s* were cloned using fusion primers and nested integrated PCR (FPNI-PCR) (Wang et al., 2011). The target primers of *SP1*, *SP2*, *SP3*, and *MYB2RepR* were used for the first FNPI-PCR, and the target primers of *pB9R4*, *pB9R3*, *pB9R2*, and *pB9R1* were used for the second FNPI-PCR to clone the 5′ flanking sequence of *IbMYB2s*. The target primers of *MYB2RepF*, *MYB2-4F2*, and *MYB2-4F3* were used for the 3′ flanking sequence cloning of *IbMYB2s.* The target primers of *3UTR-F1*, *3UTR-F2*, and *Pro MF M* were used for the 3′ flanking sequence cloning of *IbMYB1.* The fusion arbitrary degenerate primers FP1, FP3, and FP5 were used for non-target sequence binding. The primers FSP1 and FSP2 were used as nested integrated primers. All primer sequences are listed in Supplementary Table 1. The PCR products were recovered by agarose electrophoresis for Sanger sequencing.

Nucleotide sequences were analyzed using ClustalW 2.1. The InDels of flanking sequences and gene sequences were drawn using ESPript3.0^9^ (Xavier and Patrice, 2014). The genomic sequences of the cultivar “Taizhong No.6” (Yang et al., 2017) and the wild species *I. trifida* and *I. triloba* (Wu et al., 2018) were used in this study.

## Results

### Anthocyanin Accumulation Polymorphisms Are Mainly Caused by the Genotype Difference of *MYBs* in Sweet Potato

**FIGURE 1:**
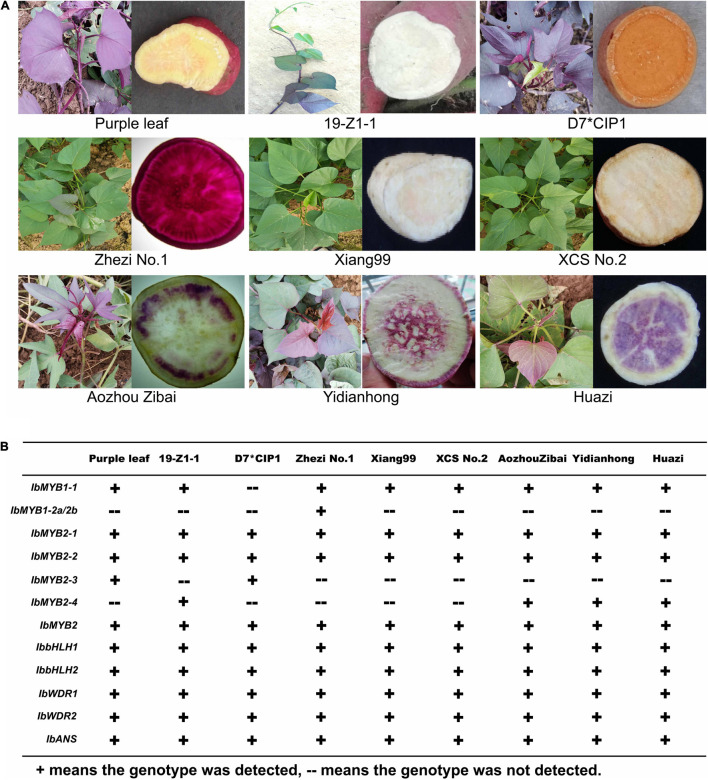
Anthocyanin accumulation polymorphisms and genotype polymorphisms in nine sweet potato cultivars. **(A)** Anthocyanin accumulation polymorphisms of nine sweet potato cultivars. **(B)** Genotype polymorphisms related to anthocyanin biosynthesis in nine sweet potato cultivars.

The cultivars presented abundant morphological diversity in anthocyanin accumulation (Figure 1A). Anthocyanins mainly accumulated in the shoots of “Purple leaf,” “D7^∗^CIP1,” and “19-Z1-1.” The storage roots showed a typical color variant; for example, “Zibai” was white with purple rings, “Huazi” was white with irregular purple plaque, and “Yidianhong” was white with a purple spot in the center. “Xiangshu99” and “XCS No.2” were used as negative controls, with non-purple flesh and green shoots. “Zhezi No.1” is a purple-fleshed cultivated species, which was used as the positive control for the major accumulation of anthocyanins in the storage root. The anthocyanin content among different cultivars was related to the intensity of the purple pigment and varied greatly in different tissues of the same strain (Supplementary Figure 3).

The major enzyme-coding genes and MBW TFs that evolved in anthocyanin biosynthesis were detected by specific molecular markers in these cultivars (Figure 1B). *IbANS*, *IbbHLH1*, *IbbHLH2*, *IbWDR1*, *IbWDR2*, and *IbMYB2* were detected in all the cultivars, indicating that these genes may not account for this color variation. The *IbMYB1* and *IbMYB2s* members showed large differences among these cultivars. *IbMYB1-2a* and *-2b* are unique to purple-fleshed sweet potatoes because they can only be detected by specific molecular markers in some purple-fleshed cultivar species (Tanaka et al., 2011). However, it could not be detected in purple-fleshed cultivars such as “Zibai,” “Huazi,” and “Yidianhong,” whose flesh colors are purple-stained with rings/spots. *IbMYB1-2a* and *-2b* are not necessarily purple leaves. *IbMYB2-3* and *IbMYB2-3*, the members of *IbMYB2s*, seem to be more associated with color variation in these cultivars.

### The Key Enzyme-Coding Genes of the Anthocyanin Biosynthesis Pathway Were Coordinately Expressed With Anthocyanin Accumulation

**FIGURE 2:**
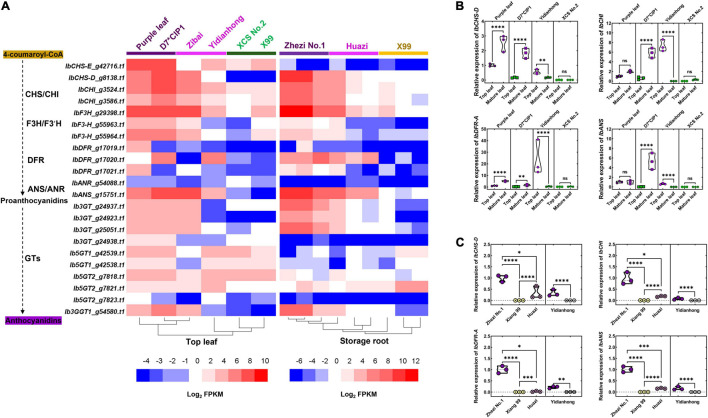
Gene expression difference of enzyme-coding genes related to anthocyanin biosynthesis. **(A)** Heatmap of differentially expressed genes (DEGs) related to enzyme-coding genes in leaf and storage root by RNA sequencing (RNA-Seq). The color of the variety name and the lower horizontal line represents the color of the sample. **(B)** Enzyme-coding gene expression comparisons between top leaf and mature leaf of varieties by RT-qPCR. **(C)** Enzyme-coding gene expression comparisons between different colors of flesh by RT-qPCR. The purple flesh of “Yidianhong” and the non-purple flesh of “Yidianhong” were analyzed. The color of jitter in the violin plot represents the color of the corresponding sample. *P < 0.0332, **P < 0.0021, ***P < 0.0002, and ****P < 0.0001; ns indicates *P* > 0.1234. *n* = 3.

The RNA-Seq was performed to quantify the expression of genes in the flesh and leaves of varieties that varied in color. The number of DEGs between purple and non-purple storage roots was much larger than that between purple and non-purple leaves (Supplementary Figure 4). We focused on the DEGs related to the anthocyanin biosynthesis pathway. Notably, 13 *IbPALs*, 3 *IbC4Hs*, 1 *Ib4CL*, 2 *IbCHSs*, 2 *IbCHIs*, 1 *IbF3H*, 2 *IbF3′Hs*, 3 *IbDFRs*, 1 *IbANS*, 1 *IbANR*, and 10 anthocyanin glucosyltransferase genes were identified in the *de novo* assembled transcripts by KIPEs. An apparent correlation between anthocyanin biosynthesis genes and pigment sedimentation was observed (Figure 2A). The anthocyanin biosynthesis genes such as *IbF3H*, *IbF3*′*H*, *IbCHI*, *IbCHS-D*, *IbDFR*, *IbANS*, and *Ib3GT* demonstrate the coordinated expression with anthocyanin accumulation in leaves and storage roots. These genes were significantly upregulated in purple tissues and were expressed at very low levels in the non-purple tissues. The anthocyanin glucosyltransferase genes such as *Ib5GT1* and *Ib5GT2* were mainly expressed in the leaves but not in the flesh.

To confirm the validity of the DEGs identified by transcriptome sequencing, the RT-qPCR was performed. The gene expression change trend in RNA-Seq from different tissues was highly consistent with RT-qPCR, either among internal reference genes or among gene regulatory modules of anthocyanin biosynthesis (Supplementary Figure 2). The DEGs were responsible for the observed color variation in the leaf of “D7^∗^CIP1,” “Yidianhong,” and the storage root of “Huazi” and “Yidianhong.” The expression of key enzyme-coding genes, *IbCHI*, *IbCHS-D*, *IbDFR*, and *IbANS*, changed with purple color variation in the leaves of the same variety (Figure 2B). The expression pattern of key enzyme-coding genes in purple flesh of “Huazi” and “Yidianhong” coincided well with the change of purple color in flesh, even in the same variety (“Yidianhong”) of flesh with different colors (Figure 2C). This expression pattern in the flesh of “Huazi” was almost similar to the purple-fleshed storage root of “Zhezi No. 1” (Figures 2A,C).

### *IbMYB1* and *IbbHLH2* of MBW Complex Work in the Most Coordination With the Key Enzyme-Coding Genes of the Anthocyanin Biosynthesis Pathway

**FIGURE 3:**
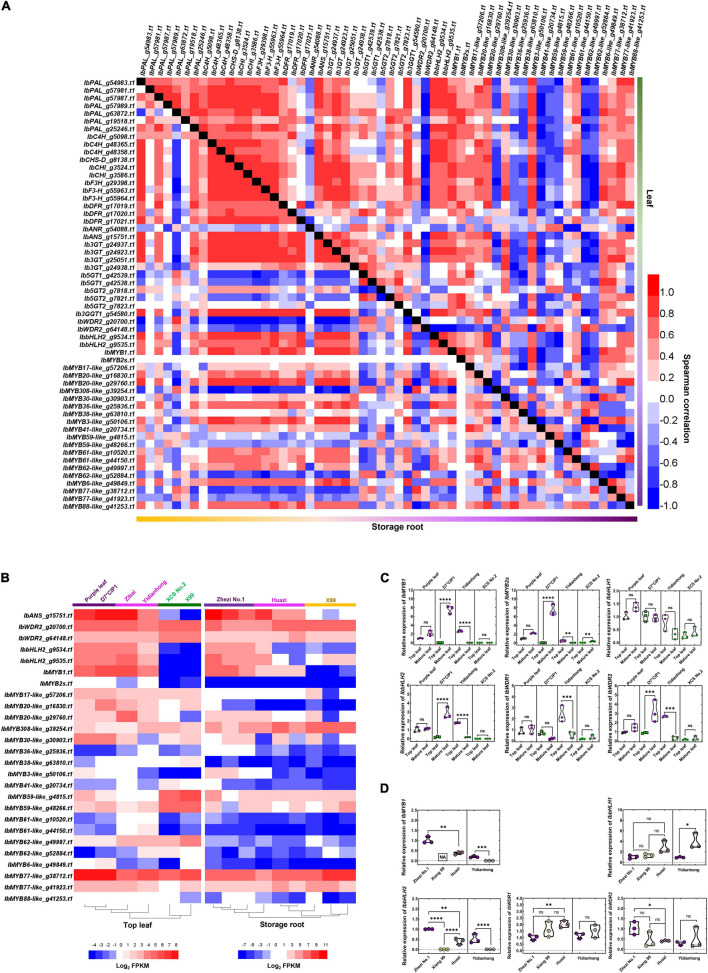
Transcription factor (TF) expression difference of MYB-bHLH-WDR (MBW) complex related to anthocyanin biosynthesis. **(A)** Spearman’s correlation coefficient among the main genes related to anthocyanin accumulation in leaf and storage root by RNA-Seq. The triangle at the bottom represents the correlation of storage root, and the upper triangle represents the correlation of leaf. **(B)** Heatmap of DEGs related to MBW complex TFs in leaf and storage root by RNA-Seq. The color of the variety name and the lower horizontal line represents the color of the sample. **(C)** MBW complex TFs expression comparisons between top leaf and mature leaf of varieties by RT-qPCR. **(D)** TFs expression comparisons between different colors of flesh by RT-qPCR. The purple flesh of “Yidianhong” and the non-purple flesh of “Yidianhong” were analyzed. The color of jitter in the violin plot represents the color of the corresponding sample. *P < 0.0332, **P < 0.0021, ***P < 0.0002, and ****P < 0.0001; ns indicates *P* > 0.1234. *n* = 3. “NA” in the box indicates that gene expression was not detected in the sample.

The change of coordinately expressed enzyme-coding genes is most likely caused by the variation of TFs in the MBW complex. *IbWDR2* (*g20700.t1*), *IbWDR2* (*g64148.t1*), *IbbHLH2* (*g9534.t1*), and *IbbHLH2* (*g9535.t1*), which are the potential members of the MBW complex that participates in the regulation of anthocyanin biosynthesis, were chosen to evaluate the expression of the MBW complex. *IbbHLH2* was coordinately expressed with anthocyanin biosynthesis genes, either in the flesh or in the leaf. The expression of *IbWDR2* was negatively correlated with the enzyme-coding genes involved in anthocyanin biosynthesis (Figure 3A). *IbbHLH1* and *IbbWDR1* showed no association with color variation in anthocyanin accumulation, although they were expressed at high levels in all tissues (Figures 3C,D).

The expression of *IbMYB1* was highly correlated with anthocyanin biosynthesis in both storage roots and leaves, although *IbMYB1-2a* and *-2b* members were not detected in these materials. A total of 156 *MYB-like* genes were identified using the KIPEs method (Supplementary Figure 5). The expression patterns of *MYBs* in storage roots and leaves were quite different (Supplementary Figure 6). Of note, 22 *MYB-like* genes were found to be highly positively or negatively correlated with the anthocyanin content in leaves or flesh. More TFs may be involved in the anthocyanin accumulation regulatory network in leaves than in flesh. The expression of *IbMYB1*, *IbMYB6-like* (g49849.t1), *IbMYB88-like* (g41253.t1), and *IbMYB3-like* (g50106.t1) were positively correlated with anthocyanin accumulation in both leaves and flesh (Supplementary Figure 7). Even so, *IbMYB1* was most coordinately expressed with anthocyanin biosynthesis genes. The expression of *IbMYB1* was almost undetectable by RNA-Seq (Figure 3B) and RT-qPCR (Figure 3D) in the flesh of “Xiangshu99,” as “Xiangshu99” is a bud mutation material from “Zhezi No.1,” and the *IbMYB1-2a* and *-2b* members were deleted. *IbMYB2s* were mainly expressed at low levels in purple leaves (Figures 3B,C) but not expressed at all in purple root flesh (Figure 3D).

### Co-expression Patterns of Key Genes at Different Developmental Stages of Leaf

**FIGURE 4:**
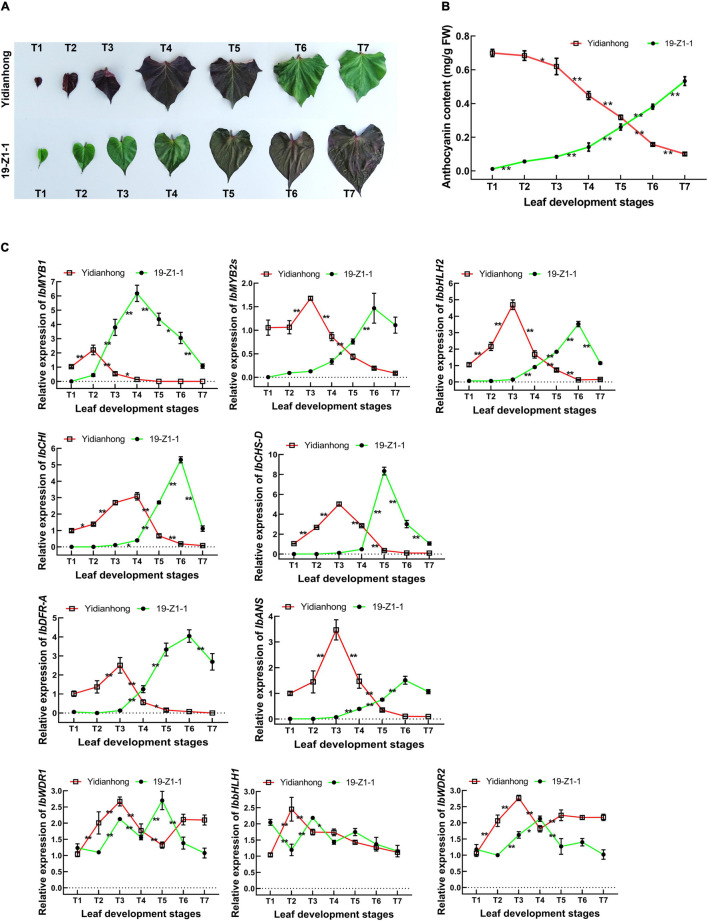
Gene expression of anthocyanin biosynthesis was synchronous with the change of anthocyanin content in leaves. **(A)** Purple changes in the leaves of “19-Z1-1” and “Yidianhong”. **(B)** Anthocyanin content of leaves changed with the color depth of purple during the leaf development stage. *P < 0.05 and **P < 0.01, *n* = 5. **(C)** Gene expression changes of anthocyanin biosynthesis by RT-qPCR. *P < 0.05, **P < 0.01, *n* = 3.

To further determine the co-expression pattern between TFs and enzyme genes, the gene expression in the leaves of “19-Z1-1” and “Yidianhong” was analyzed by RT-qPCR. The leaf color of “19-Z1-1” changed from green to purple during leaf development, while the leaf color of “Yidianhong” presented purple fading from the top leaf to the mature leaf (Figure 4A). The anthocyanin content of leaves changed with the color depth of purple, and the significant change of anthocyanin accumulation in leaves mainly occurred in the development stages of T3-T6 (Figure 4B). The expression of key genes of anthocyanin biosynthesis was evaluated by RT-qPCR, and the expression difference was analyzed at the leaf developmental stages of T1-T7 (Figure 4C). The expression of *IbMYB1*, *IbMYB2s*, *IbbHLH2*, *IbCHI*, *IbCHS-D*, *IbDFR-A*, and *IbANS* was mainly changed significantly at the T3-T5 stage, and it was coordinately expressed with the change of anthocyanin content. The expression changes of *IbMYB2s* and *IbbHLH2* were consistent with that of enzyme-coding genes. The significant change of *IbMYB1* gene expression was presented at the development stage of T2-T3. Its expression change was a development stage earlier than that of the other genes. In view of the initial role of the *IbMYB1* gene in the MBW TF complex, it is reasonable to speculate that *IbMYB1* is an initial response gene of anthocyanin biosynthesis. Therefore, the persistence and intensity expression level of *IbMYB1* is still the key to the regulation of anthocyanin biosynthesis in the leaf.

### More Members of *IbMYB1* Are Essential for Its Gene Expression in Sweet Potato

**FIGURE 5:**
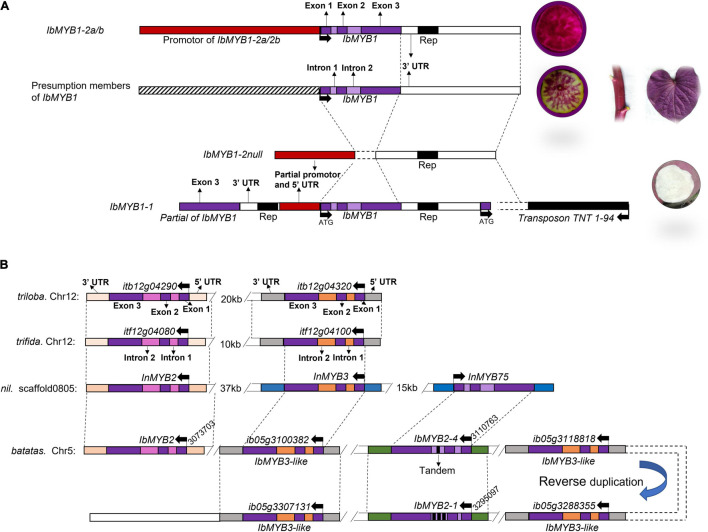
Flanking and coding sequences difference of *IbMYB1* and *IbMYB2s* in sweet potato. **(A)** Sketch map of *IbMYB1* members and their flanking sequence differences. **(B)** Sketch map of *MYB2* homologous locus on the reference genome of *Ipomoea triloba*, *Ipomoea trifida*, *Ipomoea nil*, and *Ipomoea batatas*. The same color boxes represent highly homologous sequence segments. This model assumes that the members of *IbMYB2s* are the copy number variants arranged in a tandem array in the haplotype genome. The actual distribution of these genotypes may be parallel, parallel with tandem arrays coexisting in hexaploidy, or even more complex. However, we have not obtained enough genetic data to distinguish these possible arrangements in this study.

The functional complementation between different members of *IbMYB1* may be the main reason why *IbMYB1-2* members are not necessary for *IbMYB1* expression in all cultivated species (Figure 5A). It cannot exclude the possibility that *IbMYB1-1* also retained the ability of transcription and expression but not a pseudogene. A retrotransposon sequence (Accession No: MW819731), which is homologous to the sequence of retrovirus-related polyprotein from transposon *TNT1-94* (Accession No: PKA53352), was cloned from the 3′ flanking sequence of *IbMYB1-1*. This retrotransposon had lost independent transposon activity due to sequence mutations (Supplementary Figure 8). However, due to the interference of *IbMYB1-1*, we did not clone the hypothetical members of *IbMYB1* by FPNI-PCR. The reaction conditions of FPNI-PCR should be optimized in the follow-up work, and “D7^∗^CIP1” may be an ideal research material because it does not carry any of the known members of *IbMYB1* (*IbMYB1-1*, *-2a*, and *-2b*).

The phylogenetic analysis based on amino acid sequences showed that *IbMYB2s* are the *MYBs* genes with the highest similarity sequence with *IbMYB1* (Supplementary Figure 5). Except *IbMYB1*, the cDNA derived from two transcripts of *IbMYB2s*, i.e., *IbMYB2s-a* and *IbMYB2s-b*, was cloned from purple leaves (Supplementary Figure 9A). *IbMYB2s-b* may originate from the alternative splice variant of *IbMYB2s* that did not follow the GT-AG rule, as the alternative splicing site of *IbMYB2s* is the same as that of *IbMYB1* (Kim et al., 2010), but we did not find the transcripts of *IbMYB1-b*. The alignment of RNA-Seq reads around *IbMYB2s* locus in reference genome sequence (Taizhong No. 6) prefers to support that *IbMYB2s-b* may be transcribed by different members of *IbMYB2s* (Supplementary Figures 9B,C). The exon sequences of *IbMYB2s* are highly conserved in the *IbMYB1* family, and some single-nucleotide polymorphisms (SNPs) that are present in the gene sequences lead to variations in some amino acid residues in the amino acid sequence (Supplementary Figure 9D).

The flanking sequences of *IbMYB2s* were different from those of *IbMYB1-1* and *IbMYB1-2a/2b* (Figure 5B). The alleles of *IbMYB2s* were abundant in different gene isoforms/variants (Supplementary Figures 10, 11). The copy number variations of *IbMYB2s* were found on chromosome 5 from the reference genome sequence. At least seven gene members of the *R2R3-MYB* were identified, including *IbMYB2*, *IbMYB2-4*, and *IbMYB2-1*, as well as four gene members homologous to *InMYB3* at this locus (Figure 5B). However, no sequence that is homologous to the sequences of *IbMYB1* was found at this locus. Considering the expression pattern of these genes (Figures 2, 3), it confirmed that there may be more members of *IbMYB1* in sweet potato, rather than its homologous genes, such as *IbMYB2s* and *IbMYB2*, contribute to the function of genetic complementarity.

## Discussion

The regulation networks of anthocyanin accumulation in plants are complex. Other TFs involved in anthocyanin biosynthesis have been reported, such as WRKY (Li Y. et al., 2020), MADS-box (Jaakola et al., 2010), HY5 (Kim et al., 2017), and R3-MYB suppressor factor (Cao et al., 2017; Colanero et al., 2018). Many negative feedback regulation mechanisms may act to prevent the excessive accumulation of anthocyanins in sweet potato, such as the pretranscriptional and posttranscriptional regulation of miRNA (He et al., 2019) and the competitive inhibition of suppressor factors (Deng et al., 2020; Wei et al., 2020). *IbbHLH2* is also highly involved in the regulation of anthocyanin accumulation, according to a previous study and the *C-S-A* model that *C* encodes a R2R3-MYB transcription factor, *S* encodes a bHLH protein, and *A* encodes a dihydro-flavonol reductase (DFR) (Sun et al., 2018). The heterologous expression of *Lc* (*bHLH2*) promotes the anthocyanin accumulation in purple-fleshed sweet potato (Wang et al., 2016). However, there was no difference in the genotypes of *IbbHLH2* among these materials in this study. In addition, the low expression of *IbbHLH2* was detected in non-purple tissues, such as the flesh of “Xiang99.” A previous study has confirmed that the *IbbHLH2* gene is the downstream regulatory gene of *IbMYB1*, as the MBW complexes of IbMYB1/IbMYB2/IbMYB3-IbbHLH2-IbWDR1 activated the promoters of *IbbHLH2* (Deng et al., 2020). Therefore, the persistent and vigorous expression of *IbMYB1* is still the most crucial factor in maintaining the purple color of sweet potato.

Sweet potato is a highly heterozygous crop with 90 chromosomes (2*n* = 6*x* = 90), and its ploidy type has not yet been clearly determined (Yang et al., 2017; Wu et al., 2018; Gao et al., 2020). The hexaploidy, high heterozygosity, huge genome, and outcrossing nature make it difficult to analyze the genetics of multiple-copy genes. In wild species (morning glory, *I. nil*), *InMYB1* is expressed in flowers, and *InMYB2* is expressed in petioles, stems, and roots (Morita et al., 2006). Highly similarity sequences of *InMYB2* can be found in the reference genome sequence of cultivated (*I. batatas*) and other wild species, such as *I. triloba* and *I. trifida* (Figure 5B). Relative to wild species, *IbMYB2s* only exist in the cultivated species from the reference genome sequence, whereas no gene member of *IbMYB1* was found at this locus. We speculated that these two *IbMYB2s* may be a pair of homologous genes that originated from a homologous gene of *IbMYB2*. Regrettably, we could not determine the locus of *IbMYB1* in the reference genome sequence.

This research provides a useful reference for the identification and utilization of excellent genes in germplasms. Like the birth of the black rice gene (Oikawa et al., 2015), our research supports the hypothesis that functional variation of the promoter is mainly responsible for the incomplete expression of *IbMYB1s* in certain tissues. This variation can be caused by homologous recombination, insertion/deletion, transposon movement, and other events in the chromosome. Genetic variation caused by transposon movement is common in plants (Notani, 1991; Deragon et al., 2008; Bennetzen and Wang, 2014; Vicient and Casacuberta, 2017); it has been speculated that genetic heterogeneity, such as flesh color with purple rings/spots, is a type of chimera undergoing *IbMYB1-2a/2b* mutations, which is similar to the color variation of flowers in *Petunia hybrida* (van Houwelingen et al., 1998; Nakajima et al., 2005). Based on the sequence characteristics in the 3’ flanking region of *IbMYB1-1*, it is reasonable to assume that the occurrence of *IbMYB1s* is related to retrotransposon movement. *IbMYB1-2a/2b* have been easily deleted from the genome of many cultivated species such as “AYM96” (Tanaka et al., 2011), “Xiangshu 99,” and others (Ma et al., 2016). It may be the reason why so many members of *IbMYB1s* existed in sweetpotato. However, we do not have enough data to explain it.

## Data Availability Statement

The datasets presented in this study can be found in online repositories. The names of the repository/repositories and accession number(s) can be found below: The raw sequence data used in this study can be downloaded from the NCBI Short Read Archive No. PRJNA721067. Gene sequences with InDels, the 5′ and 3′ flanking sequences of *IbMYB2s*, and the 3′ flanking sequence of *IbMYB1* can be downloaded from the GenBank under accession numbers MW819719, MW819720, MW819721, MW819722, MW819723, MW819724, MW819725, MW819726, MW819727, MW819728, MW819729, MW819730, and MW819731.

## Ethics Statement

The authors declare that the experiments comply with the current laws of the country in which they were performed.

## Author Contributions

DZ, CZ, and NT designed the study. DZ, FD, YaZ, YH, QY, YiZ, XX, and XG performed the field experiments. ZZ added the required data. YT analyzed the data. DZ and YT wrote the manuscript. CZ funded the research project. All authors have read and approved the manuscript.

## Conflict of Interest

The authors declare that the research was conducted in the absence of any commercial or financial relationships that could be construed as a potential conflict of interest.

## Publisher’s Note

All claims expressed in this article are solely those of the authors and do not necessarily represent those of their affiliated organizations, or those of the publisher, the editors and the reviewers. Any product that may be evaluated in this article, or claim that may be made by its manufacturer, is not guaranteed or endorsed by the publisher.

## Footnotes

1
https://github.com/FelixKrueger/TrimGalore

2
http://journal.embnet.org/index.php/embnetjournal/article/view/200

3
https://qubeshub.org/resources/fastqc

4
https://github.com/Trinotate

5
http://public-genomes-ngs.molgen.mpg.de/sweetpotato/

6
https://www.genome.jp/tools-bin/clustalw

7
https://www.nature.com/articles/s41587-019-0201-4

8
https://www.nature.com/articles/nbt.1754

9
http://espript.ibcp.fr/ESPript/cgi-bin/ESPript.cgi

## Supplementary Material

Supplementary Figure 1 High-resolution dissolution curves of qPCR products of *IbMYB1* and *IbMYB2s* from cDNA and DNA.

Supplementary Figure 2 The RT-qPCR was performed for the validation of the transcriptome data. **(A)** Pearson’s correlation coefficient of reference gene between Actin and 18s RNA in leaf. **(B)** Pearson’s correlation coefficient of reference gene between Actin and 18s RNA in flesh. **(C)** Pearson’s correlation coefficient of reference gene between Actin and TUA in leaf. **(D)** Pearson’s correlation coefficient of reference gene between Actin and TUA in flesh. **(E)** Pearson’s correlation coefficient of reference gene between 18s RNA and TUA in leaf. **(F)** Pearson’s correlation coefficient of reference gene between 18s RNA and TUA in flesh. **(G)** Pearson’s correlation coefficient of the gene expression level in the regulatory module of anthocyanin biosynthesis between RNA-Seq and RT-qPCR.

Supplementary Figure 3 Anthocyanin concentration in different tissues of nine cultivars. Significant differences are indicated by the letters a, b, c, etc. *n* = 5, *P*-value < 0.05.

Supplementary Figure 4 The upSet plot of DEGs between purple and non-purple leaf, purple and non-purple storage root, and purple leaf and purple storage root.

Supplementary Figure 5 Evolutionary tree of *MYBs* from *Ipomoea batatas*, *Ipomoea nil*, *Ipomoea triloba*, *Ipomoea trifida*, and *Arabidopsis thaliana*. The evolutionary tree is the unrooted tree.

Supplementary Figure 6 Pearson’s correlation coefficient between anthocyanin concentration and *MYBs* expression in leaf and storage root.

Supplementary Figure 7 Pearson’s correlation coefficient between anthocyanin concentration and *MYBs* expression in leaf and storage root that were screened out with highly positive (negative) correlation.

Supplementary Figure 8 The retrotransposon sequence that cloned from the 3′ flanking sequence of *IbMYB1-1*.

Supplementary Figure 9 Transcript sequence of *IbMYB2s*. **(A)** *IbMYB1* and *IbMYB2s* were cloned from the cDNA of leaves by RT-PCR. **(B)** Alignment of RNA-Seq reads around *IbMYB2-4* locus in reference genome sequence (Taizhong No.6). **(C)** Alignment of RNA-Seq reads around *IbMYB2-1* locus in reference genome sequence (Taizhong No.6). **(D)** Coding sequence and amino acid sequence alignment among *IbMYB1*, *IbMYB2s-a*, and *IbMYB2s-b* gene members.

Supplementary Figure 10 InDels of 5′ flanking sequence of *IbMYB2s* gene. *IbMYB2s* is the 5′ flanking sequence that cloned from the genome in this study; *IbMYB2-1* and *IbMYB2-1* are the 5′ flanking sequences that obtained from the reference genome sequence.

Supplementary Figure 11 InDels of 3′ flanking sequence of *IbMYB2s* gene. 3′UTR1–3′UTR5 were the 3′ flanking sequences of *IbMYB2s* that cloned from the genome in this study; *IbMYB2-1* and *IbMYB2-4* were the sequences that reported in references.

Supplementary Table 1 List of all primers for PCR.

Supplementary Table 2 The amino acid sequence of the anthocyanin/flavonoid-related genes from the transcriptome data.

Supplementary Table 3 The amino acid sequence for the evolutionary tree of MYBs from *Ipomoea batatas*, *Ipomoea nil*, *Ipomoea triloba*, *Ipomoea trifida*, and *Arabidopsis thaliana*.
