# Supplementary material for: The Expression of IbMYB1 Is Essential to Maintain the Purple Color of Leaf and Storage Root in Sweet Potato [Ipomoea batatas (L.) Lam]
Source: Front Plant Sci. 2021 Sep 23;12:688707. doi: 10.3389/fpls.2021.688707 (PMC8495246; doi:10.3389/fpls.2021.688707)
Supplement: Supplementary Figure 1 — High-resolution dissolution curves of qPCR products of IbMYB1 and IbMYB2s from cDNA and DNA. [file Data_Sheet_1.PDF]

Fig S1

A

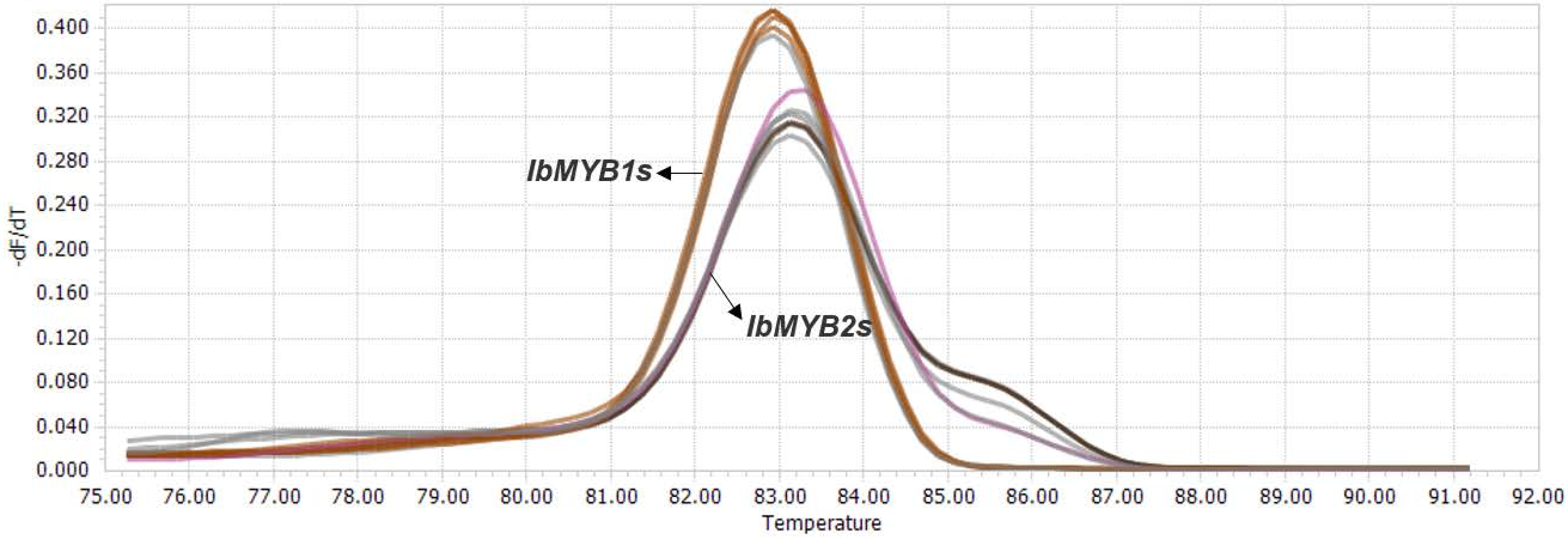

High resolution dissolution curve of amplified products using cDNA as template.

B

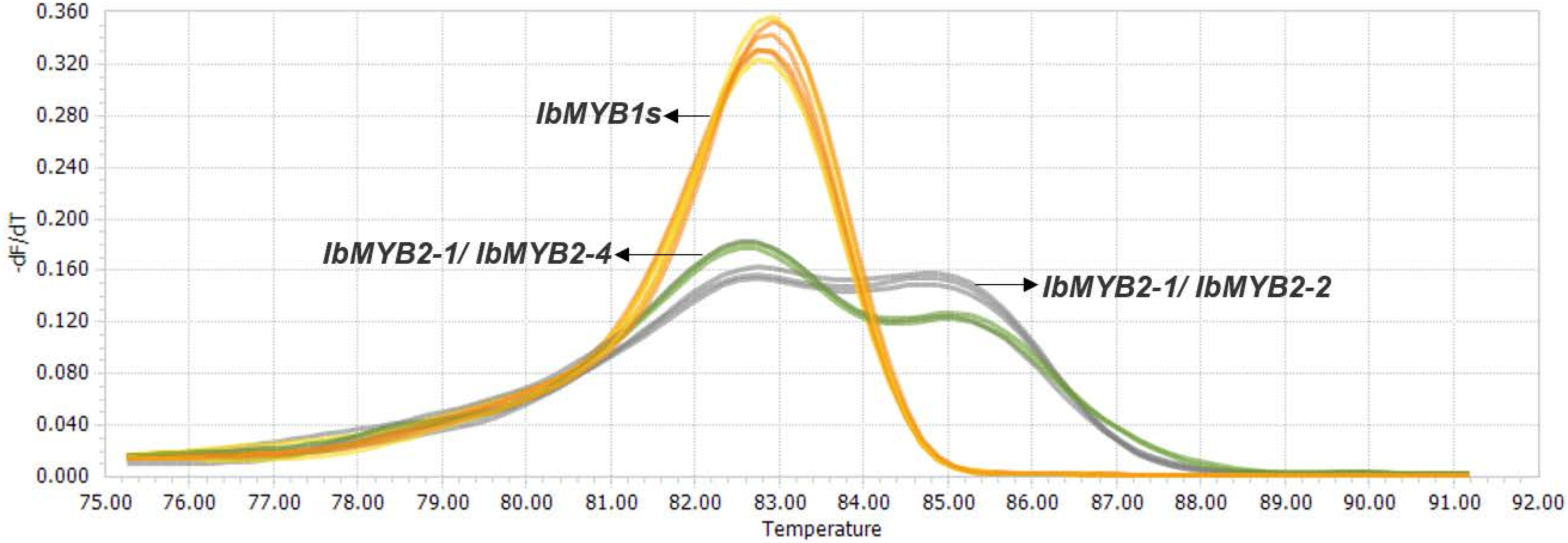

High resolution dissolution curve of amplified products using DNA as template.

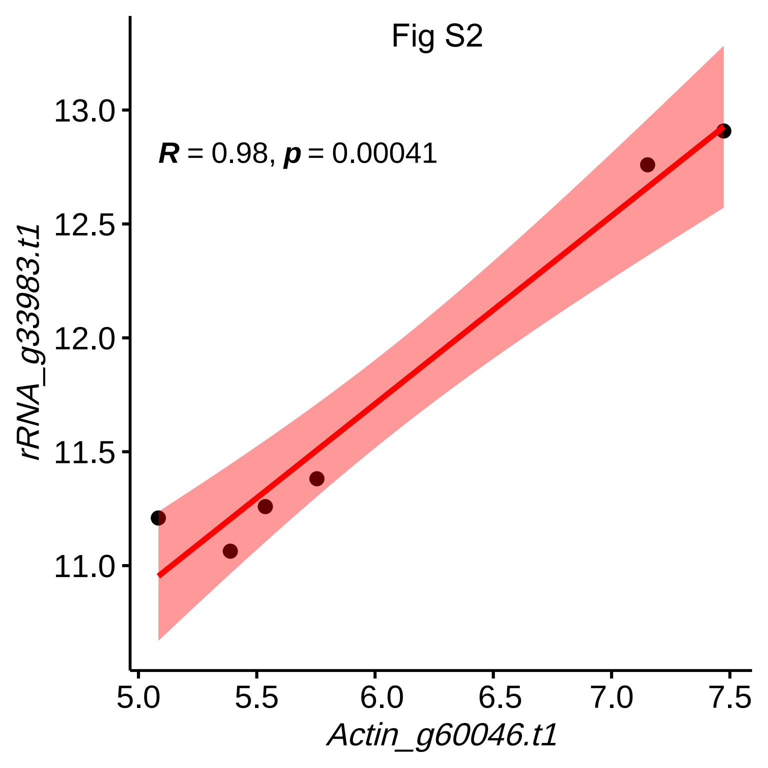
Fig S2
R = 0.98, p = 0.00041
rRNA_g33983.t1
13.0
12.5
12.0
11.5
11.0
5.0
5.5
6.0
6.5
7.0
7.5
Actin_g60046.t1

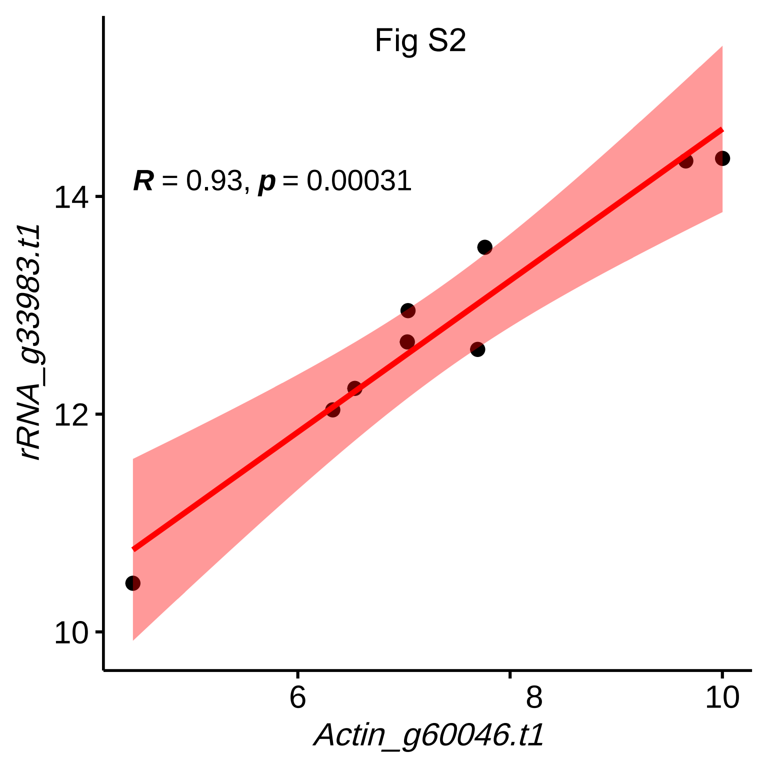

Fig S2
R = 0.93, p = 0.00031
14
12
10
rRNA_g33983.t1
6
8
10
Actin_g60046.t1

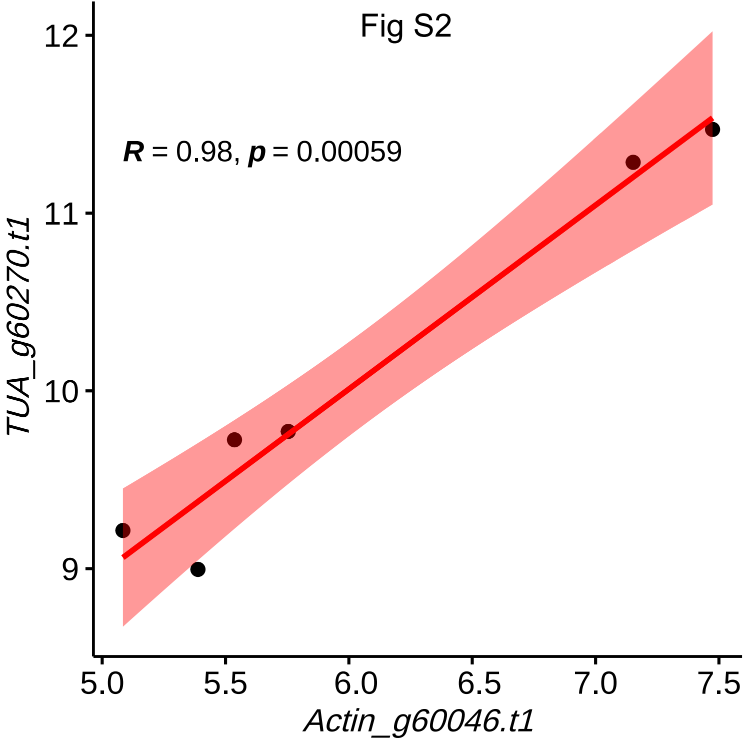
Fig S2
R = 0.98, p = 0.00059
TUA_g60270.t1
Actin_g60046.t1
12
11
10
9
5.0
5.5
6.0
6.5
7.0
7.5

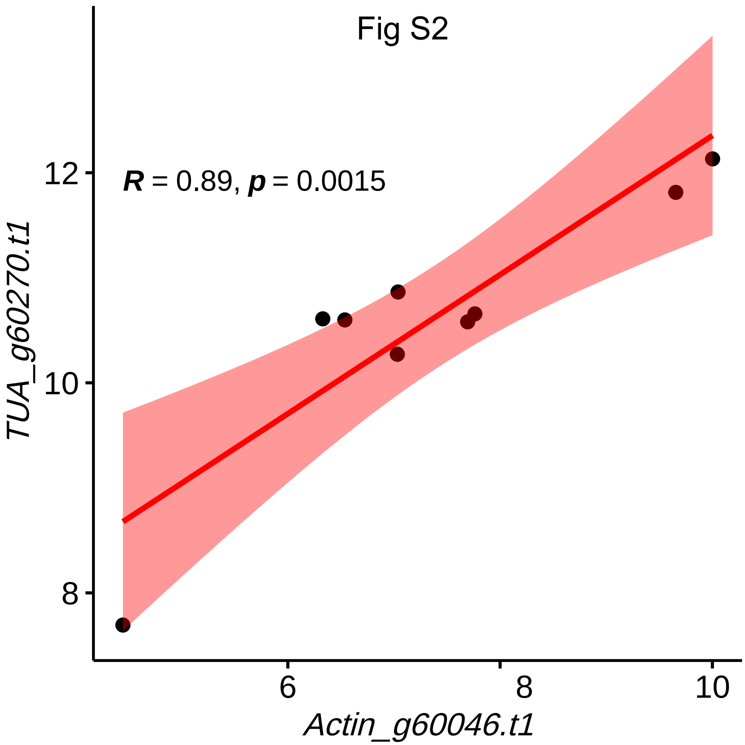

Fig S2
R = 0.89, p = 0.0015
12
10
8
TUA_g60270.t1
6
8
10
Actin_g60046.t1

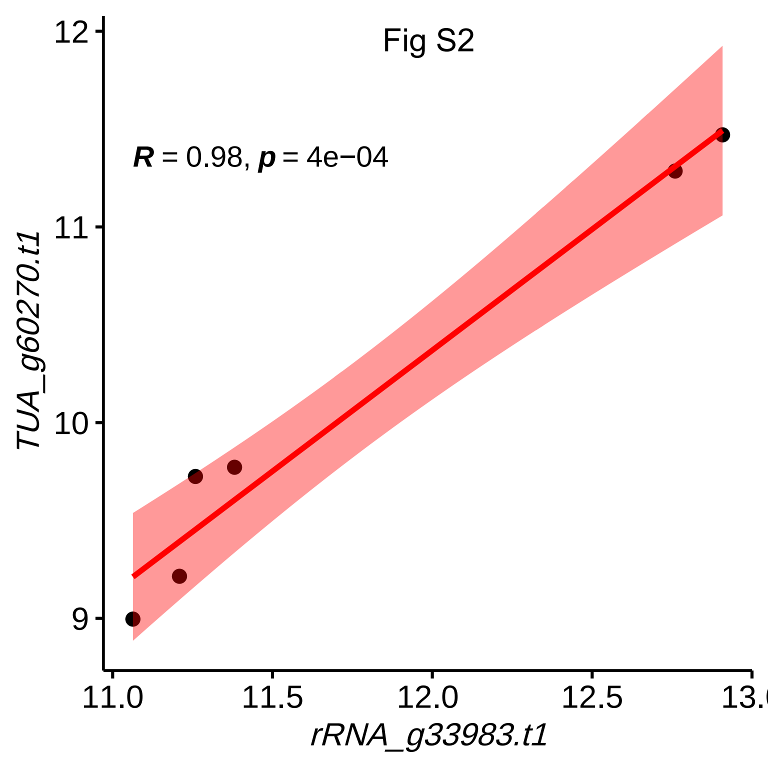

Fig S2
R = 0.98, p = 4e−04
TUA_g60270.t1
rRNA_g33983.t1
12
11
10
9
11.0
11.5
12.0
12.5
13.0

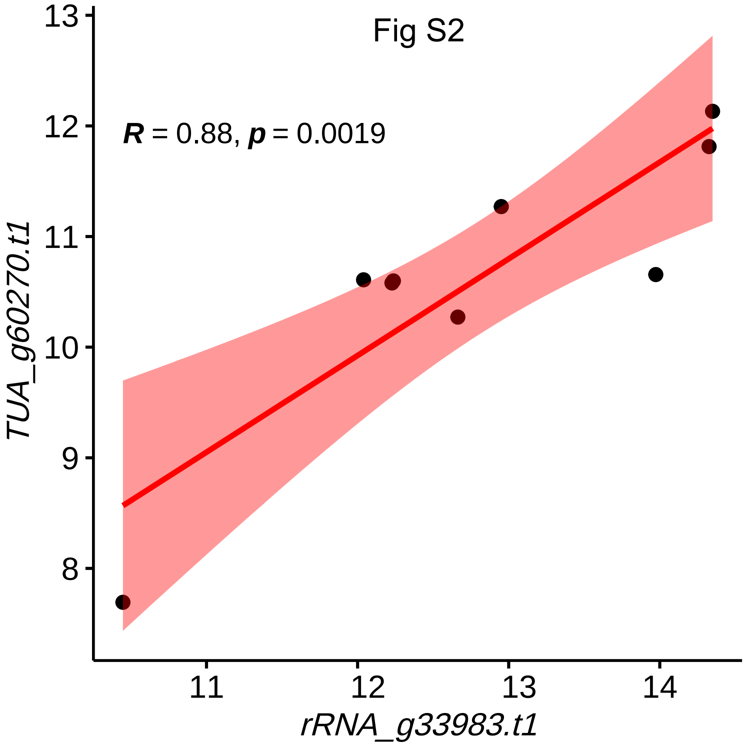

Fig S2
R = 0.88, p = 0.0019
TUA_g60270.t1
rRNA_g33983.t1
13
12
11
10
9
8
11
12
13
14

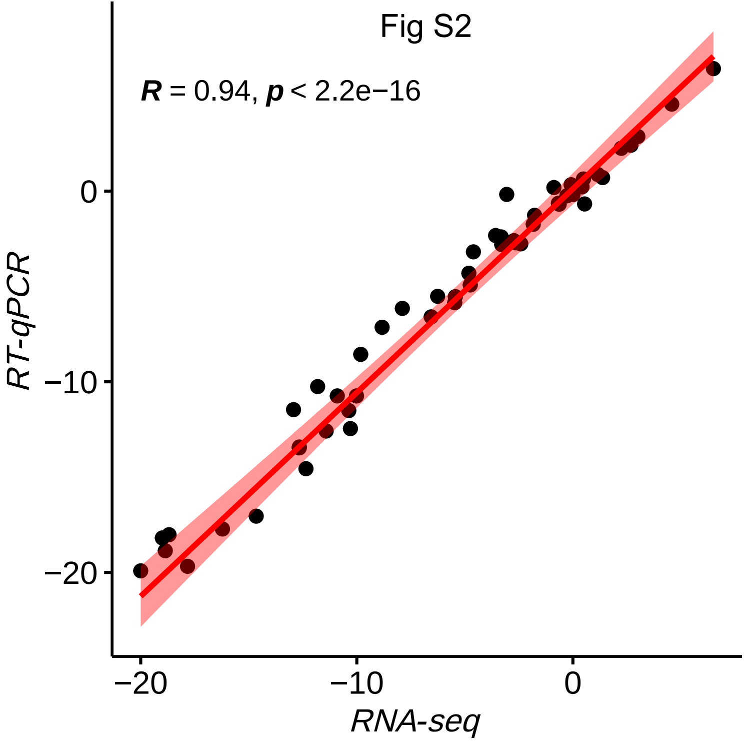
Fig S2
R = 0.94, p < 2.2e−16
RT-qPCR
RNA-seq
0
−10
−20
−20
−10
0

Fig S3

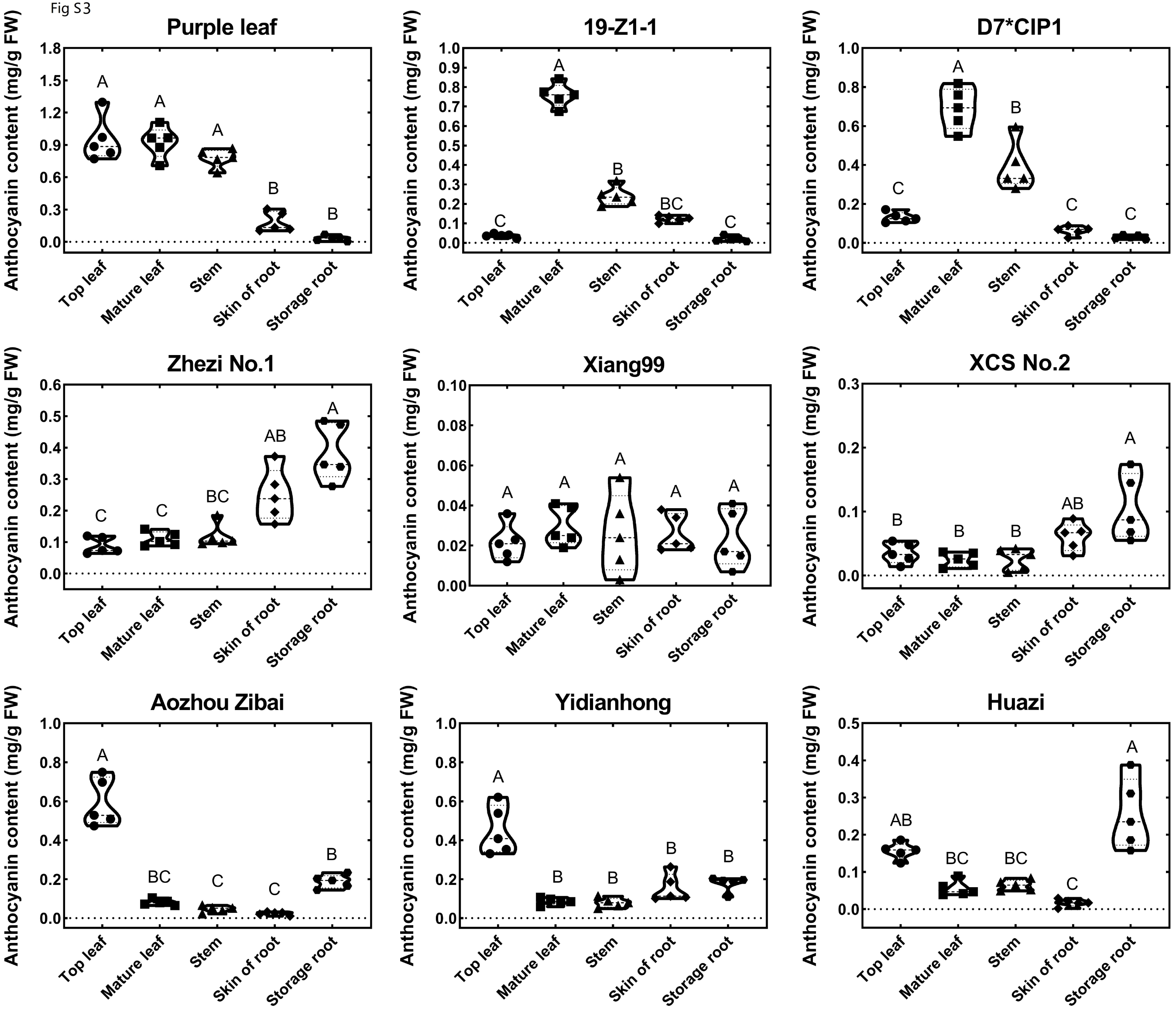
Purple leaf
19-Z1-1
D7*CIP1
Zhezi No.1
Xiang99
XCS No.2
Aozhou Zibai
Yidianhong
Huazi
Anthocyanin content (mg/g FW)
Top leaf
Mature leaf
Stem
Skin of root
Storage root

Fig S4

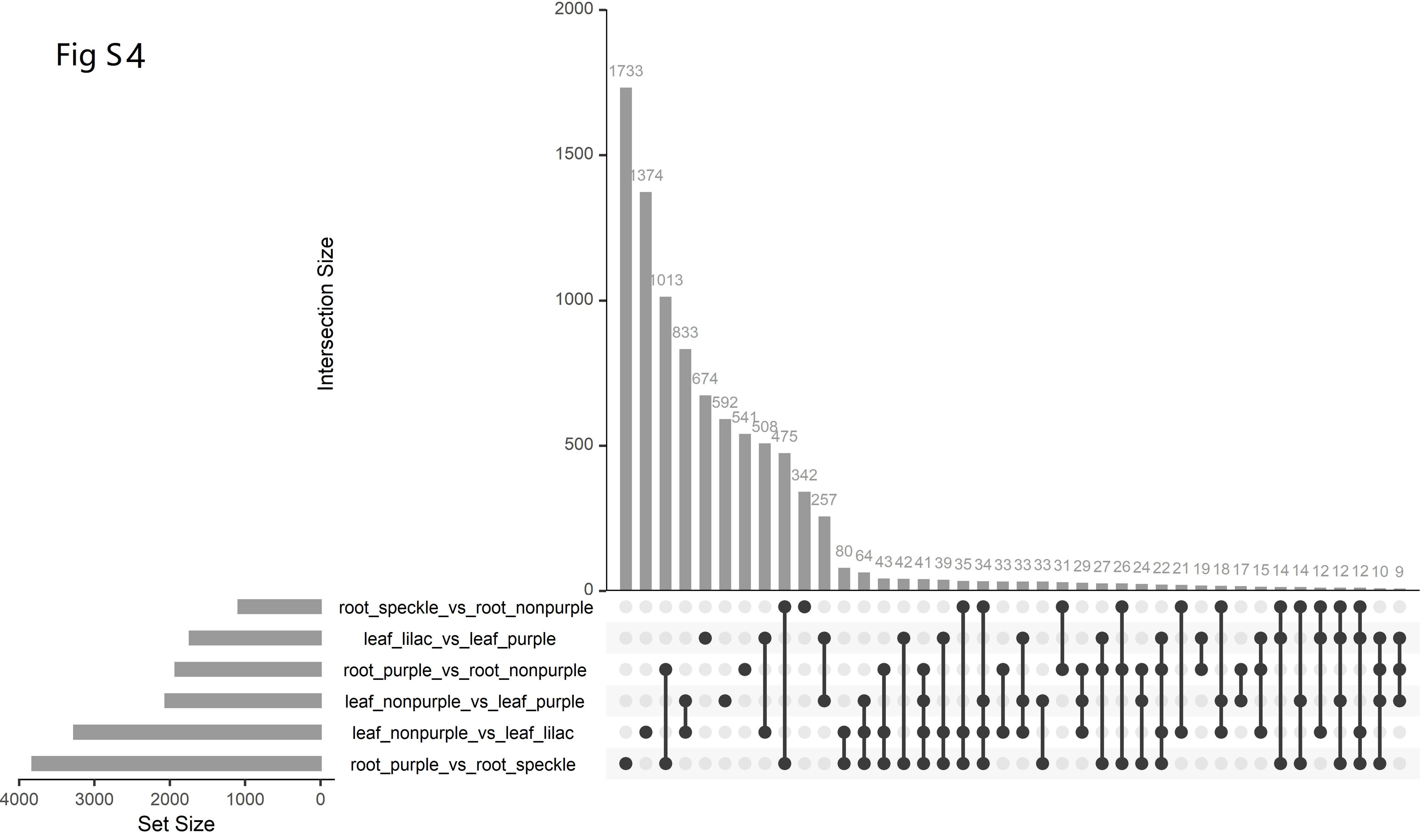

Intersection Size
2000
1500
1000
500
0
1733
1374
1013
833
674
592
541
508
475
342
257
80
64
43
42
41
39
35
34
33
33
33
31
29
27
26
24
22
21
19
18
17
15
14
14
12
12
12
10
9
root_speckle_vs_root_nonpurple
leaf_lilac_vs_leaf_purple
root_purple_vs_root_nonpurple
leaf_nonpurple_vs_leaf_purple
leaf_nonpurple_vs_leaf_lilac
root_purple_vs_root_speckle
4000
3000
2000
1000
0
Set Size

Fig S5

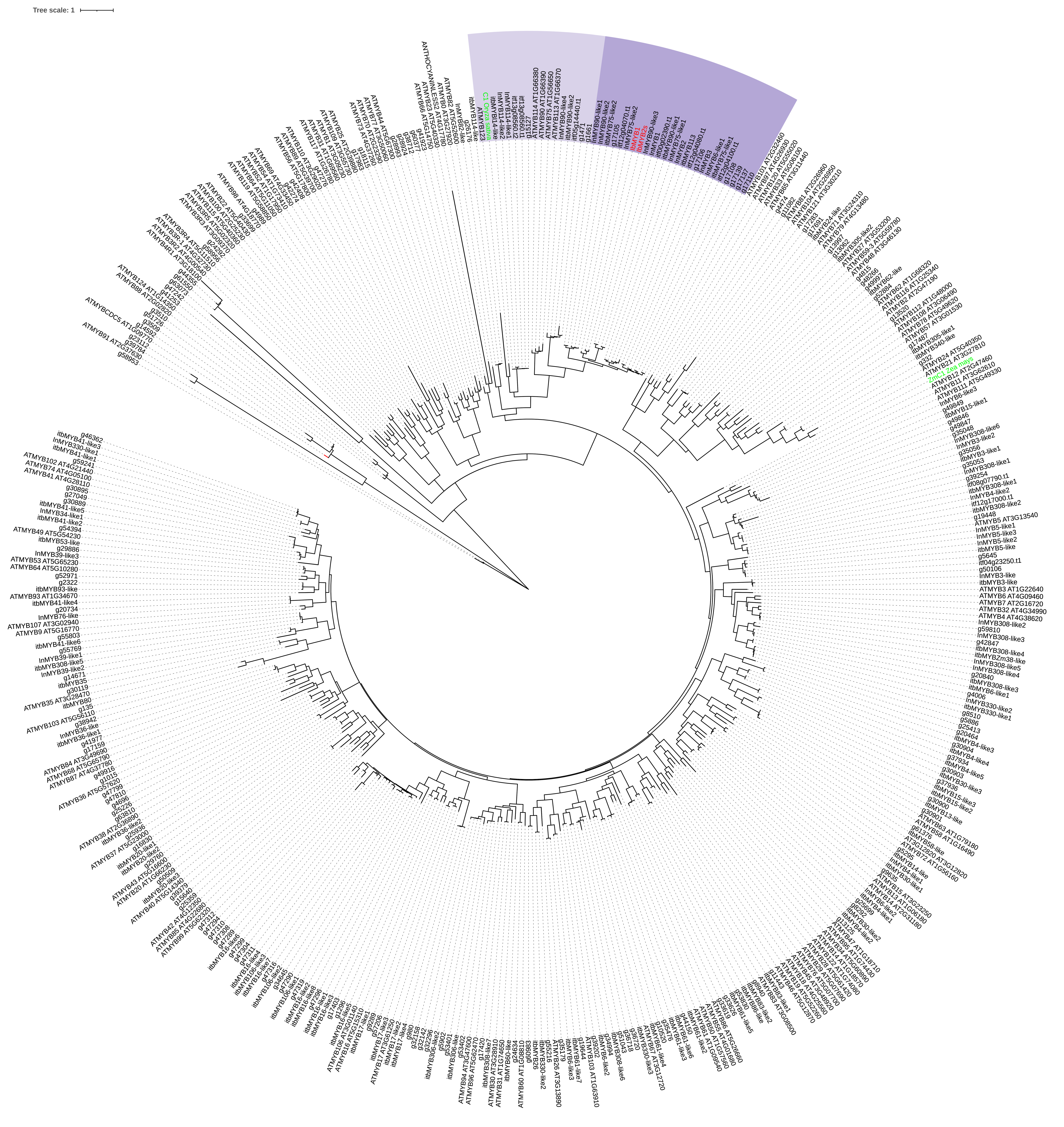

Fig S6

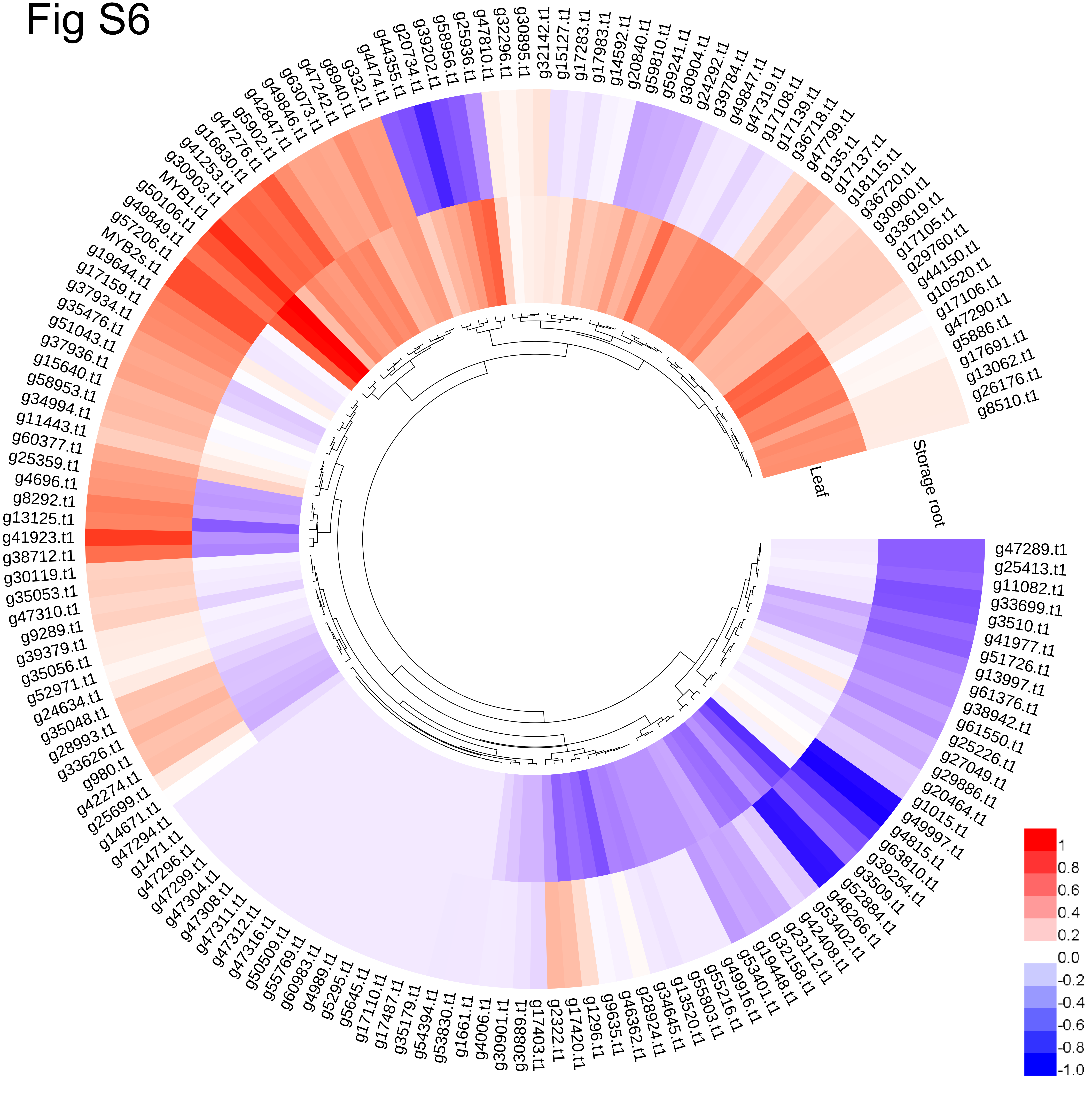
g4474.t1
g44355.t1
g20734.t1
g39202.t1
g58956.t1
g25936.t1
g47810.t1
g32296.t1
g30895.t1
g32142.t1
g15127.t1
g17283.t1
g17983.t1
g14592.t1
g20840.t1
g59810.t1
g59241.t1
g30904.t1
g24292.t1
g39784.t1
g49847.t1
g47319.t1
g17108.t1
g17139.t1
g36718.t1
g47799.t1
g135.t1
g17137.t1
g18115.t1
g36720.t1
g30900.t1
g33619.t1
g17105.t1
g29760.t1
g44150.t1
g10520.t1
g17106.t1
g47290.t1
g5886.t1
g17691.t1
g13062.t1
g26176.t1
g8510.t1
Leaf
Storage root
g47289.t1
g25413.t1
g11082.t1
g33699.t1
g3510.t1
g41977.t1
g51726.t1
g13997.t1
g61376.t1
g38942.t1
g61550.t1
g25226.t1
g27049.t1
g29886.t1
g20464.t1
g1015.t1
g49997.t1
g4815.t1
g63810.t1
g39254.t1
g33509.t1
g52884.t1
g48266.t1
g53402.t1
g42408.t1
g23112.t1
g32158.t1
g19448.t1
g53401.t1
g49916.t1
g55216.t1
g55803.t1
g13520.t1
g34645.t1
g28924.t1
g46362.t1
g9635.t1
g1296.t1
g17420.t1
g2322.t1
g17403.t1
g30889.t1
g30901.t1
g4006.t1
g1661.t1
g53830.t1
g54394.t1
g35179.t1
g17487.t1
g17110.t1
g5645.t1
g5295.t1
g4989.t1
g60983.t1
g55769.t1
g50509.t1
g47316.t1
g47312.t1
g47311.t1
g47308.t1
g47304.t1
g47299.t1
g47296.t1
g1471.t1
g47294.t1
g14671.t1
g25699.t1
g42274.t1
g980.t1
g33626.t1
g28993.t1
g35048.t1
g24634.t1
g52971.t1
g35056.t1
g39379.t1
g9289.t1
g47310.t1
g35053.t1
g30119.t1
g38712.t1
g41923.t1
g13125.t1
g8292.t1
g4696.t1
g25359.t1
g60377.t1
g11443.t1
g34994.t1
g58953.t1
g15640.t1
g37936.t1
g51043.t1
g35476.t1
g37934.t1
g17159.t1
g19644.t1
MYB2s.t1
g57206.t1
g49849.t1
g50106.t1
MYB1.t1
g30903.t1
g41253.t1
g16830.t1
g47276.t1
g5902.t1
g42847.t1
g49846.t1
g63073.t1
g47242.t1
g8940.t1
g332.t1
1
0.8
0.6
0.4
0.2
0.0
-0.2
-0.4
-0.6
-0.8
-1.0

Fig S7

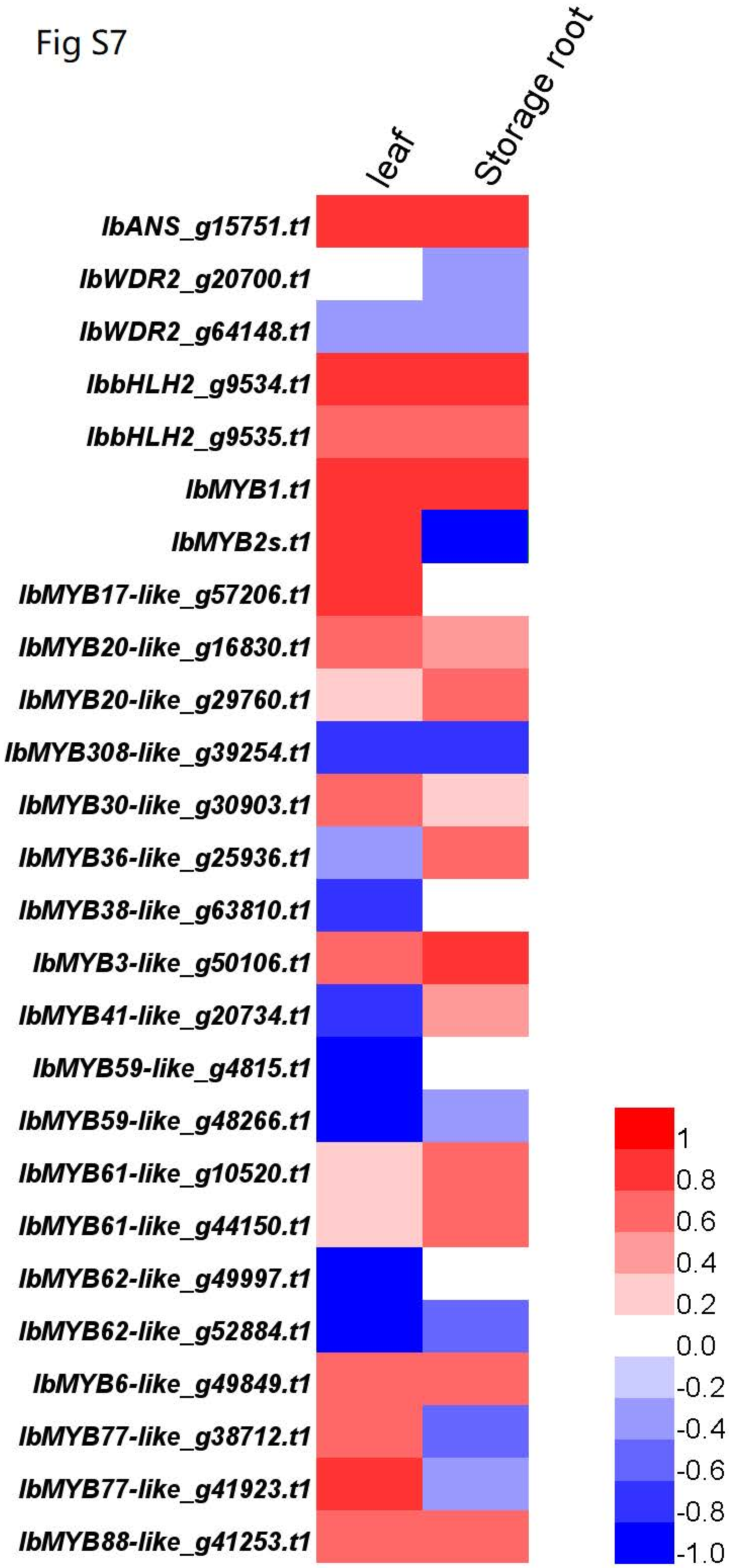

leaf
Storage root
IbANS_g15751.t1
IbWDR2_g20700.t1
IbWDR2_g64148.t1
IbbHLH2_g9534.t1
IbbHLH2_g9535.t1
IbMYB1.t1
IbMYB2s.t1
IbMYB17-like_g57206.t1
IbMYB20-like_g16830.t1
IbMYB20-like_g29760.t1
IbMYB308-like_g39254.t1
IbMYB30-like_g30903.t1
IbMYB36-like_g25936.t1
IbMYB38-like_g63810.t1
IbMYB3-like_g50106.t1
IbMYB41-like_g20734.t1
IbMYB59-like_g4815.t1
IbMYB59-like_g48266.t1
IbMYB61-like_g10520.t1
IbMYB61-like_g44150.t1
IbMYB62-like_g49997.t1
IbMYB62-like_g52884.t1
IbMYB6-like_g49849.t1
IbMYB77-like_g38712.t1
IbMYB77-like_g41923.t1
IbMYB88-like_g41253.t1
1
0.8
0.6
0.4
0.2
0.0
-0.2
-0.4
-0.6
-0.8
-1.0

Sequence: Supplementary Table5 3' flanking sequence of IbMYB1.dna (Linear / 3292 bp)
Features: 9 total
Primers: 5 total

Fig S8

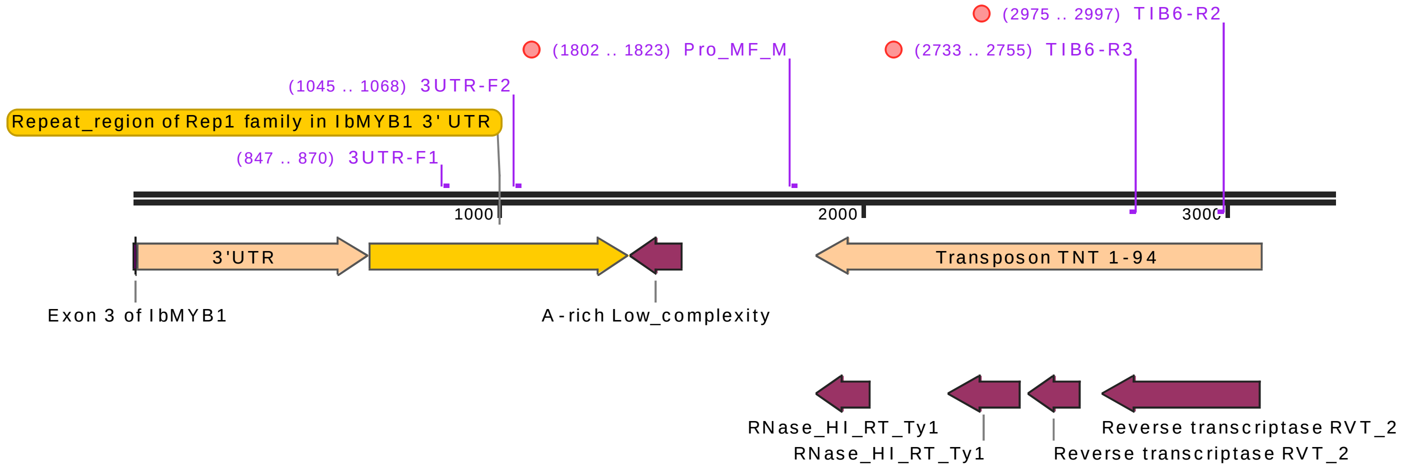

Supplementary Table5 3' flanking sequence of IbMYB1
3292 bp

8' DFWJWWDDJFWDDJFJWWDDDWWFDFWWWFDWFWWDFWDWWWWDDWWDFWWDWDJWJWJWWFWWJDWWWDDDWWF 75
6' WJDFDDWWFJDWWFJFDDWWWDDJWJDDDJWDJDDWJDWDDDDWWDDWJDDWDWFDFDFDDJDDFWDDDWWWDDJ

Exon 3 of IbMYB1 | 3'UTR

WDWWJFFDFFDDDDDDFFDDDDDDDDDJWWFDDJJDWJFDDWDWDDWFDDDWWDDDDWWJWDFJDWWDDDWWWJD 150
DWDDFJJWJJWWWWWWJJWWWWWWWWWFDDJWWFFWDFJWWDWDWWDJWWWDDWWWWDDFDWJFWDDWWWDDDFW

3'UTR

JDDWJWDDWFDWDDWFDDDWJWWDDDDDWDWWWWDJDWDWWWWDDWJDWFDWJWDDJJJWJWJFWWDWWDWWWDW 225
FWWDFDWWDJWDWWDJWWWDFDDWWWWWDWDDDDWFWDWDDDDWWDFWDJWDFDWWFFFDFDFJDDWDDWDDDWD

3'UTR

WWDDWDJFDDDWJDFDWDWJFDDWDDDWDWWWJWFDWWFDWWWFDWWDJWJWWJWDJWJFWWJDFWFWWJDWWDD 300
DDWWDWFJWWWDFWJWDWDFJWWDWWWDWDDDFDJWDDJWDDDJWDDWFDFDDFDWFDFJDDFWJDJDDFWDDWW

3'UTR

FJWJWDWFDWDWDWFFDJDWWFDFDWFDWWDDDDDWWFDJDDDDFDFJDFWJDDDDWWWDWDDDWWWFWJDFFDW 375
JFDFDWDJWDWDWDJJWFWDDJWJWDJWDDWWWWWDDJWFWWWWJWJFWJDFWWWWDDDWDWWWDDDJDFWJJWD

3'UTR

FWDDJDDWDDWWDDDDDDDDDWWDDWDWWWWWJJWFWDDDDFDDJJWWFFDDWFDWWWWWDJJDWWFDFDDDDDW 450
JDWWFWWDWWDDWWWWWWWWWDDWWDWDDDDDFFDJDWWWWJWWFFDDJJWWDJWDDDDDWFFWDDJWJWWWWWD

3'UTR

DWDFFFDWDDJFFWWJDWWDFWFDJDWFJDDWDJWJFDFDDDDWFDJJJDWWJDWJWWWDDDDDDDDFWWJWDWW 525
WDWJJJWDWWFJJDDFWDDWJDJWFWDJFWWDWFDFJWJWWWWDJWFFFWDDFWDFDDDWWWWWWWWJDDFDWDD

3'UTR

WWDWJFFFWDWDWFWWFDWDWWWWDDFJJWWJDJDWWWJWFFDWDFJJDFWDDWDDDDDFDDDWJDWFWWWFDDD 600
DDWDFJJJDWDWDJDDJWDWDDDDWWJFFDDFWFWDDDFDJJWDWJFFWJDWWDWWWWWJWWWDFWDJDDDJWWW

3'UTR

DFDDDWDDWWFFJJDWDWWDDDJWDWWWFWWJJWDFWWWWWWDWDWWJWJJWWJWDDDJDWWWWWWDDFDWDWFW 675
WJWWWDWWDDJJFFWDWDDWWWFDWDDDJDDFFDWJDDDDDDWDWDDFDFFDDFDWWWFWDDDDDDWWJWDWDJD

3'UTR | Repeat_region of Rep1 family in IbMYB1 3' UTR

WWFDDDDFJDJJJFWWWFJJDWWJDFWDDDDJJWDDWWFDWFDFFDDWWDWDDDWFWDFFDDJWWFWWFDDWFDW 750
DDJWWWWJFWFFFJDDDJFFWDDFWJDWWWWFFDWWDDJWDJWJJWWDDWDWWWDJDWJJWWFDDJDDJWWDJWD

Repeat_region of Rep1 family in IbMYB1 3' UTR

WDDFWFWFJWJDDWDDJWDDDDDWDFFWFDWFFDJJDJWDJFDWDFFFJJDDDWWWJFJDWWJWFFWWWFWFDDJ 825
DWWJDJDJFDFWWDWWFDWWWWWDWJJDJWDJJWFFWFDWFJWDWJJJFFWWWDDDFJFWDDFDJJDDDJDJWWF

Repeat_region of Rep1 family in IbMYB1 3' UTR

3UTR-F1
ACCGTGAACATTCATTAAGTGCCG

WWJWFFWWFDWFDFWFFWFDDDFFJWJDDFDWWFDWWDDJWJFFJDWWDDJDJWWFFDJWDFFDFWDFWWFFJJD 900
DDFDJJDDJWDJWJDJJDJWWWJJFDFWWJWDDJWDDWWFDFJJFWDDWWFWFDDJJWFDWJJWJDWJDDJJFFW

Repeat_region of Rep1 family in IbMYB1 3' UTR

DWWFFDDJDDDDWJJDJWWWJDDWFFWWWWFDWDWWWJFDFDWDWWWWDFDDWJWDFWDDDWDWFJDWWWJDJJD 975
WDDJJWWFWWWWDFFWFDDDFWWDJJDDDDJWDWDDDFJWJWDWDDDDWJWWDFDWJDWWWDWDJFWDDDFWFFW

Repeat_region of Rep1 family in IbMYB1 3' UTR

3UTR-F2
AGAAAT

WFFWWFDDFJJJWFWWWWWWFDDDJWJWWWFFDDDFFFDWJDWJWWWJWWWFFWFFFWFFFWFFDWWJDDJDDDW 1050
DJJDDJWWJFFFDJDDDDDDJWWWFDFDDDJJWWWJJJWDFWDFDDDFDDDJJDJJJDJJJDJJWDDFWWFWWWD

Repeat_region of Rep1 family in IbMYB1 3' UTR

3UTR-F2
AGGAGGAAGACTGACATC

DJJDJJDDJDFWJDFDWFDFFDDFWJDWWFWWJDJDDWFWWFDDWWJJFDWFWWJFFJJJDWWJFFDFWDWDDJF 1125
WFFWFFWWFWJDFWJWDJWJJWWJDFWDDJDDFWFWWDJDDJWWDDFFJWDJDDFJJFFFWDDFJJWJDWDWWFJ

Repeat_region of Rep1 family in IbMYB1 3' UTR

FDWDWJDFWDDDWDDDDWWDJJDJWDWDDJDDWWDDDDDWDWDJDDFWDDDWDJWDWWDWDDFWWDWDDJJDDFW 1200
JWDWDFWJDWWWDWWWWDDWFFWFDWDWWFWWDDWWWWWDWDWFWWJDWWWDWFDWDDWDWWJDDWDWWFFWWJD

Repeat_region of Rep1 family in IbMYB1 3' UTR

DDDDJDWDWDDDWWJDDWDDWJDDFWDDDWDWDWDWDDDWWJDDWDDDJWDDWDDDDJWWJWJDFWDDWDDDDDW 1275
WWWWFWDWDWWWDDFWWDWWDFWWJDWWWDWDWDWDWWWDDFWWDWWWFDWWDWWWWFDDFDFWJDWWDWWWWWD

Repeat_region of Rep1 family in IbMYB1 3' UTR

DDJDDWDWJWDWDDDJDDWWJWWJWJDJDDWJWJDJWDDDJFWDWDDJDDWDDWDJDJDWWWFDJDJWWJJWWWJ 1350
WWFWWDWDFDWDWWWFWWDDFDDFDFWFWWDFDFWFDWWWFJDWDWWFWWDWWDWFWFWDDDJWFWFDDFFDDDF

Repeat_region of Rep1 family in IbMYB1 3' UTR

DDDDWWWWJWWJDDDDDDDWJDDWWJDJWDDDDJJWDWWWDWDJDDJJDDDJDJDWJDJDDDDDWWJJDFJJDWJ 1425
WWWWDDDDFDDFWWWWWWWDFWWDDFWFDWWWWFFDWDDDWDWFWWFFWWWFWFWDFWFWWWWWDDFFWJFFWDF

Repeat_regio... A-rich Low_complexity

JFFDDWJFDDFJJFDDDDDDWDWDDFFJWWJFDFWDJDJWJJFFDWFFDJFFDDDDDDDJDWWDDWDDDDWDDDW 1500
FJJWWDFJWWJFFJWWWWWWDWDWWJJFDDFJWJDWFWFDFFJJWDJJWFJJWWWWWWWFWDDWWDWWWWDWWWD

A-rich Low_complexity

DDDWDDDJWWJFDDFJWJFDDFJJFFJDDDWWWJJFFJWWJFDFDWWJFDDFDWJWFDDFDWJJDJFFWWJJJFD 1575
WWWDWWWFDDFJWWJFDFJWWJFFJJFWWWDDDFFJJFDDFJWJWDDFJWWJWDFDJWWJWDFFWFJJDDFFFJW

A-rich Low_complexity

DDJFWJFJFDFDJFWWJFFFDDJDFDDDDWDDWDWWWWWWWDDWWDDDWDWDWDJWDJWJJFDWJWJJFFWDJWJ 1650
WWFJDFJFJWJWFJDDFJJJWWFWJWWWWDWWDWDDDDDDDWWDDWWWDWDWDWFDWFDFFJWDFDFFJJDWFDF

```
JJWJJFDJFDDDJFDWJJFDFDWWWJFFDDWWJFFDWJFWWWJWWJFWWWDFWJFJWJJDJWJFJWJFJWJFWJF  1725
FFDFFJWFJWWWFJWDFFJWJWDDDFJJWWDDFJJWDFJDDDFDDFJDDDWJDFJFDFFWFDFJFDFJFDFJDFJ

FFFFJDJJFFFFWDFFWWJFFFJJJFFWJJJFFFDJJWFWJJWFFWJJJFWJJJDFFFJJJFWWWWWWWWFDDDJ  1800
JJJJFWFFJJJJDWJJDDFJJJFFFJJDFFFJJJWFFDJDFFDJJDFFFJDFFFWJJJFFFJDDDDDDDDJWWWF

Pro_MF_M
ACCTGAACCGGCCCTTATTATG
DDFFWJDDFFJJFFFWWDWWDWJFJJWWFWJDWFFJJJFWDJJFFFDFWWWWDDJFDJJWWFDWJFFWJJJFFJJ  1875
WWJJDFWWJJFFJJJDDWDDWDFJFFDDJDFWDJJFFFJDWFFJJJWJDDDDWWFJWFFDDJWDFJJDFFFJJFF

WWDWWWWWFJJDFFJDWWFDJJJWWJJWWFDDFFWWJDDFFJDFFFJWJJFFDFFWFWDFFDWJDDJWJJDJWDW  1950
DDWDDDDDJFFWJJFWDDJWFFFDDFFDDJWWJJDDFWWJJFWJJJFDFFJJWJJDJDWJJWDFWWFDFFWFDWD
Transposon TNT 1-94
50 45 40 35 30
* Lys Glu Ser Arg Asn Leu Thr Pro Glu Val Lys Phe Arg Gly Thr Ala Val Glu Val Met Phe His Leu Ile
RNase_HI_RT_Ty1

JDWFDFWWJDDDDJJDDDWFFDDJWWFDJDDDJDDJDFFDFJWDDFFDFDFDDWFWFWJDDFDFJFDJFDJDFDW  2025
FWDJWJDDFWWWWFFWWWDJJWWFDDJWFWWWFWWFWJJWJFDWWJJWJWJWWDJDJDFWWJWJFJWFJWFWJWD
Transposon TNT 1-94
25 20 15 10 5 1
Ile Val Gln Phe Pro Phe Gly Leu Glu Ser Leu Leu Gly Arg Leu Trp Val Ile Glu Ser Cys Ala Ala Ser Met
RNase_HI_RT_Ty1

DJFWFWDWDFWFJJFFWFWJWDJDJJDWWWDJDJDFDFWFWFWWJFWWFWWDFDFWWFFDDJDDDWFDDJJFDWF  2100
WFJDJDWDWJDJFFJJDJDFDWFWFFWDDDWFWFWJWJDJDJDDFJDDJDDWJWJDDJJWWFWWWDJWWFFJWDJ
Transposon TNT 1-94

WFFDDJDDDDDDFJFDFFDWFFWJWJDWDJDWWWFFWDJWDWFWJJDFDJFFDJFFFDDWFWJFDWFDFWDWDWJF  2175
DJJWWFWWWWWJFJWJJWDJJDFDFWDWFWDDDJJDWFDWDJDFFWJWFJJWFJJJWWDJDFJWDJWJDWDWDFJ
Transposon TNT 1-94

WJWFDDDWJDDJDJFWJDWWJWWWJJJJDDDDDFDDFFFDFJDJWDJJWJDFFFDDWWDJDWDWFDDDFWDWJFJ  2250
DFDJWWWDFWWFWFJDFWDDFDDDFFFFWWWWWJWWJJJWJFWFDWFFDFWJJJWWDDWFWDWDJWWWJDWDFJF
Transposon TNT 1-94
65
* Val Ile Arg
RNase_HI_RT_Ty1

DFJDDWWJFDJFDDJDWJJDJDWJWFJDJJWJDJWJFDWJDDWWWDFWDDFJDWDWJDDFDJFDWDJJDJDWDWF  2325
WJFWWDDFJWFJWWFWDFFWFWDFDJFWFFDFWFDFJWDFWWDDDWJDWWJFWDWDFWWJWFJWDWFFWFWDWDJ
Transposon TNT 1-94
60 55 50 45 40
Arg Ile Ala Ala Leu His Leu His Arg Pro Ser His Met Phe Lys Ser Val Ile His Val Ala Tyr Ser Ile Asp
RNase_HI_RT_Ty1

JJJFFWWJWDDWWJWWDJJWDJDWDDDDFWDFFDDFDDJWWJFFWDWDFDDDDDDJJDWFWJJFDDWJJWWFJFF  2400
FFFJJDDFDWWDDFDDWFFDWFWDWWWWJDWJJWWJWWFDDFJJDWDWJWWWWWWFFWDJDFFJWWDFFDDJFJJ
Transposon TNT 1-94
35 30 25 20 15
Pro Arg Thr Ile Thr Leu Tyr Ile Phe Ser Gly Val Leu Gln Arg Tyr Leu Phe Pro Asp Pro Leu Pro Glu Gly
RNase_HI_RT_Ty1
```

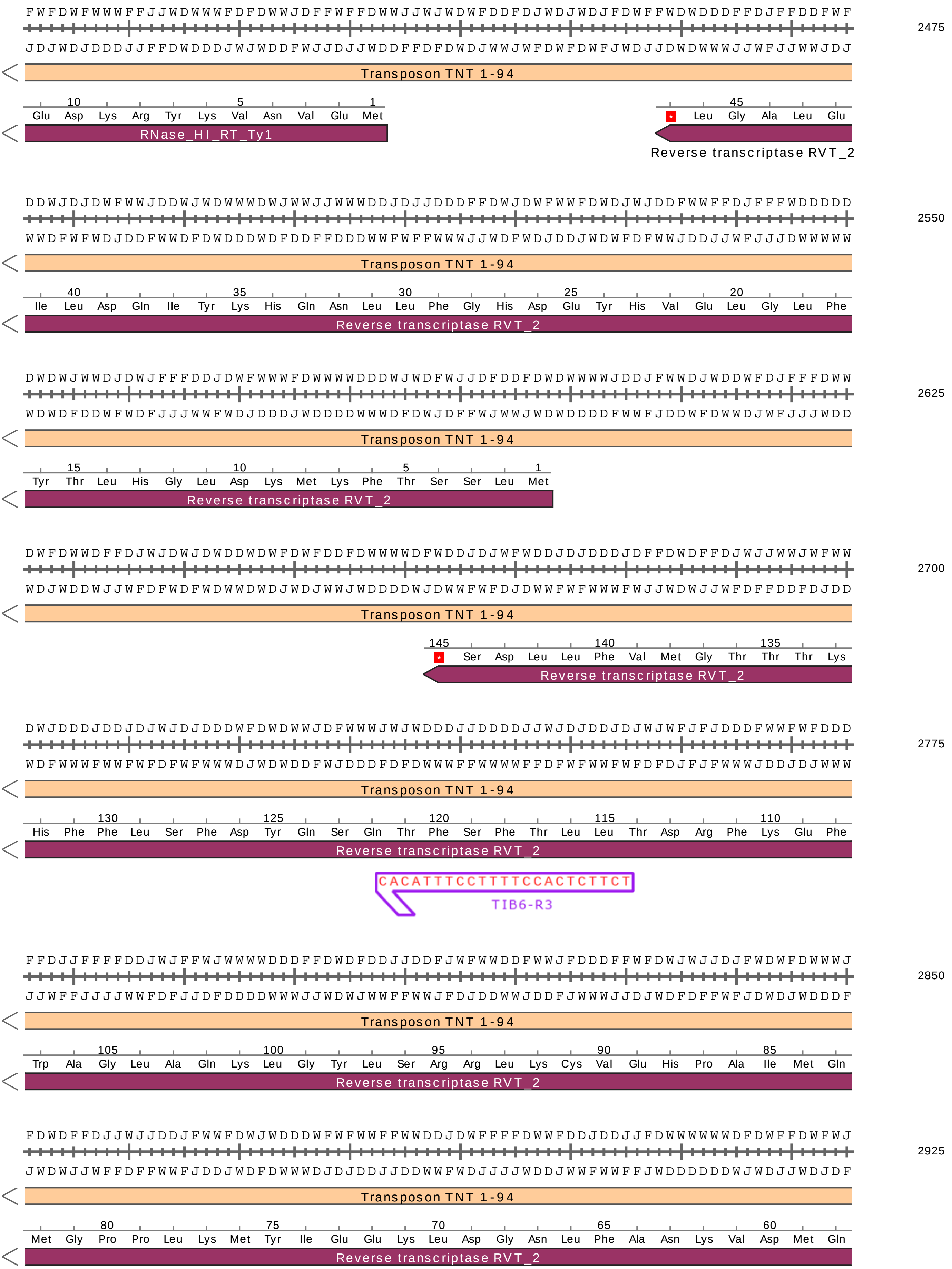
2475
Transposon TNT 1-94
RNase_HI_RT_Ty1
Reverse transcriptase RVT_2
2550
Transposon TNT 1-94
Reverse transcriptase RVT_2
2625
Transposon TNT 1-94
Reverse transcriptase RVT_2
2700
Transposon TNT 1-94
Reverse transcriptase RVT_2
2775
Transposon TNT 1-94
Reverse transcriptase RVT_2
CACATTTCCTTTTCCACTCTTCT
TIB6-R3
2850
Transposon TNT 1-94
Reverse transcriptase RVT_2
2925
Transposon TNT 1-94
Reverse transcriptase RVT_2

D W J W D D D D F F F D D J D W W J D J D J J D D J F J D W D D F D D J W D W J J W W F W F D W W J W J J W F D W F W W D J F D D F W J J W J W D D D 3000
W D F D W W W W J J J W W F W D D F W F W F F W W F J F W D W W J W W F D W D F F D D J D J W D D F D F F D J W D J D D W F J W W J D F F D F D W W W

Transposon TNT 1-94

55 50 45 40 35
His Leu Val Trp Ser Gln Ser Ser Ala Ile Val Leu Ile Thr Arg Met Thr Thr Met Lys Ala Val Pro Thr Phe

Reverse transcriptase RVT_2

CACCAGTAGAATCGTTGACCACA
TIB6-R2

W J W W W F W W F J W D J W F J D J D F F D W D W W F W W J W W W D W W F F F D D J D J F D D F F D D W F J D J F W W W D W D W F W D W F F D D W J D 3075
D F D D D J D D J F D W F D J F W F W J J W D W D D J D D F D D D W D D J J J W W F W F J W W J J W W D J F W F J D D D W D W D J D W D J J W W D F W

Transposon TNT 1-94

30 25 20 15 10
Thr Glu Glu Tyr Asp Leu Gly Tyr Glu Gln Lys Asn Gly Leu Ala Val Leu Arg Ala Lys Tyr Arg Asp Leu Ser

Reverse transcriptase RVT_2

J F W D W F D J D F W W J D J W W W F D W D J W D W D D D F F F D F W W J F W D F F D D W J J J D W W D D F D W W W W J J J J D F D F J D D D F D D W 3150
F J D W D J W F W J D D F W F D D D J W D W F D W D W W W J J J W J D D F J D W J J W W D F F F W D D W W J W D D D D F F F F W J W J F W W W J W W D

Transposon TNT 1-94

5 1
Ser Asp Ser Lys Leu Lys Met

Reverse transcriptase RVT_2

J W F F F D D W D W J D W W W W F W W F D D J W J F W W J D D J W W D W W F W W F F D W W J F W W J W W J F F D D D F D F W W D W J W W F F D W W J F F 3225
F D J J J W W D W D F W D D D D J D D J W W F D F J D D F W W F D D W D D J D D J J W D D F J D D F D D F J J W W J W J D D W D F D D J J W D D F J J

W W W W W D W D D F D D J W J J J J D W D J D J D W D J D D J D W D D F D F D J W D J D F D W D J D F D F D J J J W W J D D D D D D F F 6′
3292
D D D D D W D W W J W W F D F F F F W D W F W F W D W F W W F W D W W J W J W F D W F W J W D W F W J W J W F F F D D F W W W W W J J 8′

	Feature	Location	Size	Color	Direction	Type
✓	**Exon 3 of IbMYB1**	1 .. 13	13 bp	purple	→	exon
✓	**3'UTR**	14 .. 649	636 bp	light orange	→	3'UTR
✓	**Repeat_region of Rep1 family in IbMYB1 3'...**	650 .. 1362	713 bp	yellow	→	misc_feature
✓	**A-rich Low_complexity**	1363 .. 1507	145 bp	maroon	←	misc_feature
✓	**Transposon TNT 1-94**	1876 .. 3099	1224 bp	light orange	←	precursor_RNA
	/note	= Amino acid homologous sequence from *Apostasia shenzhenica*				
✓	**RNase_HI_RT_Ty1**	1876 .. 2025	150 bp	maroon	←	CDS
	/translation	= MSAACSEIVWLRGLLSELGFPFQVIILHFMVEVATGRFKVEPTLNRSEK* 49 amino acids = 5.6 kDa				
✓	**RNase_HI_RT_Ty1**	2239 .. 2433	195 bp	maroon	←	CDS
	/translation	= MEVNVKYRKDEGEPLPDPFLYRQLVGSFIYLTITRPDISYAVHIVSKFMHSPRHLHLAAIRRIV* 64 amino acids = 7.5 kDa				
✓	**Reverse transcriptase RVT_2**	2458 .. 2598	141 bp	maroon	←	CDS
	/translation	= MLSSTFKMKDLGHLTYFLGLEVHYEDHGFLLNQHKYIQDLIELAGL* 46 amino acids = 5.4 kDa				
✓	**Reverse transcriptase RVT_2**	2662 .. 3096	435 bp	maroon	←	CDS
	/translation	= MKLKSDSSLDRYKARLVALGNKQEYGLDYEETFTPVAKMTTMRTILVIASSQSWVLHQMDVKNAFLNGDLKEEIYMKLPPGMQMIA PHEVCKLRRSLYGLKQALGAWFEKFRDTLLTFSFTQSQYDFSLFFHKTTTGMVFLLDS* 144 amino acids = 16.7 kDa				

	Primer	Length		Binding Sites	⇄	Tm	Date Added
✓	**3UTR-F1**	24-mer		847 .. 870	→	60°C	
	/sequence = ACCGTGAACATTCATTAAGTGCCG<br>46% GC / 7336.8 Da						
✓	**3UTR-F2**	24-mer		1045 .. 1068	→	56°C	
	/sequence = AGAAATAGGAGGAAGACTGACATC<br>42% GC / 7468.0 Da						
✓	**Pro_MF_M**	22-mer	●	1802 .. 1823	→	59°C	
	/sequence = ACCTGAACCGGCCCTTATTATG<br>50% GC / 6670.4 Da						
✓	**TIB6-R3**	23-mer	●	2733 .. 2755	←	55°C	
	/sequence = TCTTCTCACCTTTTCCTTTACAC<br>39% GC / 6826.5 Da						
✓	**TIB6-R2**	23-mer	●	2975 .. 2997	←	59°C	
	/sequence = ACACCAGTTGCTAAGATGACCAC<br>48% GC / 7001.6 Da						

DNA Type: Natural DNA

Description:

Created: 2021年3月2日
Last Modified: 2021年3月2日

Accession Number:
Code Number:

Sequence Author: Trial User

Comments:

References:
Embedded Files:

Fig S9

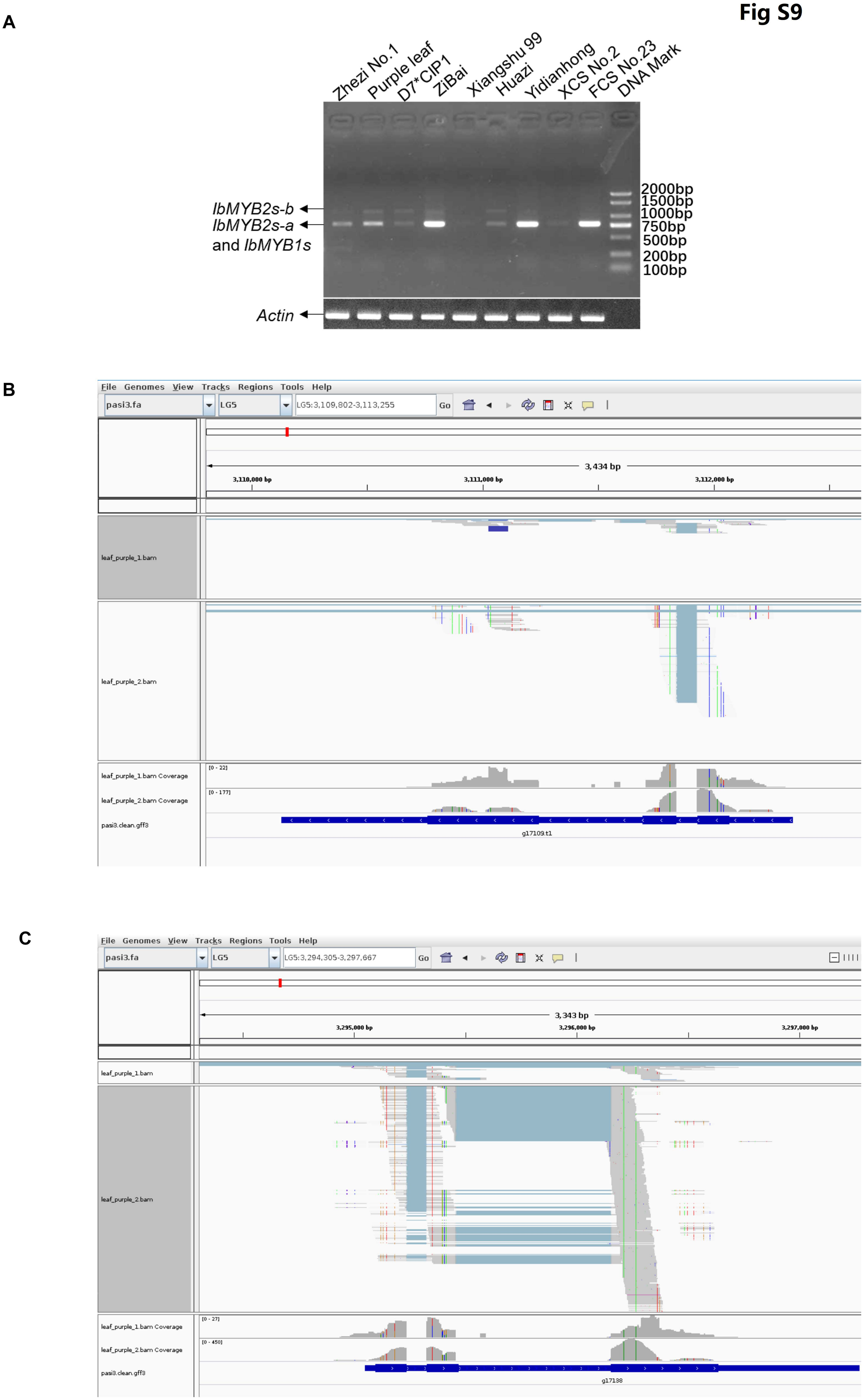
A
Zhezi No.1
Purple leaf
D7*CIP1
ZiBai
Xiangshu 99
Huazi
Yidianhong
XCS No.2
FCS No.23
DNA Mark
IbMYB2s-b
IbMYB2s-a
and IbMYB1s
2000bp
1500bp
1000bp
750bp
500bp
200bp
100bp
Actin
B
pasi3.fa
LG5
LG5:3,109,802-3,113,255
3,434 bp
3,110,000 bp
3,111,000 bp
3,112,000 bp
leaf_purple_1.bam
leaf_purple_2.bam
leaf_purple_1.bam Coverage
leaf_purple_2.bam Coverage
pasi3.clean.gff3
[0 - 22]
[0 - 177]
g17109.t1
C
pasi3.fa
LG5
LG5:3,294,305-3,297,667
3,343 bp
3,295,000 bp
3,296,000 bp
3,297,000 bp
leaf_purple_1.bam
leaf_purple_2.bam
leaf_purple_1.bam Coverage
leaf_purple_2.bam Coverage
pasi3.clean.gff3
[0 - 27]
[0 - 450]
g17138

Fig S9

D

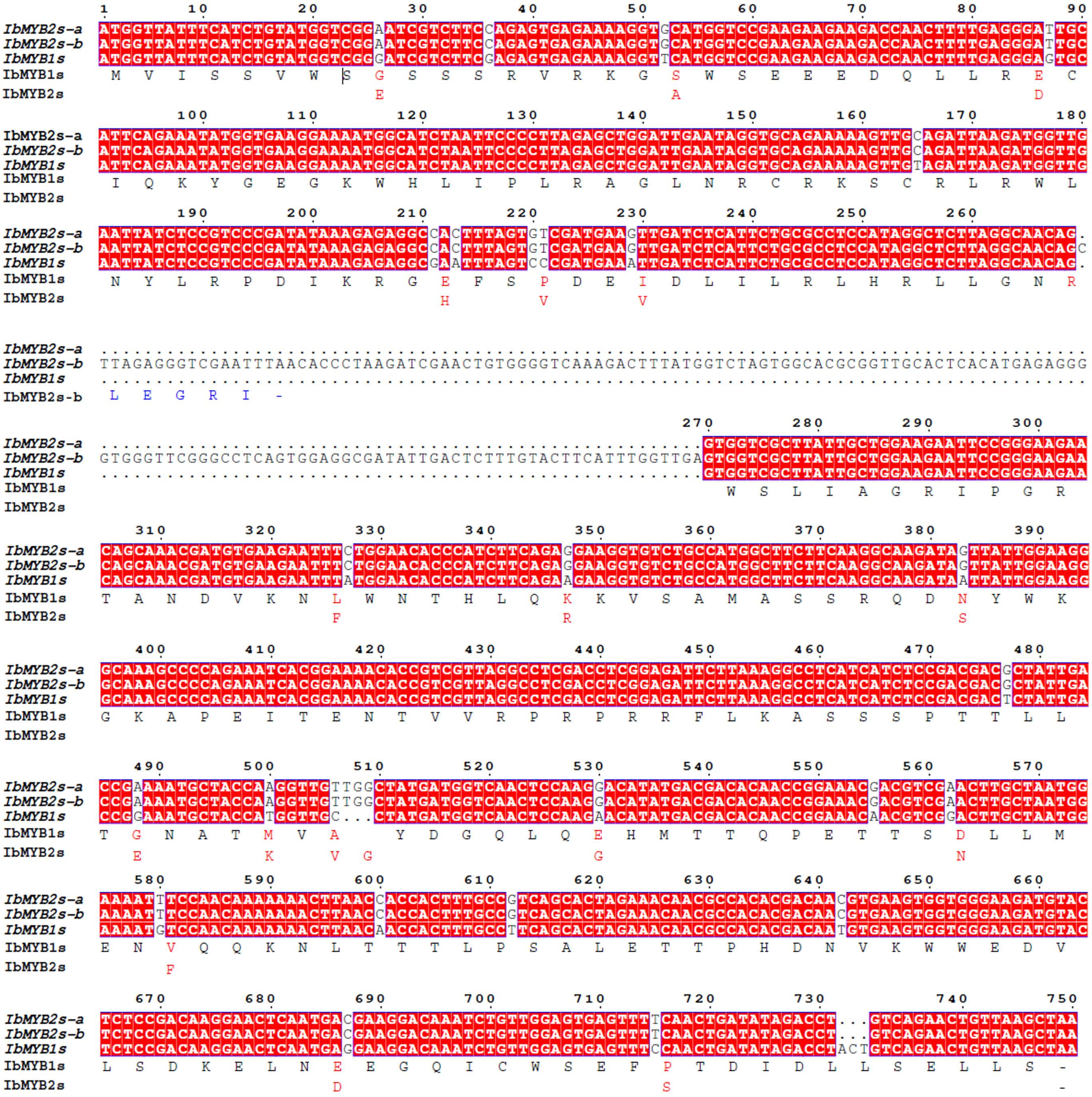

Fig S10. InDels of 5' flanking sequence of *IbMYB2s* gene. *IbMYB2s* is the 5' flanking sequence that cloned from genome in this study, *IbMYB2-1* and *IbMYB2-1* are the 5' flanking sequences that obtained from reference genome.

```
IbMYB2-4  ............................................................
IbMYB2-1  ............................................................
IbMYB2s   AGTGAAGAAACAGAGATGATTTGATTTAGGAACTTCAGATGTATGTTGTGGCAATATGAT

IbMYB2-4  ............................................................
IbMYB2-1  ............................................................
IbMYB2s   CATGATCACCTCTAGGAAGTCATGTAACCACCTTGTGAGTTTTGAATCTTTTATGAGTGA

IbMYB2-4  ............................................................
IbMYB2-1  ............................................................
IbMYB2s   TTTGGAGTTCTAAGGTAGAATGTAGAGCTCTTCACAAATGTACTGCAAAGTGTGCTTTCA

IbMYB2-4  ............................................................
IbMYB2-1  ...........GTGAAGACACACCAAGTTCAACATATTCATACATTAACCTTGTATAATA
IbMYB2s   GAAGTTTGATCGTGAAGACACACCAAGTTCAACATATTCATACATTAACCTTGTATAATA

IbMYB2-4  ............................................................
IbMYB2-1  ACTTTATAAAAAAGTTTATGAGATGCAATTTTGTACTAATTTGACAATTGTACAAAATGC
IbMYB2s   ACTTTATAAAAAAGTTTATGAGATGCAATTTTGTACTAATTTGACAATTGTACAAAATGC

                              1        10        20        30        40
IbMYB2-4  ....................CGATTGTACTGAAAAATCTCTAGAGCTGAGTTTTTCTGGC
IbMYB2-1  AATTACCTGATCAATTCACACGATTGTACTGAAAAATCTCTAGAGCTGAGTTTTTCTGGC
IbMYB2s   AATTACCTGATCAATTCACACGATTGTACTGAAAAATCTCTAGAGCTGAGTTTTTCTGGC

                  50        60        70        80        90       100
IbMYB2-4  AGTAAGCAGTGTAGCACCTTCAATATGGAGTCTGAATATTTTCTTGAAACCTGAGATAAT
IbMYB2-1  AGTAAGCAGTGTAGCACCTTCAATATGGAGTCTGAATATTTTCTTGAAACCTGAGATAAT
IbMYB2s   AGTAAGCAGTGTAGCACCTTCAATATGGAGTCTGAATATTTTCTTGAAACCTGAGATAAT

                 110       120       130        140       150
IbMYB2-4  CCCTTAAACTCCACAATTTTAAGCCTTTATAA.CATCCTTTTATAGTGTAGATTTTAATT
IbMYB2-1  CCCTTAAACTCCACAATTTTAAGCCTTTATAA.CATCCTTTTATAGTGTAGATTTTAATT
IbMYB2s   CCCTTAAACTCCACAATTTTAAGCCTTTATAAACATCCTTTTATAGTGTAGATTTTAATT

        160       170       180       190       200       210
IbMYB2-4  TCCTCCATTTTTGCAGCATGTACCCAACTACCCACGTATGGTTAGTCCACCATGTTCCCA
IbMYB2-1  TCCTCCATTTTTGCAGCATGTACCCAACTACCCACGTATGGTTAGTCCACCATGTTCCCA
IbMYB2s   TCCTCCATTTTTGCAGCATGTGCCCAACTACCCACGTATGGTTAGTCCACCATGTTCCCA

        220       230       240       250       260       270
IbMYB2-4  ATTTTCATGCCCATAAATCCATAATCACATAATCATTTTTCATTTTTCCTAGTTATGCTT
IbMYB2-1  CTTTTCATGCCCATAAATCCATAATCACATAATCATTTTTCATTTTTCCTAGTTATGCTT
IbMYB2s   CTTTTCATGCCCATAAATCCATAATCACATAATCATTTTTCATTTTTCCTAGTTATGCTT

        280       290       300       310       320       330
IbMYB2-4  AAGGTGCGTGGGTGTTGAAATTCTTAAGCTGCGTTTTTTTCTTTCTTAAGGT........
IbMYB2-1  AAGGTGCGTGGGTGTTGAAATTCTTAAGCTGCGTTGTTTTCTTTCTTAAGGTCCGTGATA
IbMYB2s   AAGGTGCGTGGGTGTTGAAATTCTTAAGCTGCGTTGTTTTCTTTCTTAAGGTGCGTGATA
```

```
                                        340       350           360       370
IbMYB2-4  ..............GCGTGATATGTATGCTTAA.GGTGTGTAAGTTGTTGAAACTTAAGG
IbMYB2-1  TGTATGCTTAAGATGCGTGATATGTATGCTTAA.GGTGTGTAAGTTGTTGAAACTTAAGG
IbMYB2s   TGTATGCTTAAGATGCGTGATATGTATGCTTAAAGGTGTGTAAGTTGTTGAAACTTAAGG

           380       390       400       410       420       430
IbMYB2-4  TACGTCATTTTAAAACCTTAGGTGCGTGGATTGTATTATTAAAGTGCATGGCTTGTATAC
IbMYB2-1  TACGTCATTTTAAAACCTTAGGTGCGTGGATTGTATTATTAAAGTGCATGGCTTGTATAC
IbMYB2s   TACGTCATTTTAAAACCTTAGGTGCGTGGATTGTATTATTAAAGTGCATGGCTTGTATAC

           440       450       460                           470
IbMYB2-4  TTAAGGTGCGCAGTGTATTCTCAACT.....................TTAGGTGCGTGGT
IbMYB2-1  TTAAGGTGCGCAGTGTATTCTCAACTAAAGGTGCACCATTTAAAACTTTAGGTGTGTGGT
IbMYB2s   TTAAGGTGCGCAGTGTATTCTCAACTGAAGGTGCACCATTTAAAACTTTAGGTGCGTGGT

            480       490       500       510       520       530
IbMYB2-4  TTGTGTTCTTAGCTTAAGATGCACCGTTTTAATGCTTAAGGTGAATATCACACAACCG..
IbMYB2-1  TTGTGTTCTTAGCTTAAGATGCGCCGTTTTAATGCTTAAGGTGAATATCACACAACCGCA
IbMYB2s   TTGTGTTCTTAGCTTAAGATGCGCCGTTTTAATGCTTAAGGTGAATATCACACAACCGCA

                540       550       560       570       580       590
IbMYB2-4  ...GAAGATATATCTGCACCCGAACCCTATATATATATATATATATATATATATATATAT
IbMYB2-1  CGGGAAGATATATCCGAACCCGAACCCT...............TATATATATATATATAT
IbMYB2s   CGGGAAGATATATCCGCACCCGAACCCT................................

                600       610       620       630       640       650
IbMYB2-4  ATATATATATATATATATATATATATATATATATATATATATATAATTGAGGCCCTGCTC
IbMYB2-1  ATATATATATATATATATATATATATATATATATATATATATATAATTGAGGCCCTGCTC
IbMYB2s   ...........................TATATATATATATATATAATTGAGGCCCTGCTC

                660       670       680           690       700
IbMYB2-4  TCTTTACACAAGCTGGGTGGGTGGTGGGGGAG...GGGAGGCGGCGCGAGGGGTCATGTC
IbMYB2-1  TCTTTACACAAGCTGGGTGGGTGGTGGGGGGGAGAGGGAGGCGGCGCGAGGGGTCATGTC
IbMYB2s   TCTTTACACAAGCTGGGTGGGTGGTGGGGGGATG.GGGAGGCGGCGCGAGGGGTCATGTC

          710       720       730       740           750       760
IbMYB2-4  CCGAAATCATCACTGGTATATATTATAGCTCTGGTAA....CTATATTGCAGGGCATGCT
IbMYB2-1  CTGAAATCATCACTGGTATATATTATAGCTCTGGTAA....CTATATTGCAGGGCATGCT
IbMYB2s   CCGAAATCATCGCTGGTATATATTATAGCTCTGGTAATTAACTATATTGCAGGGCATGCT

              770       780       790       800       810       820
IbMYB2-4  TATACCAATAATTGGATGCTGCGCTATCTTCTATTATATTACTCAAGGTTTCTCCATCTT
IbMYB2-1  TATACCAATAATTGGATGCTGCGCTATCTTCTATTATATTACTCAAGGTTTCTCCATCTT
IbMYB2s   TATACCAATAATTGTATGCTGCGCTATCTTCTATTATATTACTCAAGGTTTCTCCATCTT

              830       840        850       860       870       880
IbMYB2-4  TTCTTCACTTTTTTTTTT.CGGAATTTTGGTGCTACCACACCCAAGTAGCCTACCTATAC
IbMYB2-1  TTCTTCACTTTTTTTTTT.CGGAATTTTGGTGCTACCACACCCAAGTAGCCTACCTATAC
IbMYB2s   TTCTTCACTTTTTTTTTTTCGGAATTTTGGTGCTACCACACCCAAGTAGCCTACCTATAC

               890       900       910       920       930
IbMYB2-4  TACAACAACCTCAGCTAATTAAGAATTTCCGACACCCTTCAATATATATA..........
IbMYB2-1  TACAACAACCTCAGCTAATTAAGAATTTCCGACACCCTTCAATATATATA..........
IbMYB2s   TACAACAACCTCAGCTAATTAAGAATTTCCGACACCCTTCAATATATATAATGGTTATTT
```

**IbMYB2-4**	. . . . .
**IbMYB2-1**	. . . . .
**IbMYB2s**	C A T C T

Fig S11. InDels of 3' flanking sequence of *IbMYB2s* gene. 3'UTR1~3'UTR5 were the 3' flanking sequences of *IbMYB2s* that cloned from genome in this study, *IbMYB2-1* and *IbMYB2-4* were the sequences that reported in references.

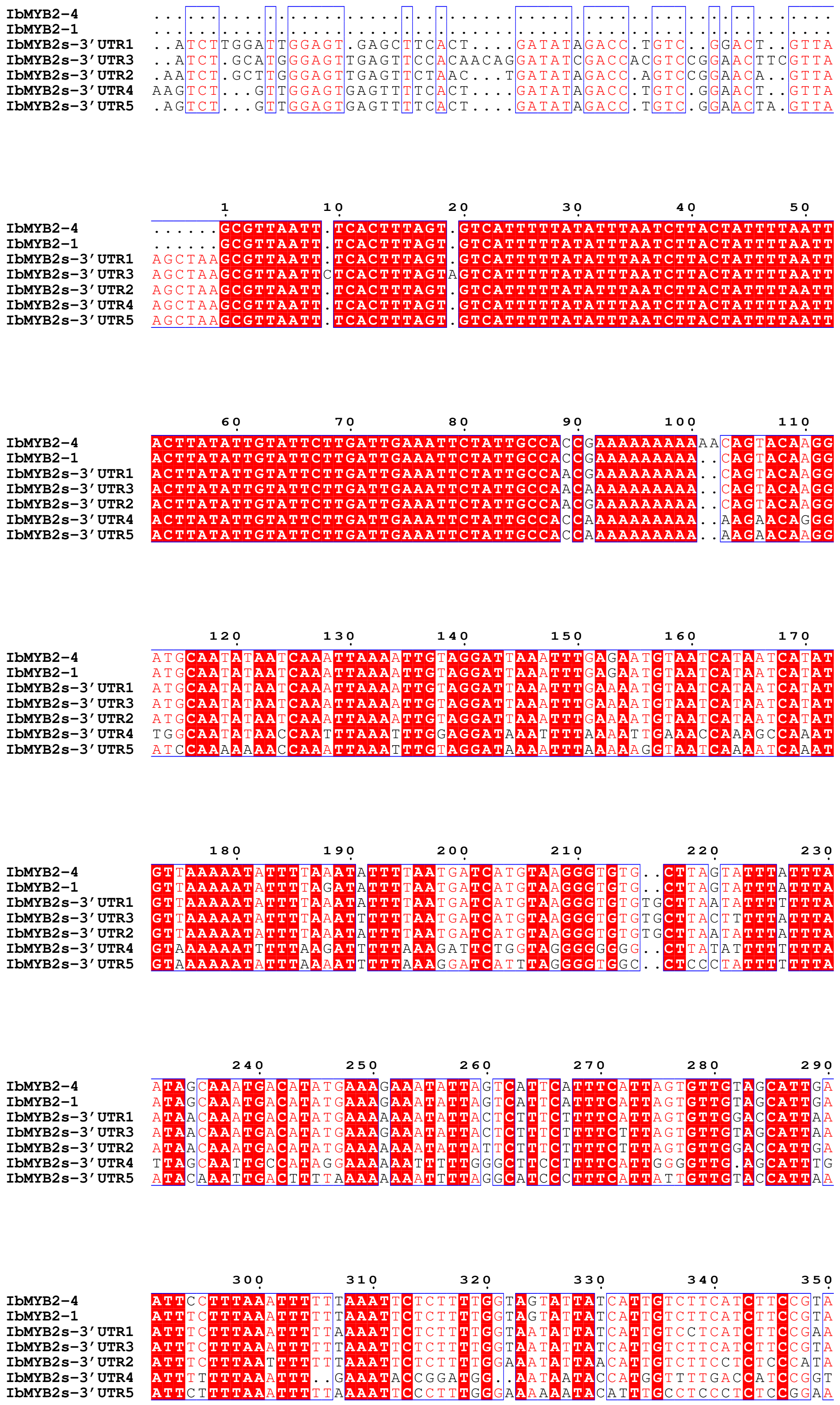

```
                  360       370       380       390       400       410
IbMYB2-4          CTTGTTTTTATTTTTTTATTTATTTATTTATTGGTAATGTGTCATGTGCAATTGGGTTAT
IbMYB2-1          ACTTGTTTTATTTATTTATTTATTTATTTATTGGTAATGTGTCATGTGCAATTGGGTTAT
IbMYB2s-3'UTR1    CTTGTTTA.........ATTTATTTATTTATTGGTAAGGTGTCATGTGCACTTGTGTTAT
IbMYB2s-3'UTR3    CTTGTTTT.........ATTTATTTATTTATTGGTAAGGGGTCATGTGCAATTGGGTATT
IbMYB2s-3'UTR2    CTTGTTTT.........ATTTATTTATTTATTGGAAAAGGGTCATGTGCAATTGGGTTAT
IbMYB2s-3'UTR4    TTG..TTTAATTCATTCATC..TGTTCAAAAGGGGATGACTGCCTATATCACCGATATAC
IbMYB2s-3'UTR5    CTGGTTTTTATTTATTTTTTGTTATGGTTAATGGTACATGGGCGTAAACAATGGGTTTAT

                  420       430       440       450       460
IbMYB2-4          CGATGGTAGTATAGAGGGTATGCCTGGTTACATGTTGTGGATTTGTCTCGTTG...TTGT
IbMYB2-1          CGATAGTAGTATAGAGGGCATGCCTGGTTACATGTTGTGGATTTGTCTCGTTG...TTGT
IbMYB2s-3'UTR1    CGATAATAATATGGAGGGCANNNNNNNNNNNNNNNNNNNNNNNNNNNNNNNNN...NNNN
IbMYB2s-3'UTR3    CGTAATTACTAAAGTGGGTATGCCTGGTTACATGTTGTGTATTTGTCTCGTTG...TTGT
IbMYB2s-3'UTR2    CGATAAACCAATAGAGGGCTTGCCTGGCTACTTGTTTTGCATTTGTCTCCTTG...TTAT
IbMYB2s-3'UTR4    C.ATACTAACATTTA.TGACCGTTATGCTTCTGTTTAAGCATGTGAGTATGCA.CAGTAC
IbMYB2s-3'UTR5    A.AGCGGATTCTTTGGTTCTTCCCAAGGCCTTTTTTGTGCTTTTGTCTGTTTATCATTCC

                  470       480       490       500       510       520
IbMYB2-4          GTGTGTATATGACTGAGATCTTAGT.TGTACTATGAACTTTTGATCTTGCAAGTGCCTGT
IbMYB2-1          GTGTGTATATGGCTGAGATCTTAGT.TGTACTATGAACTTTTGATCTTGCAAGTGCCTGT
IbMYB2s-3'UTR1    NNNNNNNNNNNNNNNNNNNNNNNNN.NNNNNNNNNNNNNNNNNNNNNNNNNNNNNNNNNN
IbMYB2s-3'UTR3    GTGTGTATATCACTGAAATCATAAT.TCTAAAATAAATTTTAATCTTGCAAGTGGCTGG
IbMYB2s-3'UTR2    GTTTGTCTATGACTGAAATCTTAAT.TATCGTATAAACTTTTAATCTTTCAACTGCCTGT
IbMYB2s-3'UTR4    GATGAAATC..ATCAACATCTAAGTACTTCATCTACTCGCAGGGTCATACTACTAT..GT
IbMYB2s-3'UTR5    GGTAATGTCTTATTACTATATTAACCTTTAATTTTTAAAATTGTCTATGGATTTATTAAT

                   530       540          550       560
IbMYB2-4          TGATATTTGGTAGTGATA.....GTGTTTTCAATTCCCTACATGTT..............
IbMYB2-1          TGATATTTGGTAGTGATA.....GTGTTTTCAATTCCTTACATGTT..............
IbMYB2s-3'UTR1    NNNNNNNNNNNNNNNNNN...NNNNNNNNNNNNNNNNNNNNNNGA...............
IbMYB2s-3'UTR3    TGATCTTTGTTGGTGTCA...CATGCTCTACACACCCTTATTTGATCCATGTGTTCGCAC
IbMYB2s-3'UTR2    TGATATTTTCTACCAATAATTTTTGGTTCCTTAATCTTTGATTGTACAGGTTGTTTGTGN
IbMYB2s-3'UTR4    CTCCTTATGCTGACCTCAGCTCAGTATATGACCTCATGGACAGGATTATCCCTGCATTCC
IbMYB2s-3'UTR5    CTTTCTTTATTTTTTTCGTTTTTCTCTCTCATCTTACTCAC.GCCTTNNNNNNNNNNNNN

                        570       580       590       600         610       620
IbMYB2-4          ......TCATGTGTTCTGAGATGTGTGTTGTACATTCCCATC.TGAAAGTATTGAACAAA
IbMYB2-1          ......TCATGTGTTCTGAGATGTGTGTTGTACATTCCCATC.TGAAAGTATTGAACAAA
IbMYB2s-3'UTR1    ...........................................................G
IbMYB2s-3'UTR3    AGGCTGTTGAATGTCCATCCTGGAACACTGCTCCCTCATATT.TATATTGATTATGCGAG
IbMYB2s-3'UTR2    NNNNNNNNNNNNNNNNNNNNNNNNNNNNNNNNNNNNNNNNNN.NNNNNNNNNNNNNNNNN
IbMYB2s-3'UTR4    ATTCCACGCTACTATACTATCTATTCATAATTCCCAATGACTGCCTAAATTATGGCTGCC
IbMYB2s-3'UTR5    NNNNNNNNNNNNNNNNNNNNNNNNNNNNNNNNNNNNNNNNNNNNNNNNNNNNNNNNNNNN

                           630                 640       650       660       670
IbMYB2-4          ATATTAATAT..........TTATTATATTTGTAGAAGGATAATGTGTAATCATAATGAC
IbMYB2-1          ATATTAATAT..........TTATTATATTTGTAGAAGGATAATGTGTAGT......ACC
IbMYB2s-3'UTR1    AAGTGATC..................AATATCAAGAGTGTAAAAATATTGTAGACAATCC
IbMYB2s-3'UTR3    ATATGATCCTAGGAGCCCCTCTGTTANNNNNNNNNNNNNNNNNNNNNNNNNNNNNNNNNN
IbMYB2s-3'UTR2    NNNNNNNNNNNNNNNNNNNNNNNNNNNNNNNNNNNNNNNNNNNNNNNNNNNNNNNNNNNN
IbMYB2s-3'UTR4    TTCTCGTCAGAGTCTGTAAATAGATTCTCCTCCTGANNNNNNNNNNNNNNNNNNNNNNNN
IbMYB2s-3'UTR5    NNNNNNNNNNNNNNNNNNNNNNNNNNNNNNNNNNNNNNNNNNNNNNNNNNNNNNNNNNNN

                       680       690          700       710       720
IbMYB2-4          TTTGTATGTAGTCCGAATACTGATGCT...TATTTGATAGTAATCATAATGACTTTGTAT
IbMYB2-1          TTTTGTTCTAGTTTTATAACT..TGTT...TATTTGATAGTAATCATAATGACTTTGTAT
IbMYB2s-3'UTR1    ATAAGGTCGAGAGGGTGTGTTTTTTTT..TTTCTTATAAGAAAGAATAATATGTGAGAAA
IbMYB2s-3'UTR3    NNNNNNNNNNNNNNNNNNNNNNNNNNN..NNNNNNNNNNNNNNNNNNNNNNNNNNNNNNN
IbMYB2s-3'UTR2    NNNNNNNNNNNNNNNNGTGCCCAGGTAAGTTTTTCTTGAGAATGATGAACAGATAAAAGA
IbMYB2s-3'UTR4    NNNNNNNNNNNNNNNNNNNNNNNNNNNNNNNNNNNNNNNNNNNNNNNNNNNNNNNNNNNN
IbMYB2s-3'UTR5    NNNGGGTATTATATAAAAAAGGAGTGGGGAAAGCTGGAAGAAACATAAATATGAATATAT
```

```
                     730       740       750       760       770       780
IbMYB2-4          GTAGTCCGAATACTGATGCCGCAATATGTCTAGTTTCATCAACAGTTTTCACACTT...A
IbMYB2-1          GTAGTCCGAATCCTGATGCCTCAATATGTCTAGTTTCATCAACAGTTTTCACACTT...A
IbMYB2s-3'UTR1    GTTGTTCTCCATTGCATTGGGTGGTAGCGCGGAAAAATTAAAAAGTTTTAA.AT......
IbMYB2s-3'UTR3    NNNNNNNNNNNNNNNNNNNNNNNNNNNNNNNNNNNNNNNNNNNNGCGATAG.ATAA...A
IbMYB2s-3'UTR2    GAAGCAGAAAAAGGCACTTCATGTAAGAGAAACGTGAGTTGATTAAAATATCAGTA...A
IbMYB2s-3'UTR4    NNNNNNNNNNNNNNNNNNNNNNNNNNNNNNNNNNNNNNNNNNNNNNNNNNNNAAGTGCTA
IbMYB2s-3'UTR5    CTAGTTCCCGAGCATGTTTATTAGTGAGGTACAGTTATACTATGAAATTTTTAACT..TA

IbMYB2-4          TTT.........................................................
IbMYB2-1          TTT.........................................................
IbMYB2s-3'UTR1    ............................................................
IbMYB2s-3'UTR3    CATAGAACAGTAAGAAATAGTGTGAGTTAGTAAACATCAGACAGCAGAC........AAC
IbMYB2s-3'UTR2    CAGAAAAGACTGATACACACTGCAGACTAG.ACTTTCGGAAAAGATATC........TTC
IbMYB2s-3'UTR4    CATTAGAGCAAAAAATGTTCGGTAGGAAAGTATCACCTTTTCCACCACG..CTATTTAAC
IbMYB2s-3'UTR5    TATCGGC.CGGAAAATATTTGGCTGTGATAAAAAATCTATTCAAGGAATGTTTATTGTGT

                                   790       800          810
IbMYB2-4          ..............TGCACCTATTCATTTTG...ACATTCAT....ATTTTTG.......
IbMYB2-1          ..............TGCACCTATTCATTTTG...ACATTCAT....ATTTTTG.......
IbMYB2s-3'UTR1    ................TTTCTTTTGG.GAGG...GTAATCAC..................
IbMYB2s-3'UTR3    ACTACGAGTGATGACGTCTCTTGTTG.TCTG...ATAATCAA....GAGCTAATATATAG
IbMYB2s-3'UTR2    ATACCACCAGGAATCGTTTAAAATAGCTGTA...GTGCTACC....AGATCTGCAGGCAG
IbMYB2s-3'UTR4    CACGATAAATTAGGCAAATCTATTACATTGTCATGTCCTCGT....AGTTTAATGAGTAA
IbMYB2s-3'UTR5    TATGAGAGGTGTGGGGTACAT.TTCCATCTGGAAGTATTGATCAAAAGATTAATATTTAT

                                                              820       830
IbMYB2-4          .........................................GAATTAGTATGAATGCGTA
IbMYB2-1          .........................................GAATTAGTATGAATGCGTA
IbMYB2s-3'UTR1    ..........................................AGTCTCCAT.........
IbMYB2s-3'UTR3    TGAAAACACAAATGACAGAGGCCTCTACGATC.........AAGTCATCATAAACAATT
IbMYB2s-3'UTR2    ATAGGACAAAAACTCAATTGGACACAATGGCT.........GAATCATGAGCCG.AGCTG
IbMYB2s-3'UTR4    TAAAGTCTGGGAAAAGGAAGCTGGCAAGCCATAAAAAATAATGGTAGTCACCGAGAATAC
IbMYB2s-3'UTR5    TATATTTGTTGAAGAATAAGGTGTGGTGCGCT.........TTGTTTTATTTTATATCT

                    840       850       860       870       880       890
IbMYB2-4          ..TAGT.ACTTGATTTTGGGTGTGTGTGTGT..TTATTTTGTAATTTTTTGTTGTTGAAT
IbMYB2-1          ..TAGT.ACTTGATTTTGTGTGTGTGTGTGTGTTTATTTTGTAATTTTTTGTTGTTGAAT
IbMYB2s-3'UTR1    ............................................CTTC............
IbMYB2s-3'UTR3    ATTGTG.AGATGAGCGTGGGGTAGAGCACATACAAAAGCCGGAGCCTCTTGAGAGCAAAA
IbMYB2s-3'UTR2    AGCGCC.ACCACACTGAACGAAGGCGTCCAATACGATGCGAGATGCTATCCAGAGAACAT
IbMYB2s-3'UTR4    AGTGTTTACATAAGTATACAATGTGATTTTTTGTATATGATAGTATCTTATATGTGTCG
IbMYB2s-3'UTR5    AGTGT...CATTGATAGTAACCATAATGCCTTTGTATGTAGT....CCGAATACGCATGG

                                                                900       910
IbMYB2-4          TGTTAT.....................................TAATCTTGGAGTACCCA
IbMYB2-1          TGTTAT.....................................TAATCTTGGAGTACCCA
IbMYB2s-3'UTR1    .............................................CGTACT..TTTTTTT
IbMYB2s-3'UTR3    AATTGTGACAGGGTGCAAATCAACAGGAAAAACGACC.AAAAGGCGCAATGATGTTTTTT
IbMYB2s-3'UTR2    CATGGT.........CGATGTGGAAGGAAAAATGGAC.CCTCACTAGGTAGACGAGTCAT
IbMYB2s-3'UTR4    AATGAGGTTGATGGCATTGGCAATAGTAAGCATATGTCAGTCGTCTTACTCACGTTACAT
IbMYB2s-3'UTR5    CGCAATATGTAAAGTTTTATCAACAGTTTGGACGTAT.AATCTGCGCCTTCACATTCTGA

                                     920       930              940       950
IbMYB2-4          ...............CCGTTGCTTTTTCACCATA.......ATGCATGCACTTAAATAGT
IbMYB2-1          ...............CCGTTGCTTTTTCACCATA.......ATGCATGCACTTAAATAGT
IbMYB2s-3'UTR1    ...............TTTTTTATTTATTGGAAAA....GGGTCACGTGCAATTGGGTTTT
IbMYB2s-3'UTR3    ...............TTTTTTATTTACAGGTAAA....GGGGCAAGTGCACTTGGGTGTC
IbMYB2s-3'UTR2    ...............TTATTTTATTACCGCCAAA....CCGGTAGGC.CAATTGGGTCTT
IbMYB2s-3'UTR4    CAAATTGATGACACATTTTTGGTGTTATATGAAA.AACATATCACATGCCATTCGGCCAT
IbMYB2s-3'UTR5    CA..TTGATA....TTTTTTGATTTATTGTAAAGTAGCATGTCA.ATGCAATTGGGTGTG
```

```
                         960                970               980                 990           1000
IbMYB2-4          ..TTGTGACTTT...GTGCACAAATATTTTTA.....AATAGTGGCTATATCCTATATAC
IbMYB2-1          ..TTGTGACTTT...GTGCACAAACATTTTTA.....AACAGTGGCTACATCCTATATAC
IbMYB2s-3'UTR1    GGATATAGGAATAGAGGGCACCCCCGGTTCCA.....CTTTGTGGATTTGTTTGGTGTTG
IbMYB2s-3'UTR3    AATTATAGAAGTGGAGGGCCCCCCCGCGCCCA.....CCCTGTGGGTTTTTTTCGTGCTT
IbMYB2s-3'UTR2    AGTTATTATTTTAGAGGGCGTACGCGGTAAAG.....AATGGTGGGTTTAACAGGGGCAT
IbMYB2s-3'UTR4    TGTTCGCAGTGTAGAGGACAGGGCCCGTTTCATTCAAAATCATGGATTTGTCTGGTTTCA
IbMYB2s-3'UTR5    GGAGTATTGAGTGGTAATTTTGTGTTGTGAAATGTATAATCTGATAGTGATCTAGGTCTA

                   1010         1020          1030          1040              1050
IbMYB2-4          TAATTA.TTTTTGTTGTCAGTATTGATGCAGATTTTGCCTTTAAT.....CCAAGCATGT
IbMYB2-1          TAATTA.TTTTTGTTGTCAGTATTGATGCAGATTTTGCCTTTAAT.....CCAAGCATGT
IbMYB2s-3'UTR1    GGGGGGGTATATGAGGGGGATTTTAGTGTTATTTTGAACTTTTGTTTTTCCAAAGGCCCG
IbMYB2s-3'UTR3    TGGGGGGTCTAGGGCGGGGATTTTTGTTGAATTATGACCTTTTGTTTTTCCAAAGCCCCG
IbMYB2s-3'UTR2    TGGGGGGTTTTTGGCGTGGTTCTTGGGGTTCCTAGAAACTTTTATTTTTTCAAAGCCCGG
IbMYB2s-3'UTR4    GTGTGCGTCTATGACTGAGATATTAGTTATACTATGAACTTTTGATCTTGCAAGTGCCTG
IbMYB2s-3'UTR5    ............................................................

               1060          1070          1080          1090           1100          1110
IbMYB2-4          TGAGTGTATAAAATGAAATGTATTGTAAATTTATATT.CGTTCTAATATATTTAAAGGAT
IbMYB2-1          TGAGTGTATAAAATGAAATGTATTGTAAATTTATATT.CGTTCTAATATATTTAAAGGAT
IbMYB2s-3'UTR1    TGGTAATTTGGGGGGGAAAGGGTTTTCAATTCCCTCCACGTTTCAGGGGTTTTGGGAGGG
IbMYB2s-3'UTR3    TGGAATTTTGGGGGGGATAGGGTTTTAAATCCCCCCCCCTTTTCAGTGGTTTTGGGAGGG
IbMYB2s-3'UTR2    TGGATTTTTGGGGTGGAGAGGGTTTTAAATTCCCTCCATGTTTCAGGGGTTTTGGGGGGG
IbMYB2s-3'UTR4    TTGATATTTGGTAGTGATAGTGTTTTCAATTCCCTACATGTTTCATGTGTTTTGAGATGT
IbMYB2s-3'UTR5    ............................................................

                 1120          1130           1140          1150               1160          1170
IbMYB2-4          GCACAATAAGTATT.TTACATACATGATTAATAAATGATGCA.....ACGATAGCTATA.
IbMYB2-1          GCACAATAAGTATT.TTACATACAGGATTAATAAATGATGCA.....ACGATAGCTATA.
IbMYB2s-3'UTR1    GGGTGGTCCATTCC.ATATGAAAGTATTGAACAAAATATTAATATTTATTATATTTGTAG
IbMYB2s-3'UTR3    GGGTGTTCCCTTCC.ATATGAAAGTATTGAACAAAATATTTATATTTTTTTTATTTGTAG
IbMYB2s-3'UTR2    GGGTGGTCCATTCC.ATATGAAAGTATTGAACAAAATATTAATATTTATTATATTTGTAG
IbMYB2s-3'UTR4    GTGTTGTACATTCCCATATGAAAGTATTGAACAAAATATTAATATTTATTATATTTGTAG
IbMYB2s-3'UTR5    ............................................................

                       1180          1190          1200          1210          1220          1230
IbMYB2-4          AGTACTAAAATATATAAATTTTTATTACAATAAGTATTTTTGGTTCAACTATTATGGTTG
IbMYB2-1          AGTACTAAAATATATAAATTTTTATTACAATAAGTATTTTTGGTTCAACTATTATGGTTG
IbMYB2s-3'UTR1    AAGGATAAAGTGTAGTACCCTTTGTTCTAGTTT.TATAACTTGTTTATTTGATAGTAATC
IbMYB2s-3'UTR3    AAGGATAAAGTGTAGTGCCCTTTGTTCTAGTTT.TATAACTTGTTTATTTGATAGTAATC
IbMYB2s-3'UTR2    AAGGATAAAGTGTAGTACCTTTTGTTCTAGTTT.TATAACTTGTTTATTTGATAGTAATC
IbMYB2s-3'UTR4    AAGGATAATGTGTAGTACCTTTTGTTCTAGTTT.TATAACTTGTTTACTTGATAGTAACC
IbMYB2s-3'UTR5    ............................................................

                       1240          1250           1260              1270          1280
IbMYB2-4          AATGAATTCTTGTAGATAGTG.AAATTCCTAGT...TAATTTAAGGAATAAGATTAAATA
IbMYB2-1          AATGAATTCTTGTAGATAGTG.AAATTCCTAGT...TAATTTAAGGAATAAGATTAAATA
IbMYB2s-3'UTR1    ATAAAGACTTTGTATGTAGTCTGAATATAGAGGCCGCAATATGTGTAGTTTCATCAACAG
IbMYB2s-3'UTR3    ATAAAGACTTTGTATGTAGTCCGAATATAGAGGCCGCAATATGTGTAGTTTCATCAACAC
IbMYB2s-3'UTR2    ATAAAGAGTTTGTATGTAGTCCGAATATTGAGGCCGCAATATGTGTAGTTTCATCAACAC
IbMYB2s-3'UTR4    ATAATGACTTTGTATGTAGTCCGAATACTGATGCCGCAATATGTCTAGTTTCATCAACAG
IbMYB2s-3'UTR5    ............................................................

                   1290          1300                1310                             1320
IbMYB2-4          AGATAATACAAATTT.......ATATATTTTAAA..CCATAT................A.
IbMYB2-1          AGGTAATACAAATTT.......ATATATTTTAAA..CCATATTAATAAAGAAAATATGA.
IbMYB2s-3'UTR1    TTTTCACACTTATTTTGCACCTATTCATTTTGACACTCATATTTTTGGAAATAGTATGAA
IbMYB2s-3'UTR3    TGTTCACACATATTTTGCACCTATTCACTTTGACACTCATATTTTTGGAGATAGTGTGAG
IbMYB2s-3'UTR2    TTTTCACACATATTTTGCACCTATTCATTTTGACACTCATATTTTTGGAAATAGTATGAA
IbMYB2s-3'UTR4    TTTTCACACTTATTTTGCACCTATTCATTTTGACATTCATATTTTTAGAATTAGTATGAA
IbMYB2s-3'UTR5    ............................................................
```

```
                           1330      1340      1350
                              .         .         .
IbMYB2-4          ..TATATA.TATATATATATATATATATATATATATATAT....................
IbMYB2-1          ..TATATA.TATATATATATATATATATATATATATATAT....................
IbMYB2s-3'UTR1    AGCGTATAGTACTTGAGTTTGTGTGTGTGTGTGTGTTTATTTTGTAATTTTTTGTTGTTG
IbMYB2s-3'UTR3    AGTGTATAGTACTTGATTTTGTGTGTGTGTGTGTGTTTATTTTGTAATTTTTTGTTGTTG
IbMYB2s-3'UTR2    AGCGTATAGTACTTGATTTTGTGTGTGTGTGTGTGTTTATTTTGTAATTTTTTGTTGTTG
IbMYB2s-3'UTR4    TGCGTATAGTACTTGATTTGTGTGTGTGTGTGT..TTATTTTGTAATTTTTTGTTGTTG
IbMYB2s-3'UTR5    ............................................................

IbMYB2-4          .........................................
IbMYB2-1          .........................................
IbMYB2s-3'UTR1    AATTGTTATAATCTGGAGACGCT.TGAGTATAG........
IbMYB2s-3'UTR3    AATTGTTATAATCTGGAGACCCAATACGTGCCTA.......
IbMYB2s-3'UTR2    AATTGTTATAATCTGGAGACGATTAGTATAGATACGGTACC
IbMYB2s-3'UTR4    AATTGTTATAATCTGAGACCCACTCAGCCTAC.........
IbMYB2s-3'UTR5    .........................................
```
