# Supplementary material for: The Expression of IbMYB1 Is Essential to Maintain the Purple Color of Leaf and Storage Root in Sweet Potato [Ipomoea batatas (L.) Lam]
Source: Front Plant Sci. 2021 Sep 23;12:688707. doi: 10.3389/fpls.2021.688707 (PMC8495246; doi:10.3389/fpls.2021.688707)
Supplement: Supplementary Table 2 — The amino acid sequence of the anthocyanin/flavonoid-related genes from the transcriptome data. [file Table_2.docx]

>IbPAL_g54983

MENGNGISSNGHAHAHDFCVEQKRRPSPSQHDPLNWNAAAEALKGSHLDEVKRMVEEYRTATVKLGGETLTIAQVAAVAA

RGTSEVTVELSEAARAGVKASSDWVMAGMENGTDSYGVTTGFGATSHRRTKQGAALQKELIRFLNAGIFGKGAESCHTLP

HSATRAAMLVRINTLLQGYSGIRFEILEAITKLLNHNITPCLPLRGTITASGDLVPLSYIAGLITGRPNSKAVGPNGEAL

DAEEAFRMAGIESGFFELQPKEGLAMVNGTAVGSGLASMVLFEANIMAVLSEVLSALFAEVMHGKPEFTDHLTHKLKHHP

GQIEAAAIMEHILDGSSYIKEATKTHELDPLQKPKQDRYALRTSPQWLGPQIEVIRAATKMIEREINSVNDNPLIDVSRN

KALHGGNFQGTPIGVSMDNVRLALAAIGKLMFAQFSELVNDYYNNGLPSNLSGGRNPSLDYGFKGAEIAMASYCSELQFL

GNPVTNHVQSAEQHNQDVNSLGLISSRKTAEALDILKLMSATYMVALCQAIDLRHLEENLKACTKNAVSQVAKKVLTVGE

NGELHPSRFSEKDLLKVVDREYVFAYADDPCSENYPLMQKLRQVLVDHALANGEAEMSSSTSIFHKIGAFEEELKAILPK

EIEGARCELESGNAAIPNRIQECRSFPLYKFVRAELGTNLLTGEKVRSPGEECDKVFTAMCEGKLIDPLLDCLKEWNGAP

LPIC

>IbPAL_g57979

MEGVIDNSHKNDFCMKVDPLNWEMAADSLKGSHLDEVKRMVAEFRNPAVKLGGQTLTVAQVAAIATRDNAVKVELSEAAR

AGVKASSDWVMNSMINGTDTYGITTGFGGTSHRRTKNGHALQQELIR

>IbPAL_g57980

MLGMFLNAGIFGIGTGKGSCHTLPHSATRAAMLVRINTLLQGYSGIRFEIMEAITKFLNHSITPCLPLRGSITASGDIVH

LSYIAALLTGRPNSKAVGPNGETLTAEEAFKLAGVQGGFFELQPKEGLALVNGTAVGSGMASMVLFEANVVAVLSEVLSA

IFAEVMHGRPEFTDHLTHKLKHHPGQIEAAAIMEHILDGSYFMKAAQKLHEMDPLEKPKQDSYALRTSPQWLGPQIEVIR

QATKMIEREINSVNDNPLIDVSRNRALHCGNFQGTPIGVSMDNSRLALASIGKLMFAQFSELVNGYYNNGLPSNLSAGRN

PSLDYGFKGAEVAMASYCSELQFLANPVTTHVQSAEQHNQGVNSLGLISAIKTEEAVDVLKLMSSTYLVALCQAIDLRHL

EENLKNAVKNTVSQAAKRTLTMGINEKDLVRVVDREYVFAYADDPCSANYPLFQKLRQVLVDHALQNGQHEKNVSTSIFQ

KIAAFEDELKAALPKEVEGARSALENGNPAIPNRISECRSYPLYKFVREELGTEMLTGEKVWSPGEVCDKVFTAVCEGGM

IDPLLECLKSWDGAPLPLTC

>IbPAL_g57981

MEGAIENGHNNDFCMKKVDPLNWEMAADSLKGSHLDEVKRMVAEFRNPAVKLGGQTLTVAQVAAIAAGDIAVKVELSQAA

RAGVKASSEWVINNGTGSNGFGANSHRRTKNGHALQQELIRNFGTGTETGSCHTLPHSATRAAMLVRINTLLQGYSGIRF

EILEAITKLLNHSITPCLPLRGSITASGDLVPLSYIAGLLTGRPNSKAVGPNGETLTAEEAFKLAGVQGGFFELQPKEGL

ALVNGTAVGSGMASMVLFEANVLAVLSEVLSAIFAEVMNGKPEFTDHLTHKLKHHPGQIEAAAIMEHILDGSYYMKAAQK

LHEMDPLQKPKQDRYALRTSPQWLGPQIEVIRQATKIIEREINSVNDNPLIDVSRNKSLQEEISKLVNDYYNNGLPSNLT

AGRDPSLDYGLKGAEIAMASYCSELQFLANPVTNHVQSAEQHNQDVNSLGLISARKTAEAVDVLKLMSSTYLVALCQAID

LRHLEENLKNAVKNTVSQVAKRTLTMGVNGELHPSRFCEKDLLRVVDREYVFAYADDPCSANYPLFQKLRQVLVDHALQN

GEHEKNVSTSIFQKIAAFEDELKAILPKEVEGARSALESGNPAIPNRISECRSYPLYKFVREELGTEMLTGEKVKSPGEL

CDKVFTAMCEGGMIDPLLECLKSWDGAPLPLIC

>IbPAL_g57984

MEGAIENGHNNDFCMKKEPAVKLGGQTLTVAQVAAIAAGNIAVKVELSQAARAGVKASSEWVINNGTGSNGFGANSHRRT

KNGHALQQELIRFLNVGIFGTGTETGSCHTLPHSATRAAMLVRVNTLLQGYSGIRFEILEAITKLLNHSITPCLPLRGSI

TASGDLVPLSYIAGLLTGRPNSKAVGPNGETLTAEEAFKLAGVQGGFFELQPKEGLALVNGTAVGSGMASMVLFEANVLA

VLSEVLSAIFAEVMNGKPEFTDHLTHKLKHHPGQIEAAAIMEHILDGSYYMKAAQKLHEMDPLQKPKQDRYALRTSPQWL

GPQIEIGPCLHWKTHLRQFSELVNDYYNNGLPSNLTAGRDPSLDYGLKGAEIAMASYCSELHFLANPVTNHVQSAEQHNQ

DVNSLAIDLRHLEENLKNAVKNTVSQVAKRTLTMGVNGELHPSRFCEKDLLRVVDREYVSPTLMILQRKLPIVSKAQASP

CRSRPAERPAREEYQPLSGNPAIPNRISECRSYPLYKFVREELGTEMLTGEKVKSPGELCDKVFTAMCEGGMIDPLLECL

KSWDGAPLPLISMLVLECRNFGTGTETGSCHTTSLSARAAMLVRINTLLQGYSGIRFEILEAITKLLNHSITPCLPLRGS

ITASGDLVPLSYIAGLLTGRPNSKAVGPNGETLTAEEAFKLAGVQGGFFELQPKEGLALVNGTAVGSGMASMVLFEANVL

AVLSEVLSAIFAEVMNGKHEFTDHLTHKLKHHPGQIEAAAIMEHILDGSYYMKAAQKLHEMDPLQKPKKIGGNFQGTPIG

VSMDNSRLALASIGKLIFAQFSELVNDYYNNGLPSNSPPGGTQVWIMDSRALKSPWLHTVPSFNSWLIPKTAEAVDVLKL

MSSTYLVALCQAIDLRHLEENLKNAVKNTVSQVAKRTLTMGVNGELQPSRFCEKDLVRVVDREYVFAYADDPCSANYPLF

QKLRQVLVDHALQNGDHEKNVSTSIFQKIAAFEDELKAALPKEVEGARSAIENGNPAIPNRITECRSYPLYKFVREELGT

EMLTGEKIKSPGELCDKVFTAMCEGGMIDPLLECLKSWDGAPLPLS

>IbPAL_g57986

MEGVIDNSHKNDFCVKVDPLNWEMAANSLKGSHLDEVKRMVAEFRNPAVKLGGQTLTVAQVAAIATRDNAVKVELSQAAR

AGVKASSDWVMNSMINGTDTYGITTGFGGTSHRRTKNGHALQQELIRFLNAGIFGIGTGKGSCHTLPHSATRAAMLVRIN

TLLQGYSGIRFEIMEAITKFLNHSITPCLPLRGSITASGDIVHLSYIAALLTGRPNSKAVGPNGETLTAEEAFKLAGVQG

GFFELQPKEGLALVNGTAVGSGMASMVLFEANVVAVLSEVLSAIFAEVMHGRPEFTDHLTHKLKHHPGQIEAAAIMEHIL

DGSYFMKAAQKLHEMDPLQKPKQDSYALRTSPQWLGPQIEVIRQATKMIEREINSVNDNPLIDVSRNRALHCGNFQGTPI

GVSMDNSRLALASIGKLMFAQFSELGAEVAMASYCSELQFLANPVTTHVQSAEQHNQVALCQAIDLRHLEENLKNAVKNT

VSQAAKRTLTMGINEKDLVRVVDREYVFAYADDPCSANYPLFQKLRQVLVDHALQNGQHEKNVSTSIFQKIAAFEDELKA

ALPKEVEGARSALENGNPAIPNRISECRSYPLYKFVREELGTEMLTGEKVWSPGEVCDKVFTAVCEGGMIDPLLECLKSW

DGAPLPLTC

>IbPAL_g57987

MEGAIENGHNNDFCMKKVDPLNWEMAADSLKGSHLDEVKRMVAEFRNPAVKLGGQTLTVAQVAAIAAGDNAVKVELSQAA

RAGVKASSEWVINNGTGSNGFGANSHRRTKNGHALQQELIRNFWDWNRNRVMSHTTSLSYKGSYASSLHVCLSAAPSPPP

VTLCLYPTSPVAHRPPQFKAVGPNGEPLTAEEAFKLAGVQGGFFELQPKEGLALVNGTAVGSGMASMVLFEANVLAVLSE

VLSAIFAEVMNGKPEFTDHLTHKLKHHPGQIEAAAIMEHILDGSYYMKAAQKLHEMDPLQKPKQDRYALRTSPQWLGPQI

EVIRQATKMIEREINSVNDNPLIDVSRNKSLQGGNFQGTPIGVSMDNSRLALASIGKLIFAQFSELVNDYYNNGLPSNLT

AGRDPSLDYGLKGAEIAMASYCSELQFLANPVTNHVQSAEQHNQDVNSLGLISARKTAEAVDVLKLMSSTYLVALCQAID

LRHLEENLKNAVKNTVSQVAKRTLTMGRKLPIVSKAQASPCRSRPAERRAREECEHINLPKIAAFEDELKAILPKEVEGA

RSALEMEILQFPTELASAGLILCTSLSAKNSGQRCSPERRSSRRENCVIRCSQQCVREG

>IbPAL_g57989

MEGAIANGHTNDFCIKVDPLNWEMAADSLKGSHLDEVKRMVAEFRNPAVKIGGQTLTVAQVAAIAARDNAVKVELSEAAR

PGVKASSDWVMNSMNNGTDSYGVTTGFGATSHRRTKNGHALQQELIRFLNAGIFGTGTGASHTLPHSATRAAMLVRINTL

LQGYSGIRFEILEAITKLLNHNITPCLPLRGTITASGDLVPLSYIAGLLTGRPNSKAVGPNGEALTAEEAFKLAGVQGGF

FELQPKEGLALVNGTAVGSGMASMVLFEANVLAVLSEVLSAIFAELLHEGCTEMHEMDPLQKPKQDRYALRTSPQWLGPQ

IEVIRQATKMIEREINSVNDNPLIDVSRNKALHGGNFQGTPIGVSMDNSRLALASIGKLIFAQFSELVNDYYNNGLPSNL

TAGRNPSLDYGFKGAEIAMASYCSELQFLANPVTNHVQSAEQHNQDVNSLGLISARKTAEAVDVLKLMSSTYLVALCQAI

DLRHLEENLKNAVKNTVSQVAKRTLTMGVNGELHPSRFCEKDLLRVVDREYVFAYADDPCSANYPLFQKLRQVLVDHALQ

NGEHEKNVSTSIFQKIAAFEDELKAVLPKEVEGARSAIENGNPAIPNRITECRSYPLYKFVREELGTEMLTGEKVKSPGE

VCDKVFTAVCDGGIIDPLLECLKSWDGAPLPIC

>IbPAL_g63872

MAPVVQNGVHQKKEAMEFCVKVDPLNWGAAAEAVKGSHLEEVKRMVAEFRKPVVKLGGETLTVAQVAAIASRDNNAVTVE

LSEEARPGVKASSDWVMESMGKGTDSYGVTTGFGATSHRRTKQGGALQKELIRFLNAGIFGNGGDSCHTLPHSATRAAML

VRINTLLQGYSGIRFEILEAITKFLNHNITPCLPLRGTITASGDLVPLSYIAGLLTGRPNSKAAGPNGESLTAEQAFRAA

GVDGGFFELQPKEGLALVNGTAVGSGMASMVLFEANVLALLSEVLSAIFAEVMNGKPEFTDHLTHKLKHHPGQIEAAAIM

EHILDGSSYVKSAEKLHEIDPLQKPKQDRYALRTSPQWLGPQIEVIRAATKMIEREINSVNDNPLIDVSRNKALHGGNFQ

GTPIGVSMDNARLALASIGKLIFAQFSELVNDYYNNGLPSNLTASRNPSLDYGLKGAEIAMASYCSELQFLANPVTNHVQ

SAEQHNQDVNSLGLISARKTAEAVEVLKLMSSTYLVGLCQAIDLRHLEENVKHAVKNSVSQVAKRTLTTGINGELHPSRF

CEKDLLRVVDREYVFAYADDPCSETYPLLQKLRQVLVDHALQNGEKEKDEGTSIFQKIAAFEDELRAVLPKEVEAARAAV

ESGNSAVPNKIKDCRSYPLYKFVREGLGTELLTGEKVRSPGEECDKVFTALCGGEIIDPLLECLESWNGAPLPIS

>IbPAL_g19510

MLVRINTLLQGYSGIRFEIMEAITKFLNHSITPCLPLRGSITASGDIVHLSYIAALLTGRPNSKAVGPNGETLTAEEAFK

LAGVQGGFFELQPKEGLALVNGTAVGSGMASMVLFEANVIAVLSEVLSAIFAEVMHGRPEFTDHLTHKLKHHPGQIEAAA

IMEHILDGSYFMKAAQKLHEMDPLEKPKQDSYALRTSPQWLGPQIEVIRQATKMIEREINSVNDNPLIDLSRNRALHCGN

FQGTPIGVSMDNSRLALASIGKLMFAQFSELVNGYYNNGLPSNLSAGRNPSLDYGFKGAEVAMASYCSELQFLANPVTTH

VQSAEQHNQVALCQAIDLRHLEENLKNAVKNTVSQAAKRTLTMGINEKDLVRVVDREYVFAYADDPCSANYPLFQKLRQV

LIDHALQNGQHEKNVSTSIFQKIAAFEDELKAILPKEVEGARSAFESGNPAIPNRISECRSYPLYKFVREELGTEMLTGE

KVKSPGEVCDKVFTAMCEGGMIDPLLECLKSWDGAPLPLIC

>IbPAL_g19515

MEGAIANGHNNDFCMKKVDPLNWEMAADSLKGSHLDEVKRMVADFRNPAVKLGGQTLTVAQVAAIAAGDIGVKVELSQAA

RAGVKASSDWVINNGTGSNGFGANSHRRTKNGHALQQELIRFLNVGIFGTGTETGSCHTLPHSATRAAMLVRINTLLQGY

SGIRFEILEAITKLLNHSITPCLPLRGSITASGDLVPLSYIAGLLTGRPNSKAVGPNGETLTAEEAFKLAGVQGGFFELQ

PKEGLALVNGTAVGSGMASMVLFEANVLAVLSEVLSAIFAEVMNGKPEFTDHLTHKLKHHPGQIEAAAIMEHILDGSYYM

KAAQKLHEMDPLQKPKQDRYALRTSPQWLGPQIEVIRQATKMIEREINSVNDNPLIDVSRNKALQGGNFQGTPIGVSMDN

SRLALASIGKLIFAQFSELVNDYYNNGLPSNLTAGRDPSLDYGFKGAEIAMASYCSELQFLANPVTNHVQSAEQHNQDVN

SLGLISARKTAEAVDVLKLMSSTYLVALCQAIDLRHLEENLKNAVKNTVSQVAKRTLTMGVNGELHPSRFCEKDLLRVVD

REYVFAYADDPCSANYPLFQKLRHVLVDHALQNGEHEKNVSTSIFQKIAAFEDELKAVLPKEVEGARSALENGNPAIPNR

ISECRSYPLYKFVREELGTEMLTGEKVKSPGELCDKVFTAMCEGGIVDPLLECLKSWDGAPLPLIXPSSRVRIDGEARVE

SSPKWDVGVGHAKSSPGKGMMGVEAKDRVGHNRESD

>IbPAL_g19518

MEGAIANGHTNDFCIKVDPLNWEMAADSLKGSHLDEVKRMVAEFRNPAVKIGGQTLTVAQVAAIAARDNAVKVELSEAAR

PGVKASSDWVMNSMNNGTDSYGVTTGFGATSHRRTKNGHALQQELIRFLNAGIFGTGTGASHTLPHSATRAAMLVRINTL

LQGYSGIRFEILEAITKLLNHNITPCLPLRGTITASGDLVPLSYIAGLLTGRPNSKAVGPNGEALTAEEAFKLAGVQGGF

FELQPKEGLALVNGTAVGSGMASMVLFEANVLAVLSEVLSAIFAEVMNGKPEFTDHLTHKLKHHPGQIEAAAIMEHILDG

SYYMKAAQKLHEMDPLQKPKQDRYALRTSPQWLGPQIEVIRQATKMIEREINSVNDNPLIDVSRNKALHGGNFQGTPIGV

SMDNSRLALASIGKLIFAQFSELVNDYYNNGLPSNLTAGRNPSLDYGFKGAEIAMASYCSELQFLANPVTNHVQSAEQHN

QDVNSLGLISARKTAEAVDVLKLMSSTYLVALCQAIDLRHLEENLKNAVKNTVSQVAKRTLTMGVNGELHPSRFCEKDLL

RVVDREYVFAYADDPCSANYPLFQKLRQVLVDHALQNGEHEKNVSTSIFQKIAAFEDELKAVLPKEVEGARSAIENGNPA

IPNRITECRSYPLYKFVREELGTEMLTGEKVKSPGEVCDKVFTAVCDGGIIDPLLECLKSWDGAPLPIC

>IbPAL_g25246

MDAVKVQNNGHQNGFCIKKQQVDPLNWEMAAESLRGSHLDEVKRMVAEFRKPAVKLGGETLTVAQVAAIASRDNAVAVEL

SEEARAGVKASSDWVMDSMNKGTDSYGVTTGFGATSHRRTKQGGALQKELIRFLNAGIFGNGTESCHTLPHSATRAAMLV

RINTLLQGYSGIRFEILEAITKLLNHNITPCLPLRGTITASGDLVPLSYIAGLITGRPNSKAVGPNGETLNAEEALRLAG

VDGGFFELQPKEGLALVNGTAVGSGMASMVLFEANVLAVLSEVLSAIFAEVMNGKPEFTDHLTHKLKHHPGQIEAAAIME

HILDGSSYVKAAQKLHEMDPLQKPKQDRYALRTSPQWLGPQIEVIRAATKMIEREINSVNDNPLIDVARSKALHGGNFQG

TPIGVSMDNSRLALASIGKLLFAQFSELVNDYYNNGLPSNLTAGRNPSLDYGFKGAEIAMASYCSELQFLANPVTNHVQS

AEQHNQDVNSLGLISARKTAEAVDVLKLMSSTYLVALCQAIDLRFLEENLRNAVKNAVTQVAKRTLTVGANGELHPARFS

EKDLLRVVDREYVFAYADDPCSANYPLMQKLRQALVDHALQNGENEKNTGTSIFLKVAAFEDELKAVLPKEVEAARIAVE

SGNPAIPNRIKECRSYPLYKFVREGLGTELLTGEKVRSPGEECDKVFTAMCEGSIIDPLLECLKSWDGAPLPIC

>IbC4H_g5098

MDLLLLEKTLLGFFVAIVVAIVVSKLRGKKYKLPPGPIPVPVFGNWLQVGDDLNHRNLTEYAKKFGDIFLLRMGQRNLVV

VSSPELAKEVLHTQGVEFGSRTRNVVFDIFTGKGQDMVFTVYGEHWRKMRRIMTVPFFTNKVVQQYREGWENEIASVVED

VKKNPEAATAGTVLRRRLQLMMYNNMYRIMFDRRFESEDDPLFNKLKALNGERSRLAQSFEYNYGDFIPILRPFLRGYLK

ICKEVKERRLQLFKDYFVDERKKLSSTKSMDTNSLKCAIDHILDAQQKGEINEDNVLYIVENINVAAIETTLWSIEWGIA

ELVNNPHIQKKLRDEIDTVLGPGVQITEPDTHKLPYLQAVIKETLRLRMAIPLLVPHMNLNDAKLGGYDIPAESKILVNA

WWLANNPAHWKKPEEFRPERFFEEEKHVEANGNDFRYLPFGVGRRSCPGIILALPILGIVLGRLVQNFELLPPPGQSKVD

TSEKGGQFSLHILKHSTIVMKPRTF

>IbC4H_g48358

MDLLLLEKTLLGLFFAILVAIVVSKLRGKKYKLPPGPLPVPVFGNWLQDGAEEPGGGVVAGASEGGSPHARGGVRLPNPE

RGVRYLHRERAGHGVHRLRRPLAEDAADHDGALLHQQGCAAVPARVGGRDRVGGGGGEEESGGGDSTGIVLRRRLQLMMY

NNLFRIMFDRRFESEEDPMFKKLRALNGERSRLAQSFDYNYGDFIPILRPFLRGYLKICKEVKERRLQLFKDHFVEERKK

LSSTKGMDNNSLKCAIDHILEAQQKGEINEDNVLYIVENINVAAIETTLWSIEWGIAELVNNPGIQKKLREEIDAVLGPG

VQLTEPDTHKLPYLQAVIKETLRLRMAIPLLVPHMNLHDAKLGGYDIPAESKILVNAWWLANNPSTWKNPEEFRPERFFE

EEKHVEANGNDFRFLPFGVGRRSCPGIILALPILGIVLGRLVQNFELLPPPGHSKVDTTEKGGQFSLHILKHSTIVMKPR

CF

>IbC4H_g48365

MDLLLLEKTLLGLFFAILVAIVVSKLRGKKYKLPPGPLPVPVFGNWLQVGDDLNHRNLTDYAKKFGDIFLLRMGQRNLVV

VSSPELAKEVLHTQGVEFGSRTRNVVFDIFTGKGQDMVFTVYGDHWRKMRRIMTVPFFTNKVVQQYRRGWEDEIGLVVEE

VKKNPEAASTGIVLRRRLQLMMYNNLFRIMFDRRFESEEDPMFKKLRALNGERSRLAQSFDYNYGDFIPILRPFLRGYLK

ICKEVKERRLQLFKDHFVEERKKLSSTKGMDNNSLKCAIDHILEAQQKGEINEDNVLYIVENINVAAIETTLWSIEWGIA

ELVNNPGIQKKLREEIDAVLGPGVQLTEPDTHKLPYLQAVIKETLRLRMAIPLLVPHMNLHDAKLGGYDIPAESKILVNA

WWLANNPSTWKNPEEFRPERFFEEEKHVEANGNDFRFLPFGVGRRSCPGIILALPILGIVLGRLVQNFELLPPPGHSKVD

TTEKGGQFSLHILKHSTIVMKPRCF

>Ib4CL_g1267

MGSNVGQTSTKQDDEHIFRSKYPPVQVPDDMTLPDFVLHNVELYADKVAIVDATTGKEFSYGQVARDVRRFAKALRSLGL

RKGRLVVVVLPNVAEYPIIALGIMAAGGVLSGANPTSHASELKKQVEVADAKLIVTDAPTYYKVKDLGLPVIVVDEERVQ

GTINWDELLEAADRASNDFAITEDEVVKQNDLCALPFSSGTTGMSKGVMLTHRNLVANLCSSLFSVGPEMIGQVIILGLI

PFFHIYGLVGICCATMRNKGKVVAMRRYELRAFLDALITHEVNFAPIVPPIILGLVKNPIVDEFDLSKLKLKSIMTAAAP

LAPEILNEFQKKFPGIQVQEAYGMTEHSCITLTHGDPRKGHGIAKKNSVGFILPNLEVKFIDPDSGLSLRRNTPGEICVR

SQCVMKGYYNNEYETALTIDKDGWLHTGDIGYIDEDGDIFIVDRIKELIKFKGFQVAPAELEATLLSHPSVEDAAVVGLP

DEEAGEVPAACVVLSAGAKDSEEDIMNYVASSVAHYKRVRVLHFVDAIPKSHSGKIMRRVIKDKMVQNLTTASSHDVLKK

LLA

>IbCHS-E_g42716

MVTVEEVRKAQRAQGPATIMAIGTSTPPNCVDQSTYPDYYFRITNSDHMVELKEKFKRMCEKSMISKRYMHLTEEILKEN

PNICAYMAPSLDARQDIVVVEVPKLGKEAAQKAIKEWGQPKSKITHLVFCTTSGVDMPGADYQLTKLLGLQPSVKRFMMY

QQGCFAGGTVLRLAKDLAENNKGARVLVVCSEITAVTFRGPSDAHLDSLVGQALFGDGAAALIIGSDPVAEIERPLFQLV

SAAQTILPDSGGAIDGHLREVGLTFHLLKDVPGLISKHIEKSLNEAFEPLGIRDWNSLFWIAHPGGPAILDQVEAKLELK

PEKLRATRHVLSEYGNMSSACVLFILDEMRKASSKEGLNTTGEGLEWGVLFGFGPGLTVETVVLHSVSA

>IbCHS-D_g8138

MVTVEEVRKAQRAEGPATILAIGTVTPANCVNQSTYPDYYFRITNSEHKTELKEKFQRMCDKSMITKRYMHLTEEILKEN

PSFCEYMAPSLDARQDIAVVEVPKLGKEAAQSAIKEWGQPKSKITHVVFCTTSGVDMPGADYQLTKLLGLRPSVKRLMMY

QQGCFAGGTVLRLAKDLAENNKGARVLIVCSEITVVTFRGPSETHLDSLLVSAAQTLAPNSCGAIDGHLREVGLTFHLLK

DVPSVVSNNIEKCLFEAFNPLGISDWNSVFWIAHPGGPAILDQVEDKLGLKPEKLRATRHVLSEYGNMSSACVLFILDEM

RKASSNAGLGTTGEGLEWGVLFGFGPGLTIETVVLHSVPIKPGPH

>IbCHI_g3524

MSAPPCVAEVKVESYVFPATAKPPGTTKTLILGGAGARGLNIDGKFVKFTAIGVYLEADAVPSLAVKWNGKSAEELTDSV

QFFRDVVTGPFEKLTRITMILPLSGKQYSEKVSENCVAFWKAAGIYGDAESKAIEKFNDVFSDQMFPPGASIFFTQSPLG

SLTISFSKDGSMPEIASAVIENKPLSEAVLESIIGSKGVSPEAKQSLAVRLSELFKNGVNGGDAITGKVGCENDAIPQTV

VSK

>IbCHI_g3586

MSAPPCAAEVKVESYVFPATAKPPGTAKTLILGGAGARGLNIDGKFVKFTAIGVYLEADAVPSLAVKWNGKSAEELTDSV

QFFRDIVTGPFEKLTRITMILPLSGKQYSEKVSENCVAFWKAAGIYGDAESKAIEKFNDVFSDQIPVGFNFLGVIESSQD

EMILVGGDNTDGVGHSSNNLTQYSPIHYELQISFSKEGSMPEIASAVIENKPLSEAVLESIIGSKGVSPEAKQSLAVRLS

ELFKNGVSGGDAITGKVGCENDVIPQTVVSK

>IbF3H_g29398

MAATLSTLTALAGEKALQSSFVRDEDERPKVGYNEFSDEIPVISLKGIDDVNGRRVQIRNDIVKACEDWGIFQVVDHGVD

AGLIGEMTRLSKDFFALPPEEKLRFDMSGGKKGGFIVSSHLQGEAVKDWREIVTYFSYPVRARDYSRWPDKPEGWRAVTE

KYSEKLMDLACKLLEVLSEAMGLEKEALTKACVDMDQKVVVNFYPKCPQPDLTLGLKRHTDPGTITLLLQDQVGGLQATK

DGGKTWITVQPVDGAFVVNLGDHGHFLSNGRFKNADHQAVVNSERSRVSIATFQNPAPEATVYPLTVREGDKPILEEPIT

FAEMYRRKMSKGSGACKAQEVRKGAAANHKGCYCG

>IbF3-H_g55963

MVEEMMVLAGSFNIGDFIPVLGLFDLQGIVGKMKKLHSRFDSFLNTILEEHKFVNNQHRTLSKDVDFLSTLIRLRDNGAD

MDGEEGKLTDTEIKALFLVAGTDTSSTAVEWAFAELLRNPKILNQAQQELDLVVGPNRLVTESDLTQLPFLQAIVKETFR

LHPSTPLSLPRMAAQSCEINGYFIPKGATLLVNVWAIARDPNVWTNPLEICSGMRLGIRMVHLLVATLVHAFDWDLGNGQ

SVETLNMEEAYGLTLQRAVPLMLHSKPRIRGIT

>IbF3-H_g55964

MLDGIFVQRSVLEGNSVQLVVFVGKSKLTDRVKRYPLPLPPGPKPWPVVGNLPHLGEKPHQSIAALAQSYGPLMHLRLGF

VHVVVAASAAVAAQFLKVHDANFSNRPPNSGAKHIAYNYQDLVFAPYGPRWRRLRKITSVHLFSAKALDDFSHVRQGEVA

TLTRSLASAGKTPVKLGQLLNVCTTNALARVMLGRKVFNDGSSKSDPKAEEFKSMVEEMMVLAGSFNLGDFIPVLGLFDL

QGIVAGTDTSSSTVEWAFAELLRNPKILKQAQQELDSVVGPNRLVSESDLTQLPFLQAIVKETFRLHPSTPLSLPRMAAQ

SCEINGHFIPKGATLLVNVWAIARDPNVWTNPLEFNPHRFLPGGEKPSVDIKGNDFEVIPFGAGRRICSGMSLGIRMVHL

LIATLVHAFDWDLGNGQSVETLNMEEAYGLTLQRAVPLMLHPKPRLQPHLYTLN

>IbDFR_g17019

MVGGNHALASPAPTVCVTGAAGFVGSWLVMKLLQRGYIVHATVRDPEIGNYAIEAHQCWQTGKHRILTYIDNVGDTNKVK

HLLELPKAEGKLKVWKGVLEEEGSFDEAIAGCEGVFHVAAAVNFASKDPENEVIKPAVKGILSIINSCAKAKTVKKLVFT

SSTAAVHIKQTQQLVYDESSWSDLDFIYANKMGGWMYFASKTLAEKEAWKAAKEKQIEFISIIPPLVIGPFLIPTFPLSL

VTALSPIMDPVGNGFHHNIIKQGKFVHLDDLCEAQIFLYQHPKAQGRFICSSHHATIHDVAKMIRHNWPEYYVPSEFKGI

EKELPIVSFSSKKLQEMGFEFKYTLEDMYRGAIETLRKKGLLPFSTKEPADIEQEQHSGKEPKS

>IbDFR_g17020

MVDGNHPKVVCVTGAAGFIGSWGVMEEEGSFDEAIAGCEGVFHVATPMDFDSKDPENEVIKPAINGVLNIINSCVKAKTV

KRLVFTSSAGTLNVQPQQKPVYDESCWSDLDFIYAKKMTGWMYFASKILAEKEAWKATKEKKIDFISIIPPLVVGPFITP

TFPPSLITALSLITGNQAHYSIIKQGQYVHLDDLCEAHIFLYEHPKAEGRFICSSHHTTIHGLGEMIRQNWPEYYIPSQF

KGIEKDLPVVYFSSKKLQDMGFQFKYSLEDMYRGAIETLRKKGLLPYSTKEPAAIEEEQETVPLKVQEPTKQEATTVPLK

PAAIEQKQETVPLKLEEPTAIEQKQKVVPLKA

>IbDFR_g17021

MSGGGLPAPKVCVTGAAGYVGSWLVMKLLQRGYVVHATVRDPGNTKKVKHLLELPKAEGNLRLWKGVLEEEGSFDDAIAG

CEGVFHVAATPVDFVSDDPQNEIIRPAVKGILSIINSCAKAKTVKRLVFTSSAVTLLVQENPKPVYDESSWSDLDLIYAK

KMPGWMYFASKTQAEKEAWKAAKEKQIDFISIIPPLVIGPSIVPTVPLSSIIALSPVTGNEAHYFVIKQGQYVHLDDLCE

AQIFLFEHPKAEGRFICSSHDATIYDLAKMIRQNWPEYYVPSEFKGIEKDLPVVSLSSKKLLNMGFQFKYTLEDMYREAI

ETLRNKCVLPYSIKPPPKEQEWENGKTLEA

>IbANR_g54088

MEGSGGDGTAMRGSKTYCVTGGSGFIGSWLIKSLLRRGYDVHATVRNPEKALHLLKLSERLKLFKADLQEEGSFDEAVRS

CDGVFHVAASMEFGVEPNHNIDSYVQENVIDPAAKGTLNVLKSCLKANSVKRVIFTSSISTMTAKDSSGKWRPVVDESCN

IPTEHVWHTKPSGWVYALSKVFTEKAAIRFANENGIDLVSIITPTVAGPFLTPTVPSSIRMLLSLITGDVKLLPILAAVN

SRMGSIALVHIEDICSVHIFLMENARAEGRYICCTQNCSISELIAQLSQEYPVPTTHSLMMEKHDSQPPVISSKKLRDLG

YSFKYSVQDIIHDTLQSCKQQGFLSHTQXLWFSSVEVDRCSKYLIDFKIVTITCTKNFQHFLDGFWEEILCDFTWSNSPN

PTPNLFQIQRFQNSYHSKFKL

>IbANS_g15751

MVTTITVPSRVERLAGSGIERIPKEYIRPEEERPSIGDIFAEEKMGGGPQVPTVDLKGINSEDLEVREKCREELRKAAVD

WGVMHLVNHGIPEELTGRVKAAGEGFFGQPIEEKEKYANDQATGNVQGYGSKLANNASGQLEWEDYFFHCVFPEDKTDLS

IWPKTPSDYIDATREYAKQLRALTTTVLAVLSLGLGLEEGRLEKEVGGMEELLLQMKINYYPKCPQPELALGVEAHTDVS

ALTFILHNMVPGLQLFYEGKWVTAKCVPNSIIMHVGDTVEILSNGKYKSILHRGVVNREKVRVSWAVFCEPPKEKIILQP

LPETVSEADPPRFPPRTFAQHIKHKLFRLTDQEGADTPKPDE

>IbWDR_g20700

MENSTQGSNLRSENSVTYESPYPIFAMAVSSFAGAHHGLRRRSVAVGSFLEEYKNRVEILSFEEDTVTLKTNPGLAFDHP

YPPTKLMFHPNPTASMKSTDLLVSSGDYLRLWEVREASIEPVSTLNNSKTSEYCAPLTSFDWNEVEPRRIGTSSIDTTCT

IWDIEKGVVETQLIAHDKEVYDIAWGEAGVFASVSADGSVRIFDLRDKEHSTIIYESPHPDTPLLRLAWNKQDLRYMATI

LMDSNKVVILDIRSPAMPVAELERHNASVNAIAWAPQSSRHISSAGDDGQALIWELPTVAGPNGIDPMSMYSAGAEINQL

QWSAAQPDWIAIAFSNKLQMLKV

>IbWDR_g64148

MGASSDPNQDGSDEQQRRSEIYTYEAPWHIYAMNWSVRKDKRYRLAIASLLEQYPNRVEIVQLDDSNGEIRSDSNLSFEH

PYPPTKVIFIPDKECQKPDLLATSSDYLRVWRVADDNSRVEIKTVLNNNRNSEFSGPLTSFDWNEAEPKRIGTSSIDTTC

TIWDIERETVDTQLIAHDKEVYDIAWGGVGVFASVSADGSVRVFDLRDKEHSTIIYESSEPDTPLVRLGWNKQDPRYMAT

IIMDSSKVVVLDIRFPTLPVVELQRHQASVNAIAWAPHSSCHICTAGDDSQALIWDLSSMGQPIEGGLDPILAYTAGAEI

EQLQWSSSQPDWVAIAFSNKLQILRVCHASPLLPTLPIFQHLGGQCSTALGFCLVFMKRVHSRCGGNQKFFGNVNLYDGS

GLSNTRHVLATSEFQVRTVLDGVPS

>IbbHLH1_g16071

MARGEGFAENLREKLALAIRSIEWSYAIFWTISSAQPGVLEWGDGYYNGDIKTRKTVQAAETSTDQLGLQRTEHLRELYG

SLLAGETNLHAKIPSAALSPEDLTDTEWYFLVCMSFVFNIGQGLPGKALAKNQTVWLCNAPQADGRTVVCFPHLGGVIEL

GVTELVKEDLGLIQHLKTSYLDIPCPIVPGVPNYISTDDGNDETLSIADEQGGDEFKVKGATAEASQPPNCQIVEDDISN

CIHNSTNSSDCISQNYENPEKVSDFLNDEEMVNHSPPENQECNQESLAPLDNRGQGHDVHYQSILSSVLKSSHQFILGPY

FRNGNRESSFVGWKKEISSNTQTLRIETSQRLLKKVLSGVARMVCIPDTRKEGDGKNDPVDKVSILDETIEYLKNLKTRV

WEAESQKEGFELNARMGRNCKDCDDAERTSDNCGTNIIDNNKKPSSKKRKASKTEGASKSTAKNGSAREVAVSVTDEDVT

IEIGCQWSEGVLIKIIQALNNLHLDCETIQSSNSDDGTLSVSVKCKMKASKLTSPSPALIRQALKRVI

>IbbHLH1_g16106

MGGRRRLHGVAISLIMARGEGFAENLREKLALAIRSIEWSYAIFWTISSAQPGVLEWGDGYYNGDIKTRKTVQAAETSTD

QLGLQRTEHLRELYGSLLAGETNLHAKIPSAALSPEDLTDTEWYFLVCMSFVFNIGQGLPGKALAKNQTVWLCNAPQADG

RTVVCFPHLGGVIELGVTELVKEDLGLVQHLKTSYLDIPCPIVPGVPNYISTDDGNERDILNSKPNQDTLEASPKEENID

SPDNSSNGLEADEQGGDEFKVKGATAEASQPPNCQIVEDDISNCIHNSTNSSDCISQNYENPEKVSDFLNDEEMVNQSPP

ENQECNQESLAPLDNRGQGHDVHYQSILSSVLKSSHQFILGPYFRNGNRESSFVGWKKEISSNTQTLRIETSQRLLKKVL

SGVARMVCIPDARKEGDGKNDPCKLEADESDRSRVVSERRRREKINERFMILSSLIPSSGKVDKVSILDETIEYLKNLKT

RVWEAESQKEGFELNARMGRNCKDCDDAERTSDNCGTNIIDNNKKPSSKKRKASKTEGASKSTAKNGSAREVAVSVTDED

VTIEIGCQWSEGVLIKIIQALNNLHLDCETIQSSNGDDGTLSVSVKCKMKASKLTSPSPALIRQALKRVI

>IbbHLH2_g9534

MAAENPVGGRLESLLQTAVQSVQWTYSLFWKLCPHNGMLVWSDGYYNGAIKTRKTVQGTEVSAEEASLHRSQQIKELYES

LSSTAEESNGGSAGQQPPRRPSAALSPEDLTESEWFYLMCISFSFPSALGLPGKAYAKRQHIWLTGANEVESKVFSRAIL

AKTVVCIPLMDGVVELGTTERVKEDYEFIQLIKNHFMEPHPHHHHHHPKPALSEHSSSEPPSQQLHSPVMASGHHRRQDR

AEEEDEEEEEDEEEEEEDDEEDEEEELQSDADVSPKNQQTGDHVMAAAEEEEEGRRTLTVWWRKQRTWRRLAS

>IbbHLH2_g9535

MPKWSSRHLGALGGQCLPGGAPIMDEFSREETHYSETISSILRHQCGQWSEFSTTVAGDYVAHSATSAFSSWTTAATS

TCSTHRSSAQWILKYALLTVPFLHEKNSHGAAADGGDATIPSSKLCKAAPQEEPNANHVLAERRRREKLNERFIILRSLVPFVTKMDKASILGDTIEY

VKQLRRRIQELEAARGGAWEVDRQSITGGVTRKNPAQKCGASRTLMGPTLRKRGMRTAERPANDTAEDAVVQVEVSIIESDALVELRCTYREGLILEV

MQMLKELGLEITTVQSSVNGGIFCAELRAKLKENLKGRKATIMEVKKAIHSIIPQF
