# Supplementary material for: The Expression of IbMYB1 Is Essential to Maintain the Purple Color of Leaf and Storage Root in Sweet Potato [Ipomoea batatas (L.) Lam]
Source: Front Plant Sci. 2021 Sep 23;12:688707. doi: 10.3389/fpls.2021.688707 (PMC8495246; doi:10.3389/fpls.2021.688707)
Supplement: Supplementary Table 3 — The amino acid sequence for the evolutionary tree of MYBs from Ipomoea batatas, Ipomoea nil, Ipomoea triloba, Ipomoea trifida, and Arabidopsis thaliana. [file Table_3.docx]

>ATMYB84(AT3G49690)

MGRAPCCDKANVKKGPWSPEEDAKLKSYIENSGTGGNWIALPQKIGLKRCGKSCRLRWLNYLRPNIKHGGFSEEEENIICSLYLTIGSRWSIIAAQLPGRTDNDIKNYWNTRLKKKLINKQRKELQEACMEQQEMMVMMKRQHQQQQIQTSFMMRQDQTMFTWPLHHHNVQVPALFMNQTNSFCDQEDVKPVLIKNMVKIEDQELEKTNPHHHQDSMTNAFDHLSFSQLLLDPNHNHLGSGEGFSMNSILSANTNSPLLNTSNDNQWFGNFQAETVNLFSGASTSTSADQSTISWEDISSLVYSDSKQFF

>ATMYB111(AT5G49330)

MGRAPCCEKIGLKRGRWTAEEDEILTKYIQTNGEGSWRSLPKKAGLLRCGKSCRLRWINYLRRDLKRGNITSDEEEIIVKLHSLLGNRWSLIATHLPGRTDNEIKNYWNSHLSRKIYAFTAVSGDGHNLLVNDVVLKKSCSSSSGAKNNNKTKKKKKGRTSRSSMKKHKQMVTASQCFSQPKELESDFSEGGQNGNFEGESLGPYEWLDGELERLLSSCVWECTSEEAVIGVNDEKVCESGDNSSCCVNLFEEEQGSETKIGHVGITEVDHDMTVEREREGSFLSSNSNENNDKDWWVGLCNSSEVGFGVDEELLDWEFQGNVTCQSDDLWDLSDIGEITLE

>ATMYB78(AT5G49620)

MGDKGRSLKINKNMEEFTKVEEEMDVRRGPWTVEEDLELINYIASHGEGRWNSLARCAELKRTGKSCRLRWLNYLRPDVRRGNITLEEQLLILELHTRWGNSNEFDHKFSSSCEDRWSKIAQYLPGRTDNEIKNYWRTRVQKHAKQLKCDVNSQQFKDTMKYLWMPRLVERIQAASIGSVSMSSCVTTSSDQFVINNNNTNNVDNLALMSNPNGYITPDNSSVAVSPVSDLTECQVSSEVWKIGQDENLVDPKMTSPNYMDNSSGLLNGDFTKMQDQSDLNWFENINGMVPNYSDSFWNIGNDEDFWLLQQHQQVHDNGSF

>ATMYB38(AT2G36890)

MSLSLIITEPENSFSFPFFSFKKQNKDISTKTLRPRETDREMGRAPCCDKANVKRGPWSPEEDAKLKDYIEKQGTGGNWIALPHKAGLRRCGKSCRLRWLNYLRPNIRHGDFTEEEDNIIYSLFASIGSRWSVIAAHLQGRTDNDIKNYWNTKLKKKLIATMAPPPHHHLAIATSSSSASPSSSSHYNMINSLLPYNPSTNQLLTPHQGIMMTMMGQQQQLFYQEDMGNLVNSPNRNNLIMSHQEDNQEQSTNKGIMLLSDVRSGSSTTSTVTRVKMEHRDHDDHHHHHEEDERSMTSVVMEDYGMEEIKQLISSSCTSSNNSLWFDENKTEDKFMLYY

>ATMYB11(AT3G62610)

MGRAPCCEKVGIKKGRWTAEEDRTLSDYIQSNGEGSWRSLPKNAGLKRCGKSCRLRWINYLRSDIKRGNITPEEEDVIVKLHSTLGTRWSTIASNLPGRTDNEIKNYWNSHLSRKLHGYFRKPTVANTVENAPPPPKRRPGRTSRSAMKPKFILNPKNHKTPNSFKANKSDIVLPTTTIENGEGDKEDALMVLSSSSLSGAEEPGLGPCGYGDDGDCNPSINGDDGALCLNDDIFDSCFLLDDSHAVHVSSCESNNVKNSEPYGGMSVGHKNIETMADDFVDWDFVWREGQTLWDEKEDLDSVLSRLLDGEEMESEIRQRDSNDFGEPLDIDEENKMAAWLLS

>ATMYB13(AT1G06180)

MGRRPCCEKIGLKKGPWSAEEDRILINYISLHGHPNWRALPKLAGLLRCGKSCRLRWINYLRPDIKRGNFTPHEEDTIISLHQLLGNRWSAIAAKLPGRTDNEIKNVWHTHLKKRLHHSQDQNNKEDFVSTTAAEMPTSPQQQSSSSADISAITTLGNNNDISNSNKDSATSSEDVLAIIDESFWSEVVLMDCDISGNEKNEKKIENWEGSLDRNDKGYNHDMEFWFDHLTSSSCIIGEMSDISEF

>ATMYB62(AT1G68320)

MENSMKKKKSFKESEDEELRRGPWTLEEDTLLTNYILHNGEGRWNHVAKCAGLKRTGKSCRLRWLNYLKPDIRRGNLTPQEQLLILELHSKWGNRWSKIAQYLPGRTDNEIKNYWRTRVQKQARQLNIESNSDKFFDAVRSFWVPRLIEKMEQNSSTTTTYCCPQNNNNNSLLLPSQSHDSLSMQKDIDYSGFSNIDGSSSTSTCMSHLTTVPHFMDQSNTNIIDGSMCFHEGNVQEFGGYVPGMEDYMVNSDISMECHVADGYSAYEDVTQDPMWNVDDIWQFRE

>ATMYB101(AT2G32460)

MDGGGETTATATMEGRGLKKGPWTTTEDAILTEYVRKHGEGNWNAVQKNSGLLRCGKSCRLRWANHLRPNLKKGSFTPDEEKIIIDLHAKLGNKWARMASQLPGRTDNEIKNYWNTRMKRRQRAGLPLYPHEIQHQGIDIDDEFEFDLTSFQFQNQDLDHNHQNMIQYTNSSNTSSSSSSFSSSSSQPSKRLRPDPLVSTNPGLNPIPDSSMDFQMFSLYNNSLENDNNQFGFSVPLSSSSSSNEVCNPNHILEYISENSDTRNTNKKDIDAMSYSSLLMGDLEIRSSSFPLGLDNSVLELPSNQRPTHSFSSSPIIDNGVHLEPPSGNSGLLDALLEESQALSRGGLFKDVRVSSSDLCEVQDKRVKMDFENLLIDHLNSSNHSSLGANPNIHNKYNEPTMVKVTVDDDDELLTSLLNNFPSTTTPLPDWYRVTEMQNEASYLAPPSGILMGNHQGNGRVEPPTVPPSSSVDPMASLGSCYWSNMPSIC

>ATMYB41(AT4G28110)

MGRSPCCDKNGVKKGPWTAEEDQKLIDYIRFHGPGNWRTLPKNAGLHRCGKSCRLRWTNYLRPDIKRGRFSFEEEETIIQLHSVMGNKWSAIAARLPGRTDNEIKNHWNTHIRKRLVRSGIDPVTHSPRLDLLDLSSLLSALFNQPNFSAVATHASSLLNPDVLRLASLLLPLQNPNPVYPSNLDQNLQTPNTSSESSQPQAETSTVPTNYETSSLEPMNARLDDVGLADVLPPLSESFDLDSLMSTPMSSPRQNSIEAETNSSTFFDFGIPEDFILDDFMF

>ATMYB109(AT3G55730)

MEGETHQSEPLPLASGDSDEGISAAIEAELAELAAGDSSGGGGCGGGGGGIRSKVKGPWSTEEDAVLTKLVRKLGPRNWSLIARGIPGRSGKSCRLRWCNQLDPCLKRKPFSDEEDRMIISAHAVHGNKWAVIAKLLTGRTDNAIKNHWNSTLRRKYADLWNNGQWMANSVTTASVKNENVDETTNPPSSKQQLPQGDINSSPPKPPQVSDVVMEEAANEPQEPQEQQEQAPPVVSNVPTENNVFRPVARVGAFSIYNPTSQKNGYRDYNIVPCEGPLIQAAKPDSLAGKFLQSLCDEPQIPSKCGHGCSTLPAETKFSRNSVLGPEFVDYEEPSAVFNQELISIATDLNNIAWIKSGLDNAVVREAEQSLKMDNYNYNDPRIKFTGMMPRQDFFCARS

>ATMYB17(AT3G61250)

MGRTPCCDKIGLKKGPWTPEEDEVLVAHIKKNGHGSWRTLPKLAGLLRCGKSCRLRWTNYLRPDIKRGPFTADEEKLVIQLHAILGNRWAAIAAQLPGRTDNEIKNLWNTHLKKRLLSMGLDPRTHEPLPSYGLAKQAPSSPTTRHMAQWESARVEAEARLSRESMLFSPSFYSGVVKTECDHFLRIWNSEIGEAFRNLAPLDESTITSQSPCSRATSTSSALLKSSTNSWGGKEVTVAIHGSDYSPYSNDLEDDSTDSALQLLLDFPISDDDMSFLEENIDSYSQAPPIGLVSMVSKF

>ATMYB6(AT4G09460)

MGRSPCCEKAHTNKGAWTKEEDQRLVDYIRNHGEGCWRSLPKSAGLLRCGKSCRLRWINYLRPDLKRGNFTDDEDQIIIKLHSLLGNKWSLIAGRLPGRTDNEIKNYWNTHIKRKLLSHGIDPQTHRQINESKTVSSQVVVPIQNDAVEYSFSNLAVKPKTENSSDNGASTSGTTTDEDLRQNGECYYSDNSGHIKLNLDLTLGFGSWSGRIVGVGSSADSKPWCDPVMEARLSLL

>ATMYB45(AT3G48920)

MVFKSEKSNREMKSKEKQRKGLWSPEEDEKLRSHVLKYGHGCWSTIPLQAGLQRNGKSCRLRWVNYLRPGLKKSLFTKQEETILLSLHSMLGNKWSQISKFLPGRTDNEIKNYWHSNLKKGVTLKQHETTKKHQTPLITNSLEALQSSTERSSSSINVGETSNAQTSSFSPNLVFSEWLDHSLLMDQSPQKSSYVQNLVLPEERGFIGPCGPRYLGNDSLPDFVPNSEFLLDDEISSEIEFCTSFSDNFLFDGLINELRPM

>ATMYB19(AT5G52260)

MTKSGERPKQRQRKGLWSPEEDQKLKSFILSRGHACWTTVPILAGLQRNGKSCRLRWINYLRPGLKRGSFSEEEEETILTLHSSLGNKWSRIAKYLPGRTDNEIKNYWHSYLKKRWLKSQPQLKSQISDLTESPSSLLSCGKRNLETETLDHVISFQKFSENPTSSPSKESNNNMIMNNSNNLPKLFFSEWISSSNPHIDYSSAFTDSKHINETQDQINEEEVMMINNNNYSSLEDVMLRTDFLQPDHEYANYYSSGDFFINSDQNYV

>ATMYB86(AT5G26660)

MGRHSCCFKQKLRKGLWSPEEDEKLLNYITRHGHGCWSSVPKLAGLQRCGKSCRLRWINYLRPDLKRGAFSQDEESLIIELHAALGNRWSQIATRLPGRTDNEIKNFWNSCLKKKLRRKGIDPTTHKPLITNELQSLNVIDQKLTSSEVVKSTGSINNLHDQSMVVSSQQGPWWFPANTTTTNQNSAFCFSSSNTTTVSDQIVSLISSMSTSSSPTPMTSNFSPAPNNWEQLNYCNTVPSQSNSIYSAFFGNQYTEASQTMNNNNPLVDQHHHHQDMKSWASEILHYTEHNQSSETVIEAEVKPDIANYYWRSASSSSSPNQEAATLLHDANVEVYGKNLQKLNNMVFDQSL

>ATMYB26(AT3G13890)

MGHHSCCNKQKVKRGLWSPEEDEKLINYINSYGHGCWSSVPKHAGTYTHIHGFCLQRCGKSCRLRWINYLRPDLKRGSFSPQEAALIIELHSILGNRWAQIAKHLPGRTDNEVKNFWNSSIKKKLMSHHHHGHHHHHLSSMASLLTNLPYHNGFNPTTVDDESSRFMSNIITNTNPNFITPSHLSLPSPHVMTPLMFPTSREGDFKFLTTNNPNQSHHHDNNHYNNLDILSPTPTINNHHQPSLSSCPHDNNLQWPALPDFPASTISGFQETLQDYDDANKLNVFVTPFNDNAKKLLCGEVLEGKVLSSSSPISQDHGLFLPTTYNFQMTSTSDHQHHHRVDSYINHMIIPSSSSSSPISCGQYVIT

>ATMYB25(AT2G39880)

MNGEISRPPELISSRNPCKSFENAIHKAVEAELAELAKSDANGGGKSKVKGPWLPEQDEALTRLVKMCGPRNWNLISRGIPGRSGKSCRLRWCNQLDPILKRKPFSDEEEHMIMSAQAVLGNKWSVIAKLLPGRTDNAIKNHWNSNLRRKPAEQWKIPLLMSNTEIVYQLYPSMVRRISNASPKEHLPQEEETGVLSDDKMDDEAKEPPREQNSKTGVYRPVARMGAFSVCKPGYMAPCEGPLVQASRPDSLAGKFLQSLCYDPIIPSKCGHGCCNHQDSTTLSSSSVLGSEFVDYEEHSSAELDKELISISNDLNNTAWIRSGKEAEQSLKADDQFRREYAHSKFSGMVNNGVSSQMVRQDLRALS

>ATMYBCDC5(AT1G09770)

MRIMIKGGVWKNTEDEILKAAVMKYGKNQWARISSLLVRKSAKQCKARWYEWLDPSIKKTEWTREEDEKLLHLAKLLPTQWRTIAPIVGRTPSQCLERYEKLLDAACTKDENYDAADDPRKLRPGEIDPNPEAKPARPDPVDMDEDEKEMLSEARARLANTRGKKAKRKAREKQLEEARRLASLQKRRELKAAGIDGRHRKRKRKGIDYNAEIPFEKRAPAGFYDTADEDRPADQVKFPTTIEELEGKRRADVEAHLRKQDVARNKIAQRQDAPAAILQANKLNDPEVVRKRSKLMLPPPQISDHELEEIAKMGYASDLLAENEELTEGSAATRALLANYSQTPRQGMTPMRTPQRTPAGKGDAIMMEAENLARLRDSQTPLLGGENPELHPSDFTGVTPRKKEIQTPNPMLTPSMTPGGAGLTPRIGLTPSRDGSSFSMTPKGTPFRDELHINEDMDMHESAKLERQRREEARRSLRSGLTGLPQPKNEYQIVAQPPPEESEEPEEKIEEDMSDRIAREKAEEEARQQALLKKRSKVLQRDLPRPPAASLAVIRNSLLSADGDKSSVVPPTPIEVADKMVREELLQLLEHDNAKYPLDDKAEKKKGAKNRTNRSASQVLAIDDFDENELQEADKMIKEEGKFLCVSMGHENKTLDDFVEAHNTCVNDLMYFPTRSAYELSSVAGNADKVAAFQEEMENVRKKMEEDEKKAEHMKAKYKTYTKGHERRAETVWTQIEATLKQAEIGGTEVECFKALKRQEEMAASFRKKNLQEEVIKQKETESKLQTRYGNMLAMVEKAEEIMVGFRAQALKKQEDVEDSHKLKEAKLATGEEEDIAIAMEASA

>AT3G12820(AT3G12820)

MGNRRAPCCDKSQVKRGPWSDEESERLRSFILKNGHQNWRSLPKLAGLMRCGKSCRLRWINYLRPGLKRGNFTKEEEDTIIHLHQAYGNKWSKIASNFPGRTDNEIKNVWNTHLKKRLVKRSISSSSSDVTNHSVSSTSSSSSSISSVLQDVIIKSERPNQEEEFGEILVEQMACGFEVDAPQSLECLFDDSQVPPPISKPDSLQTHGKSSDHEFWSRLIEPGFDDYNEWLIFLDNQTC

>ATMYB2(AT2G47190)

MEDYERINSNSPTHEEDSDVRKGPWTEEEDAILVNFVSIHGDARWNHIARSSGLKRTGKSCRLRWLNYLRPDVRRGNITLEEQFMILKLHSLWGNRWSKIAQYLPGRTDNEIKNYWRTRVQKQAKHLRCDVNSNLFKETMRNVWMPRLVERINAQSLPTTCEQVESMITDPSQPVNEPSPVEPGFVQFSQNHHQQFVPATELSATSSNSPAETFSDVRGGVVNGSGYDPSGQTGFGEFNDWGCVGGDNMWTDEESFWFLQDQFCPDTTSYSYN

>ATMYB3R2(AT4G00540)

MTESIDLNRSESESDNNTDDVTPIFAIDDSSKGRVSGPTRRSTKGGWTAEEDQILTNVVKKYQGRNWKRIAECLPGSEENRRNDVQCQHRWLKVLDPSLQKGAWKKEEDELLSELVKDYMENDRPPWSKISKELPGRIGKQCRERWHNHLNPTIIKSPWTREEELILVQAQRGNGNKWAEIAKLLPGRTENNIKNHWNCSVKKRLEQFPSNLFSGVVYGSKPSSGFEYNFFNQRNTMVESCITSQIKEAAKSPQRDFLDLTLGLNWRSISSSTSSLRGEESVSSSVDSVCARLNACLETPQNSNNDTVCVKEVREMKERLRMAARTFDTPSIISKTSSPASGLKRLRQKYDTPFPTDARSHMSSEEDHSVSASPSSKYRFVKRNTCSGSKPLERRLDFDFLLWDEHGRRNGIVNFSVRILPQKSDLKSGLVRPFWLR

>ATMYB90(AT1G66390)

MEGSSKGLRKGAWTAEEDSLLRLCIDKYGEGKWHQVPLRAGLNRCRKSCRLRWLNYLKPSIKRGRLSNDEVDLLLRLHKLLGNRWSLIAGRLPGRTANDVKNYWNTHLSKKHESSCCKSKMKKKNIISPPTTPVQKIGVFKPRPRSFSVNNGCSHLNGLPEVDLIPSCLGLKKNNVCENSITCNKDDEKDDFVNNLMNGDNMWLENLLGENQEADAIVPEATTAEHGATLAFDVEQLWSLFDGETVELD

>ATMYB12(AT2G47460)

MGRAPCCEKVGIKRGRWTAEEDQILSNYIQSNGEGSWRSLPKNAGLKRCGKSCRLRWINYLRSDLKRGNITPEEEELVVKLHSTLGNRWSLIAGHLPGRTDNEIKNYWNSHLSRKLHNFIRKPSISQDVSAVIMTNASSAPPPPQAKRRLGRTSRSAMKPKIHRTKTRKTKKTSAPPEPNADVAGADKEALMVESSGAEAELGRPCDYYGDDCNKNLMSINGDNGVLTFDDDIIDLLLDESDPGHLYTNTTCGGDGELHNIRDSEGARGFSDTWNQGNLDCLLQSCPSVESFLNYDHQVNDASTDEFIDWDCVWQEGSDNNLWHEKENPDSMVSWLLDGDDEATIGNSNCENFGEPLDHDDESALVAWLLS

>ATMYB4(AT4G38620)

MGRSPCCEKAHTNKGAWTKEEDERLVAYIKAHGEGCWRSLPKAAGLLRCGKSCRLRWINYLRPDLKRGNFTEEEDELIIKLHSLLGNKWSLIAGRLPGRTDNEIKNYWNTHIRRKLINRGIDPTSHRPIQESSASQDSKPTQLEPVTSNTINISFTSAPKVETFHESISFPGKSEKISMLTFKEEKDECPVQEKFPDLNLELRISLPDDVDRLQGHGKSTTPRCFKCSLGMINGMECRCGRMRCDVVGGSSKGSDMSNGFDFLGLAKKETTSLLGFRSLEMK

>ATMYB79(AT4G13480)

MVEEVWRKGPWTAEEDRLLIEYVRVHGEGRWNSVSKLAGLKRNGKSCRLRWVNYLRPDLKRGQITPHEESIILELHAKWGNRWSTIARSLPGRTDNEIKNYWRTHFKKKAKPTTNNAEKIKSRLLKRQHFKEQREIELQQEQQLFQFDQLGMKKIISLLEENNSSSSSDGGGDVFYYPDQITHSSKPFGYNSNSLEEQLQGRFSPVNIPDANTMNEDNAIWDGFWNMDVVNGHGGNLGVVAATAACGPRKPYFHNLVIPFC

>ATMYB104(AT2G26950)

MIQDQANDLLAMKKTFTKSKWKPEEDRILKDYVIQYGDRTWTHVPKRTGLPHNPASCRFRWMNHLKPSLKKGPFTDEEEKRVLQLHAVLGNKWSQMAREFPGRTDNEIKNFWNARRMRLKGKGLPVYPDEVREQAIRTAAQYGVKVELLNAHYSQDSLMAGNVEKPQELNNLALNQCSPYYQSTLANVQPSRNRVMEPETTFPFTGGSAMNEQNPTLLCNPYVESTQEQLPDSHLFGNVTYSSPPMPLIHEVENLELPSFQGFDFHEEPSSFGAEQYNPMLNLEPHNTLVQSPLIGQTPTDFPSSFYDELLDELLESVVNGSLGEIPKTDTSSESQLFQSSLRSHTDATPDIANTTGYVGSNERNTTNDDDWIRLLLDEGFI

>ATMYB103(AT5G56110)

MGRIPCCEKENVKRGQWTPEEDNKLASYIAQHGTRNWRLIPKNAGLQRCGKSCRLRWTNYLRPDLKHGQFSEAEEHIIVKFHSVLGNRWSLIAAQLPGRTDNDVKNYWNTKLKKKLSGMGIDPVTHKPFSHLMAEITTTLNPPQVSHLAEAALGCFKDEMLHLLTKKRVDLNQINFSNHNPNPNNFHEIADNEAGKIKMDGLDHGNGIMKLWDMGNGFSYGSSSSSFGNEERNDGSASPAVAAWRGHGGIRTAVAETAAAEEEERRKLKGEVVDQEEIGSEGGRGDGMTMMRNHHHHQHVFNVDNVLWDLQADDLINHMV

>ATMYB82(AT5G52600)

MECKREEGKSYVKRGLWKPEEDMILKSYVETHGEGNWADISRRSGLKRGGKSCRLRWKNYLRPNIKRGSMSPQEQDLIIRMHKLLGNRWSLIAGRLPGRTDNEVKNYWNTHLNKKPNSRRQNAPESIVGATPFTDKPVMSTELRRSHGEGGEEESNTWMEETNHFGYDVHVGSPLPLISHYPDNTLVFDPCFSFTDFFPLL

>ATMYB72(AT1G56160)

MGKGRAPCCDKNKVKRGPWSPQEDLTLITFIQKHGHQNWRSLPKLAGLLRCGKSCRLRWINYLRPDVKRGNFSKKEEDAIIHYHQTLGNKWSKIASFLPGRTDNEIKNVWNTHLKKRLTPSSSSSSLSSTHDQSTKADHDKNCDGAQEEIHSGLNESQNSATSSHHQGECMHTKPELHEVNGLNEIQFLLDHDDFDDITSEFLQDNDILFPLDSLLHNHQTHISTQEMTREVTKSQSFDHPQPDIPCGFEDTNEESDLRRQLVESTTPNNEYDEWFNFIDNQTYFDDFNFVGEVCL

>ATMYB46(AT5G12870)

MRKPEVAIAASTHQVKKMKKGLWSPEEDSKLMQYMLSNGQGCWSDVAKNAGLQRCGKSCRLRWINYLRPDLKRGAFSPQEEDLIIRFHSILGNRWSQIAARLPGRTDNEIKNFWNSTIKKRLKKMSDTSNLINNSSSSPNTASDSSSNSASSLDIKDIIGSFMSLQEQGFVNPSLTHIQTNNPFPTGNMISHPCNDDFTPYVDGIYGVNAGVQGELYFPPLECEEGDWYNANINNHLDELNTNGSGNAPEGMRPVEEFWDLDQLMNTEVPSFYFNFKQSI

>ATMYB95(AT1G74430)

MGRTTWFDVDGLRKGEWTAEEDRKLVVYINEHGLGEWGSLPKRAGLQRCGKSCRLRWLNYLRPGIKRGKFTPQEEEEIIKYHALLGNRWAAIAKQMPNRTDNDIKNHWNSCLKKRLAKKGIDPMTHEPTTTTSLTVDVTSSSTTSSPTPSPTSSSFSSCSSTGSARFLNKLAAGISSRKHGLESIKTVILAEQPREAVDEEKMMTINMKEKELISCYMEIDETMSIDELPCDDSTSGFVAFDDYSLIDPYRDGVYVSDFYDETEHLDLFLL

>ATMYB63(AT1G79180)

MGKGRAPCCDKTKVKRGPWSPEEDIKLISFIQKFGHENWRSLPKQSGMSLLLSSQSKQKPLQLFFLFFMILNVYICKNEGLLRCGKSCRLRWINYLRPDLKRGNFTSEEEETIIKLHHNYGNKWSKIASQLPGRTDNEIKNVWHTHLKKRLAQSSGTADEPASPCSSDSVSRGKDDKSSHVEDSLNRETNHRNELSTSMSSGGSNQQDDPKIDELRFEYIEEAYSEFNDIIIQEVDKPDLLEIPFDSDPDIWSFLDTSNSFQQSTANENSSGSRATTEEESDEDEVKKWFKHLESELGLEEDDNQQQYKEEESSSSSLLKNYELMIH

>ATMYB67(AT3G12720)

MREKWEMKRDEMGHRCCGKHKVKRGLWSPEEDEKLLRYITTHGHPSWSSVPKLAGLQRCGKSCRLRWINYLRPDLRRGSFNEEEEQIIIDVHRILGNKWAQIAKHLPGRTDNEVKNFWNSCIKKKLLSQGLDPSTHNLMPSHKRSSSSNNNNIPKPNKTTSIMKNPTDLDQSTTAFSITNINPPTSTKPNKLKSPNQTTIPSQTVIPINDNMSSTQTMIPINDPMSSLLDDENMIPHWSDVDGMAIHEAPMLPSDKAVVGVDDDDLNMDILFNTPSSSAFDPDFASIFSSAMSIDFNPMDDLGSWTF

>ATMYB110(AT3G29020)

MKMDFSCFQEYPFEFHCRGTTFNGFRENNAVSETVEEFCNKRRMQKKSDDLKTKKKKKQSVSRVCSRGHWRISEDTQLMELVSVYGPQNWNHIAESMQGRTGKSCRLRWFNQLDPRINKRAFSDEEEERLLAAHRAFGNKWAMIAKLFNGRTDNALKNHWHVLMARKMRQQSSSYVQRFNGSAHESNTDHKIFNLSPGNVDDDEDVNLKKCSWEMLKEGTTNLKAQYLQEEYSSSRMPMQGPHHHYSTFPADSLALTLHVSIQEPSSSSSLSLPSSSTTGEHTMVTRYFETIKPPAFIDFLGVGH

>ATMYB56(AT5G17800)

MNPNLLEKDLRGKETTNGSIRYKEANNFRSLPNSHTAACKTSLNNPSISRNHPHNKSASVLESEDEHGNERGENEKSLRMRGKSGINTKVCSRGHWRPTEDAKLKELVAQFGPQNWNLISNHLLGRSGKSCRLRWFNQLDPRINKRAFTEEEEFRLLAAHRAYGNKWALISRLFPGRTDNAVKNHWHVIMARRTRESQRQRQQPPPTLSRDAEMTVSSSCRYNQGKFINEEDDDDDVSAVSTCTTELSLTPPSSAYQPRFFNYDSTLASGKDGQCVQRAEVNGIYGKKMDHQNHHTISVSERKVEMKMRSGYYYFDFLGVGAS

>ATMYB112(AT1G48000)

MNISRTEFANCKTLINHKEEVEEVEKKMEIEIRRGPWTVEEDMKLVSYISLHGEGRWNSLSRSAGLNRTGKSCRLRWLNYLRPDIRRGDISLQEQFIILELHSRWGNRWSKIAQHLPGRTDNEIKNYWRTRVQKHAKLLKCDVNSKQFKDTIKHLWMPRLIERIAATQSVQFTSNHYSPENSSVATATSSTSSSEAVRSSFYGGDQVEFGTLDHMTNGGYWFNGGDTFETLCSFDELNKWLIQ

>ATMYB107(AT3G02940)

MGRSPCCDESGLKKGPWTPEEDQKLINHIRKHGHGSWRALPKQAGLNRCGKSCRLRWTNYLRPDIKRGNFTAEEEQTIINLHSLLGNKWSSIAGHLPGRTDNEIKNYWNTHIRKKLIQMGIDPVTHRPRTDHLNVLAALPQLLAAANFNNLLNLNQNIQLDATSVAKAQLLHSMIQVLSNNNTSSSFDIHHTTNNLFGQSSFLENLPNIENPYDQTQGLSHIDDQPLDSFSSPIRVVAYQHDQNFIPPLISTSPDESKETQMMVKNKEIMKYNDHTSNPSSTSTFTQDHQPWCDIIDDEASDSYWKEIIEQTCSEPWPFRE

>ATMYB1(AT3G09230)

MEAEIVRRSEVTGLRREVEESSIGRGDCDGDGGDVGEDAAGFVGTSGRGRRDRVKGPWSKEEDDVLSELVKRLGARNWSFIARSIPGRSGKSCRLRWCNQLNPNLIRNSFTEVEDQAIIAAHAIHGNKWAVIAKLLPGRTDNAIKNHWNSALRRRFIDFEKAKNIGTGSLVVDDSGFDRTTTVASSEETLSSGGGCHVTTPIVSPEGKEATTSMEMSEEQCVEKTNGEGISRQDDKDPPTLFRPVPRLSSFNACNHMEGSPSPHIQDQNQLQSSKQDAAMLRLLEGAYSERFVPQTCGGGCCSNNPDGSFQQESLLGPEFVDYLDSPTFPSSELAAIATEIGSLAWLRSGLESSSVRVMEDAVGRLRPQGSRGHRDHYLVSEQGTNITNVLST

>ATMYB40(AT5G14340)

MCDLLACLRKISMGRKPCCDKIGLKRGPWTIEEDHRLMNFILNNGIHCWRIVPKLAGLLRCGKSCRLRWINYLRPDLKRGGFTDAEEDRIMELHSQLGNRWSKIASHFSGRTDNEIKNHWNTKIKKKMKHLGLDPATHKPMNDITHQTDPNQDKKPNMCSTINEGEEIKDQTPKDDVITETTKTLMLSDNDEELVAKNCKILCAEEVDLESLFETQCNEISSSSFSSLCSNISRSESSSYLAEDSISLEQWDLDMTDPFVPWDLFANLDDNLFLL

>ATMYB14(AT2G31180)

MGRAPCCEKMGVKRGPWTPEEDQILINYIHLYGHSNWRALPKHAGLLRCGKSCRLRWINYLRPDIKRGNFTPQEEQTIINLHESLGNRWSAIAAKLPGRTDNEIKNVWHTHLKKRLSKNLNNGGDTKDVNGINETTNEDKGSVIVDTASLQQFSNSITTFDISNDNKDDIMSYEDISALIDDSFWSDVISVDNSNKNEKKIEDWEGLIDRNSKKCSYSNSKLYNDDMEFWFDVFTSNRRIEEFSDIPEF

>ATMYB55(AT4G01680)

MGRHSCCYKQKLRKGLWSPEEDEKLLRYITKYGHGCWSSVPKQAGTFLFIQIHLLFGLQRCGKSCRLRWINYLRPDLKRGAFSQDEENLIIELHAVLGNRWSQIAAQLPGRTDNEIKNLWNSCLKKKLRLRGIDPVTHKLLTEIETGTDDKTKPVEKSQQTYLVETDGSSSTTTCSTNQNNNTDHLYTGNFGFQRLSLENGSRIAAGSDLGIWIPQTGRNHHHHVDETIPSAVVLPGSMFSSGLTGYRSSNLGLIELENSFSTGPMMTEHQQIQESNYNNSTFFGNGNLNWGLTMEENQNPFTISNHSNSSLYSDIKSETNFFGTEATNVGMWPCNQLQPQQHAYGHI

>ATMYB0(AT3G27920)

MRIRRRDEKENQEYKKGLWTVEEDNILMDYVLNHGTGQWNRIVRKTGLKRCGKSCRLRWMNYLSPNVNKGNFTEQEEDLIIRLHKLLGNRWSLIAKRVPGRTDNQVKNYWNTHLSKKLVGDYSSAVKTTGEDDDSPPSLFITAATPSSCHHQQENIYENIAKSFNGVVSASYEDKPKQELAQKDVLMATTNDPSHYYGNNALWVHDDDFELSSLVMMNFASGDVEYCL

>ATMYB69(AT4G33450)

MEMSRGSNSFDNKKPSCQRGHWRPVEDDNLRQLVEQYGPKNWNFIAQHLYGRSGKSCRLRWYNQLDPNITKKPFTEEEEERLLKAHRIQGNRWASIARLFPGRTDNAVKNHFHVIMARRKRENFSSTATSTFNQTWHTVLSPSSSLTRLNRSHFGLWRYRKDKSCGLWPYSFVSPPTNGQFGSSSVSNVHHEIYLERRKSKELVDPQNYTFHAATPDHKMTSNEDGPSMGDDGEKNDVTFIDFLGVGLAS

>ATMYB120(AT5G55020)

MIMYGGGGAGKDGGSTNHLSDGGVILKKGPWTAAEDEILAAYVRENGEGNWNAVQKNTGLARCGKSCRLRWANHLRPNLKKGSFTGDEERLIIQLHAQLGNKWARMAAQLPGRTDNEIKNYWNTRLKRLLRQGLPLYPPDIIPNHQLHPHPHHQQQQQHNHHHHHHQQQQQHQQMYFQPQSSQRNTPSSSPLPSPTPANAKSSSSFTFHTTTANLLHPLSPHTPNTPSQLSSTPPPPPLSSPLCSPRNNQYPTLPLFALPRSQINNNNNGNFTFPRPPPLLQPPSSLFAKRYNNANTPLNCINRVSTAPFSPVSRDSYTSFLTLPYPSPTAQTATYHNTNNPYSSSPSFSLNPSSSSYPTSTSSPSFLHSHYTPSSTSFHTNPVYSMKQEQLPSNQIPQIDGFNNVNNFTDNERQNHNLNSSGAHRRSSSCSLLEDVFEEAEALASGGRGRPPKRRQLTASLPNHNNNTNNNDNFFSVSFGHYDSSDNLCSLQGKTKTTYNTSNLNYSSLQVKCKMFMIKTQI

>ATMYB59-3(AT5G59780)

MKLVQEEYRKGPWTEQEDILLVNFVHLFGDRRWDFVAKVSGLNRTGKSCRLRWVNYLHPGLKRGKMTPQEERLVLELHAKWGNRWSKIARKLPGRTDNEIKNYWRTHMRKKAQEKKRPMSPTSSSSNCCSSSMTTTTSQDTGGSNGKMNQECEDGYYSMDDIWREIDQSGANVIKPVKDNYYSEQSCYLNFPPLASPTWESSLESIWNMDADESKMSSFAIDQFPLSFEHGSGRL

>ATMYB76(AT5G07700)

MSKRPYCIGEGLKKGAWTTEEDKKLISYIHDHGEGGWRDIPEKAGLKRCGKSCRLRWTNYLKPDIKRGEFSYEEEQIIIMLHASRGNKWSVIARHLPKRTDNEVKNYWNTHLKKRLIDDGIDPVTHKPLASSNPNPVEPMKFDFQKKSNQDEHSSQSSSTTPASLPLSSNLNSVKSKISSGETQIESGHVSCKKRFGRSSSTSRLLNKVAARASSIGNILSTSIEGTLRSPASSSGLPDSFSQSYEYMIDNKEDLGTSIDLNIPEYDFPQFLEQLINDDDENENIVGPEQDLLMSDFPSTFVDEDDILGDITSWSTYLLDHPNFMYESDQDSDEKNFL

>ATMYB66(AT5G14750)

MRKKVSSSGDEGNNEYKKGLWTVEEDKILMDYVKAHGKGHWNRIAKKTGLKRCGKSCRLRWMNYLSPNVKRGNFTEQEEDLIIRLHKLLGNRWSLIAKRVPGRTDNQVKNYWNTHLSKKLGIKDQKTKQSNGDIVYQINLPNPTETSEETKISNIVDNNNILGDEIQEDHQGSNYLSSLWVHEDEFELSTLTNMMDFIDGHCF

>ATMYB113(AT1G66370)

MGESPKGLRKGTWTTEEDILLRQCIDKYGEGKWHRVPLRTGLNRCRKSCRLRWLNYLKPSIKRGKLCSDEVDLVLRLHKLLGNRWSLIAGRLPGRTANDVKNYWNTHLSKKHDERCCKTKMINKNITSHPTSSAQKIDVLKPRPRSFSDKNSCNDVNILPKVDVVPLHLGLNNNYVCESSITCNKDEQKDKLININLLDGDNMWWESLLEADVLGPEATETAKGVTLPLDFEQIWARFDEETLELN

>ATMYB65(AT3G11440)

MSYTTATADSDDGMHSSIHNESPAPDSISNGCRSRGKRSVLKKGPWTSTEDGILIDYVKKHGEGNWNAVQKHTSLARCGKSCRLRWANHLRPNLKKGAFSQEEEQLIVEMHAKMGNKWAQMAEHLPGRTDNEIKNYWNTRIKRRQRAGLPLYPPEIYVDDLHWSEEYTKSNIIRVDRRRRHQDFLQLGNSKDNVLFDDLNFAASLLPAASDLSDLVACNMLGTGASSSRYESYMPPILPSPKQIWESGSRFPMCSSNIKHEFQSPEHFQNTAVQKNPRSCSISPCDVDHHPYENQHSSHMMMVPDSHTVTYGMHPTSKPLFGAVKLELPSFQYSETSAFDQWKTTPSPPHSDLLDSVDAYIQSPPPSQVEESDCFSSCDTGLLDMLLHEAKIKTSAKHSLLMSSPQKSFSSTTCTTNVTQNVPRGSENLIKSGEYEDSQKYLGRSEITSPSQLSAGGFSSAFAGNVVKTEELDQVWEPKRVDITRPDVLLASSWLDQGCYGIVSDTSSMSDALALLGGDDIGNSYVTVGSSSGQAPRGVGSYGWTNMPPVWSL

>ATMYB14(AT1G18570)

MVRTPCCKAELGLKKGAWTPEEDQKLLSYLNRHGEGGWRTLPEKAGLKRCGKSCRLRWANYLRPDIKRGEFTEDEERSIISLHALHGNKWSAIARGLPGRTDNEIKNYWNTHIKKRLIKKGIDPVTHKGITSGTDKSENLPEKQNVNLTTSDHDLDNDKAKKNNKNFGLSSASFLNKVANRFGKRINQSVLSEIIGSGGPLASTSHTTNTTTTSVSVDSESVKSTSSSFAPTSNLLCHGTVATTPVSSNFDVDGNVNLTCSSSTFSDSSVNNPLMYCDNFVGNNNVDDEDTIGFSTFLNDEDFMMLEESCVENTAFMKELTRFLHEDENDVVDVTPVYERQDLFDEIDNYFG

>ATMYB3(AT1G22640)

MGRSPCCEKAHMNKGAWTKEEDQLLVDYIRKHGEGCWRSLPRAAGLQRCGKSCRLRWMNYLRPDLKRGNFTEEEDELIIKLHSLLGNKWSLIAGRLPGRTDNEIKNYWNTHIKRKLLSRGIDPNSHRLINESVVSPSSLQNDVVETIHLDFSGPVKPEPVREEIGMVNNCESSGTTSEKDYGNEEDWVLNLELSVGPSYRYESTRKVSVVDSAESTRRWGSELFGAHESDAVCLCCRIGLFRNESCRNCRVSDVRTH

>ATMYB30(AT3G28910)

MVRPPCCDKGGVKKGPWTPEEDIILVTYIQEHGPGNWRAVPTNTGLLRCSKSCRLRWTNYLRPGIKRGNFTEHEEKMIVHLQALLGNRWAAIASYLPQRTDNDIKNYWNTHLKKKLNKVNQDSHQELDRSSLSSSPSSSSANSNSNISRGQWERRLQTDIHLAKKALSEALSPAVAPIITSTVTTTSSSAESRRSTSSASGFLRTQETSTTYASSTENIAKLLKGWVKNSPKTQNSADQIASTEVKEVIKSDDGKECAGAFQSFSEFDHSYQQAGVSPDHETKPDITGCCSNQSQWSLFEKWLFEDSGGQIGDILLDENTNFF

>ATMYB96(AT5G62470)

MGRPPCCEKIGVKKGPWTPEEDIILVSYIQEHGPGNWRSVPTHTGLRRCSKSCRLRWTNYLRPGIKRGNFTEHEEKTIVHLQALLGNRWAAIASYLPERTDNDIKNYWNTHLKKKLKKINESGEEDNDGVSSSNTSSQKNHQSTNKGQWERRLQTDINMAKQALCEALSLDKPSSTLSSSSSLPTPVITQQNIRNFSSALLDRCYDPSSSSSSTTTTTTSNTTNPYPSGVYASSAENIARLLQDFMKDTPKALTLSSSSPVSETGPLTAAVSEEGGEGFEQSFFSFNSMDETQNLTQETSFFHDQVIKPEITMDQDHGLISQGSLSLFEKWLFDEQSHEMVGMALAGQEGMF

>ATMYB49(AT5G54230)

MGKSSSSEESEVKKGPWTPEEDEKLVGYIQTHGPGKWRTLPKNAGLKRCGKSCRLRWTNYLRPDIKRGEFSLQEEETIIQLHRLLGNKWSAIAIHLPGRTDNEIKNYWNTHIKKKLLRMGIDPVTHCPRINLLQLSSFLTSSLFKSMSQPMNTPFDLTTSNINPDILNHLTASLNNVQTESYQPNQQLQNDLNTDQTTFTGLLNSTPPVQWQNNGEYLGDYHSYTGTGDPSNNKVPQAGNYSSAAFVSDHINDGENFKAGWNFSSSMLAGTSSSSSTPLNSSSTFYVNGGSEDDRESFGSDMLMFHHHHDHNNNALNLS

>ATMYB42(AT4G12350)

MGRQPCCDKLMVKKGPWTAEEDKKLINFILTNGHCCWRALPKLAGLRRCGKSCRLRWTNYLRPDLKRGLLSDAEEQLVIDLHALLGNRWSKIAARLPGRTDNEIKNHWNTHIKKKLLKMEIDPSTHQPLNKVFTDTNLVDKSETSSKADNVNDNKIVEIDGTTTNTIDDSIITHQNSSNDDYELLGDIIHNYGDLFNILWTNDEPPLVDDASWSNHNVGIGGTAAVAASDKNNTAAEEDFPERSFEKQNGESWMFLDYCQEFGVEDFGFECYHGFGQSSMKTGHKD

>ATMYB94(AT3G47600)

MGRPPCCDKIGVKKGPWTPEEDIILVSYIQEHGPGNWRSVPTHTGLRRCSKSCRLRWTNYLRPGIKRGNFTEHEEKMILHLQALLGNRWAAIASYLPERTDNDIKNYWNTHLKKKLKKMNDSCDSTINNGLDNKDFSISNKNTTSHQSSNSSKGQWERRLQTDINMAKQALCDALSIDKPQNPTNFSIPDLGYGPSSSSSSTTTTTTTTRNTNPYPSGVYASSAENIARLLQNFMKDTPKTSVPLPVAATEMAITTAASSPSTTEGDGEGIDHSLFSFNSIDEAEEKPKLIDHDINGLITQGSLSLFEKWLFDEQSHDMIINNMSLEGQEVLF

>ATMYB7(AT2G16720)

MGRSPCCEKEHMNKGAWTKEEDERLVSYIKSHGEGCWRSLPRAAGLLRCGKSCRLRWINYLRPDLKRGNFTHDEDELIIKLHSLLGNKWSLIAARLPGRTDNEIKNYWNTHIKRKLLSKGIDPATHRGINEAKISDLKKTKDQIVKDVSFVTKFEETDKSGDQKQNKYIRNGLVCKEERVVVEEKIGPDLNLELRISPPWQNQREISTCTASRFYMENDMECSSETVKCQTENSSSISYSSIDISSSNVGYDFLGLKTRILDFRSLEMK

>ATMYB98(AT4G18770)

MENFVDENGFASLNQNIFTRDQEHMKEEDFPFEVVDQSKPTSFLQDFHHLDHDHQFDHHHHHGSSSSHPLLSVQTTSSCINNAPFEHCSYQENMVDFYETKPNLMNHHHFQAVENSYFTRNHHHHQEINLVDEHDDPMDLEQNNMMMMRMIPFDYPPTETFKPMNFVMPDEISCVSADNDCYRATSFNKTKPFLTRKLSSSSSSSSWKETKKSTLVKGQWTAEEDRVLIQLVEKYGLRKWSHIAQVLPGRIGKQCRERWHNHLRPDIKKETWSEEEDRVLIEFHKEIGNKWAEIAKRLPGRTENSIKNHWNATKRRQFSKRKCRSKYPRPSLLQDYIKSLNMGALMASSVPARGRRRESNNKKKDVVVAVEEKKKEEEVYGQDRIVPECVFTDDFGFNEKLLEEGCSIDSLLDDIPQPDIDAFVHGL

>ATMYB60(AT1G08810)

MGRPPCCDKIGIKKGPWTPEEDIILVSYIQEHGPGNWRSVPTNTGLLRCSKSCRLRWTNYLRPGIKRGNFTPHEEGMIIHLQALLGNKWASIASYLPQRTDNDIKNYWNTHLKKKLNKSDSDERSRSENIALQTSSTRNTINHRSTYASSTENISRLLEGWMRASPKSSTSTTFLEHKMQNRTNNFIDHHSDQFPYEQLQGSWEEGHSKGINGDDDQGIKNSENNNGDDVHHEDGDHEDDDDHNATPPLTFIEKWLLEETSTTGGQMEEMSHLMELSNML

>ATMYB88(AT2G02820)

MEETTKQNNMKKKKKILLHSDDSKKKERHIVTWSPEEDDILRKQISLQGTENWAIIASKFNDKSTRQCRRRWYTYLNSDFKRGGWSPEEDTLLCEAQRLFGNRWTEIAKVVSGRTDNAVKNRFTTLCKKRAKHEAMAKENRIACCVNSDNKRLLFPDGISTPLKAESESPLTKKMRRSHIPNLTEIKSYGDRSHIKVESTMNQQRRHPFSVVAHNATSSDGTEEQKQIGNVKESDGEDKSNQEVFLKKDDSKVTALMQQAELLSSLAQKVNADNTDQSMENAWKVLQDFLNKSKENDLFRYGIPDIDFQLDEFKDLVEDLRSSNEDSQSSWRQPDLHDSPASSEYSSGSGSGSTIMTHPSGDKTQQLMSDTQTTSHQQNGGELLQDNGIVSDATVEQVGLLSTGHDVLKNSNETVPIPGEEEFNSPVQVTPLFRSLAAGIPSPQFSESERNFLLKTLGVESPSPYPSANPSQPPPCKRVLLDSL

>ATMYB15(AT3G23250)

MGRAPCCEKMGLKRGPWTPEEDQILVSFILNHGHSNWRALPKQAGLLRCGKSCRLRWMNYLKPDIKRGNFTKEEEDAIISLHQILGNRWSAIAAKLPGRTDNEIKNVWHTHLKKRLEDYQPAKPKTSNKKKGTKPKSESVITSSNSTRSESELADSSNPSGESLFSTSPSTSEVSSMTLISHDGYSNEINMDNKPGDISTIDQECVSFETFGADIDESFWKETLYSQDEHNYVSNDLEVAGLVEIQQEFQNLGSANNEMIFDSEMDFWFDVLARTGGEQDLLAGL

>ATMYB103(AT1G63910)

MGHHSCCNQQKVKRGLWSPEEDEKLIRYITTHGYGCWSEVPEKAGLQRCGKSCRLRWINYLRPDIRRGRFSPEEEKLIISLHGVVGNRWAHIASHLPGRTDNEIKNYWNSWIKKKIRKPHHHYSRHQPSVTTVTLNADTTSIATTIEASTTTTSTIDNLHFDGFTDSPNQLNFTNDQETNIKIQETFFSHKPPLFMVDTTLPILEGMFSENIITNNNKNNDHDDTQRGGRENVCEQAFLTTNTEEWDMNLRQQEPFQVPTLASHVFNNSSNSNIDTVISYNLPALIEGNVDNIVHNENSNVQDGEMASTFECLKRQELSYDQWDDSQQCSNFFFWDNLNINVEGSSLVGNQDPSMNLGSSALSSSFPSSF

>ATMYB116(AT1G25340)

MSNITKKKCNGNEEGAEQRKGPWTLEEDTLLTNYISHNGEGRWNLLAKSSGKSCRLRWLNYLKPDIKRGNLTPQEQLLILELHSKWGNRWSKISKYLPGRTDNDIKNYWRTRVQKQARQLNIDSNSHKFIEVVRSFWFPRLINEIKDNSYTNNIKANAPDLLGPILRDSKDLGFNNMDCSTSMSEDLKKTSQFMDFSDLETTMSLEGSRGGSSQCVSEVYSSFPCLEEEYMVAVMGSSDISALHDCHVADSKYEDDVTQDLMWNMDDIWQFNEKILIFEDQTVLVLVSLDLLRRTNLISLLHVKESKR

>ATMYB29(AT5G07690)

MSRKPCCVGEGLKKGAWTAEEDKKLISYIHEHGEGGWRDIPQKAGLKRCGKSCRLRWANYLKPDIKRGEFSYEEEQIIIMLHASRGNKWSVIARHLPKRTDNEIKNYWNTHLKKLLIDKGIDPVTHKPLAYDSNPDEQSQSGSISPKSLPPSSSKNVPEITSSDETPKYDASLSSKKRCFKRSSSTSKLLNKVAARASSMGTILGASIEGTLISSTPLSSCLNDDFSETSQFQMEEFDPFYQSSEHIIDHMKEDISINNSEYDFSQFLEQFSNNEGEEADNTGGGYNQDLLMSDVSSTSVDEDEMMQNITGWSNYLLDHSDFNYDTSQDYDDKNFI

>ATMYB24(AT5G40350)

MEKRESSGGSGSGDAEVRKGPWTMEEDLILINYIANHGEGVWNSLAKSAGLKRTGKSCRLRWLNYLRPDVRRGNITPEEQLTIMELHAKWGNRWSKIAKHLPGRTDNEIKNFWRTKIQKYIIKSGETTTVGSQSSEFINHHATTSHVMNDTQETMDMYSPTTSYQHASNINQQLNYGNYVPESGSIMMPLSVDQSEQNYWSVDDLWPMNIYNGN

>ATMYB70(AT2G23290)

MSGSTRKEMDRIKGPWSPEEDDLLQSLVQKHGPRNWSLISKSIPGRSGKSCRLRWCNQLSPEVEHRGFTAEEDDTIILAHARFGNKWATIARLLNGRTDNAIKNHWNSTLKRKCSGGGGGGEEGQSCDFGGNGGYDGNLTDEKPLKRRASGGGGVVVVTALSPTGSDVSEQSQSSGSVLPVSSSCHVFKPTARAGGVVIESSSPEEEEKDPMTCLRLSLPWVNESTTPPELFPVKREEEEEKEREISGLGGDFMTVVQEMIKTEVRSYMADLQLGNGGGAGGGASSCMVQGTNGRNVGFREFIGLGRIE

>ATMYB71(AT3G24310)

MSLWGGMGGGWGMVEEGWRKGPWTAEEDRLLIDYVQLHGEGRWNSVARLAGLKRNGKSCRLRWVNYLRPDLKRGQITPHEETIILELHAKWGNRWSTIARSLPGRTDNEIKNYWRTHFKKKTKSPTNSAEKTKNRILKRQQFQQQRQMELQQEQQLLQFNQIDMKKIMSLLDDDNNNGDNTFSSSSSGESGALYVPHQITHSTTTSGCEPNSNGYYPVVPVTIPEANVNEDNAIWDGLWNLDFEGQGSFGGAACAPRKHYFQNMVIPFC

>ATMYB47(AT1G18710)

MGRTTWFDVDGMKKGEWTAEEDQKLGAYINEHGVCDWRSLPKRAGLQRCGKSCRLRWLNYLKPGIRRGKFTPQEEEEIIQLHAVLGNRWAAMAKKMQNRTDNDIKNHWNSCLKKRLSRKGIDPMTHEPIIKHLTVNTTNADCGNSSTTTSPSTTESSPSSGSSRLLNKLAAGISSRQHSLDRIKYILSNSIIESSDQAKEEEEKEEEEEERDSMMGQKIDGSEGEDIQIWGEEEVRRLMEIDAMDMYEMTSYDAVMYESSHILDHLF

>ATMYB122(AT1G74080)

MVRTPCCRAEGLKKGAWTQEEDQKLIAYVQRHGEGGWRTLPDKAGLKRCGKSCRLRWANYLRPDIKRGEFSQDEEDSIINLHAIHGNKWSAIARKIPRRTDNEIKNHWNTHIKKCLVKKGIDPLTHKSLLDGAGKSSDHSAHPEKSSVHDDKDDQNSNNKKLSGSSSARFLNRVANRFGHRINHNVLSDIIGSNGLLTSHTTPTTSVSEGERSTSSSSTHTSSNLPINRSITVDATSLSSSTFSDSPDPCLYEEIVGDIEDMTRFSSRCLSHVLSHEDLLMSVESCLENTSFMREITMIFQEDKIETTSFNDSYVTPINEVDDSCEGIDNYFG

>ATMYB97(AT4G26930)

MIVYGGGASEDGEGGGVVLKKGPWTVAEDETLAAYVREYGEGNWNSVQKKTWLARCGKSCRLRWANHLRPNLRKGSFTPEEERLIIQLHSQLGNKWARMAAQLPGRTDNEIKNYWNTRLKRFQRQGLPLYPPEYSQNNHQQQMYPQQPSSPLPSQTPASSFTFPLLQPPSLCPKRCYNTAFSPKASYISSPTNFLVSSPTFLHTHSSLSSYQSTNPVYSMKHELSSNQIPYSASLGVYQVSKFSDNGDCNQNLNTGLHTNTCQLLEDLMEEAEALADSFRAPKRRQIMAALEDNNNNNNFFSGGFGHRVSSNSLCSLQGLTPKEDESLQMNTMQDEDITKLLDWGSESEEISNGQSSVITTENNLVLDDHQFAFLFPVDDDTNNLPGIC

>ATMYB117(AT1G26780)

MFITEKQVWMDEIVARRASSSWDFPFNDINIHQHHHRHCNTSHEFEILKSPLGDVAVHEEESNNNNPNFSNSESGKKETTDSGQSWSSSSSKPSVLGRGHWRPAEDVKLKELVSIYGPQNWNLIAEKLQGRSGKSCRLRWFNQLDPRINRRAFTEEEEERLMQAHRLYGNKWAMIARLFPGRTDNSVKNHWHVVMARKYREHSSAYRRRKLMSNNPLKPHLTNNHHPNPNPNYHSFISTNHYFAQPFPEFNLTHHLVNNAPITSDHNQLVLPFHCFQGYENNEPPMVVSMFGNQMMVGDNVGATSDALCNIPHIDPSNQEKPEPNDAMHWIGMDAVDEEVFEKAKQQPHFFDFLGLGTA

>ATMYB64(AT5G10280)

MGRSPISDDSGLKKGPWTPDEDEKLVNYVQKHGHSSWRALPKLAGLNRCGKSCRLRWTNYLRPDIKRGRFSPDEEQTILNLHSVLGNKWSTIANQLPGRTDNEIKNFWNTHLKKKLIQMGFDPMTHRPRTDIFSGLSQLMSLSSNLRGFVDLQQQFPIDQEHTILKLQTEMAKLQLFQYLLQPSSMSNNVNPNDFDTLSLLNSIASFKETSNNTTSNNLDLGFLGSYLQDFHSLPSLKTLNSNMEPSSVFPQNLDDNHFKFSTQRENLPVSPIWLSDPSSTTPAHVNDDLIFNQYGIEDVNSNITSSSGQESGASASAAWPDHLLDDSIFSDIP

>ATMYB121(AT3G30210)

MLDWGVQGHHQKHDHDIYQQQHQQQGCRKGPWTLEEDKLLAEYVTSHGEGRWSTVAKCAGLNRSGKSCRLRWVNYLRPGLKRGQITPQEEGIILELHSLWGNKWSTIARYLPGRTDNEIKNYWRTHYKKNQKSSSKQDKVKKSLSRKQQQVDLKPQPQAQSENHQSQLVSQDHMNIDNDHNIASSLYYPTSVFDDKLYMPQSVATTSSDHSMIDEGHLWGSLWNLDEDDPHSFGGGSGQGTAADIDEKFPDSGIEAPSCGSGDYSYTGVYMGGYIF

>ATMYB28(AT5G61420)

MSRKPCCVGEGLKKGAWTTEEDKKLISYIHDHGEGGWRDIPQKAGLKRCGKSCRLRWTNYLKPEIKRGEFSSEEEQIIIMLHASRGNKWSVIARHLPRRTDNEIKNYWNTHLKKRLMEQGIDPVTHKPLASSSNPTVDENLNSPNASSSDKQYSRSSSMPFLSRPPPSSCNMVSKVSELSSNDGTPIQGSSLSCKKRFKKSSSTSRLLNKVAAKATSIKDILSASMEGSLSATTISHASFFNGFTEQIRNEEDSSNTSLTNTLAEFDPFSPSSLYPEHEINATSDLNMDQDYDFSQFFEKFGGDNHNEENSMNDLLMSDVSQEVSSTSVDDQDNMVGNFEGWSNYLLDHTNFMYDTDSDSLEKHFI

>ATMYB89(AT5G39700)

MYLFMYKCNIVLEETHVFQNTPCDVSLQRPFNGFGENNALPLRKMHQEEKKKKHRGGHWTLSEDLKLKELVAVFGPQNWKFIGEKMEPRTSLSCRQRWFNQLDPKINKRNFTDEEEEKLLRAHILYGNKWSKIAKLFNRRTDHAVKNHWHSLMNRIIRKQSASDIRSFDNIQNYQTSNFLPGLCLLNTKQ

>ATMYB83(AT3G08500)

MMMRKPDITTIRDKGKPNHACGGNNNKPKLRKGLWSPDEDEKLIRYMLTNGQGCWSDIARNAGLLRCGKSCRLRWINYLRPDLKRGSFSPQEEDLIFHLHSILGNRWSQIATRLPGRTDNEIKNFWNSTLKKRLKNNSNNNTSSGSSPNNSNSNSLDPRDQHVDMGGNSTSLMDDYHHDENMMTVGNTMRMDSSSPFNVGPMVNSVGLNQLYDPLMISVPDNGYHQMGNTVNVFSVNGLGDYGNTILDPISKRVSVEGDDWFIPPSENTNVIACSTSNNLNLQALDPCFNSKNLCHSESFKVGNVLGIENGSWEIENPKIGDWDLDGLIDNNSSFPFLDFQVD

>ATMYB36(AT5G57620)

MGRAPCCDKANVKKGPWSPEEDVKLKDYIDKYGTGGNWIALPQKIGLKRCGKSCRLRWLNYLRPNIKHGGFSEEEDRIILSLYISIGSRWSIIAAQLPGRTDNDIKNYWNTKLKKKLLGRQKQMNRQDSITDSTENNLSNNNNNKSPQNLSNSALERLQLHMQLQNLQSPFSSFYNNPILWPKLHPLLQSTTTNQNPKLASQESFHPLGVNVDHQHNNTKLAQINNGASSLYSENVEQSQNPAHEFQPNFGFSQDLRLDNHNMDFMNRGVSKELFQVGNEFELTNGSSWWSEEVELERKTTSSSSWGSASVLDQTTEGMVMLQDYAQMSYHSV

>ATMYB64(AT5G11050)

MEEQKIQEKSLAHGAAPPLTAVERFLNGQKNEALCFKKQERSIDRPIVKTTRAIEIRNENKENMMFGPRKEKNLAVIGEIVVKGAAKDYTCKDITKKQPYKNIIKGQWTADEDRKLIKLVMQHGERKWAVISEKLEGRAGKQCRERWHNHLRPDIKKDSWSEEEERLLVEAHTRIGNKWAEIAKLIQGRTENSIKNHWNATKRRQNSKRKHKRSKNADSNSDIDDLSPSAKRPRILEDYIKNIENNDKNNGENIMTTSGNNVLSTSNYDQFNSEDSTSSLLDDPYDEELVFLKNIFENHSLENINLSQGTEITQSSSSGFMIENPKPKPNLYNNTFGTHLGAMVTEPANSSHLASDIYLSDLLNGTASSSSSLTFLSSNNNEHAGENELLLPQANSTSERREMDLIEMLSGSTQGSNIWFPLF

>ATMYB18(AT4G25560)

MAKTKYGERHRKGLWSPEEDEKLRSFILSYGHSCWTTVPIKAGLQRNGKSCRLRWINYLRPGLKRDMISAEEEETILTFHSSLGNKWSQIAKFLPGRTDNEIKNYWHSHLKKKWLKSQSLQDAKSISPPSSSSSSLVACGKRNPETLISNHVFSFQRLLENKSSSPSQESNGNNSHQCSSAPEIPRLFFSEWLSSSYPHTDYSSEFTDSKHSQAPNVEETLSAYEEMGDVDQFHYNEMMINNSNWTLNDIVFGSKCKKQEHHIYREASDCNSSAEFFSPSTTT

>ATMYB22(AT5G40430)

MGEPQLFDVPVLEGIKNTTNEIMNQLEDDKMKKTYENKKEASTSKYLKKSDITKKRWTESEDIKLKEMVALEPKKWTKVAKHFEGRTPKQCRERWHNHARPNVKKTTWSEEEDQILIEVHKVIGAKWIQISEQLPGRSYNNVKNHWNTTKRRVQNKSGRTVNRVGNNILENYIRSITINNDDESDGEPTNIENYHDDSEDMLYGEMNLSPEAITQTTKPLTDASTISPYIPMPKENYTLEVCESLEDYLELLRWWD

>ATMYB123/ATTT2(AT5G35550)

MKTRSLEITSPLTAKANGALSLTKLVLISLSTDPSLYLLRTIIVLAHIKGLKRCGKSCRLRWKNYLRPGIKRGNISSDEEELIIRLHNLLGNRWSLIAGRLPGRTDNEIKNHWNSNLRKRLPKTQTKQPKRIKHSTNNENNVCVIRTKAIRCSKTLLFSDLSLQKKSSTSPLPLKEQEMDQGGSSLMGDLEFDFDRIHSEFHFPDLMDFDGLDCGNVTSLVSSNEILGELVPAQGNLDLNRPFTSCHHRGDDEDWLRDFTC

>ATMYB74(AT4G05100)

MGRSPCCEKKNGLKKGPWTPEEDQKLIDYINIHGYGNWRTLPKNAGLQRCGKSCRLRWTNYLRPDIKRGRFSFEEEETIIQLHSIMGNKWSAIAARLPGRTDNEIKNYWNTHIRKRLLKMGIDPVTHTPRLDLLDISSILSSSIYNSSHHHHHHHQQHMNMSRLMMSDGNHQPLVNPEILKLATSLFSNQNHPNNTHENNTVNQTEVNQYQTGYNMPGNEELQSWFPIMDQFTNFQDLMPMKTTVQNSLSYDDDCSKSNFVLEPYYSDFASVLTTPSSSPTPLNSSSSTYINSSTCSTEDEKESYYSDNITNYSFDVNGFLQFQ

>ATMYB93(AT1G34670)

MGRSPCCDENGLKKGPWTPEEDQKLIDYIHKHGHGSWRALPKLADLNRCGKSCRLRWTNYLRPDIKRGKFSAEEEQTILHLHSILGNKWSAIATHLQGRTDNEIKNFWNTHLKKKLIQMGIDPVTHQPRTDLFASLPQLIALANLKDLIEQTSQFSSMQGEAAQLANLQYLQRMFNSSASLTNNNGNNFSPSSILDIDQHHAMNLLNSMVSWNKDQNPAFDPVLELEANDQNQDLFPLGFIIDQPTQPLQQQKYHLNNSPSELPSQGDPLLDHVPFSLQTPLNSEDHFIDNLVKHPTDHEHEHDDNPSSWVLPSLIDNNPKTVTSSLPHNNPADASSSSSYGGCEAASFYWPDICFDESLMNVIS

>ATMYB3R-1(AT4G32730)

MKREMKAPTTPLESLQGDLKGKQGRTSGPARRSTKGQWTPEEDEVLCKAVERFQGKNWKKIAECFKDRTDVQCLHRWQKVLNPELVKGPWSKEEDNTIIDLVEKYGPKKWSTISQHLPGRIGKQCRERWHNHLNPGINKNAWTQEEELTLIRAHQIYGNKWAELMKFLPGRSDNSIKNHWNSSVKKKLDSYYASGLLDQCQSSPLIALQNKSIASSSSWMHSNGDEGSSRPGVDAEESECSQASTVFSQSTNDLQDEVQRGNEEYYMPEFHSGTEQQISNAASHAEPYYPSFKDVKIVVPEISCETECSKKFQNLNCSHELRTTTATEDQLPGVSNDAKQDRGLELLTHNMDNGGKNQALQQDFQSSVRLSDQPFLSNSDTDPEAQTLITDEECCRVLFPDNMKDSSTSSGEQGRNMVDPQNGKGSLCSQAAETHAHETGKVPALPWHPSSSEGLAGHNCVPLLDSDLKDSLLPRNDSNAPIQGCRLFGATELECKTDTNDGFIDTYGHVTSHGNDDNGGFPEQQGLSYIPKDSLKLVPLNSFSSPSRVNKIYFPIDDKPAEKDKGALCYEPPRFPSADIPFFSCDLVPSNSDLRQEYSPFGIRQLMISSMNCTTPLRLWDSPCHDRSPDVMLNDTAKSFSGAPSILKKRHRDLLSPVLDRRKDKKLKRAATSSLANDFSRLDVMLDEGDDCMTSRPSESPEDKNICASPSIARDNRNCASARLYQEMIPIDEEPKETLESGGVTSMQNENGCNDGGASAKNDQETSGSFFELRLCSPGMTRARPDNKVNASAKDLSNQHKISLGDFPTEEMSSEPLCTVDSIPLSAIDKTNTAETSFDIENFNIFDGTPFRKLLDTPSPWKSPLLFGSFLQSPKLPPEITFEDIGCFMSPGERSYDAIGLMKHLSEHSATAYADALEVLGNDTPESILKKRQLNKSIQGKENQHQPHDQLGNRSQVECRALDFSDCGTPGKAKVPSASPGGYSSPSSYLLKSCR

>ATMYB4R1(AT3G18100)

MNRNSLYEADDDDDDDEEDDIGEDLEDLRRACMVSDVNSDQFASKTGSIEPEGVGGGEIPSDSENEDDFEMLRTIKSQLASSKDAGRSSGPPMGLSLLSDSESEDDFEMIRSIKSQLSLSMDVSLPPIGLSDDEEDDAFETLRAIRRRFSAYKNFDSEGKFMNDSHGKKKQVHNSDNEPSSEILSRSNTCESFPDHGKSVVTVPDSEDVQDGHMPAASSSFPEAARAFVDAIRRNRAYQKFLRGKLAEIEATIEQNEKHKKNVRIVKDFQASCKRITKLALCQRKDPRVELISTRKSGPCDSSEVIGPCDSFEGNDKKISPLTLGPAENPCVENYRMALEKYPISVKRRKWSTEENKNLAKGLKQEVQKILLSEAIERSSDLEGATYDIDTINESIGNLEITPEMIRQFLPKINWDSLDIKDRSAAECEARWMSSEDPLINHGPWTAAEDKNLLRTIEQTSLTDWVDIAVSLGTNRTPFQCLARYQRSLNPSILKKEWTAEEDDQLRTAVELFGEKDWQSVANVLKGRTGTQCSNRWKKSLRPTRKGTWSLEEDKRVKVAVTLFGSQNWHKISQFVPGRTQTQCRERWLNCLDPKVNRGKWTEEEDEKLREAIAEHGYSWSKVATNLSCRTDNQCLRRWKRLYPHQVALLQEARRLQKEASVGNFVDRESERPALVTSPILALPDISLEPEPDSVALKKKRKAKQKKSDAERQPKRRRKGLKNCSGDVCRQENETVCENEPNNGGEERMLALECHDEIQDNAKEKPKQRRKSVAETVCENEPNNGGEERMLALECDNEIQDNAKEKRKRRRKSVAETSNNTTGLKKLTPRRRKISAVVPIKNQDAPN

>ATMYB44(AT5G67300)

MADRIKGPWSPEEDEQLRRLVVKYGPRNWTVISKSIPGRSGKSCRLRWCNQLSPQVEHRPFSAEEDETIARAHAQFGNKWATIARLLNGRTDNAVKNHWNSTLKRKCGGYDHRGYDGSEDHRPVKRSVSAGSPPVVTGLYMSPGSPTGSDVSDSSTIPILPSVELFKPVPRPGAVVLPLPIETSSSSDDPPTSLSLSLPGADVSEESNRSHESTNINNTTSSRHNHNNTVSFMPFSGGFRGAIEEMGKSFPGNGGEFMAVVQEMIKAEVRSYMTEMQRNNGGGFVGGFIDNGMIPMSQIGVGRIE

>ATMYB34(AT5G60890)

MVRTPCCKEEGIKKGAWTPEEDQKLIAYLHLHGEGGWRTLPEKAGLKRCGKSCRLRWANYLRPDIKRGEFSPEEDDTIIKLHALKGNKWAAIATSLAGRTDNEIKNYWNTNLKKRLKQKGIDAITHKPINSTGQTGFEPKVNKPVYSSGSARLLNRVASKYAVELNRDLLTGIISGNSTVAEDSQNSGDVDSPTSTLLNKMAATSVLINTTTTYSGFSDNCSFTDEFNEFFNNEEISDIYTTVDNFGFMEELKSILSYGDASAGVIENSPEVNVADAMEFIDSWNEDDNMVGVFV

>ATMYB81(AT2G26960)

MGKVRQDSGSDDDNSIKKSFTKGPWTQAEDNLLIAYVDKHGDGNWNAVQNNSGLSRCGKSCRLRWVNHLRPDLKKGAFTEKEEKRVIELHALLGNKWARMAEELPGRTDNEIKNFWNTRLKRLQRLGLPVYPDEVREHAMNAATHSGLNTDSLDGHHSQEYMEADTVEIPEVDFEHLPLNRSSSYYQSMLRHVPPTNVFVRQKPCFFQPPNVYNLIPPSPYMSTGKRPREPETAFPCPGGYTMNEQSPRLWNYPFVENVSEQLPDSHLLGNAAYSSPPGPLVHGVENFEFPSFQYHEEPGGWGADQPNPMPEHESDNTLVQSPLTAQTPSDCPSSSLYDGLLESVVYGSSGEKPATDTDSESSLFQSFTPANENITGKTCFLTLYALHALHCLCNQFKKSPLLHLHDKLNWCNKFRFNSFKSGTHIL

>ATMYB31(AT1G74650)

MGRPPCCEKIEVKKGPWTPEEDIILVSYIQQHGPGNWRSVPANTGLLRCSKSCRLRWTNYLRPGIKRGNFTQPEEKMIIHLQALLGNRWAAIASYLPQRTDNDIKNYWNTHLKKKLVMMKFQNGIINENKTNLATDISSCNNNNNGCNHNKRTTNKGQWEKKLQTDINMAKQALFQALSLDQPSSLIPPDPDSPKPHHHSTTTYASSTDNISKLLQNWTSSSSSKPNTSSVSNNRSSSPGEGGLFDHHSLFSSNSESGSVDEKLNLMSETSMFKGESKPDIDMEATPTTTTTDDQGSLSLIEKWLFDDQGLVQCDDSQEDLIDVSLEELK

>ATMYB31(AT1G69560)

MMFKVIFNICSSPPMRMEMVHADVASLSITPCFPSSLSSSSHHHYNQQQHCIMSEDQHHSMDQTTSSDYFSLNIDNAQHLRSYYTSHREEDMNPNLSDYSNCNKKDTTVYRSCGHSSKASVSRGHWRPAEDTKLKELVAVYGPQNWNLIAEKLQGRSGKSCRLRWFNQLDPRINRRAFTEEEEERLMQAHRLYGNKWAMIARLFPGRTDNSVKNHWHVIMARKFREQSSSYRRRKTMVSLKPLINPNPHIFNDFDPTRLALTHLASSDHKQLMLPVPCFPGYDHENESPLMVDMFETQMMVGDYIAWTQEATTFDFLNQTGKSEIFERINEEKKPPFFDFLGLGTV

>ANTHOCYANINLESS2(AT4G17780)

MEEEEKNPSSIYIVADLLEDIFLRLPLKSILISKSVSKRWRSILESKTFVERRMSLQKKRKILAAYNCKCGWEPRLLPGSSQCKGNEEIVYLHCNAAQPSFTCDGLVCILEPRWIDVLNPWTRQLRRYGFGFGTIFGVGSAFSPRHWAMGFGKDKVTGSYKVVKMCLISFSEICARDPEVEYSVLDVETGEWRMLSPPPYKVFEVRKSECANGSIYWLHKPTERAWTILALDLHKEELHNISVPDMSVTQETFQIVNLEDRLAIANTYTKTEWKLEIWSMDTEVETWTKTYSIDLENRVASRERRNRWFTPVSVSKQGNIVFYDNHKRLFKYYPRKNEILYLSADTCVISPFFENLAPLPQKSTLHTPIIAGEPNAPLTTLVVVGSIWSPLFLFSVKL

>ATMYB20(AT1G66230)

MGRQPCCDKVGLKKGPWTAEEDRKLINFILTNGQCCWRAVPKLSGLLRCGKSCRLRWTNYLRPDLKRGLLSDYEEKMVIDLHSQLGNRWSKIASHLPGRTDNEIKNHWNTHIKKKLRKMGIDPLTHKPLSIVEKEDEEPLKKLQNNTVPFQETMERPLENNIKNISRLEESLGDDQFMEINLEYGVEDVPLIETESLDLICSNSTMSSSTSTSSHSSNDSSFLKDLQFPEFEWSDYGNSNNDNNNGVDNIIENNMMSLWEISDFSSLDLLLNDESSSTFGLF

>ATMYB87(AT4G37780)

MGRAPCCDKMAVKKGPWSTEEDAVLKSYIEKHGTGNNWISLPQRIGIKRCGKSCRLRWLNYLRPNLKHGGFTDEEDYIICSLYITIGSRWSIIASQLPGRTDNDIKNYWNTRLKKKLLSKQGKAFHQQLNVKFERGTTSSSSSQNQIQIFHDENTKSNQTLYNQVVDPSMRAFAMEEQSMIKNQILEPFSWEPNKVLFDVDYDAAASSYHHHASPSLNSMSSTSSIGTNNSSLQMSHYTVNHNDHDQPDMFFMDGFENFQAELFDEIANNNTVENGFDGTEILINNNYLDHDISSFIDYPLYDNE

>ATMYB77(AT3G50060)

MADRVKGPWSQEEDEQLRRMVEKYGPRNWSAISKSIPGRSGKSCRLRWCNQLSPEVEHRPFSPEEDETIVTARAQFGNKWATIARLLNGRTDNAVKNHWNSTLKRKCSGGVAVTTVTETEEDQDRPKKRRSVSFDSAFAPVDTGLYMSPESPNGIDVSDSSTIPSPSSPVAQLFKPMPISGGFTVVPQPLPVEMSSSSEDPPTSLSLSLPGAENTSSSHNNNNNALMFPRFESQMKINVEERGEGRRGEFMTVVQEMIKAEVRSYMAEMQKTSGGFVVGGLYESGGNGGFRDCGVITPKVE

>ATMYB27(AT3G53200)

MDFKKEETLRRGPWLEEEDERLVKVISLLGERRWDSLAIVSGLKRSGKSCRLRWMNYLNPTLKRGPMSQEEERIIFQLHALWGNKWSKIARRLPGRTDNEIKNYWRTHYRKKQEAQNYGKLFEWRGNTGEELLHKYKETEITRTKTTSQEHGFVEVVSMESGKEANGGVGGRESFGVMKSPYENRISDWISEISTDQSEANLSEDHSSNSCSENNINIGTWWFQETRDFEEFSCSLWS

>ATMYB54(AT1G73410)

MIMCSRGHWRPAEDEKLKDLVEQYGPHNWNAIALKLPGRSGKSCRLRWFNQLDPRINRNPFTEEEEERLLAAHRIHGNRWSIIARLFPGRTDNAVKNHWHVIMARRTRQTSKPRLLPSTTSSSSLMASEQIMMSSGGYNHNYSSDDRKKIFPADFINFPYKFSHINHLHFLKEFFTGKIALNHKANQSKKPMEFYNFLQVNTDSNKSEIIDQDSGQSKRSDSDTKHESHVPFFDFLSVGNSAS

>ATMYB3R3(AT3G09370)

MIAFLGFLCFLSKSGSFFSFIEMMDLQEETGEVKIEDQCVENKQSTPASCSSVSEGSAGSSHKSPTIASPATVSPTHRYLGRTSGPIRRAKGGWTPEEDETLRQAVDTFKGKSWKNIAKSFPDRTEVQCLHRWQKVLNPDLIKGPWTHEEDEKIVELVEKYGPAKWSIIAQSLPGRIGKQCRERWHNHLNPDINKDAWTTEEEVALMNAHRSHGNKWAEIAKVLPGRTDNAIKNHWNSSLKKKSEFYLLTGRLPPPTTTRNGVPDSVTKRSSSAQKRVFGSVAQTSSVTTDVNNLAEDGNGQINSSVPVEEVVAASRMTSLNEYARSPQLPNPEPLPENGGAANNGYHLYYTPQIDYYRASEVDTQRMYGNECGCSPSASPVSFFTPPPCRNVHSNGSTPRSPESYLREAGRTYPNTPSIFRKRRPRVVVQDNNNAKKTDEAKEVDQKVNDGKDSSEIQNNGSNAYNLSPPYRIRSKRTAVFKSRQLEFISREEEKADDETKSSEKDMLIDGDSQLLG

>ATMYB37(AT5G23000)

MGRAPCCDKTKVKRGPWSPEEDSKLRDYIEKYGNGGNWISFPLKAGLRRCGKSCRLRWLNYLRPNIKHGDFSEEEDRIIFSLFAAIGSRWSIIAAHLPGRTDNDIKNYWNTKLRKKLLSSSSDSSSSAMASPYLNPISQDVKRPTSPTTIPSSSYNPYAENPNQYPTKSLISSINGFEAGDKQIISYINPNYPQDLYLSDSNNNTSNANGFLLNHNMCDQYKNHTSFSSDVNGIRSEIMMKQEEIMMMMMIDHHIDQRTKGYNGEFTQGYYNYYNGHGDLKQMISGTGTNSNINMGGSGSSSSSISNLAENKSSGSLLLEYKCLPYFYS

>ATMYB3R4(AT5G11510)

MEAESSTPQERIPKLRHGRTSGPARRSTRGQWTAEEDEILRKAVHSFKGKNWKKIAEYFKDRTDVQCLHRWQKVLNPELVKGPWTKEEDEMIVQLIEKYGPKKWSTIARFLPGRIGKQCRERWHNHLNPAINKEAWTQEEELLLIRAHQIYGNRWAELTKFLPGRSDNGIKNHWHSSVKKKLDSYMSSGLLDQYQAMPLAPYERSSTLQSTFMQSNIDGNGCLNGQAENEIDSRQNSSMVGCSLSARDFQNGTINIGHDFHPCGNSQENEQTAYHSEQFYYPELEDISVSISEVSYDMEDCSQFPDHNVSTSPSQDYQFDFQELSDISLEMRHNMSEIPMPYTKESKESTLGAPNSTLNIDVATYTNSANVLTPETECCRVLFPDQESEGHSVSRSLTQEPNEFNQVDRRDPILYSSASDRQISEATKSPTQSSSSRFTATAASGKGTLRPAPLIISPDKYSKKSSGLICHPFEVEPKCTTNGNGSFICIGDPSSSTCVDEGTNNSSEEDQSYHVNDPKKLVPVNDFASLAEDRPHSLPKHEPNMTNEQHHEDMGASSSLGFPSFDLPVFNCDLLQSKNDPLHDYSPLGIRKLLMSTMTCMSPLRLWESPTGKKTLVGAQSILRKRTRDLLTPLSEKRSDKKLEIDIAASLAKDFSRLDVMFDETENRQSNFGNSTGVIHGDRENHFHILNGDGEEWSGKPSSLFSHRMPEETMHIRKSLEKVDQICMEANVREKDDSEQDVENVEFFSGILSEHNTGKPVLSTPGQSVTKAEKAQVSTPRNQLQRTLMATSNKEHHSPSSVCLVINSPSRARNKEGHLVDNGTSNENFSIFCGTPFRRGLESPSAWKSPFYINSLLPSPRFDTDLTIEDMGYIFSPGERSYESIGVMTQINEHTSAFAAFADAMEVSISPTNDDARQKKELDKENNDPLLAERRVLDFNDCESPIKATEEVSSYLLKGCR

>ATMYB23(AT5G40330)

MRMTRDGKEHEYKKGLWTVEEDKILMDYVRTHGQGHWNRIAKKTGLKRCGKSCRLRWMNYLSPNVNRGNFTDQEEDLIIRLHKLLGNRWSLIAKRVPGRTDNQVKNYWNTHLSKKLGLGDHSTAVKAACGVESPPSMALITTTSSSHQEISGGKNSTLRFDTLVDESKLKPKSKLVHATPTDVEVAATVPNLFDTFWVLEDDFELSSLTMMDFTNGYCL

>ATMYB91(AT2G37630)

MKERQRWSGEEDALLRAYVRQFGPREWHLVSERMNKPLNRDAKSCLERWKNYLKPGIKKGSLTEEEQRLVIRLQEKHGNKWKKIAAEVPGRTAKRLGKWWEVFKEKQQREEKESNKRVEPIDESKYDRILESFAEKLVKERSNVVPAAAAAATVVMANSNGGFLHSEQQVQPPNPVIPPWLATSNNGNNVVARPPSVTLTLSPSTVAAAAPQPPIPWLQQQQPERAENGPGGLVLGSMMPSCSGSSESVFLSELVECCRELEEGHRAWADHKKEAAWRLRRLELQLESEKTCRQREKMEEIEAKMKALREEQKNAMEKIEGEYREQLVGLRRDAEAKDQKLADQWTSRHIRLTKFLEQQMGCRLDRP

>ATMYB99(AT5G62320)

MGGRKPCCDEVGLRKGPWTVEEDGKLVDFLRARGNCGGGGGGWCWRDVPKLAGLRRCGKSCRLRWTNYLRPDLKRGLFTEEEIQLVIDLHARLGNRWSKIAVELPGRTDNDIKNYWNTHIKRKLIRMGIDPNTHRRFDQQKVNEEETILVNDPKPLSETEVSVALKNDTSAVLSGNLNQLADVDGDDQPWSFLMENDEGGGGDAAGELTMLLSGDITSSCSSSSSLWMKYGEFGYEDLELGCFDV

>ATMYB68(AT5G65790)

MGRAPCCDKANVKKGPWSPEEDAKLKDYIENSGTGGNWIALPQKIGLRRCGKSCRLRWLNYLRPNIKHGGFSEEEDNIICNLYVTIGSRWSIIAAQLPGRTDNDIKNYWNTRLKKKLLNKQRKEFQEARMKQEMVMMKRQQQGQGQGQSNGSTDLYLNNMFGSSPWPLLPQLPPPHHQIPLGMMEPTSCNYYQTTPSCNLEQKPLITLKNMVKIEEEQERTNPDHHHQDSVTNPFDFSFSQLLLDPNYYLGSGGGGEGDFAIMSSSTNSPLPNTSSDQHPSQQQEILQWFGSSNFQTEAINDMFINNNNNIVNLETIENTKVYGDASVAGAAVRAALGGGTTSTSADQSTISWEDITSLVNSEDASYFNAPNHV

>ATMYB57(AT3G01530)

METTMKKKGRVKATITSQKEEEGTVRKGPWTMEEDFILFNYILNHGEGLWNSVAKASGLKRTGKSCRLRWLNYLRPDVRRGNITEEEQLLIIQLHAKLGNRWSKIAKHLPGRTDNEIKNFWRTKIQRHMKVSSENMMNHQHHCSGNSQSSGMTTQGSSGKAIDTAESFSQAKTTTFNVVEQQSNENYWNVEDLWPVHLLNGDHHVI

>ATMYB5(AT3G13540)

MMSCGGKKPVSKKTTPCCTKMGMKRGPWTVEEDEILVSFIKKEGEGRWRSLPKRAGLLRCGKSCRLRWMNYLRPSVKRGGITSDEEDLILRLHRLLGNRWSLIAGRIPGRTDNEIKNYWNTHLRKKLLRQGIDPQTHKPLDANNIHKPEEEVSGGQKYPLEPISSSHTDDTTVNGGDGDSKNSINVFGGEHGYEDFGFCYDDKFSSFLNSLINDVGDPFGNIIPISQPLQMDDCKDGIVGASSSSLGHD

>ATMYB102(AT4G21440)

MARSPCCEKNGLKKGPWTSEEDQKLVDYIQKHGYGNWRTLPKNAGLQRCGKSCRLRWTNYLRPDIKRGRFSFEEEETIIQLHSFLGNKWSAIAARLPGRTDNEIKNFWNTHIRKKLLRMGIDPVTHSPRLDLLDISSILASSLYNSSSHHMNMSRLMMDTNRRHHQQHPLVNPEILKLATSLFSQNQNQNLVVDHDSRTQEKQTVYSQTGVNQYQTNQYFENTITQELQSSMPPFPNEARQFNNMDHHFNGFGEQNLVSTSTTSVQDCYNPSFNDYSSSNFVLDPSYSDQSFNFANSVLNTPSSSPSPTTLNSSYINSSSCSTEDEIESYCSNLMKFDIPDFLDVNGFII

>ATMYB124(AT1G14350)

MEDTKKKKKKNINNNQDSKKKERHIVTWSQEEDVILREQITLHGTENWAIIASKFKDKSTRQCRRRWYTYLNSDFKRGGWSPEEDMLLCEAQRVFGNRWTEIAKVVSGRTDNAVKNRFTTLCKKRAKHEAMTKDSNSNTKRMLFLDGISTPRKSENETPIAKKLKRSHILDLTEISNYGRAEACVNQQIRSPFSVLARNATGIDSLEEQNQTSNVNESDGEGMFLKKDDPKVTALMQQAELLSSLAQKVNADNTEQSMENAWKVLQDFLNKGKENDLFRYGIPDIDFKIEEFKDLIEDLRSGYEDNQLSWRQPDLHDSPASSEYSSGSTIMVDQSGDKTQPFSADTQTEHKQVGEELLVPKNPDENMPISGEEKFSSPIQVTPLFRSLADGIPSPQFSESERSFLLKTLGIESSSPCPSANPSKPPPCKRVLLHSL

>ATMYB33(AT5G06100)

MSYTSTDSDHNESPAADDNGSDCRSRWDGHALKKGPWSSAEDDILIDYVNKHGEGNWNAVQKHTSLFRCGKSCRLRWANHLRPNLKKGAFSQEEEQLIVELHAKMGNRWARMAAHLPGRTDNEIKNYWNTRIKRRQRAGLPLYPPEMHVEALEWSQEYAKSRVMGEDRRHQDFLQLGSCESNVFFDTLNFTDMVPGTFDLADMTAYKNMGNCASSPRYENFMTPTIPSSKRLWESELLYPGCSSTIKQEFSSPEQFRNTSPQTISKTCSFSVPCDVEHPLYGNRHSPVMIPDSHTPTDGIVPYSKPLYGAVKLELPSFQYSETTFDQWKKSSSPPHSDLLDPFDTYIQSPPPPTGGEESDLYSNFDTGLLDMLLLEAKIRNNSTKNNLYRSCASTIPSADLGQVTVSQTKSEEFDNSLKSFLVHSEMSTQNADETPPRQREKKRKPLLDITRPDVLLASSWLDHGLGIVKETGSMSDALAVLLGDDIGNDYMNMSVGASSGVGSCSWSNMPPVCQMTELP

>ATMYB32(AT4G34990)

MGRSPCCEKDHTNKGAWTKEEDDKLISYIKAHGEGCWRSLPRSAGLQRCGKSCRLRWINYLRPDLKRGNFTLEEDDLIIKLHSLLGNKWSLIATRLPGRTDNEIKNYWNTHVKRKLLRKGIDPATHRPINETKTSQDSSDSSKTEDPLVKILSFGPQLEKIANFGDERIQKRVEYSVVEERCLDLNLELRISPPWQDKLHDERNLRFGRVKYRCSACRFGFGNGKECSCNNVKCQTEDSSSSSYSSTDISSSIGYDFLGLNNTRVLDFSTLEMK

>ATMYB61(AT1G09540)

MGRHSCCYKQKLRKGLWSPEEDEKLLTHITNHGHGCWSSVPKLAGLQRCGKSCRLRWINYLRPDLKRGAFSPEEENLIVELHAVLGNRWSQIASRLPGRTDNEIKNLWNSSIKKKLKQRGIDPNTHKPISEVESFSDKDKPTTSNNKRSGNDHKSPSSSSATNQDFFLERPSDLSDYFGFQKLNFNSNLGLSVTTDSSLCSMIPPQFSPGNMVGSVLQTPVCVKPSISLPPDNNSSSPISGGDHVKLAAPNWEFQTNNNNTSNFFDNGGFSWSIPNSSTSSSQVKPNHNFEEIKWSEYLNTPFFIGSTVQSQTSQPIYIKSETDYLANVSNMTDPWSQNENLGTTETSDVFSKDLQRMAVSFGQSL

>ATMYB50(AT1G57560)

MKRHSCCYKQKLRKGLWSPEEDEKLLNYITKHGHGCWSSVPKLAGLERCGKSCRLRWINYLRPDLKRGAFSSEEQNLIVELHAVLGNRWSQIAARLPGRTDNEIKNLWNSCIKKKLMKKGIDPITHKPLSEVGKETNRSDNNNSTSFSSETNQDLFVKKTSDFAEYSAFQKEESNSVSLRNSLSSMIPTQFNIDDGSVSNAGFDTQVCVKPSIILLPPPNNTSSTVSGQDHVNVSEPNWESNSGTTSHLNNPGMEEMKWSEEYLNESLFSTQVYVKSETDFNSNIAFPWSQSQACDVFPKDLQRMAFSFGGQTL

>ATMYB106(AT3G01140)

MPIHVRDREKGRLQNLNRDIFCCVSPSIYQSDAKRAAFVIILIMIISPCCDKAGLKKGPWTPEEDQKLLAYIEEHGHGSWRSLPEKAGLQRCGKSCRLRWTNYLRPDIKRGKFTVQEEQTIIQLHALLGNRWSAIATHLPKRTDNEIKNYWNTHLKKRLIKMGIDPVTHKHKNETLSSSTGQSKNAATLSHMAQWESARLEAEARLARESKLLHLQHYQNNNNLNKSAAPQQHCFTQKTSTNWTKPNQGNGDQQLESPTSTVTFSENLLMPLGIPTDSSRNRNNNNNESSAMIELAVSSSTSSDVSLVKEHEHDWIRQINCGSGGIGEGFTSLLIGDSVGRGLPTGKNEATAGVGNESEYNYYEDNKNYWNSILNLVDSSPSDSATMF

>ATMYB73(AT4G37260)

MSNPTRKNMERIKGPWSPEEDDLLQRLVQKHGPRNWSLISKSIPGRSGKSCRLRWCNQLSPEVEHRAFSQEEDETIIRAHARFGNKWATISRLLNGRTDNAIKNHWNSTLKRKCSVEGQSCDFGGNGGYDGNLGEEQPLKRTASGGGGVSTGLYMSPGSPSGSDVSEQSSGGAHVFKPTVRSEVTASSSGEDPPTYLSLSLPWTDETVRVNEPVQLNQNTVMDGGYTAELFPVRKEEQVEVEEEEAKGISGGFGGEFMTVVQEMIRTEVRSYMADLQRGNVGGSSSGGGGGGSCMPQSVNSRRVGFREFIVNQIGIGKME

>ATMYB43(AT5G16600)

MGRQPCCDKVGLKKGPWTIEEDKKLINFILTNGHCCWRALPKLSGLLRCGKSCRLRWINYLRPDLKRGLLSEYEEQKVINLHAQLGNRWSKIASHLPGRTDNEIKNHWNTHIKKKLRKMGIDPLTHKPLSEQEASQQAQGRKKSLVPHDDKNPKQDQQTKDEQEQHQLEQALEKNNTSVSGDGFCIDEVPLLNPHEILIDISSSHHHHSNDDNVNINTSKFTSPSSSSSSTSSCISSVVPGDEFSKFFDEMEILDLKWLSSDDSLGDDISKDGKFNNSTVDTMNLWDINDLSSLDMFMNEHDDGFIGNGNGCSRMVLDQDSWTFDLL

>ATMYB16(AT5G15310)

MGRSPCCDKLGLKKGPWTPEEDQKLLAYIEEHGHGSWRSLPEKAGLHRCGKSCRLRWTNYLRPDIKRGKFNLQEEQTIIQLHALLGNRWSAIATHLPKRTDNEIKNYWNTHLKKRLVKMGIDPVTHKPKNETPLSSLGLSKNAAILSHTAQWESARLEAEARLARESKLLHLQHYQTKTSSQPHHHHGFTHKSLLPNWTTKPHEDQQQLESPTSTVSFSEMKESIPAKIEFVGSSTGVTLMKEPEHDWINSTMHEFETTQMGEGIEEGFTGLLLGGDSIDRSFSGDKNETAGESSGGDCNYYEDNKNYLDSIFNFVDPSPSDSPMF

>ATMYB48(AT3G46130)

MKMMQEEGNRKGPWTEQEDILLVNFVHLFGDRRWDFIAKVSGLNRTGKSCRLRWVNYLHPGLKRGKMTPQEERLVLELHAKWGNRWSKIARKLPGRTDNEIKNYWRTHMRKKAQEKKRPVSPTSSFSNCSSSSVTTTTTNTQDTSCHSRKSSGEVSFYDTGGSRSTREMNQENEDVYSLDDIWREIDHSAVNIIKPVKDIYSEQSHCLSYPNLASPSWESSLDSIWNMDADKSKISSYFANDQFPFCFQHSRSPWSSG

>ATMYB53(AT5G65230)

MGRSPSSDETGLKKGPWLPEEDDKLINYIHKHGHSSWSALPKLAGLNRCGKSCRLRWTNYLRPDIKRGKFSAEEEETILNLHAVLGNKWSMIASHLPGRTDNEIKNFWNTHLKKKLIQMGFDPMTHQPRTDDIFSSLSQLMSLSNLRGLVDLQQQFPMEDQALLNLQTEMAKLQLFQYLLQPSPAPMSINNINPNILNLLIKENSVTSNIDLGFLSSHLQDFNNNNLPSLKTLDDNHFSQNTSPIWLHEPPSLNQTMLPTHDPCAQSVDGFGSNQASSSHDQEVAVTDSVDWPDHHLFDDSMFPDISYQS

>ATMYB108(AT3G06490)

MDEKGRSLKNNNMEDEMDLKRGPWTAEEDFKLMNYIATNGEGRWNSLSRCAGLQRTGKSCRLRWLNYLRPDVRRGNITLEEQLLILELHSRWGNRWSKIAQYLPGRTDNEIKNYWRTRVQKHAKQLKCDVNSQQFKDTMKYLWMPRLVERIQSASASSAAAATTTTTTTTGSAGTSSCITTSNNQFMNYDYNNNNMGQQFGVMSNNDYITPENSSVAVSPASDLTEYYSAPNPNPEYYSGQMGNSYYPDQNLVSSQLLPDNYFDYSGLLDEDLTAMQEQSNLSWFENINGAASSSDSLWNIGETDEEFWFLQQQQQFNNNGSF

>ATMYB21(AT3G27810)

MEKRGGGSSGGSGSSAEAEVRKGPWTMEEDLILINYIANHGDGVWNSLAKSAGLKRTGKSCRLRWLNYLRPDVRRGNITPEEQLIIMELHAKWGNRWSKIAKHLPGRTDNEIKNFWRTRIQKYIKQSDVTTTSSVGSHHSSEINDQAASTSSHNVFCTQDQAMETYSPTPTSYQHTNMEFNYGNYSAAAVTATVDYPVPMTVDDQTGENYWGMDDIWSSMHLLNGN

>ATMYB100(AT2G25230)

MKKNYQKKNIKVVSTSKYLKKSDIDKVNWTESEDIKLKEIMALGPKNKWTKVAKKFEGRTGKQCRERWYNHARPNIKKTAWSEEEDQILIEAHKVLGTKWVEIAQQLPGRSDNNIKNHWNTTKRRVQNKRGGTVNPVGNNILENYIRCITINNEDFLKTDGSYGEPTNIESDDDSKDMLYGEMNLSLETITTQTTKPLTNASTTSPYVQMPEDNYTMEDCESLEDILELLRWWE

>ATMYB119(AT5G58850)

MEDRRLVHGAAPPLTAVERFLYGQKNDALCSKKQESSRDQPIVKTKISIETRSDNKENTTFGPTREKHLVLNGGNRNPTGEVVARSAARDYQNSTKKRSSKNLIKGQWTAEEDRKLIRLVRQHGERKWAMISEKLEGRAGKQCRERWHNHLRPDIKKDGWSEEEERVLVESHMRIGNKWAEIAKLIPGRTENSIKNHWNATKRRQNSKRKHKRESNADNNDRDASPSAKRPCILQDYIKSIERNNINKDNDEKKNENTISVISTPNLDQIYSDGDSASSILGGPYDEELDYFQNIFANHPISLENLGLSQTSDEVTQSSSSGFMIKNPNPNLHDSVGIHHQEATITAPANTPHLASDIYLSYLLNGTTSSYSDTHFPSSSSSTSSTTVEHGGHNEFLEPQANSTSERREMDLIEMLSGSIQGSNICFPLV

>ATMYB115(AT5G40360)

MYHQNLISSTPNQNSNPHDWDIQNPLFSIHPSAEIPSKYPFMGITSCPNTNVFEEFQYKITNDQNFPTTYNTPFPVISEGISYNMHDVQENTMCGYTAHNQGLIIGCHEPVLVHAVVESQQFNVPQSEDINLVSQSERVTEDKVMFKTDHKKKDIIGKGQWTPTEDELLVRMVKSKGTKNWTSIAKMFQGRVGKQCRERWHNHLRPNIKKNDWSEEEDQILIEVHKIVGNKWTEIAKRLPGRSENIVKNHWNATKRRLHSVRTKRSDAFSPRNNALENYIRSITINNNALMNREVDSITANSEIDSTRCENIVDEVMNLNLHATTSVYVPEQAVLTWGYDFTKCYEPMDDTWMLMNGWN

>ATMYB58(AT1G16490)

MGKGRAPCCDKTKVKRGPWSHDEDLKLISFIHKNGHENWRSLPKQAGLLRCGKSCRLRWINYLRPDVKRGNFSAEEEDTIIKLHQSFGNKWSKIASKLPGRTDNEIKNVWHTHLKKRLSSETNLNADEAGSKGSLNEEENSQESSPNASMSFAGSNISSKDDDAQISQMFEHILTYSEFTGMLQEVDKPELLEMPFDLDPDIWSFIDGSDSFQQPENRALQESEEDEVDKWFKHLESELGLEENDNQQQQQQHKQGTEDEHSSSLLESYELLIH

>ATMYB35(AT3G28470)

MGRPPCCDKSNVKKGLWTEEEDAKILAYVAIHGVGNWSLIPKKAGLNRCGKSCRLRWTNYLRPDLKHDSFSTQEEELIIECHRAIGSRWSSIARKLPGRTDNDVKNHWNTKLKKKLMKMGIDPVTHKPVSQLLAEFRNISGHGNASFKTEPSNNSILTQSNSAWEMMRNTTTNHESYYTNSPMMFTNSSEYQTTPFHFYSHPNHLLNGTTSSCSSSSSSTSITQPNQVPQTPVTNFYWSDFLLSDPVPQVVGSSATSDLTFTQNEHHFNIEAEYISQNIDSKASGTCHSASSFVDEILDKDQEMLSQFPQLLNDFDY

>ATMYB75(AT1G56650)

MEGSSKGLRKGAWTTEEDSLLRQCINKYGEGKWHQVPVRAGLNRCRKSCRLRWLNYLKPSIKRGKLSSDEVDLLLRLHRLLGNRWSLIAGRLPGRTANDVKNYWNTHLSKKHEPCCKIKMKKRDITPIPTTPALKNNVYKPRPRSFTVNNDCNHLNAPPKVDVNPPCLGLNINNVCDNSIIYNKDKKKDQLVNNLIDGDNMWLEKFLEESQEVDILVPEATTTEKGDTLAFDVDQLWSLFDGETVKFD

>ATMYB3R5(AT5G02320)

MSSSSNPPVCSPEKEERSEMKIEIQCMENKQPLAASCSSASEGSGCFFLKSPEIATPATVSSFPRRTSGPMRRAKGGWTPEEDETLRRAVEKYKGKRWKKIAEFFPERTEVQCLHRWQKVLNPELVKGPWTQEEDDKIVELVKKYGPAKWSVIAKSLPGRIGKQCRERWHNHLNPGIRKDAWTVEEESALMNSHRMYGNKWAEIAKVLPGRTDNAIKNHWNSSLKKKLEFYLATGNLPPPASKFIVLKDIADGDRDSKQSSATKPFKDSDSLTQTSSGNTDSNEVGRDHFDSSSALLEEVAASRRIGVNEYACSPVEYKPQLPNLEPISEEVRINSKAYFERSIQRKVENGFGTPKHGNLYYKSPLDYYFPSEADLQHMYGYECGCSPGAASPVSLMTTPCNKDSGLTATRSPESFLREAARTFPNTPSIFRKRRKVVLAAKTDAVVVVNGVVKEVDRKEESKDMRKSLLLETTDNCSDDEELGLNGNAFNLSPPYRLRAKRTAVIKSRQLEFTSEKEKQPDNEIEFTSAKEKQPDNEIKTSEEDKPV

>ATMYB52(AT1G17950)

MMCSRGHWRPAEDEKLRELVEQFGPHNWNAIAQKLSGRSGKSCRLRWFNQLDPRINRNPFTEEEEERLLASHRIHGNRWSVIARFFPGRTDNAVKNHWHVIMARRGRERSKLRPRGLGHDGTVAATGMIGNYKDCDKERRLATTTAINFPYQFSHINHFQVLKEFLTGKIGFRNSTTPIQEGAIDQTKRPMEFYNFLQVNTDSKIHELIDNSRKDEEEDVDQNNRIPNENCVPFFDFLSVGNSASQGLC

>ATMYB114(AT1G66380)

MEGSSKGLRKGAWTAEEDSLLRQCIGKYGEGKWHQVPLRAGLNRCRKSCRLRWLNYLKPSIKRGKFSSDEVDLLLRLHKLLGNRWSLIAGRLPGRTANDVKNYWNTHLSKKHEPCCKTKIKRINIITPPNTPAQKVDIF

>ATMYB9(AT5G16770)

MGRSPCCDENGLKKGPWTQEEDDKLIDHIQKHGHGSWRALPKQAGLNRCGKSCRLRWTNYLRPDIKRGNFTEEEEQTIINLHSLLGNKWSSIAGNLPGRTDNEIKNYWNTHLRKKLLQMGIDPVTHRPRTDHLNVLAALPQLIAAANFNSLLNLNQNVQLDATTLAKAQLLHTMIQVLSTNNNTTNPSFSSSTMQNSNTNLFGQASYLENQNLFGQSQNFSHILEDENLMVKTQIIDNPLDSFSSPIQPGFQDDHNSLPLLVPASPEESKETQRMIKNKDIVDYHHHDASNPSSSNSTFTQDHHHPWCDTIDDGASDSFWKEIIEQTCSEPWPFPE

>ATMYB85(AT4G22680)

MGRQPCCDKLGVKKGPWTVEEDKKLINFILTNGHCCWRALPKLAGLRRCGKSCRLRWTNYLRPDLKRGLLSHDEEQLVIDLHANLGNKWSKIASRLPGRTDNEIKNHWNTHIKKKLLKMGIDPMTHQPLNQEPSNIDNSKTIPSNPDDVSVEPKTTNTKYVEISVTTTEEESSSTVTDQNSSMDNENHLIDNIYDDDELFSYLWSDETTKDEASWSDSNFGVGGTLYDHNISGADADFPIWSPERINDEKMFLDYCQDFGVHDFGF

>itf12g04080.t1

MVNSLSSAWPSPSGLMRKGAWTEEEDNLLRKCIQKYGEGKWHLVPLRAGLNRCRKSCRLR

WLNYLRPDIKRGDFSVDEVDLIMRLHRLLGNRWSLIAGRIPGRTANDVKNYWNTHIQKKV

FAMAAASSRMQDNWKGKAPEISKNTVVKPQPRRFLNTSSLSRTPMTGKATAVTNYAQIQA

HTLPQRETTTTSDLVMENVQKNDTIASFPSELETTTFDDRVQWWEELLFDKELNDEGTAC

MHEGQIGWSHLPTDIDLLELLS

>itf12g04100.t1

MANSCAWSGVRKGAWSEEEDNLLRKCIQKYGEGKWHLIPFRAGLNRCRKSCRLRWVNYLR

PDIKRGDFKLDEVDLILRLHKLLGNRWSLIAGRIPGRTANDVKNYWNTHIQKKVFAMAAA

SSDNWKGKATEIRENTVVRPRPRRLSRTPLTGKATAVTYDAQIQGHKIPTSELVMENLQQ

NNTITSELETTTLNDRLQWWEDFLFDNEGNTCMNQGQVCWANFPTDMDLSELLS

>itf12g04070.t1

MADSSSSEPPSGVKKGAWTEQEDNLLRKCIHKYGEGKWHLVPVRAGLNRCRKSCRLRWLN

YLRPDIKRGDFNLDEIDLIMRLHKLLGNRWSLIAGRIPGRTANDVKNLWNTRLQKKTIIA

NNTPSSGQEKWKDKAPKTTENTAVIRPRPRRFVMNSSSRTLPITGKTTIVTSEVVQLQGH

NKPPEAAESTSAPRLIENVDPNNSIIDLPGGAETSDDLGQWLDDFLLDMEFDGDGMACMQ

EGQIEWCDFHIDSDILDLLS

>itf00g02390.t1

MVNSSARSSPRVRKGAWSEEEDDLLRKCIQKFGEGKWHLVPFRAGLNRCRKSCRLRWLNY

LHPDIKRGHFSLEEADLILRLHKLLGNRWSLIAGRIPGRTANDVKNYWHSHLKKKVVGMH

MTTSSNSSRQDNNWDDEKSKAPQITENTLFRPRPRRFFRTSPALSTLTGKANAPHQLQAS

QSEATPPPDLLMVNNVQQTNNSIATNLPSETTSVQWWEDLLYDDNEHQGTTDMHQGIIDW

TDDGFPIDVDLLTLLDPTN

>itf05g14440.t1

MDKTAAKTGMKKGAWTEEEDNLLRKYIEEFGEGKWHQVPAKAGLNRCRKSCRLRWLNYLR

PNIKRGEFAWDEVDLIIRLHKLLGNRWSLIAGRIPGRTANDVKNYWNTHLKKTAEFGMPK

PPRRQQENGRGNIVDIGPPPRPPNFSKLSSFLSLGTETTTTTTTSMPDNDVKTTMPHNDV

SKRTFTPSPPADSVTQQWWENLLAIGDSRDGAITWSPSLTISDEGDKAVADGETWSRVIG

DGNSAFIEEGEFSWNEFQFPMGTSKWNDGIWDLGEQLK

>itf13g08560.t1

MSSRKSGGARMHVHKGAWTAEEDKKLTQYIENHGAKKWKTVAIKSGLNRCGKSCRLRWLN

YLRPNIKRGNIAEDEADLILRLHKLLGNRWSLIAGRLPGRTDNEIKNYWNTHLSKKVTQV

GKSSLSATQNQPPKANAEQVGGNKESEEEEDPELNFDVDEFFDFSVEGTYGTEWVNKFLE

VEN

>itf13g08560.t2

MSSRKSGGARMHVHKGAWTAEEDKKLTQYIENHGAKKWKTVAIKSGLNRCGKSCRLRWLN

YLRPNIKRGNIAEDEADLILRLHKLLGNSRWSLIAGRLPGRTDNEIKNYWNTHLSKKVTQ

VGKSSLSATQNQPPKANAEQVGGNKESEEEEDPELNFDVDEFFDFSVEGTYGTEWVNKFL

EVEN

>itf04g23250.t1

MRTQSSSSAKGAAGASPASKAKGKGKTTPCCSKVGLKRGPWTPEEDKLLTDYINKEGEGR

WRTLPKSAGLLRCGKSCRLRWMNYLRPTVKRGHITPDEEDLILRLHRLLGNRWSLIAGRI

PGRTDNEIKNYWNTHLTKKLISQGIDPRNHKPLLVNPTNHTSSSYSAPIIKPTPIHVGLS

NQDKTVKINSSVNVGGTTNHQIDQPNSMGGTTNQTTGNGGGDEDFNMDGGGIISCCIGED

NEDNVGMDFCPDEDVFSTFFDSLMNHDVFFAASQNNQQSNHDITPLQPSTSEHNDNNQPL

NPLQQMNFPFSTAGWVDGDGDGDDFLP

>itf08g07790.t1

MGRAPCCSKEGLRKGPWSAKEDLLLTNHIQEHGEGQWRSLPKKAGLLRCGKSCRLRWMNY

LRPGIKRGNFSQEEEDLIVRLHSLLGNRWSLIAGRLPGRTDNEIKNYWNTHLLKKLKSAG

IEPKPRQAKKKPAKPRPDSQNLNPNKKKKQKARNSEQPLRQADQTTPPEKTQNRTKVYAP

KPIRLSPPGISRINSLEDVAGSVSSSSGEVDNKAAVTAEPPPPFIPWHLYELGGGGDVDF

CDQILDGCDLSSPKCSGPTTDGLLEKVYDEYLHLLSENCFESLTDDYLCDYPFVDDNVAP

TTSSNNSDLN

>itf12g17000.t1

MARTYLVSALFIALLPPVQSWLQRKGAATLTNLWSFFSLSFLNLGEREMGRAPCCSKEGL

KKGPWSTKEDLLLTNYIQQHGEGQWRSLPKKAGLLRCGKSCRLRWMNYLRPGIKRGNFSP

EEEDLIVRLHSLLGNRWSLIAGRLPGRTDNEIKNYWNTHLLKKLKSSGIEPRPPRKIVTS

KKKATIPKIVASKKPANNNSRNKKLQRKEISDDNQRCYKVYAPKAIRLSSRNNSVEDIAG

SVSSSSGEVENKGIIDGSSSFIPWNLYELRDDFCAEVLTAAGDDLSPQCALPVGSDDCLL

DKVYDEYLQLLSENCFLEDDPFGANL

>itbMYB113

MVINSSSAWPSPSGLMRKGAWTEEEDNLLRKCIQKYGEGKWHLVPLRAGLNRCRKSCRLRWLNYLRPDIKRGDFSVDEVD

LIMRLHRLLGNRWSLIAGRIPGRTANDVKNYWNTHIQKKVFAMAAASSKMQDNWKGKAPEIRENTVVRPQPRRFLNTSSL

SRTSMAGKATAVTYDAQIQAHALPHPEPTTSDLVMENVQKNDTIATFPSELETTTFDDRVRWWEDLLFDKELNDEGTACM

HEGQVGWSHLPTDIDLLELLS

>itbMYB75-like1

MANSCAWSGVRKGAWSEEEDNLLRKCIQKYGEGKWHLIPFRAGLNRCRKSCRLRWLNYLCPDIKRGDFKLDEVDLILRLH

KLLGNRWSLIAGRIPGRTANDVKNYWNTHIQKKVFAMAAASSDNWKGKATEMRENTVVRPRPRRLSRTPLTGKAAAVTCD

AQIQGHKIPTSELVMENLQQNNTITSELETTTSNDRLQWWEDFLFDNEGNTCMNQGQVCWANFPTDMDLSELLS

>itbMYB75-like2

MADSSSSEPPSGVKKGAWTEQEDNLLRKCIHRYGEGKWHLVPVRAGLNRCRKSCRLRWLNYLRPDIKRGDFNLDEIDLIM

RLHKLLGNRWSLIAGRIPGRTANDVKNLWNTRLQKKTIANNTPSSGQETWKDKAPKTTENTAVIRPRPRRFAMTSSSRTL

PITGKTTIVTSEVVQLPPPAEAAESTSEPRLIENVDPKSMPGETETSDDLGQWLDDFLFDMEFDGDGMACMQEGQIEWCD

FHIDSDLLDLLS

>itbMYB90-like1

MVNSSARSSPRVRKGAWSEEEDDLLRKCIQKFGEGKWHLVPFRAGLNRCRKSCRLRWLNYLHPDIKRGHFSLEEADLILR

LHKLLGNRWSLIAGRIPGRTANDVKNYWHSHLKKKVVGMHMTTSSNSSRQDNNWDDEKSKAPQITENTLFRPRPRRFFRT

SPALSTLTGKAPPPPHQLQASQSESTPPPDLLMVNNVQQNNNSIATNLPSETTSVQWWEDLLYDDNEQLNHQGTTDMHQG

IIDWTDDGFPIDVDLLTLLDPTN

>itbMYB90-like2

MDKTAAKTGMKKGAWTEEEDNLLRKYIEEFGEGKWHQVPAKAGLNRCRKSCRLRWLNYLRPNIKRGEFAWDEVDLIIRLH

KLLGNRWSLIAGRIPGRTANDVKNYWNTHLKKTAEFGKPPRRQQENGRENIVIGPPNSSKISSFLSLGTETTTTKTTMPD

NDVSKRTFTPSPPADSVTQWWENLLAIGERDGAITWSPSLTTSDEGDKAVGDGETWSRVIGDGNSTFIDEGEFSWIEFQF

RMGTSKWNDGIWDLGE

>itbMYBl14-like

MTSRKSGGARMHVHKGAWTAEEDKKLTQYIENHGAKKWKTVAIKSGLNRCGKSCRLRWLNYLRPNIKRGNIAEDEADLIL

RLHKLLGNRWSLIAGRLPGRTDNEIKNYWNTHLSKKVTQVGKSSLSATQNQPPKANAEQVGGNKESEEEEDPELNFDVDE

FFDFSVEGTYGTEWVNKFLEVEN

>itbMYB5-like

MRTQSSSSAKGAAGASPPSKAKGKGKTTPCCSKVGLKRGPWTAEEDKLLTDYINKEGEGRWRTLPKLAGLLRCGKSCRLR

WMNYLRPTVKRGHITPDEEDLILRLHRLLGNRWSLIAGRIPGRTDNEIKNYWNTHLTKKLISQGIDPRNHKPLLVNPTNN

PKNHTSSSSSVPIIKPTPIHVGLSNKDKTVKINSSVNVGGTTNHQIDQPNSVGGTTNQTTGNGGADEDFNMDGGGIISCC

IGEDNEDNVGMDFCPDEDVFSTFFDSLMNHDVFFAASQNNQQSNHDITPLQPSTSEHNNNNNNQPLNPLEQMNFPFSTAG

WVDGDDGDDFLP

>itbMYB308-like1

MGRAPCCSKEGLRKGPWSAKEDLLLTNHIQEHGEGQWRSLPKKAGLLRCGKSCRLRWMNYLRPGIKRGNFSQEEEDLIVR

LHSLLGNRWSLIAGRLPGRTDNEIKNYWNTHLLKKLKSAGIEPKPRQAKKKPAKPRPDSQNLNPNKKKKQKARNSEQPLR

QADQTTPPEKTQNRTKVYAPKPIRLSPPGISRINSLEDVAGSASSSSGEVDNKAAVTADPPPFIPWHLYELGGDVDFCDQ

ILDGCDLSSPKCSGPTTDGLLEKVYDEYLHLLSENCFESLTDDYLCDYPFVDDNVAPTTSSNNSDLN

>itbMYB308-like2

MGRAPCCSKEGLKKGPWSTKEDLLLTNFIQQHGEGQWRSLPKKAGLLRCGKSCRLRWMNYLRPGIKRGNFSPEEEDLIVR

LHSLLGNRWSLIAGRLPGRTDNEIKNYWNTHLLKKLKSSGIEPRPPRKIVTSKKKATIPKIVTSKKPANNSRNKKLQRKE

ISDHNQRYKVYAPKAIRLSSRNNSVEDIAGSVSSSSGEVENKGIIDGSSSFIPWNLYELRDDFCAEVLTAAGDDLSPQCA

LPVGSDDCLLDKVYDEYLQLLSENCFLEDDPFGANL

>itbMYBZm38-like

MGRSPCCEKEHTNKGAWTKEEDDRLIRYIKKHGEGCWRTLPKAAGLLRCGKSCRLRWINYLRPDLKRGNFTEEEDELIIN

LHSLLGNKWSLIAARLPGRTDNEIKNYWNTHIKRKLLSRGIDPQTHRPLTTSAAGAAAVITPATPTPQQPSPTTSSGGLS

SSEETAYLNLELSISLPTPADEEHSNSGSSHAVCLCQKLGFQSGNACNCSKMAASINYAARDGMQTLFTPLSL

>itbMYB3-like1

MGRAPCCSKDGLKKGPWSTKEDSLLTTYIQQHGEGHWRSLPKNAGLLRCGKSCRLRWMNYLRPGIKRGNFAPEEDDLIVR

LHSLLGNRWSLIAGRLPGRTDNEIKNYWNTHLLKKLKSEGIQPKPARTSRAIPKNKNTAKPDKTGRDEKKKKHRHVSKPD

VENGVKDRPKMIKVYAPRPVRVSTGLARNYSSDNLAVVSASHGSIASNNNNNNTVEGTSFVPWNMYEVGDDLFNDFMDGC

DLSANYLLPDPDDALLEKVYDEYLELLSEDSYLQTCLPS

>itbMYB308-like3

MGRSPCCEKEHTNKGAWTKEEDERLIRYIKKHGEGCWRTLPKAAGLLRCGKSCRLRWINYLRPDLKRGNFTEEEDELIIN

LHSLLGNKWSLIAARLPGRTDNEIKNYWNTHIKRKLLSRGIDPQTHRPVSSAQNTAAADTAAVPVANNLDQQHETAAGGC

LPWMRNTKAENSNNTSSTTEDSNSSNGLSSEEIELHPAAPLINLELSISLPQSPAPSPSTTKLTGKEFNDQTLKSHIFFS

QRAVCLCYNLGFQNSNACNCDKMMTTSSINAEAGMHSFYRPLSL

>itbMYB6-like1

MGRSPCCEKAHTNKGAWTKEEDQRLINYIRSHGEGCWRSLPKAAGLLRCGKSCRLRWINYLRPDLKRGNFTEEEDDLIIK

LHSLLGNKWSLIAARLPGRTDNEIKNYWNTHIKRKLLSRGLDPQTHRPINAAAAAGGGGGGSAAKDICLDFRNAAAPAKS

SNEKATLSLSQEDTKCNSGTTTEESQSHQQQKDDQTALNLGLSIGLSTAAETPSSSNTAESVAPPQEPPSAAAVGYAMTQ

TVCLCWQLGWSPSGKLCTKCHNSYKWFP

>itbMYB308-like4

MGRSPCCEKAHTNKGAWTKEEDERLIAYIKAHGEGCWRSLPKAAGLLRCGKSCRLRWINYLRPDLKRGNFTHEEDELIIK

LHSLLGNKWSLIAGRLPGRTDNEIKNYWNTHIRRKLLSRGIDPTTHRPINGGAEPPKETTISFGAVKPEDAENNNSIPGK

DSEPKKEENKEETLLFKSEEPQVAEACPDLNLELRISPPSSEETRPPPLPLEAAKSGGGGRVNGLCFACILGIPNSIDCT

CNNNEDYSSGSSN

>itbMYB330-like1

MGRSPCCEKAHTNKGAWTKEEDQLLINYIRLHGEGCWRSLPKAAGLFRCGKSCRLRWINYLRPDLKRGNFTQQEDDLIIK

LHSLLGNKWSLIAARLPGRTDNEIKNYWNTQIKRKLISRGIDPQTHRPLDSSAGGGGGTTKPENISMDLSSSAPSQEETK

CSCGTTSEESHHQSLKDKQRNEQMGGLDLSIGLALHPKTEDSAESTASGESLPVAPPPPAAVELSVTEAVCLCWQLGSRS

GGLCNK

>itbMYB4-like1

MVRAPCCEKMGMKKGPWTPEEDQILTSFIQRYGHENWRALPRQAGLLRCGKSCRLRWINYLRPDIKRGNFTKDEEETIIQ

LHQTLGNRWSAIASRLPGRTDNEIKNFWNTHLKKRLQHHGSPYSPNNINVVGNITPIQIGDSSIHLRFPAPMTVNCNSVG

RQNRMYSSSLMTTKMEEEESMQESYQNLGTTSNDDSGIVYLPSSSSVLPMELGGCETSSSISNDAVFWYNLLINAGNTS

>itbMYB114-like

MKAKEVKTGLKRGFWTPEEDLTLKKCVETHGEGNWATISKKSGLMRSGKSCRLRWKNYLRPNIKRGMMSEDEKDLIIRMH

KLLGNRWSLIAGRLPGRTDNEVKNFWNTHLNKRSRRGKRMKITPKDDDSISASIPTQSMENTNLVEGSIKQEDEMDSIMN

SWMEHMGIENCNINSSISTNNLPWIFEDVPLIPILDDVLLDAFQRTGDETLLDGIHPFLL

>itbMYB41-like1

MGRAPCCDKDGLKKGPWTPEEDQKLVDYIQKHGYGNWRTLPKNAGLQRCGKSCRLRWTNYLRPDIKRGRFSFEEEETIIQ

LHSILGNKWSAIAARLPGRTDNEIKNYWNTHIRKRLLRMGIDPVTHSPRLDLLDLSSILNTPSPLYNYPCQQINLSRLLG

LQQNLLNPHVLRLASSLFSPPVPRHGLNPELGLQNNVNQMCNLENPGFQTVQDNVQFSCPQSVTQEQTELPVQTDVNNVT

FSQVTQQPNNVDQFQPQFSSFPIGNCQENEWQNNGIPSSLTEDYLPLQNYGCYQPATVDPQSIMDPPPSDASTFCRSFQS

VLSTPSSSPAPLNSNSTYINSCSTTTMDDEIDISYSSNLIDFDYSSILEVNEFM

>itbMYB30-like1

MGRSPCCEKMGLKKGPWTKDEDEILVDYISRHGHGNWRALPKHAGLLRCGKSCRLRWINYLRPDIKRGNFSHEEEDAIIK

LHQALGNRWSVIAARLPGRTDNEIKNIWHTRLKKRLNDYDLVSPQPRLKSKSQPLKPFAMDLLITNGSMLTPSSPPHSST

TTSTEFHASSACSISSDCVVSDAVLQSDPPEVDESFWSQVFSSENSSDAGDLPATVDGGSRFDSTENETYETKSSVEFWH

RLFSKAEDLPVSPEL

>itbMYB41-like2

MGRSPCCVDKSGLKKGPWTPEEDQKLIHYIQAHGPGNWRTLPKNAGLQRCGKSCRLRWTNYLRPDIKRGRFSFEEEETII

QLHSVLGNKWSAIAARLPGRTDNEIKNYWNTHIRKRLLRMGIDPVTHTPRLDLLDLSSILGTSTHLNFPSLLGLQAILNP

ELLRLATNLFASQNDNNINPDLLSNPHLQNQNPQMLLRKLQESQLLINAPNFQSNNSDHQFQHPLPPCTTSNNNVSPACS

DLPMQPYSTMNSSNLQNNGQVFQESLLQSLENYSGCFGSNQSLSNSSNQSGLENFSFDSSVLSTPLNSSSTLVNSGGANT

EDEKESFCSNLMRFDIPDSLDFEDLL

>itbMYB106-like1

MGRSPCCEKEGLKKGPWTPEEDKQLLAYIEQYGHGSWRALPAKAGLQRCGKSCRLRWTNYLRPDIKRGNFSLQEEQSIIQ

LHALLGNRWSAIAAHLPKRTDNEIKNYWNTHLKKKLSKMGIDPMTHRPKINSSFGSAANLSHMAQWETARLEAEARLVRH

SKFISSSLISPHHFRNNPPPPPPPKVPPSLDVLKAWQETWTKPPRTRVSSSVDGGAFVSNATPHHSPTTLNFSDQNLCNV

ETPYVHESTSNIGNPNNTTGDDIIPHVAMDPLSELPTFIHGFSELSPETLTGYLDDDDVVGNCGTADMEDNSRYWNSILN

NLVASPVGSPVF

>itbMYB41-like3

MGRAPCCDKDGLKRGPWTAEEDQKLIDYINKNGYGNWRTLPTNAGLQRCGKSCRLRWMNYLRPDIKRGRFSSEEEHIIIQ

LHSILGNKWSAIAARLPGRTDNEIKNYWNTHIRKKLLRMGIDPVTHRRRVDLLDLSSILNNPSLLYNSRILGAQTLANPH

LLRFAASQHHNNNVINSEKANNVVQDTQQLHAPPPLVQDFPIYSPNMAAAQITQQPNVEFGLENYPANDFWLPESEMTQD

YLLPPLQNYGYYEAAVDPQSAMDPPAPAADESCRFGFRQVWSTPSSSQVNSGSTTTTEDEREISYGSNLLNFDVANIFGV

NEFM

>itbMYB41-like4

MQVVAGTMRRPSSPTLSGSSGGRGDENAGGVKKGPWTPEEDKKLVDYIRKHGHGSWRAVPKLAGLNRCGKSCRLRWTNYL

RPDIKRGKFEEEEEQLIIKLHSVLGNKWSAIAMRLPGRTDNEIKNHWNTHLRKRLLQMGIDPVTHRPRTDINFIDALANL

PQLLVAAANMGNNSNVANPLWDSINALRLCSDAAQIANELQLLQNFMALQLQLRGSVNNTTNEAQSQIPELATQFGSCNQ

LLDHLALLNPQLQGGLCNLGSSYNFSRLPPNISCSGSVATSSTSQNSEIQIHHPGIISNETNQGQTTNSNVSRINDDSNK

LMTNAFTVSSSSPLNVPSSEGIPSNPIFPTLIPASPFPENPSSSIDWETDKEKYTISANLKHDIPNHVPNATTTFEAWRD

IKVDDDEATDSYWQDILYQTFSP

>itbMYB3-like

MRKASCDHSHHHHHHEINKGAWSKQEDQKLLDYIRKHGEGGWRDLPKAAGLLRCSKSCRLRWMNHLKQTAKRGNFGDDEE

DLIIKLHALLGDRWSLIAGRLPGRTEEEVKNYWNSHIKKKLLDMGIDPNNHRLSCTYSRPHNIAAQTSAGKSRVTSPEKQ

RVESDGEVSDAGSSNVR

>itbMYB93-like

MGKVPCCDENGLKKGPWTPEEDKKLSDFIEKHGHGSWRALPKLAGLNRCGKSCRLRWTNYLRPDIKRGKFSQEEEQTILH

LHSILGNKWSTIATHLPGRTDNEIKNFWNTHLKKKLIQMGYDPMTHRPRTDLFSNLPNLLALATLLQSHPLEEHAAAAAR

LQAQAATQNMAAKIQYLQMLFQSSSSSSMTTTSSSSYDNNGDFWDFNLPNLSNKETDNNPLLSSLSQMENQTLFSIENSG

ASQLLHNEQVPFNFQTHLNNDNNNKSLNSDSILPPLTDCFLNNNNNNNQGDSSSTSSNGDYQGTSSPSSYNWPELFLEEA

FMHDIS

>itbMYB106-like2

MGRSPCCEKVGLKKGPWTLEEDKQLLAYIEQYGHGSWRALPAKAGLQRCGKSCRLRWTNYLRPDIKRGNFSLQEEQSIIQ

LHALLGNRWSAIAAHLPKRTDNEIKNYWNTHLKKKLCKMGIDPMTHRPKINSSFGSAANLSHMAQWETARLEAEARLVRP

SKFIGSSLISPRHFRNNPPPPPPPKVPPPLDVLKAWQETWTKPPRTRVSSDGAFVSTATLNQSPTTLNFSDQILNFSDQN

LCNKETQSSIGNPNNTAGDGIIPHVSMDPLTELPTFIHGLSDLSPEALTGYLDDDNFVGNCGTADVEDNSRYWNSILNNL

VASPVGSPVF

>itbMYB4-like2

MVRAPCCEKMGLKKGPWTPEEDQILISYIQKNGHGNWRALPKQAGLLRCGKSCRLRWTNYLRPDIKRGNFTKEEEDTIIQ

LHEMLGNRWSAIAARLPGRTDNEIKNVWHTHLKKKLKGYQSPQNAKRQFSGKVAGAVAGDGDVAGDSSTTTSEDDGSTGM

NVLVSSPERSSSASSEMSSVTEGVAVDAPAVGVKQEDVNSSPEYLPEIDESFWTEEAAAGLPWVQVDEFPVVGASLANSD

DVDRMMWHSTTLDDDMDFWYNVFVRSAGELPELPEF

>itbMYB16-like1

MESSPCCEKVGLKKGPWTPEEDKQLLAYIEQYGHGSWQALPEKAGLQRCGKSCRLRWTNYLRPDIKRGNFSLQEEQSVIQ

LHAFLGNRWSAIASHLPKRTDNEIKNYWNTHLKKKLSKMGIDPMTHRPKINSSFGSAANLNHMAQWETARLEAEARHSKT

SKLLYIESSQPVTHTDLTIQIPQLCSDNYKIWKERILLNLGWKELDYAVNNNKPQIPTNSSTPDEIALYERWERSNRLSV

ILIKSNVSDSVRGIVDAYTDVKPLLEALDAQYASSVKSLTSTLIMKFSSLRLKHC

>itbMYB106-like3

MGRSPCCEKVGLKKGPWTLEEDKQLLAYIEQYGHGSWRALPAKAGLQRCGKSCRLRWSNYLRPDIKRGNFSLQEEQSIVQ

LHALLGNRWSAIASHLPRRTDNEIKNYWNTHLKKKLSKMGIDPMTHRPKMNSSFGSAANLSHMAQWETARLEAEARLVRH

SKSIASSLISPHHKPPPPPPKVPPSLDMLKAWQETWTKPPRTRVSLSHVDHGAFLSNATPTTLNFPDQNLCYMETPYVHE

SSSNIVNLNTTGDDIIPHVAMDPLSDLPTFIHGFSELSPETLTGYLDDDNVVGNCGTADMEDNSRYWNSILHNLVMSPVG

SPVF

>itbMYB53-like

MGRSPCCDESTGLKKGPWTPEEDQKLINYINKNGHGSWRALPKHAGLNRCGKSCRLRWTNYLRPDIKRGKFSQEEEQTIL

NLHAILGNKWSAIATHLPGRTDNEIKNFWNTHLKKKLIQMGYDPMTHRPRTDIFSSLPHLIALANLKELVDQHQSSWEEQ

AAMARLQSEMAKFQYLQYLLQTSPAPAGPAFSPNNLNNTVTANNNNNNTNNVSDMEAYSHLLSTVMGTSSSTQMESSVLP

LSSLGQAIQDSAIPFSHLPDLQPPCAIFQQSSDHNKDNNMGQAQGISGFTVLSDGENSPTSPWLPSSLSPSPPPAVGPEP

PTVVDGSGASSYGGAPPSSVVWPDLLLDDDPLFPDL

>itbMYB16-like2

MGRSPCCEKVGLKKGPWTPEEDKQLLAYIEQYGHGSWRALPAKAGLQRCGKSCRLRWTNYLRSDIKRGNFSLQEEQSILQ

LHALLGNRWSAIAAHLPKRTDNEIKNYWNTHLKKKLSKMGIDPMTHRPKINSSFGSAANLSHMAQWETARLEAEARHSKF

ISSAQNSSFRLPTNNPPPPPLPKVPPPLDVLKAWQETWTKPPRTRVSSGGAFVPNATPHQSPTTLNNVSDQNLNFSHQNL

CYVETPYVHESSSNIVNPNSTGDAIIPHVAMDPLSDLPTFIHGFSELSPQTLTGYFDDDNVVGNCGTADMEDNNSYWNII

LNNLVASPVGSPVF

>itbMYB16-like3

MGRSPCCDKLGLKKGPWTPEEDHKLLAYIQEHGHGSWRALPPKAGLQRCGKSCRLRWTNYLRPDIKRGKFSLQEEQSIIQ

LHALLGNRWSAIATHLPKRTDNEIKNYWNTHLKKRLAKMGIDPVTHKPKNDVTLFPRDAAAATLSHMAQWESARLEAETR

LARRSTLACSENVAQISTAAAKIGNSSASDFHGGIFPDNAAWTTEDSVRTANTQHIPTPTGNFVDTFTDLLLSRASGDRR

FCDGGSTDSINGGGSNYYEDNKNYWNSILNLVNSSPSDSPMF

>itbMYB15-like1

MGRTPCCEKVGLKRGRWTAEEDRILTDYIHANGEGSWRSLPKNAGLLRCGKSCRLRWINYLRSDLKRGKFSPQEEEIIIK

SHAILGNRWSLIAAQLPGRTDNEIKNYWNSHLSRKFYSFRRAGSEKTIENLETDLAKAAEQTKRRRGKVSRSAMKKNKTT

DYKHYSNINHAPELPYFQRHKDTIPSLYTEPVNNNVAASAVQIPSSLPILMEKEDIDTSSFLCMVGSEYFSLDDIMPILV

EDMDPTRTILSTSLNGSLENSVKEFGLQVEQPQHDLAKSGNSSIYNYSGLIPSDHHHQFGGGENSESTATSSSFPVEHHC

SLAQNIIDWDDWQYYWDDDSGNNLYNTHNLMPQQNKDDVMLSSPWPWDDTFYI

>itbMYB41-like5

MGRSPCCDKNGLKKGPWTPEEDLKLIHYIQLHGPGNWRTLPKNAGLERCGKSCRLRWTNYLRPDIKRGRFSFEEEETIIQ

LHSVLGNKWSAIAARLPGRTDNEIKNYWNTHIRKRLLRSGIDPVTHSPRLDLLDLTSLLNLTQLNLSGLLGLQALASPEV

LRLLCTLMATQNENNTPQILLQKLQESQLNNNNQVENENPLLLLQKLQENQSPNAPIQNHQAAAFQPCDQFLDTVPSNNV

CASSSLPMQPTNLSAGQVIQESLIPSNGGNLMSGLQNNYYGVFEAEQSLSDWSNQCNFGLDSVLSTPLSSPGSTTFVNSG

SCTEDEKESDCISNMMKFEIPSASLDFEDLL

>itbMYB308-like5

MGRYPCCKDSEEVKKGPWTPEEDQKLSDYVSKNGHGNWQMLPKKAGLNRCGKSCRLRWTNYLRPDIKRGGFSQEEEQIII

NLHSSLGNKWSRIAAHLPGRTDNEIKNFWNTNLRKKLLRMGIDPKTHQPITDINLLLNLSHQIMLSNTNNPLGSALRLQN

ILQFLNSYPILFPTNKQENFPMGLDYNNTINTINNYVSTATQNSSDFIGSDFNPHMKMLTDNAGENNNDHSEYSLPSLVQ

SSSEYIPTLDQTIMPSPGLEANNNEFLAWEEFLKNEEDYSSPLWNDILQAKQM

>itbMYB17-like1

MGRAPCCDKKGLKKGPWTPEEDEKLTEYIKKNGHGSWRSLPKFAGLLRCGKSCRLRWTNYLRPDIKRGPFSPEEEELVVQ

LHGLLGNRWAAIASQLPGRTDNEIKNLWNTHLKKRMISMRIDPQTHKPSSDANGLMWTMPTTPSARHMAQWESARLEAEA

RLSRESLHLVPSPTTVGSETDFFLRMWNSEIGESFRKLKKGEKADCQSPTSETSSSMKCGSASGTTEVDPTLTVHSAADC

NPNKDPEQKSCKSYIEYPLSEMSCTDELDDSSESALQLLLDFPTHNDMSFLEHTDAYTIHPTPFSPKIL

>itbMYB17-like2

MGRTPCCNKEGLRKGAWTAEEDKILVAYITKNGHGNWRSLPKLAGLLRCGKSCRLRWTNYLRPGIKRGQFSSEEVDTIIQ

LHTVLGNKWSAIASHLPGRTDNDIKNFWNSHLRKQGSDPNHQNHQMAHPHGNIDEKVETTLSTNHIVQWDSVSVAPSLLD

LPSASKMDHHDPFLRLWNSEVGEAFCSFKKPRGVPCQSPVSSSSKFESSSGITLHSEPASKLLSSADTVEGVPMNCKVEE

EEEEDTKSYELVDPSETTLKLLLDFPPVVNDMGFFQGPRDNLSIYLQN

>itbMYB16-like4

MGRSPCCEKVGLKKGPWTPDEDKQLLAYIEQYGHGSWRALPAKAGLQRCGKSCRLRWSNYLRPGIKRGNFSLQEEQSIIQ

LHALLGNRWSAIASHLPKRTDNEIKNYWNTHLKKKLSKMGIDPMTHRPKINSSFGSAANLSHLAQWETARLEAEARLVRP

SKFIASSLISPHHKPPPPPLKVPPCLDMLKAWQETWTKPPRTRVSSNLDGGAFVSNATPHQFPTTLNFSDQKLTTLNFSN

QNLTTLNFSDQNLCFMGTPYVHESNIGNPNPTGNDIIPHVAMDPLSELPTFIHGFSELSPETLTGYFADDNVVGNYGTAD

LEDNSRYWNSILNNLVASPVGSPVF

>itbMYB16-like5

MGRSPCCDKVGLKKGPWTPEEDQKLLAYIEQHGHGSWRALPSKAGLQRCGKSCRLRWTNYLRPDIKRGKFSLQEEQTIIQ

LHALLGNRWSAIATHLPKRTDNEIKNYWNTHLKKRLAKMGIDPVTHKPKNDALLSSDGQSKSAANLSHMAQWESARLEAE

ARLVRQSKLRSVSLQNPLEPSSPLTKPEVPAAGSPRCLDILKAWGGGGAGGSGGGAAFSVAGLGVGVEALESPTSTLSYS

ENAPQISSSTAAFMKEESEDQAWKCFGNAAEHFKHGGGVEDSAAAAGFASAGLLGLTNIPAAMESGAWTTQEHIPTGNFV

ERFTDLLLSANSTERSLSEAGSTESNNAAGDGGSGNHYYEDNKNYWDSILNLVNSSPSNSPMF

>itbMYB61-like6

MGRHSCCYKQKLRKGLWSPEEDEKLINHIEKFGHGCWSSVPKLAGLERCGKSCRLRWINYLRPDLKRGTFSQEEENLIIE

LHALLGNKWSQIAARLPGRTDNEIKNLWNSSIKKKLRQKGIDPNTHKPLSQVENAEKAQVFNNNSGFLSSNRNNLVEFDN

PKSSQMISGFNPNPNSNTILDHHCSSSIKLQGSPVFFNSSGFPWLVSGSEKSEKLTDPEDIKWSEYLLGNSIPSQPEPQF

TVTEEGSFSTTASTWLQNQHPSLQAANLCTASKHFQRPSAASTQFS

>itbMYB16-like6

MGSSPCFENVGLKKGPWTPDEDQKLVAYVQQYGHGSWLALPSKAGLNRCGKSCRMRWTNYLRPDIKRGKFSPQEEQTIIQ

LHALLGNRWSAIAANLPKRTDNEIKNYWNSHLKKRLSEMGIDPITHKPKSNAFGSKEAANLRHMAQWETARLEAEARLVR

HNSTMFGSSLSSPSPPHHVSLLHKAPRNPPPPPTVPPPLNVLKAWQAAWTTEPPMTAPNPRISLSPPNVDDGDMFVSAAA

LDQSPTTLNFSDQNLVAFPAVGFGDDVLCYLEGSTSNMNTLRNPNTTGDGIIGPAMDPLSGFPTFIVPENLTAHCSTGYL

DSVIGNCGAGW

>itbMYB61-like2

MGRHSCCYKQKLRKGLWSPEEDEKLINHIEKFGHGCWSSVPKLAGLERCGKSCRLRWINYLRPDLKRGTFSQEEENLIIE

LHALLGNKWSQIAARLPGRTDNEIKNLWNSSIKKKLRQKGIDPNTHKPLSQVENAEKAQVFNNNSGFLSSNRNNLVEFDN

PKSSQMISGFNPNPNSNTILDHHCSSSIKLQGSPVFFNSSGFPWLVSGSEKSEKLTDPEDIKWSEYLLGNSIPSQPEPQF

TVTEEGSFSTTASTWLQNQHPSLQAANLCTASGGQ

>itbMYB16-like7

MRMGRSPCCEKVGLKKGPWTPEEDKQLLAYIEQYGYGSWRDLPAKAGLQRCGKSCRLRWSNYLRPGIKRGNFSLQEEQSI

IQLHALLGNRWSAIASHLPKRTDNEIKNYWNTHLKKKLSKMGIDPMTHRPKINSSFGSAANLSHMAQWEIARLEAEARLV

RHSKFISSSLISPHHFRLPTNNPPPPPQVPPSLDVLKAWQEMWTKPPRTRVSSLVDDGAFVSNATLHQSPTSLNFSDQNL

CFMETPYVRESTSNIGNPNPTGIIPHVALDPLSELPTFIHGFSELSPETLTGYLDDNSRYWNSILNNLVVSPVGSPVF

>itbMYB16-like8

MGRSPCCQKVGLKKGPWTPDEDKQLLAYIEQYGHGSWRDLPAKAGLQRCGKSCRLRWTNYLRPDIKRGNISLQEEQSIIQ

LHALLGNRWSAIAAHLPKRTDNEIKNYWNTHLKKKLCKMGIDPMTHRPKINSSFGSAANLSHMAQWETARLEAEARLVRH

SKFISSSLISPLHFRNNPPPPPKVPPSLDVLKAWQETWTKPPRTRVSSDGAFLSNATTHQSPTTFNFSDQNLTTLNFSDQ

NLCYMETPYVHESTLNIGNPNTNGNNIIPHAMDPLSDLPTFIHGFSELTPEILTGYLDDHNVAGNCGTADMEDNSRYWNS

ILNNLVMSPVGSPVF

>itbMYB41-like6

MGRYPCCKESSELKKGPWSTDEDKKLADYIHENGIGNWQMVPKGAGLNRCGKSCRLRWTNYLRPDIKRGCFSEEEEDMII

QLHKQLGNRWSKIAAKLPAGRTDNEVKNYYNTHIKKKLMRLGIDPITHKPLPPNLNHLLNNLPHNYYNSFNNNPIISPLE

SILRLQANLTQMANAQLLQNIAQFLNNNNNNIPLPLAQNNNNVPMLFNNNVTTFDNFNVESNESPLLFDHPLSTTPNSYS

IESVLDQPSPMSNSLGFVPGNCDLVPSVLTSNNILSLPPLVSATPETSNSDQMFPTGAMQCGGGNDDDFSALEKIFCDEG

NSSLLESIFQ

>itbMYB15-like2

MVRAPSVDKNGLKKGAWSEEEDDKLRAYVLRYGHWNWRQLPKFAGLSRCGKSCRLRWMNYLKPGIKRGSFSKDEDEMIIK

LHKELGNKWSAIAGKLPGRSDNEIKNHWHAHLKKHLQTKQDPKIIRSEQKINETAEYETFQKVKIAEDESSIYDAISSYS

QGASSSEESSCLSSNSKSSDLISCNTVWTVPEEGMMMTSSQSFEETFFWDDLLFADASFSSSESEGGFMSPTSMVEEEFT

LPYSLFGEDDVNFLNNFM

>itbMYB17-like3

MGRTPCCDKKGLKKGPWTPEEDEKLIDFIKKNGQGSWRSLPKLAGLLRCGKSCRLRWTNYLRPDIKRGPFSSEEEKLVIQ

LHGILGNRWAAIASQLPGRTDNEIKNLWNTHLKKRLLSMGIDPQTHEPSSESNGPQRRLPTTPSARHMAQWESARLEAEA

RLSRESKLLVPSTIGGSEADFFLRIWNSEIGEAFRKLKKGVKFACQSPISQASSSTKYGSASGTTTEMDITLAGSADAGG

NPNEDPEGKSNRSYTDDPLQGSETSCSNELEDSSESALQLLLDFPTYNDMSFLGHNDTYSIYPAFLTESTLNCSSAEH

>itbMYB61-like3

MGRHSCCYKQKLRKGLWSPEEDEKLIKHINKYGHGCWSSVPKLAGLQRCGKSCRLRWINYLRPDLKRGTFSQEEESLIVE

LHAVLGNKWSQIAARLPGRTDNEIKNLWNSSIKKKLRQKGIDPNTHKPLSELENDQENAGKKNGIASQESGELSLMESEH

NNLISPPEIAPDNNKLSAMPIDQFSAPPQQSTQEFFLNNSPKIPDLTGYLCFQQMNNNNNIGLSMNSNNSNLFFNSNNNN

VVPGSVLPGPSISPNNNCSSFFEANNGGLAWDCGKAEKESEEIKWSDYLQTPFLLGNTIHNIQAPHHHHDLFNETKPETQ

FASPQPSLPATWLQNDQQPSLYNNTKHFQRLSAAFGQFS

>itbMYB306-like

MGRPPCCDKVGVKKGPWTPEEDIILVSYIQEHGPGNWRAVPTNTGLLRCSKSCRLRWTNYLRPGIKRGNFTEQEEKMIIH

LQALLGNRWAAIASYLPQRTDNDIKNYWNTHLKKKLKKMQGEDGAESSGGQDGNSSSSSHHSISKGQWERRLQTDIHLAK

QALCEALSMDKSKPVLSAGGSSNPATVPVQTGPYASSAENIARLLESWVKNNGPTRSNSETTVTQTGFAGSTSSPSEATF

DHSLFSFNSTCNNSEALSAVESKPVFARAAVAPPTFLTQPKPNNYNIAPPETQMPLTLLENWLFDDAANVQPQEGIMGMP

VPLAGTAELF

>itbMYB306-like2

MGRPPCCDKVGVKKGPWTPEEDIILVSYIQEHGPGNWRAVPTNTGLLRCSKSCRLRWTNYLRPGIKRGNFTDQEEKMIVH

LQALLGNRWAAIASYLPQRTDNDIKNYWNTHLKKKLKKVEGSSEDGQDGNSSSSSHQSISKGQWEKRLQTDIHMAKQALC

DALSIHKPATPEPFQPVHALNPPVQPSSGSTYASSTENIARLLQTWTKPVQSRSNSETTIQSSLNNNPSLGPGSSSSPSE

GTALSSAAFDQTVFGFNSNMDENNPWVFPIESKPQSVAPQNGNTSNNLGTQLPLTFLEKWLLDDANNVPAPDDLMDMGMG

IDFF

>itbMYB61-like4

MGRHSCCYKQKLRKGLWSPEEDEKLIKHITTYGHGCWSSVPKLAGLQRCGKSCRLRWINYLRPDLKRGTFSQEEENLIIE

LHAVLGNKWSQIAARLPGRTDNEIKNLWNSSIKKKLRQKGIDPNTHKPLSEVENEEKLASLTNNNEKLTLSEGSSELNFV

EAESCNNNNNNNQVPSLTVMMDSYPMIDNNVVCSNPMSSNTPPTHEFFLNKTPDLSGYLSFFNNNIGSSFPMQYPSNSSL

FLNPKSNVVLPMETNSSFFENNNASFPWGPATDHHHHCGGKPEKQSADLDETKWSEYLQAPFYLQPPPPPNHQDLYAQGK

PETQFATQGGSPLIPTTTSAATATATTTWLQNQAMYNNSKNFQRLHAAFGQFS

>itbMYB330-like2

MGHHSCCNKQKVKRGLWSPEEDEKLINYISTYGHGCWSSVPRLAGLQRCGKSCRLRWINYLRPDLKRGSFSPQEAALIIE

LHLILGNRWAQIAKYLPGRTDNEVKNFWNSSIKKKIISRACFSDHLSAAISSNIPADPNTAPFDHQTLYSLNPNYNNLIL

HTTHHHQVLQDQAAALISTAGTTSSSSPTSYLQAAAHQMIDQNGNLVLPMMPSDPAWFLGQQPQNLEHNFSIFSAAANDH

IAPDFDMMVFPAMPKLCEMIKAGGEGENIPISSSSSSSSSAVVAAAGCGGSDLVVPGSSLSCYPSGYNARDLHVPAGYEM

EQTDTIILPSFPLPPPPPPSLSSLSPPSSFSGQSGNQLITYQS

>itbMYB26

MGHHNCCNKQKVKRGLWSPEEDEKLINYISNYGHGCWSTLPKLAGLQRCGKSCRLRWINYLRPDLKRGSFSPQEAALIIE

LHRILGNRWAQIAKHLPGRTDNEVKNFWNSSIKKKLISHGGRLSHHLPSSPAPILSNVSSNPNFQTFYSQLPSSNNPNFI

QNNNNVVLNVPAQLQAEDQMNNLVFPVMPSVPLPQLETTSIDPTWFLGYPQPQNLIDHHHYQISSYNNNNFDNEMMVPLL

HHEDEPIIIPKLLPGNLVLSSSSLSSGIAQQDDFIVPNPIPYSHHDLQIPTYEIMPSMLPPMPALSLSSSSSSSSFSAVP

CSQILMNPSTWVP

>itbMYB4-like3

MVNLKKGAWSPQEDRRLISYITKYGIWNWSQMPKFAGLARTGKSCRLRWINYLRPDVKRGPFSVEEVEVIVRMYLSLGNR

WSAMAAQLPGRTDNDIKNFYHTHLKKNLEAVSVPVSRRAAENGRKRTNKSKNNNNAPPPVMVQKHPQVVAEKPRHDDDQV

LLMNSSPGGFGNPNVAHRQQEVHRRFSSLSSEDYDEDRSFWYNVLKEADDLKF

>itbMYB61-like5

MGRHSCMVKQKLRKGLWSPEEDEKLYNYITNFGVGCWSSVPKHAGLQRCGKSCRLRWINYLRPDLKRGMFSQEEEDLILS

LHEVLGNRWAQIAAKLPGRTDNEIKNFWNSCLKKKLIKQGIDPNTHKPMAETQDDPKNPTLPTPLNELPTNFPTTPQMEP

SKLPFITTKQIFDPLFLYEPQENLNFPSTYLNPTYAFTSLPGLMNFDTNGQITETDYFSDGSNSRMGSSNSSNNIGAAQM

NNNMENGSRFSWEVGNRMESLFDQYGFSNNGEMIIKPEEEEEERQLIEAHCHDYTLTSLPQDLGGANLDVFHQL

>itbMYB30-like2

MVRAPCCDKIGVKKGPWSPEEDQILISYIQRNGHANWRALPKQAGLLRCGKSCRLRWINYLSPEIKRGNFTKEEEDAIIH

LHETLGNRWSAIAARLPGRTDNEIKNVWHTHLKKRLSNYEPPMKPIKHPKTTTATSEKHSNIIQHATGPTSPHQTSSDDM

SSSSSATDSSAMTDTITEHPAMIKLENADSAEGFVEIDESFWTDVPSFGSPVSSLENFMEQSGGDMNMNMVEDDRDFWYN

LFVRAGEISELAEF

>itbMYB15-like3

MVRAPSVDQNGKKKGAWSKEEDDTLRAYVLKHGHQNWRQLPNLAGLSRCGKSCRLRWVNYLKPGIKRGNFTRDEDQLILN

LHKQLGNKWSAIAARLPGRSDNEIKNHWHTCLKKFDNVGVIESSKTQSSHSDDAQSVQSSDKSQQQHLAPFDLQAAMETL

PFSPKTEPFSYFMDSAVLSSEEGTSNDDDDDDSRELEQEIFVPDRLFVDEEIQSLLNDFP

>itbMYB80

MGRIPCCEKENVKRGQWTPEEDHKLSSYIAQHGTRNWRLIPKHAGLQRCGKSCRLRWTNYLRPDLKHGQFSEAEEQTIVT

LHSVLGNRWSVIAAQLPGRTDNDVKNHWNTKLKKKLSGMGIDPVTHKPFSHLITEIATTLAPPQVPHLAEAALGCFKDEM

LHLLTKKRIGFQLHHPVGPVKHEDKDETIEKIKYGLSRAIKDPAVEMLPAGGNNKPWDHAGGATSSNLGETSNGFPTSDH

HHAGFHYSLPCLMHDGGEDGSPWNQSMCTGSTCTPAEQQGRHVHDKAVEDENGGECSEGGGKRTTATDAPPPSIFNSDCV

LWDISSEDLINPMV

>itbMYB14-like

MGRAPCCDKTAVKKGPWSKEEDQILINYINKYGHGNWRQIPKNAGLVRCGKSCRLRWMNYLRPGIKRGHFTHEEEFIVVK

LHKTFGNRWASIAARLPGRTDNEIKNIWHTRLKKRLHDFDIPPDNQVVGNTLENDGASNFAAEDNDPSGGLAGVTQSDFP

LGDDDQFYKSGFEFNGADFTPTMDNDDVGGGVDPGSQQFWQDQLLTWTGDELLDLWK

>itbMYB308-like6

MLRSTLESGDRIIECGRAYMGHHCCSKQKVKRGLWSPEEDEKLIQHITSHGHGCWSAVPKLAGLQRCGKSCRLRWINYLR

PDLKRGSFTEQEERTIIDVHRILGNRWAQIAKHLPGRTDNEVKNFWNSCIKKKLIAQGLDPNTHNLLSPNQSKNNIHKPP

NKNSYHHHHQLSPSTFTIDTTAPHTIKEVIPMGMKAALSSITPPFHQPVDYTNSMIPTSNDQNLDYQNPTTIMDFGSCSS

SMESTTSSLIAAAGFGIMAGVFGTDTTFEAAAQGFEPLRTQEERQEQEEQVYKVINGEDHHHDEFNNGGQNMFDAASNFD

FEFMDAALMPCGVYSNVNPIDQLAWDC

>itbMYB308-like7

MGRPPCCDKQGVKKGPWTPEEDIMLVSYVQQHGPANWRTVPAHTGLRRCSKSCRLRWTNYLRPGIKRGCFTDQEEKMIIQ

LQALLGNKWAAIASYLPERTDNDIKNYWNTHLKKKLKLVESGGLMIDSSSRFSSSSSSSHSSSSRGQWERTLQADINTAK

QALNDALSLEKSTRHRPLDFIPQPPAPVQYASSADNIARLLKGWMESPAKSYSTPSTSNSNGGAATVTDTSSSSSDDAPG

GICLSEAFESLFGFDSDQFSQSYESKPQVPLSVMLENWLLDDDI

>itbMYB20-like1

MGRKPCCDKVGLKKGPWTTEEDKKLINFILKNGQCCWRAVPKLAGLLRCGKSCRLRWTNYLRPDLKRGLLSDYEEKMVID

LHAQLGNRWSKIASYLPGRTDNEIKNHWNTHIKKKLKKMGIDPVTHRPLSSATIDDVNKETAKQRESGQEMDQREVVETP

LMDQSTITDAFEEDNKNMETSPTLVDPLHQVNNNNGFFSSPDNNEILATSLSSSSSSSTSSLNSHGGFVSSVEDNNNNNN

NVGSLFEDLDFLPGFDWHNDYINDVGLWVEDDDFFKELCFVDMFSH

>itbMYB35

MMGRPPCCDKANVKRGPWTPEEDAKILAYVASHGIGNWTLVPQKAGLNRCGKSCRLRWTNYLRPDLKHDNFTPEEEACIL

ELHKTIGSRWSLIAKHLPGRTDNDVKNYWNTKLKKKLKNMGIDPLTHKPFAQVFAEFGKLSGLPSPSNQNALLKNTIKNE

AVFEPEPRSFPTNVQNSRFVSPEMNKHLQIQNSPLVHNFPREPIQPPHSSPDTSFPHFASSPYCSSSYEQPLSQFLTSSS

STPWNEFILQDPDTELPRQDSKFPGTFSLDDPMTPSVQGEAGPICGFTNEGVNNLDEDITEGTRGEASSSMAEDSFVENI

LARDRQMQLEYPQLLDGYFD

>itbMYB330-like3

MGHRCCSKQRVKRGLWSPEEDEKLIKHITTFGHGCWSTVPKLAGLQRCGKSCRLRWINYLRPDLRRGSFSEQEERTIIDV

HRIIGNRWAQIAKHLPGRTDNEVKNFWNSCIKKKLIAQGLDPNTHNLISPSQAKTTKHNNSHTRHQKSSSSAFTIETCSS

SHKDVVPIEIKATLAAAFPPFPPNTTPPNDKLLYHKNTITLSHERNNPVLDYASCSSMEITSNHNNLSSSSSSNLSGFGI

LNSNNCMWGGTTNLELLFGGSTAAGRQEEEEEEEEEEEEEDHIQEEKEVGTETQTGANQVYKLNIDDEFNNNNGQSIQIN

LFDNSNIGIDFVESSLMPCAMYCNANSMDQLPWDC

>itbMYB86-like

MGRHSCMVMKQKLRKGLWSPEEDEKLYNYVIKFGVGCWSSVAKHAGLQRCGKSCRLRWINYLRPDLKRGLFSPEEEHIIL

VLHEVLGNKWAQIAAKLPGRTDNEIKNFWNSCLKKKLIKRGIDPNTHKPIMNTSSDAPKSFAEDHNNINNSSSTIPKPTL

PWPLNLSELEQPQAFDPLFLYDFQQSLNNNSEQYFHGFSTLPSLANFENINMVNNGPITFPNWETCDNNINTDSVSAHQF

PFSAKSSGDVEGCSWQDDFSEYSLPALPQDLSGENNLEFFMSCTKFNL

>itbMYB13-like

MVRGPCVDKNGLKKGAWSEEEDDKLRAYVLRYGHWNWRQLPRFAGLSRCGKSCRLRWLNYLKPGVRRGRFSQEEDEMILE

LHKELGNKWSAIAAKLSGRSDNEIKNHWHTNLKKRLGLALHNKRDPEPSDADQLDLQPSKNSQEPQATQDQKHQINTAII

CDAVQAEISSHSQETSCSTTEGSSTFTSSQSFEEPLESFWNELLFSDPIYGGNGNSYYSSSESEEGLIMSSNSSTVEEDF

TLPYSLFCDDINFLSNFMQ

>itbMYB340-like

MDKQTCCNSSSHDPEIRKGPWTMEEDLILVNYIANHGEGVWNSLARSAGLKRTGKSCRLRWLNYLRPDVRRGNITPEEHL

LIMELHAKWGNRWSKIAKHLPGRTDNEIKNFWRTRIQKHLMKQEAARNESTVISGHGSSCEKTDQASSTTSASQLSSIGQ

GDIYSSPQSSFACNIEIDTGFQGHHFPSHEYSNDAVWSMDDISWSMQILNGD

>itbMYB6-like2

MGHHCCGKHKVKRGLWSPEEDEKLIKHLATHGHGCWSSVPKLAGLQRCGKSCRLRWINYLRPDLKRGSFTEQEERTIIDV

HRILGNRWAQIAKHLPGRTDNEVKNFWNSCIKKKLLAQGLDPNTHNLLSSNQAKINKNNKPINSQHFTIDQNSSPPKDEP

HIPLDLKASIAAFTCIPNNEPHKYPLKFKYGPTGGYQYQNPSQNKLVTGFGTSCSSVESTSNNLSSSPSSSLNPAPGLGA

MNVENSMWGTGAFEPLFGPDRFVHHQQQQQEMYKVNTEFCSNNVQNMEMVNTFDDSSDFDFDFGDDSALLPFAAVYCDAN

SMDQLAWDNC

>itbMYB58-like

MGKGRAPCCDKSKVKKGPWSPAEDLRLISFIQKHGHPNWRALPKQAGLLRCGKSCRLRWINYLRPDVKRGNFTPEEEETI

IKLHNSLGNKWSKIAAHFPGRTDNEIKNVWNTHLKKRLTNKKGKGRSNNTDDDEDDDESPKNDAVSPDTPPPKDASLDAS

PTSSSSSSSTKHDQATAEAPPEPPTPGDHDSVIDMEIWDMLDTLDNPSPQDPEIVVADLQATGSSCGGEIDKQVEWLRYL

ENELGLEDSPNNNNHAQPQKDDDDSNHNLVHLDSFDDNDDFNDMVASLYFPTWPPCSPQHFGI

>itbMYB83-like1

MRKPEFSSGKNGANVGNSNINSNNNNNSGSNNVVVANKLRKGLWSPEEDEKLMHYMLSNGQGCWSEVARNAGLQRCGKSC

RLRWINYLRPDLKRGAFSPQEEELIIHLHSLLGNRWSQIAARLPGRTDNEIKNFWNSTLKKRMKNTSSSPSTTTSPNTSD

SSSLEPCRDNIMGGLISSSSMQDHHHHHALMSSMYMHDPSSSSSSSSSFSSSMPLSAAAAADPLPMLGHSISAAASFMET

GHALGGGGAYYGDHRIFGGNIGAEEGPNHLFMVPPLESVNNAVKSEGFVSRMSIHNTTNPNHNTNNINTNSIDINNHFMN

NNNVKVENVGGGFGSYWDNGGEWDLEELMKDVPNSFPFLDFQVE

>itbMYB83-like2

MRKPDPMGKDGKLGNNNSKAKLRKGLWSPEEDEKLMGYMLRNGQGCWSDIARNAGLQRCGKSCRLRWINYLRPDLKRGAF

SPQEEELIIHLHNILGNRWSQIAARLPGRTDNEIKNFWNSTIKKRLKNANNNQNNSSLSPNTSDSSSENPRAMIGGCCGG

GGAASALILPAMHHHQQPEFMASICMDSSPSSSSSMHTNMLPHFNPFPPPPAGFEAEAAAAGLFGLPPSLAAQLVGVGGP

SAAGECGFLGDYNVVVEPYGVMGLENDLSVPALESISATNNHTNKDSKGENNVVDNHNNNNGNVNNINNVDYYGIFDKKA

NAVNSNNQHLYEEGSIKVEDYMVGFGNHHWSGESLRIGELDWEGLLANVPSLPYLDFQVQ

>itbMYB6-like3

MGHHSCCNQQKVKRGLWSPEEDEKLIRYISTHGYGCWSEVPDKAGLQRCGKSCRLRWINYLRPDIRRGRFTPEEEKLIIN

LHGAVGNRWAHIASHLPGRTDNEIKNYWNSWIKKKLKKPQSTSHHHQTTTTIIGTNSNGTHQQIQFPFNNNPANDQRFFT

QDLATKPLQFQETLFSTPNIITTIAPFSTFDPLFQAGQGDCSANRVDPLFQDSNEFWQQQIQSTTSFGSGMGSGYLLPPL

MDDLGPLDVPPPCSLFDAARDNHASEWGVDTTQQQQQRQQQQQCPGSNYLLWDEVTEGNLGGEEEELIGVGVPTTSPMAA

MLSSFPPSL

>itbMYB60-like

MGRPPCCDKIGIKKGPWTPEEDIILVSYIQEHGPGNWRSVPTNTGLLRCSKSCRLRWTNYLRPGIKRGNFTPHEEGMIIH

LQALLGNKWAAIASYLPQRTDNDIKNYWNTHLKKKLKKLQSPGSENTAQMGSENSPTSATSYHLVSKNSFTDRPQIATSS

SSLYASSAENISRLLEGWMRASSSSSSSKGFTTNSHTNNTSHGGDLEEEDEDLTRPEVAATDNNGVIPKEEYLDSILTFG

SLMNGMESESCSGNNNKQKVNEGNDSDNPPPLSFLEKWLLDESAAQVEEEEGEDNQGAVMELPTIFS

>itbMYB4-like4

MVRTPCCDVSGLKKGTWTVEEDKKLAAYISQYGCWNWRQLPKYAGLSRCGKSCRLRWMNYLRPNIKRGNYTQEEDQLILK

MHRQLGNKWSAIAAHLPGRTDNEIKNHWHTSLKKLTEQGYASSSSSPTQQPRKKPSAGRTKRSRKQQPIPSASAPYSSSS

NVSAHEILESSQWSSSQQAFSSSSPSSSSPSIGTSSPNAGNTPEACADQNQTPPQIVGSTSEDESFWNEPFLLDNAFASG

DDFLDYRPASPFSQYGQFSSSCNLFDELVNELMDYL

>itbMYB24-like

MVGASEKVGWRKGPWTPEEDKLLGDYVSLYGEGRWSSVARCAGLNRNGKSCRLRWVNYLRPGLKRGHISPQEEGIIIELH

ALWGNKWSTIARYLPGRTDNEIKNYWRTHFKKKPASGKTSEKQDRRKNRRKRNEEKVIKDTKPQETMSNNDSSCITAAAA

PIGGTSANTTTSLYHEDIESWVDSFAMDMDGLWGGGLWNLDNDDSLNLHDPEAALLEQGHVIQNPCGFGADHAVNLWNGG

FIF

>itbMYB61-like7

MGHHSCCNQQKVKRGLWSPEEDEKLIRYITNHGYGCWSEVPEKAGLQRCGKSCRLRWINYLRPDIRRGRFSPEEEKLIIS

LHGVVGNRWAHIASHLPGRTDNEIKNYWNSWIKKKLKKPLLSTSHTNSTTSPTTNTTNNHRVLQPQTSSPFSFINTNCNN

QLDFFNTQDFTTKPPLQLRQTLFSFPNTNPNPFFPFDANAALEGVNGDYTDRAAAVDQPFQDSPAFWQQELQAAAVFSMG

MDSGYLPPLMENIMAPPPPSVELPAPPCNVALARESDHVSEWAAVDTQQCPGSFLFWDQTDGEELALPTTSNIGAILSSS

FPSSI

>itbMYB62-like

MMVSKSGEIQSLSNNNEERFGELRRGPWTLEEDTLLIKYIAAHGEGRWNALAKCAGLRRTGKSCRLRWLNYLKPDIKRGN

LTPQEQILILELHSKWGNRWSKIAQHLPGRTDNEIKNYWRTRVQKQARQLKIDSNSKKFIEAVKRFWMPRLIEKMEQHQT

STLSSSFISSPSSSSSISTMEKQSPLNSLPSPITIPEPPKTDNPENKNLNICSTDSFQLHDDDDCYHVEIMGNSCYAGEE

EGFGHPAMSAFESRDISMLECQLAPDDWFGNDVADSLWNIDETWQYRKLEDFGDLS

>itbMYB30-like3

MVKTPSVDKNGIKKGLWSKEEDDKLKTFIESHGHKNWRQLPKIAGLSRCGKSCRLRWMNYLRPGLKKGAFSVQEDEIIVR

LHNKLGNKWSAMAELLPGRSDNEIKNHWHTHLKNRAKQTQKPEESVEELLPPSAETSEFSDFEFKPPQDDIFNILNLDEV

ALPQNSQTVNIPLSQEVSAASSGGGLTSSFSSSSSSISDWILDDISTISLESFMDPLESFWTEPFVADTSYPKNVGYPVT

LFEGENFPVQTSPLEGNFVPPYFEDAAWY

>itbMYB4-like5

MVRTPCCDKSGFKKGTWTPEEDRKLAAYVTRYGCWNWRQLPKYAGLARCGKSCRLRWMNYLRPNIKRGNYTKEEDEIIMR

MHEQLGNKWSAIAAHLPGRTDNEIKNHWHTSLKKNSRQLSSSPAQPKKKSSSSRTKRQDRANSFPENPIISAHEILESSQ

WSPQQSSSEACSSSPSSSSTTTTTTKSDEIIHDDQTVQGEFGHMECDESFWSEPFIMDSLWSKNEFDVPSIDFGLLSPPS

PFRDYDFLCSFDHFPEGVNNLNW

>itbMYB305-like1

MDKKPCNSQDVEIRKGPWTMEEDLILINYIANHGEGVWNSLARSAGLKRTGKSCRLRWLNYLRPDVRRGNITPEEQLLIM

ELHAKWGNRWSKIAKHLPGRTDNEIKNYWRTRIQKHMKPQQGGENAATRQSSSSCCGGEQNEQASTSQASAPDTVETYSP

TSYNNMDATFQGTFPTESNVDNMWSMEDLWSMHLLNGD

>itbMYB20-like2

MGRQPCCDKVGLKKGPWTAEEDKKLITFILNNGQCCWRAVPKLAGLLRCGKSCRLRWINYLRPDLKRGLLSEFEEKMVID

LHAQLGNRWSKIASHLPGRTDNEIKNHWNTHIKKKLRKMGIDPVTHKPLSATDHEEEDEDDDQKTNKTKPQTENGTSADQ

ESPVPGTSNDQSSITDLMVEEDNKSMETSQQSPVFESSIMEVSNGFCTDEVPLIEPHEILVPTCSSSSSDNYTAAGGDFV

SSNNVNINNNNNNNINNVVEDMEFLPSLDWQCDSIDDMGFWGDDFITTTLDSLFNSDSNNFIINNNNNNLNQVPLMVTDE

ESWKLDQLL

>itbMYB36-like1

MGRSPCCDKNNVKKGPWSAEEDAKLKAFIEQHGTGGNWIALPQKIGLKRCGKSCRLRWLNYLRPNLKHGGFSEEEDTIIC

SLYISIGSRWSIIAAQLPGRTDNDIKNYWNTKLKKKLFGKQVNDHGHSLIRANQRSNLQPEMVSCGPTSSPNNINIYQNP

SYPELVSEPVLYPKEESGFKNPAHLTDTLPIVNSILSSAGEYTMEDCLINQMVYNVNPQNLEGVEFLCDNIFYNWKDGSS

SSSAGGLDWNEINEIQSLVPSPPSPLASSYEGLEPAGNSVQLPAFG

>itbMYB17-like4

MGRTPCCNKEGLRKGAWTAEEDKILVAYITKNGHGLLRCGKSCRLRWTNYLRPGIKRGQFSSEEVDTIIQLHTVLGNKWS

AIASHLPGRTDNDIKNFWNSHLRKQGSDPNHQNHQMAHPHGNIDEKVETTLSTNHIVQWDSVSVAPSLLDLPSASKMDHH

DPFLRLWNSEVGEAFCSFKKPRGVPCQSPVSSSSKFESSSGITLHSEPASKLLSSADTVEGVPMNCKVEEEEEEDTKSYE

LVDPSETTLKLLLDFPPVVNDMGFFQGPRDNLSIYLQN

>itbMYB305-like2

MMGTEQMGWGIMGGWRKGPWTAEEDRLLVEYVRFHGEGRWNSVARFAGLKRNGKSCRLRWVNYLRPDLKRGQITPHEERI

IVELHARWGNRWSTIARSLPGRTDNEIKNYWRTHFKKKAAANSSDHPKARILRQQQQQKKKKQEESNHQMDMRKMMSSFD

EIDENRLSVPQPQPPTDLLSDQEQGFLYSMINGYASVPEPYSTEDMIMCDGLWNLDDFHGSFSANKACFLQPVAAPFY

>itbMYB36-like2

MGRAPCCDKTKVKRGPWSPEEDTTLKSYLQKHGTGGNWIALPQKAGLKRCGKSCRLRWLNYLRPDIKHGGFTDEEDQIIL

TLYTNIGSRWSVIASHLPGRTDNDVKNYWNTKLKKKLAAAVAATNKPDVFPQTVPTNFNPTTYYAVQEMVGSQPHQFPLP

NLMEVQENCTTSTANNSSYNPQVSVGCGGYSTSFAYNNNENSFGTSWFGNGGIEEDVFRMDMMSSSTGLSSSSSPSSSSP

SCFDHLMMNYGFDFQDNFAPPNITHNFTNHPSNGFQY

>itbMYB20-like3

MGRQPCCDKVGLKRGPWTIEEDHKLMTFILNNGIQCWRLVPKLAGLMRCGKSCRLRWINYLRPDLKRGALTEAEEEMIIK

LHSQLGNRWSKIAAHFPGRTDNEIKNHWNTRIKKKLKLRGLDPTTHKPLDQPGSSIAKINGVDDHNQQQSSSSGEEEMMM

IMKNKDYPIPNYEYALQQTLNSSLDESLELESNIIPAGSEDMDTLSMDMYNPDDQRQEPYQTWIGSPLQWDLFNNLDGNF

L

>InMYB1

MVNSSARWSPRVRKGAWSEEEDDLLRKCIQKFGEGKWHLVPFRAGLNRCRKSCRLRWLNYLHPDIKRGHFSLEEADLILR

LHKLLGNRWSLIAGRIPGRTANDVKNYWHSHLKKKVVGMHMASSNSSRQDNNWDDEKGKAPQIKENILFRPRPRRFFRTS

LSSPALSTLTGKAKAVAYDAPPPPHHHQLQAQPEATSPPADLLMVFNVQQNNNSMATNFPAQTTAPPSHDGVKWWDLLYD

DDHQGLIDWTTDDDFPIDVDLLKLLDTTI

>InMYB2

MVNSSSAWPPPSSSRLMRKGAWTEEEDNLLRKCIQKYGEGKWHLVPLRAGLNRCRKSCRLRWLNYLRPDIKRGDFSVDEV

DLIMRLHRLLGNRWSLIAGRIPGRTANDVKNYWNTHIQKKVFAMARMQDNWKGKAPEIRENTVVRPRPRRFLNTSLSPTS

KTGKATAVTYDAQIQGHTLPQPPEAIITTSDLVMENVQLNNTIATLPSELETTTSDDRVRWWEDLLFDKEFNDDEGNACM

HEGQVGWTNLPIDMDLLELLS

>InMYB3

MANSSAWSGVRKGAWSEEEDNLLRKCIQEYGEGKWHLIPIRAGLNRCRKSCRLRWLNYLRPDIKRGDFKLDEVDLIMRLH

KLLGNRWSLIAGRIPGRTANDVKNYWNTHIQKKVFAMASSMQDNWKGKAPEMRENNVVRPRPRRLFLNTTSSLLSGTPPL

TGKATAVTFDAQIQGHNKIPQPEATSELVTKNLQENNTIITASELETTTSNDRVQWWEDFLFDNAGSTCMNQGQVDWPNF

PTDMDLSELLS

>InMYB75-like1

MVNSSSAWPPPSSSRLMRKGAWTEEEDNLLRKCIQKYGEGKWHLVPLRAGLNRCRKSCRLRWLNYLRPDIKRGDFSVDEV

DLIMRLHRLLGNRWSLIAGRIPGRTANDVKNYWNTHIQKKVFAMARMQDNWKGKAPEIRENTVVRPRPRRFLNTSLSPTS

KTGKATAVTYDAQIQGHTLPQPPEAIITTSDLVMENVQLNNTIATLPSELETTTSDDRVRWWEDLLFDKEFNDDEGNACM

HEGQVGWTNLPIDMDLLELLS

>InMYB75-like2

MVISSVLSGSSSRVRKGAWSEEEDHLLRECIQKYGEGKWHLIPLRAGLNRCRKSCRLRWLNYLHPDIKRGDFSPDEVDLI

LRLHKLLGNRWTLIAGRIPGRTANDVKNFWNTHLQKKVSAMAMASPRQDNWKGKAPEITENTVVRPRPRRFLNAASSSPT

TLFTGNATTVAYDGQLQGHNITTQPETTSDLLMENVQQNNSTTTLPSVLLETTPHDKVDNVTWWEDILSDKELSDEGQFS

WSEFPTDIDLSELLS

>InMYB90-like1

MADSSSEPPSGVMKKGAWTEQEDNLLRKCIHKYGEGKWHLVPVRAGLNRCRKSCRLRWLNYLRPDIKRGDFNLDEIDLIM

RLHKLLGNRWSLIAGRIPGRTANDVKNLWNTRLQKKTIATPSSGQEKWKDKAPKTTEKTVVIRPQPRRFVVTSSSRTLPM

TGKTTIVTSEEVVQLQGHNMPPPPPPPPEAAESTSVPRLMENVDPNNSIIDPPEEAETSDNLAPWLDDFLLDMEFDGDGM

TCMQEGQIEWCDFHIDSDLLDLLS

>InMYB90-like2

MADSSSEPPSGVMKKGAWTEQEDNLLRKCIHKYGEGKWHLVPVRAGLNRCRKSCRLRWLNYLRPDIKRGDFNLDEIDLIM

RLHKLLGNSFVCRWSLIAGRIPGRTANDVKNLWNTRLQKKTIATPSSGQEKWKDKAPKTTEKTVVIRPQPRRFVVTSSSR

TLPMTGKTTIVTSEEVVQLQGHNMPPPPPPPPEAAESTSVPRLMENVDPNNSIIDPPEEAETSDNLAPWLDDFLLDMEFD

GDGMTCMQEGQIEWCDFHIDSDLLDLLS

>InMYB90-like3

MVNSSARWSPRVRKGAWSEEEDDLLRKCIQKFGEGKWHLVPFRAGLNRCRKSCRLRWLNYLHPDIKRGHFSLEEADLILR

LHKLLGNRWSLIAGRIPGRTANDVKNYWHSHLKKKVVGMHMASSNSSRQDNNWDDEKGKAPQIKENILFRPRPRRFFRTS

LSSPALSTLTGKAKAVAYDAPPPPHHHQLQAQPEATSPPADLLMVFNVQQNNNSMATNFPAQTTAPPSHDGVKWWDLLYD

DDHQGLIDWTTDDDFPIDVDLLKLLDTTI

>InMYB6-like1

MRLHKLLGNRWSLIAGRIPGRTANDVKNYWNTHIQKKVFAMASSMQDNWKGKAPEMRENNVVRPRPRRLFLNTTSSLLSG

TPPLTGKATAVTFDAQIQGHNKIPQPEATSELVTKNLQENNTIITASELETTTSNDRVQWWEDFLFDNAGSTCMNQGQVD

WPNFPTDMDLSELLS

>InMYB90-like4

MDKTAAKTGMKKGAWTEQEDNLLRKYIQEFGEGKWHQVPAKAGLNRCRKSCRLRWLNYLRPDIKRGDFAWDEVDLIIRLH

KLLGNRWSLIAGRLPGRTANDVKNYWNTHLKKTAEFGMPSRRQQENGRGNIVIGPPFPPRPPNFSMISSFLSLGTETTTT

TTTLPDNDVTKRTFTPSPPTRAMPAESETHWWENLLAIGDRDGAITWSPLTTSGEGDNAVADGETWSRAIGAGNSMFIEQ

GEFSWNDGIWESN

>InMYB114-like1

MTSRKSGGARMHVHKGAWTAEEDKKLTHYIENHGAKKWKTVAIKSGLNRCGKSCRLRWLNYLRPNIKRGNIAEDEADLIL

RLHKLLGNRWSLIAGRLPGRTDNEIKNYWNTHLSKKVTQLGKSSLPATENQPPKNGMADAEQMGGNKESEEDPELNFDVD

EFFDFSVEGTYGTEWVNKFLEVEKGMP

>InMYB5-like1

MRTQSSTSSEKGAAGASPPAKAKGKQTPCCSKVGLKRGPWTPAEDKLLTDYINKEGEGQWRTLPKMAGLLRCGKSCRLRW

MNYLRPTVKRGHITPDEEDLILRLHRLLGNRWSLIAGRIPGRTDNEIKNYWNTHLTKKLISQGIDPRNHKPLLINPTNNH

PKSHTSSSSVPIIKPTPIHVGLSNQDKTVKMNSSTTVNVVGGTANHQNDQIDQPNSVGGTTTDHQTTGNGDEGFNMDIGD

DNEDNVGMDFCPDEDAFSTFFDSLMNEDVFFAAAQNNQQSNHHDITPLPSTSENNNNNQPLNPFQHMNFPFSTEGWVDDD

DFLP

>InMYB114-like2

MTSRKSGGARMHVHKGAWTAEEDKKLTHYIENHGAKKWKTVAIKSGLNRCGKSCRLRWLNYLRPNIKRGNIAEDEADLIL

RLHKLLGNSRWSLIAGRLPGRTDNEIKNYWNTHLSKKVTQLGKSSLPATENQPPKNGMADAEQMGGNKESEEDPELNFDV

DEFFDFSVEGTYGTEWVNKFLEVEKGMP

>InMYB5-like2

MRTQSSEKGAAAGEAPSKAKGKRTPCCSKVGLKRGPWTPAEDKLLTDYINKEGEGQWRTLPKMAGLLRCGKSCRLRWMNY

LRPTVKRGHITPDEEDLILRLHGLLGNRWSLIAGRIPGRTDNEIKNYWNTHLSKKLISQGIDPRNHKPLPVNPTNSNHPK

NHTPSSSSVPIIKPTPIHVGLSNQDKNVKMNSSTTVNVVGGTTNLQNDQIDQPNSVGGTTENHQPTGNGDEEFNMDLGDD

NEDNVGMDFCPDEDVFSTFFDSLMNEDVFFAAQNNQQPNHHDITPLPSTSENNNNNNQPLNPFQHMNFPFSTEGWVDDDD

DDFLP

>InMYB308-like1

MGRAPCCSKEGLRKGPWSAKEDLLLTNHIQHHGEGQWRSLPKKAGLLRCGKSCRLRWMNYLRPGIKRGNFSQEEEDLIVR

LHSLLGNRWSLIAGRLPGRTDNEIKNYWNTHLLKKLKSAGIEPKPRHSKDSKKKPAKPRPNPQKLTANTKKKHKKARNDE

QSPRQDSDQTATAPEKRTKVYAPKPIRLSPSPAFSRNHSLEDVAGSVSSSSGEVDNKAVVLQGTTAEPPPPPFIPWHLYE

LGGDVDFCDQILDGCDLSSPKCSGPTSDGLLEKVYDEYLHLLSENCFEPLTDDCLCDYPFVDYNVAPTSSNNSSLN

>InMYB4-like2

MARTYLVSALFIGLLLPSPVQSCLERKGAATLSLLTNLWSFLSLSLFLEFGREMGRAPCCSKEGLKKGPWSTKEDLLLTN

YIQQHGEGQWRSLPKKAGLLRCGKSCRLRWMNYLRPGIKRGNFSQEEEDLIVRLHSLLGNRWSLIAGRLPGRTDNEIKNY

WNTHLLKKLKSSGIEPRKIVASKKKATKIVAPKKPGDAKKKPKQQQQQRKESDDNQRYKVYAPKAIRLSSGGVSRNNSVD

DIAGSVSSSSGEVENKAMADGSSSSFIPWNLYELRDDFCAEVLTAAAAGDHLSPQCALPDDCLLDKVYDEYLQLLSENCF

LEDDPFGANL

>InMYB5-like3

MRTQSSEKVAAGASPPAKAKGKRTPCCSKVGLKRGPWTPAEDKLLTDYINKEGEGQWRTLPKMAGLLRCGKSCRLRWMNY

LRPTVKRGHITPDEEDLILRLHRLLGNRWSLIAGRISGRTDNEIKNYWNTHLTKKLISQGIDPRNHKPLLVNPTNSNHPK

NHNPSSSSVPIIKPTPIHVGLSNQDKTVKINSSTTVNVVGGTTNHQIDQPNSVGGTTNHQTTGNGDEEFNMDIGDDNEDN

VGMDFCPDEDAFSTIFDSLMNEDVFFAAAQNNQQSNHHDITPCPQLLKIIIIISL

>InMYB308-like2

MGRSPCCEKAHTNKGAWTKEEDERLIAYIRAHGEGCWRSLPKAAGLLRCGKSCRLRWINYLRPDLKRGNFTEEEDELIIK

LHSLLGNKWSLIAGRLPGRTDNEIKNYWNTHIRRKLLSRGIDPTTHRPMDDPKEKVTTISFGPAQEEEDVDDEKKKMIPQ

DSLPTTTTVKQEYSTSPVRRERCPDLNLELRISPPYQPNNQQPPLTFTAGSAPGSLVNVDNTTVVCFACSLGIQNSKDCT

CTTNANAITSIAAGYDFLGLRNNALLDYRNLEMMN

>InMYB308-like3

MGRSPCCEKAHTNKGAWTKEEDERLIAYIKAHGEGCWRSLPKAAGLLRCGKSCRLRWINYLRPDLKRGNFTHEEDELIIK

LHSLLGNKWSLIAGRLPGRTDNEIKNYWNTHIRRKLLSRGIDPTTHRPINGGAAEAKETTATTISFGAVKPEDAENYSIT

TGKDLGPKKEEKEEETLLFKSEEPQVVEACPDLNLELRISPPSFQETQPPLPLEAAGSGGGGRVNGLCFACILGIPNSID

CTCNNNEDYSSSN

>InMYB6-like2

MVRAPCCEKMGMKKGPWTPEEDQILTSFIQRYGHENWRALPRQAGLLRCGKSCRLRWINYLRPDIKRGNFSKDEEETIIQ

LHQTLGNRWSAIASRLPGRTDNEIKNFWNTHLKKRLQHHGSPYSPNFNVVGNITPIQIGDSSIHLRFPAAPTTVNSMYSS

SPMTTKMEEAEESMQERESYQNLGTTNDDSGIVYLPSSSSVLPMELGGCETSSSIGNDAVFWYNLLINAGNTS

>InMYB308-like4

MGRSPCCEKEHTNKGAWTKEEDERLIRYIKKHGEGCWRTLPKAAGLLRCGKSCRLRWINYLRPDLKRGNFTEEEDELIIN

LHSLLGNKWSLIAARLPGRTDNEIKNYWNTHIKRKLLSRGIDPQTHRAVSSAQNTAAADAASPSAAVVPLALSSSAKNLD

QQQHETAAAGFLPWMRNTKAENSNNTSTTTEDSNSSSGLSSEEIVLHPAAAAAPLINLELSISLPQPPAAPPSTTKLAGK

EFNDQTRKNHLFFSQRAVCLCYNLGFQNSNACNCDKMMTTSSINAEAGMHSFYRPLSL

>InMYB308-like5

MGRSPCCEKEYTNKGAWTKEEDDRLIRYINKHGEGCWRTLPKAAGLLRCGKSCRLRWINYLRPDLKRGNFTEEEDELIIN

LHSLLGNKWSLIAARLPGRTDNEIKNYWNTHIKRKLLSRGIDPQTHRSLITSAKPTVTPQQPSPTTSSGGLSSSSSEETT

YHLNLELSISLPQPADRGGAFQFRNVARGDLSVSQIRVSEWKCM

>InMYB3-like2

MGRAPCCSKDGLKKGPWSTKEDSLLTTYIQHHGEGHWRSLPKNAGLLRCGKSCRLRWMNYLRPGIKRGNFTPEEDDLIVR

LHSLIGNRWSLIAGRLPGRTDNEIKNYWNTHLLKKLKSEGFQPKPRRTSRAIPKNKKTAKPEKTGRDEKRTKRKSGSKPD

AGNRHPDPPEMTTKVKVYAPKPVRVSTGFARNYSFDDLAGASTSSSSGHNNNNNNNNNNNNNNNNNNNNNNNNNTVDGTS

FVPWNIYDYGDDLFKDLMDGCDLSANYSLSDLNDAMLEKVYDEYLELLSEDSYLQTCFPS

>InMYB82-like

MNPLSDFTIKVEFSEDSNPMKAKEVKAGLKRGFWTPEEDLTLKKCVETHGEGNWATISKKSGLMRSGKSCRLRWKNYLRP

NIKRGMMSEDEKDLIIRMHKLLGNRWSLIAGRLPGRTDNEVKNFWNTHLNKRSRRGKRMKITPKDDDSISASIPTQSMEN

PNLVDGSSKQEDEMDSIMNSWMEHMGIENCNINSSISTNNLPWIFEDVPLIPILDDVLLDAFQSTGDETLLDGIHPFLL

>InMYB330-like2

MGRSPCCEKAHTNKGAWTKEEDQLLINYIRLHGEGCWRSLPKPAGLLRCGKSCRLRWINYLRPDLKRGNFTQQEDELIIK

LHSLLGNKWSLIAARLPGRTDNEIKNYWNTQIKRKLISCGIDPQTHRPLDASAAAASCGGATKPENISLDLSSSTPSQEE

TKCSSGTTSEESHHQPLKDKQRNDEQMAGLDLSIGLALHPKTEDSADSTASGGLLLVAPPPPAAVELSVTEAVCLCWQLG

SRSGGLCNKCHATKCFLG

>InMYB34-like1

MGRAPVDKSGLKKGPWTPEEDQKLIHYIQAHGPGNWGALPKNAGLQRCGKSCRLRWSNYLRPDIKRGRFSFDEEETIIQL

HSVLGNKWSAIAARLPGRTDNEIKNYWNTHIRKRLLRMGIDPVTHTPRLDLLDLSSILGSTHQLNFPSLLGLQAILNPEL

LRLAATNLFTSQNDNINPDLLSNPHLQNPQTMLLRKLQESQLLMNAPNFQANSDHHHQFQHPLPPCTTSDNNVSSACSDL

PMQPYSTMNSSSNLQNGHVFQESLVAANGGSLLQTLENYGGGCFESNQSLSNSSNQSGLENFSFDSVLSTPLNSSSTLVN

SGGANTTEDEKESFCSNLMRFDIPDSLDFEDLL

>InMYB308-like6

MGRAPCCSKVGLKKGPWSAKEDSMLTTYIQHHGVGHWRSLPKNAGLLRCGKSCRLRWMNYLRPGIKRGNFTPEEDDLIVR

LHSLIGNRWSLIAGRLPGRTDNEIKNYWNTHLLKKLKSAGFESKPRKTSRTIPKKKKKTAKPDKTRSEKRKKLRHVSKPD

VGNGIQDRPEMIKVYAPRPIRLSTGFARNYSFDDLATSAASSSSGNNKATLNDNGNNNNNGEELTVIPWHLYEVGDDLLD

DYMDGCDLSARYSLPTSDTLMEKVYDEYLQLLSEDCCLQTCSS

>InMYB4-like1

MGRSPCCEKMGLKKGPWTKEEDQILVDYISRHGHGNWRALPKRAGLLRCGKSCRLRWINYLRPDIKRGNFSHEEEDAIIK

LHQALGNRWSVIAARLPGRTDNEIKNIWHTRLKKRLNDYGLVPPQPSLKSKSQPLKPFAMDLQTPSSPPHSSTTTSTDVH

ASSYCSISSHCVVSDAVLLQSDLPEVDESFWSQVFSSENSSDAGDLPATVDGESRFDSTKNETYETNSSVEFWHRLFSKT

ENLPVLPEL

>InMYB330-like1

MGRTPCCSDKDGLKRGPWTAEEDQKLIDYINKHGYGNWRTLPTNAGLQRCGKSCRLRWMNYLRPDIKRGRFSLEEEQIII

QLHSIIGNKWSAIAARLPGRTDNEIKNYWNTHIRKKLLRMGIDPVTHRRRVDLLDLSSILNNNPSLLYNSRILGAQTFAN

PHLLRLAASQHHNNNVINSDTANNVVQDTQQLHAPPPPPLVQDFPVYSPAMAAAQLTQQPNGEFGLENCPANDFWLGTGL

PESELTQDYLLPPLQNYGYYEPAVDPQSAMDPPAPAADESYRFGFRQGWSTPSSSQVNSSSTTTTEDEREISYCSNLWNF

DVANIF

>InMYB6-like3

MGRTPCCEKVGLKRGRWTAEEDQILADYIHANGEGSWRSLPKNAGLLRCGKSCRLRWINYLRSDLKRGKFSPQEEEIIIK

SHAILGNRWSLIAAQLPGRTDNEIKNYWNSHLSRKFYSIIRRAGSDKNIENLETELAKVAEQTKRRPGKVSRSAMKKNKT

TDYKHSSNNINHAPGLQTQKQDTTIPASLYTDDPVNNNNAASSPPILMEKEDTIDTSSFLGSEHFSLDDIMPILMEEMQD

PAGTILSTSSLNSAKEIERDLAKSGDSGVDIYSGLIPSSDHNQFGGENSESTATSSSFPVEHYCSLAQNIIDWDNDWQYC

WDYDSGNNLCNTHNYLMPQQNDDDVMLSSPWPWDDTFYDIVVDNNNNNIAGEEGRVG

>InMYB39-like1

MGRYPCCKESSAELKKGPWSTEEDKKLADYIHENGIGNWQLVPKRAGLNRCGKSCRLRWTNYLRPDIKRGSFSEAEENMI

IQLHKQLGNRWSKIAAKLPAGRTDNEVKNYFNTHIKKKLMRMGIDPITHKPLPNLNHLLNNLPHNYYNSFNNNNPIISPL

ESILRLQANLTQMANAQLLQNIAQILNNNNIPLPLVQNNINNVPLLLNNNNVTTFDNFNVGSNESPLFDHPLSTTPNSYT

IESVLDQPSPMSNSFGFVPGNDDLVPRVLTSNISSLPPLVSATPETSISDQMFPMGAMQCGSGGGDDDDFSAWEKVFCDE

GNSSLLESIFQ

>InMYB76-like

MQVVASTMRRPSSPTLSGSSGGRGDENGGGVKKGPWTPEEDEKLVDYIRKNGHGSWRAVPKLAGLNRCGKSCRLRWTNYL

RPDIKRGKFSEEEEQLIIKLHSVLGNKWSAIAMRLPGRTDNEIKNHWNTHLRKRLLQMGIDPVTHRPRTDFNFIDALANL

PQLLVAAANMGNNSNVANPLWDSINALRLCSDAAQLANELQLLQNFMALQLQLRGSVNNTTNEAQNQIPELATQFGSWNQ

LLDHLALLNPQLQGGLCNPGSSYNFSRLPPNISGSGSVATSSTSQNSEIQIHHPGIISNETNQRQTTNSNVSRINDDSNK

LMTNAFTVSSSSSLNVPSGEDIPSNPIFPALIPASPFPENPSSSIDWETDKEKYTISANLKHDIPNHVPNATTTFEAWRD

IKVDDDEATDSYWQDILYQTFSP

>InMYB39-like2

MGRHPCCKDSEEVKKGPWTAEEDQKLSDYVLKNGHGNWQMLPKKAGLNRCGKSCRLRWTNYLRPDIKRGDFSQEEEQIII

NLHSSLGNKWSRIAAHLPGRTDNEIKNFWNTNLRKKLLRMGIDPKTHQPITDINLLLNLSHQMLSNTNNPLGSALRLQNI

FQFLNSYPILFPTNKQENFPMGLDNNNTIDTINNYVSATHNSSDFIGSDFNPHMKILTDAGENNGHSEYSLPSLVQSSSE

YIPTLDQTIIPSPGGLEANNNEFLAWEEFLKNEEDCSSLWNDILQAKQT

>InMYB3-like

MRKASCDHSHHHHEINKGAWSKQEDQKLVDYIRKHGEGGWRDLPKAAGLLRCSKSCRLRWMNHLKQTAKRGNFGDDEEDL

IIKLHALLGDRWSLIAGRLPGRTDEEVENYWNSHIRKKLVDMGIDPNNHRVSCTYPRPHNSAGVAQTSAGKSRVTSPEKQ

RIESDGEVSDAGSSNVR

>InMYB39-like3

MGRSPCCDENTGLKKGPWTPEEDQKLINYINKNGHGSWRALPKHAGLNRCGKSCRLRWTNYLRPDIKRGKFSQEEEQTIL

NLHAILGNKWSAIATHLPGRTDNEIKNFWNTHLKKKLIQMGYDPMTHRPRTDIFSSLPHLIALANLKELVDQHQSSWEEQ

AAMARLQSEMAKFQYLQYLLQTSPAPAPPPPPPGPGFSPNNLNNTATANNNNNSNNNVSDMEAYSHLLSTVLGSSSATTQ

MDSSVLTLSNLGQAIQDSSIPFSHLPDLQPPCAAIFQQSSKDNNNNNNNMVQAQGISGFTVLSDGENSPTSPWLPSSLSP

SPPPPPVVPEPPTVDGSGASSYGGAPPSSVWPDLLLDDDPLFPDL

>InMYB36-like

MGRSPCCDKNNVKRGPWSAEEDAKLKAFIEQHGTCGNWISLPQKIGLKRCGKSCRLRWLNYLRPNLKHGGFSEEEDKIIC

SLYISIGSRWSIIAAQLPGRTDNDIKNYWNTKLKKKLFGKQVNDHGHSLRANQRSNLQPEMVSCGPASSSPSNINIYQNP

SCHELVPVAMQPVPYPKEEPGFKDPAHLTDTLPVVNSILSSTGMEDCLINQMVYNVNPQNLEGVEFLCDNIFYNWKVGSS

SSTAGGMDWNEINEMQSLVPSPPSPLASSYEGLEPTGKQCAYFKT

>g135

MEKTGNVWTALAHIITAVIGSGVLLLPWSIAQLGWIAGPIIMLCFAFVTLISAFLLCNCYELRDQHGKITYTHGSYLDAV

QSILGMTTVRFRTGGFRFRNRTNRNRPYWYGCSSVSSLPSLYLMNTIAALACHSSDLPPPRSYIYNSLPEIRVLSPHHFS

DSSVIGKDDGRSGVVPKEYPLQCDISKQNLRSNGVRNFASQFREKSSSSIHRRPLDPTATAGGKVLQSARKGNRNAWFCR

FFVSINSIKLGIVYTITFALSISAIRKSNCYHNEGHKAECQYSNTKYMIIFGVVQVLLSQIPDFRSTKWICIVAAFMSLT

YSSIGSGLSLEKVIDNGEIKGGIGGWPSPNAAKKVWPVAQALGNIAFAFPFSIIILEIQDTLKESTEKATMKKASIMAVC

ISTIFYLCCGGFGYAAFGTETPGNLLAGFGFYEPYWLLDFANACIAVQLCGGYQVFSQPWYAITEKWLLKKLPQNHFFVR

DYNLKAFRFSFLRVIFRTTYVAIITGIAILFPYLNQVVGVAGAITFWPIVVYFPVEMYLKQKRIESWTTEKILLRMQLCD

VELSLMAMVSYVGVITGIAILFPYFNQVVGVAGAINFWPIVVYFPVEMAWELMRKKKKTRWVILSSSQMCPCVQQPCSAP

LSPPSPQSELAKMENHVTLLRPMHDYYKRRSLILGNYMPSSPHEKSGNEWTALAHIITAVIGSGVLSLAWSIAQLGWIAG

PITMLCFASVTLISAFLLCNCYELIDQHGTITNRHGSYLDAVQSILGDRNAWFCGIIVRINFIKLGIVYTITSAISIRAI

QKSNCYHNDGHEAECRYSNTKYMIIFGLVQALVSQIPDFRHTEWLSIVAALMSFAYSFIGSGLSLAKVIDNGEIKGGIGG

WPSPNAGKKIWSVAEALGNIAFAFPFSVIFLEIQDTLKGPTEKVTMKKASIMAVCTTTFFYLCCGGLGYAAFGTKTPGNL

LTGFGFYDPYWLVDFANACVAVHLVGGYQVFSQPLYAITEKWLRLKLPQNQFFQVDYNLKLNKHLPAFRLSFLRVIFRTS

YVAVITGIAILFPYFNQVVGVAGAINFWPIVVYFPVEMYLKQKKIESWTTKKIVLRIYTYVCLVVILFAFVGSIRDSGPR

FVGRVLRKLRNDDETLTVETQICHLCDHTDTSGNLSYIIMGRIPCCEKENVKRGQWTPEEDHKLSSYIAQHGTRLQRCGK

SCRLRWTNYLRPDLKHGQFSEAEEQTIVTLHSVLGNRWSVIAAQLPGRTDNDVKNHWNTKLKKKLSGMGIDPVTHKPFSH

LITEIATTLAPPQVPHLAEAALGCFKDEMLHLLTKKRIGFQLHHPVGPVKHEDKDETIEKIKYGLSRAIKDPAVDQMLPA

AGNNKPWDHAGGATSSNLLGKPAMVFPPPIITADFTTAFRALCTTAARTGRRGTRACAPEARARRRSNRGGTCMTKRRRM

KTAASVRRAAENAQPRLTRRRPAFSTPTVSCGIFRLKISSTLWLRRPVKVAIGISAVGDMSSVGLMAYLRSQSGAHSHHI

GGLSSASSALVEQEQYLAELLEERSKLKPFMPVLPHCYRLLNQAEILRVTTLLGNASVLDQSGLEHANPLASGGIFSNGG

ANVDRWASVSPFRSESSGLTEPLSSQNWRSSQSSSSGLIVRRTIRIDIPVDLFPSYNFVGRLLGPRGNSLKRVEASTDCR

VLIRGHGSIKDPTKEMMRGKPGYEHLNEPLHLIVEAELPVEIIDARLMQAREILEDLLKPVDECQDFYKKQQLRELALLN

GPKPRGDLAVRCGWASKLTRSSKRLLSSSFNATTAVAKPTSLQFQCNSGCYPANAFKCHRGIGYISFNI

>g332

MDMKTCCNSSSHDPEIRKGPWTMEEDLILINYIANHGEGVWNSLARSAGLKRTGKSCRLRWLNYLRPDVRRGNITPEEQL

LIMELHAKWGNRWSKIAKHLPGRTDNEIKNFWRTRIQKHLMKQEAARNESTVISGHGSSCEKTDQASSTTSASQLSSIGQ

GDIYSSPQSSFACNMEIETGFQGHHFPSHEYSNDAVWSMDDISWRRRGGMGRAFVYVIVGGGVAAGHAAHEFVKRGVSPG

ELCIISEEPVAPYERPALSKGYLLPEDPARLPSFHCCVGTNEERLTPKWYKEHGIELVLGTRVKSADVKRKTLLTASGET

ITYKILIVATGARALKLEEFSVSGSDAENVCYLRDLDDANKMVNMIQSCSGKNAVVIGGGYIGMECAASLVINKINVTMV

FPEAHCMARLFTPKIASYYEEFFISKGVKFIKGTTLASFDFDADKKVDTVNLRDGTKLPADMVVVGIGIRPNTSLFEGQL

TIEKGGIKVNGEFQSSNSSVYAVGDVAAFPLKIFGETRRLEHVDSARKSARHAVAAILGKETKDFNYLPLFYSRVFTLSW

QFYGDNVGEVVHFGDFSGHSFGAYWVNKGHLVGSFLESGAKEEYEAISIATRLRPAIEDLTELESQGLAFAMALSLDSPQ

LQPVDAAGSSALVVEKPLYPWHATAGVILAASIAAFGYWYGRRRRRW

>g980

MGRTPCCNKEGLRKGAWTAEEDKILVAYITKNGHANWRSLPKLAGLLRCGKSCRLRWTNYLRPGIKRGQFSSEEVDTIIQ

LHTVLGNKWSAIASHLPGRTDNDIKNFWNSHLRKQGSDPNHQNHQMPHPHGKIDEKVETTPSTNHIVQWESVSVAPSLLD

LPSASKMDHHDPFLRLWNSEVGEAFCSFKKPRGVPCQSPVSSSSKFESSSGITLHSEPASKLLSSADTVEGVPMNCKVEE

IEEEEDTKSYELVDPSETTLKLLLDFPPVVNDMGFFQGPGDNLSIYLQN

>g1015

MGRAPCCDKANVKKGPWSPEEDAKLKSYIQLHGTGGNWITLPQKIGLKRCGKSCRLRWLNYLRPNIKHGEFTQEEDNIIC

SLYITIGSRGLKGQSSTRWWRMVDGGQNGQILHLTFQNVKLSASHAYKEIKWSIIAAQLPGRTDNDIKNYWNTKLKKKLL

GTNLQPSQHHRPADLYSLPQQTLPYYSDIHNQPPSLSCKFQCPDDNDLQPHQNPIAATANTAFSSASIFSVGTSNSQLGF

NSFPIELADDLFYGGYDQTQESFSYSMELHNLHQEMIPPNILEGPTYLI

>g1296

MGRSPCCDKVGLKKGPWTPEEDQKLLAYIEQHGHGSWRALPSKAGLQRCGKSCRLRWTNYLRPDIKRGKFSLQEEQTIIQ

LHALLGNRWSAIATHLPKRTDNEIKNYWNTHLKKRLAKMGIDPVTHKPKNDALLSSDGQSKSAANLSHMAQWESARLEAE

ARLPAHQARGARGRLAAVSGHTKSLGRRRRQRRRRAAFSVAGLGVGVEALESPTSTLSYSENAPQISSSTAAFMKEESED

QAWKCFGNAAEHFKGGGGVEDSAAAAGFASAGLLGLTNIPAAMESGAWTTQEHIPTGNFVERFTDLLLSANSTERSLSEA

GSTESNNAAGDGGSGNHYYEDNKNYWDSILNLVNSSPSNSPMF

>g1471

MSTELILSSLGLTLVPEAEPQLGLGWDEHGLILSSQCRDSGEAKHGLGLGSLRGSGHGLGFAAKTGMKKGAWTEEEDNLL

RKYIEEFGEGKWHQVPAKAGCFHTAYQPLGTRSLVSSPKLHPCNLFNHLGDSRENGERREELLEYPPQKTAGIGMPRPPH

RQQENGRENIVIGPPNSSKISSFLSLGTETTTTKTTMPDNDVSKRTFTPSPPADSVTQWWENLLAIGDRDGAITWSPSLT

TSDEGDKAVADGETWSRVIGDGNSAFIDEGEFSWNEFQFPMGTRYKRTEPCSSIGWLELEFVKEARLEFELELAYF

>g1661

MDKTAAKTGMKKGAWTEEEDNLLRKYIEEFGEGKWHQVPAKAGLNRCRKSCRLRWLNYLRPNIKRGEFAWDEVDLIIRLH

KLENSERREELLEYPPQKTAEFGMPKPPRRQQENGRGNIVDIGPPPRPPNFSNLSSFLSLGTETTTTTTMPDNDVKTTMP

HNDVSKRTFTPSPPADSVTQWWENLLAIGDRDGAITWSPSLITSDEGDKAVGDGETWSSRVIGDGNSTFIDEGEFSWNEF

QFPMGTSKWNDGIRDLGE

>g2322

MGKAPCCDENGLKKGPWTPEEDKKLSDYIEKHGHGSWRALPKLAGLNRCGKSCRLRWTNYLRPDIKRGKFSQEEEQTILH

LHSILGNKWSTIATHLPGRTDNEIKNFWNTHLKKKLIQMGYDPMTHRPRTDLFSNLPNLLALATLLQSHPLEEHAAAAAR

LQAQATAQNMAAKIQYLQMLFQSSSSSSSMTTTSSSSYDNNGDFWDFNLPNLSNKETDNNPLLNSLSQMENQTLFSVENS

GASQLLHNQVPFNFQTQHLNNDNNNKSLNSDSILPPLTDCFLNNNNHKGDSSSTSSNGDYQGTSSPSSYNWPELLLEGAF

MHDIS

>g3509

MRFMIKGGIWKNTEDEILKAAVMKYGKNQWARISSLLVRKSAEQCKARWYEWLDPSIKKASTEWTREEDEKLLHLAKLMP

TQWRTIAPIVGRTPSQCLEHYEKLLDAAARAKDENYDYPRNWRLRSGEIDPNPESRPARPDPVDMDEDEKEMLSEARARL

ANTRGKKAKRKAREKQLQEARRLASLQKRRELKAAGIDDVPHRNRKRKGIDYNAEIPFEKKPPPGFYDVTDEDHTVEQPK

FPTTIEELEGERRVDKEARLRKQDIARYKIAQRQDAPSAILHDSETVRKRTKLNLPAPQISDHELEAIAKFGIASYQRHS

ASSYNSLFFANCEPKSKVSMDVRTEHYNLDRKGSTKDYLSDDLTLPPLSTSSSPSSILNSLPSGRRSAVPIANRQWPWTL

LSSTLFVDDYENSNFPKQMLLAAFSDDEKCPTRVFGVRHYYAHQLHCLQPHDGTRCTSAAVSTIHVEHRRFRNLSRGRRY

VELQSGEARLLSQKLECTGVRGVLFRDRRVEGHRDRWLGIVAYDPYHGIPSLCIGLQGSLWYFDVLVTSKDDFQFSGFCS

WVFGDYDNCNNNLSLYQKTKIKDISFEDNLDIDVLVGFVPVPITLHPFKMDMICLGWGDVLVSFNMKTRKLEALDIPNGE

TLRLVGAVADEEEQQKGQKCPSVSQRLNVKLDGGLKLSNVYKV

>g3510

MRIMIKGGVWKNTEDEILKAAVMKYGKNQWARISSLLVRKSAKQCKARCIQCVNCATKVIAEYKYCLSACKTEWTREEDE

KLLHLAKLMPTQWRTIAPIVGRTPSQCLERYEKLLDAACAKDENYEAGDDPRKLRPGEIDPNPESKPARPDPVDMDEDEK

EMLSEARARLANTRGKKAKRKAREKQLEEARRLASLQKRRELKAAGIDVRHRKRKRKGIDYNAEIPFEKKPPPGFYDVTD

EDRTVEQPKFPTTIEELEGERRVDKEARLRKQDIARNKIAQRQDAPSAILHANKLNDPETVRKRTKLNLPAPQISDHELE

AIAKFGIASDLIGSEELLEGNAATRALVANYTQTPRQGMTPLRTPQRTPANKQDAIMMEAENQRRLSQSQTPLLGGENPM

LHPSDFSGVTPKKKEIQTPNPLLTPSATPGGTGLTPRIGMTPSSDGYSFGMTPKGTPMRDELHINEEMDMDGGKVGRSDS

RRELRSRLESLPNPKNEYQIVMQPQPEESEEPEEKIEEDMSERIAREKAEEEARQQALLRKRSKALQRDLPRPPAASLDL

IKSSLIRADEDKSSFVPPTLIEQADELIRKELLSLLEHDNVKYPIDEKSEKEKKKGTKRKSVSVPVIEDFEEDELKEAED

LIKDEAQFLCVAMGHETESFDEFVEAHKTCLSDIMYFPTRNAYGLSSVAGNMEKLSALQSEFENVKKKMDDDTKKAQKLE

QKVKVLTNGYQFRAGKIWSQIEATFKQMDTAGTELECFKVLQKQEQLAASNRINNIWEEVQKQKDLERTLQKRYGDLLGE

KERIEHLMDEYKKQAQMQEIEAKNRALELATAEADAADNKMIVAPSNEDPEPVASVNEHESSTAVDPAQESPNKQTEDGS

SMAVGPAQESPNKQAEHEGSMADDPAQENPNEQTDNAQEQPSGSPKLGMDIDEVGSTTDTNDLSQSTPAARESSLTDEVH

AENACNESESGVTSGGPQLMNADENPTSGNGGEASADASVSPIAEDQVS

>g4006

MGRSPCCEKAHTNKGAWTKEEDQRLINYIRSHGEGCWRSLPKAAGLLRCGKSCRLRWSLIAARLPGRTDNEIKNYWNTHI

KRKLLSRGLDPQTHRPINAAAAAGGGGGGSAAKDICLDFRNAAAPAKSSNEKATLSLSQEDTKCNSGTTTEESQSHQQQK

DDQTALNLGLSIGLSTAAETPSSSNTAESVAPPQAPPSAAAVGYAAMTQSVCLCWQLGWSPSGKLCTKCHNSYKWFP

>g4474

MTDYYSSFQDSSMKTESEDKMTSKGGISEDTTGGGNAGSVGGKALKKGPWTSAEDAILVEYVTKHGEGNWNAVQKHSGLA

RCGKSCRLRWANHLRPDLKKGAFTPEEESRIIELHAKMGNKWARMAAEENLSADWHVHLELREDKRAGLPIYPPDICLKA

LNESKQNEDFSTFPNGGTHHPDLLQINNFEIPAVEFRSLEVNQQLYPPALLEIPSSSLLDIPASSLLAHGLNSSYGNRSV

LSTMHPSKRIRGSEPLFPGLNVNAGDILPACSPYQNDSSVEIAQSFGFSSAYDQNLASGHSSFSGVISGSHASLNGNSSS

LEPTWAKKMELPSFQSQMGSWGSPSSPLPSLESVDTLIQSPPTEHNTGSGSLSPRNSGLLDAVLYESQTLKHTKSNSSEQ

TSGAFMMPGDIMDSSCPDLHGTELEAYGDPISPLGHSVASVFSEYTAISGSSLDEPQSVETMTGDKVKQEDAGSASVQCD

DKTDASNQDEMFSRPDFLLDSNCFDMKADHGKNHSILKDAFGAVLFDDFSKDCKIMGAVTTSTGQDSCAWDAMSTF

>g4696

MGRAPCCDKSKVKKGPWSPEEDAKLKDFIDKNGTGGNWIALPQKAGLKRCGKSCRLRWLNYLRPNIKHGEFSEEEDRVIC

SLYATIGSRWSIIAAQLPGRTDNDIKNYWNTKLKKKLMSMMLLPPSSSSSSSKHRSVISSSMSPLFSAAPTGPSVLLPTP

PQQQFSFYTPHRSFSGLEPRPLSTTRISGPGMNKGNHNLLMFGGSEVSGSSSSDGSCSQISYERSKIADHNVKQEPAADH

QLLGKYAFQGDPPPPPHHQIISSTNAGFQDDNNQIQGHTFFLDYGGLSNGATATAAATATQQLQYDLEVVRELVSSSSSN

GHGHDNHNNNNNNNNDFLLFNYENKTDDQKGMYFY

>g4815

MVQEEFRKGPWTEQEDVQLVFYVNLFGDRRWDFLAKVSGLKRTGKSCRLRWVNYLHPGLKRCKMTPQEERLILELHSKWG

NKWSRIARKLPGRTDNEIKNYWRTHMRKKAQENKKKKGGACISASSSSLTNCCSYSSSANSPAAVESEVDEANERNFYDT

GGIDEETTQVPPVQENGAAAKAYTMDEIWKDIEQLGDVYCGNNQQRSVTTSSPMWNYWAETLWMTTADYNHGGGESKTAS

FPPLPLPLPLPPPPTNNDQFYSSFDNQGSIFLTG

>g4989

MEDSGGGGGGFGYTNLQNGHHFRSGPRLPLDRFLWAHNRQNQFSSSQQQVPENRSETMFFPENRFFDSPLIVGEHSNGHA

ASLVGGAASWPIPQEVSFYDAATFVHGVDRSALVGNNVNESPGRQVISRSSSSTLLIKGQWSEEEDRNLIRLVKQYGVRK

WAQVAENMAGRAGKQCRERWQNHLRPDIKKDSWSEEEERVLIEAHEELGNRWAEIAKRIPGRTENSIKNHWNATKRRQNS

RRRIKKVKDNNNESNTVNKSTVLQDYIKAKYPKVDSGAGVSESPSIQTDQILSPPAGEDDDSSSLLMQEAYHDEDMSFME

SLFGNPTPYPTIMAADHPDNLKKHNEEPIPAPPDRYLAYLLEGGTSSSSLMEFTMGSYGYPSLSSGTSKAKDIDLMELIF

SSPSQSSQGSNNNTN

>g5295

MGRAPCCDKTAVKKGPWSKEEDQILINYINKYGHGNWRQIPKNAGLVRCGKSCRLRWMNYLRPDIKRGHFTHEEESIVVK

LHKTFGNRWASIAARLPGRTDNEIKNIWHTRLKKRLHDFDNQVAGDTLNNDGASNFAAAPPAEDNDPSGGLAGLTQSDFP

LGDDDQFWPQINDKSGFEFNGADFNPTMDNDDVGGVDPDRLQFWQDHLLTWTGDEFVDLWK

>g5645

MRTQSSSSAKGAAGASPASKAKGKGKTTPCCSKVGLKRGPWTPEEDKLLTDYINKEGEGRWSLIAGRIPGRTDNEIKNYW

NTHLTKKLISQGIDPRNHKPLLVNPTNNPKNHTSSSYSAPIIKPTPIHVGLSNQDKTVKINSSVNVGGTTNHQIDQPNSV

GGTTNQTTGNGGGDEDFNMDGGGIISCCIGEDNEDNVGMDFCPDEDVFSTFFDSLMNHDVFFAASQNNQQSNHDITPLQP

STSEHNDNNNQPLNPLQQMNFPFSTAGWVDGDGDDFLP

>g5886

MGRSPCCEKAHTNKGAWTKEEDQLLINYIRLHGEGCWRSLPKAAGLLRCGKSCRLRWINYLRPDLKRGNFTQQEDDLIIK

LHSLLGNKWSLIAARLPGRTDNEIKNYWNTQIKRKLISRGIDPQTHRPLDSSAGAGAGTGTTKPENFSMDLSSSAPSQEE

TKCSSGTTSEESNHHSLKDKQRNEQMGGLDLSIGLALHPKTEDSAESTASGELLPVAPPPPAAVELSVTEAVCLCWQLGS

RTGGLCNKCHTTKCFLGYCQPS

>g5902

MGRPPCCDKVGVKKGPWTPEEDIILVSYIQEHGPGNWRAVPTNTGLLRCSKSCRLRWTNYLRPGIKRGNFTDQEEKMIVH

LQALLGNRWAAIASYLPQRTDNDIKNYWNTHLKKKLKKVEGSSEDGQDGNSSSSSHQSISKGQWEKRLQTDIHMAKQALC

DALSIHKPATPNRFNRSIPLNPPVQPSSGSTYASSTENIARLLQTWTKPVQSRSTGDHNPEFVEQQPVPWTRFKLQPERR

HGLELCAFDQTVLASTLTWTKITLGFSPLKASHKPWLPKMVTPQII

>g8292

MVRAPCCDKIGVKKGPWSPEEDQILISYIQRNGHANWRALPKQAGLLRCGKSCRLRWINYLSPEIKRGNFTKEEEDAIIH

LHETLGNRWSAIAARLPGRTDNEIKNVWHTHLKKRLSNYEPPMKPIKHPKTTTATSEKHSNIIQHATGPTSPHQTSSDDM

SSSSSATDSSAMTDTITEHPAMIKLENAGSAEGFVEIDESFWTDVPSFGSPVSSLENFMEQSGGRYEYEYDGG

>g8510

MGRSPCCEKAHTNKGAWTKEEDQLLINYIRLHGEGCWRSLPKAAGLLRCGKSCRLRWINYLRPDLKRGNFTQQEDDLIIK

LHSLLGNKWSLIAARLPGRTDNEIKNYWNTQIKRKLISRGIDPQTHRPLDSSAGAGAGTGTTKPENISMDLSSSAPSQEE

TKCSSGTTSEESNHQSLKDKQRNEQMGGLDLSIGLALHPKTEDSAESTASGELLPVAPPPPAAVELSVTEAVCLCWQLGS

RTGGLCNKCHTTKCFLGYCQPS

>g8940

MRKPDPMGKDGKLGNNNSKAKLRKGLWSPEEDEKLMGYMLRNGQGCWSDIARNAGLQRCGKSCRLRWINYLRPDLKRGAF

SPQEEELIIHLHNILGNRMTLQCTPSYGNDIVVSNCGASPGRTDNEIKNFWNSTIKKRLKNANNNQNNSSLSPNTSDSSS

ENPRAMIGGCGGGGGAAAAALILPAMHHHQPEFMASICMDSSPSSSSSMHTNMLPHFNPFPPQPAGFEAAPGLFGLPPSL

AAQLVGVGGPSAAGECGFLGDYNVVVEPYGVMGLENDLSVPALESISATNNHTNKDSKGENNVVDNNNNGNVNNINNVDY

YGIFDKKANAVNPNNNNNQHLYEEGSIKVEDYMVGFGNHHWSGESLRIGELDWEGLLANVPSLPYLDFQVQ

>g9289

MGRAPCCDKKGLKKGPWTPEEDEKLTEYIKKNGHGSWRSLPKFAVSQEEKLAYAIVWEELAVDMSSMGIFLVTVNSSDKT

FAKMQVGLLRCGKSCRLRWTNYLRPDIKRGPFSPEEEELVVQLHGLLGNRWAAIASQLPGRTDNEIKNLWNTHLKKRMIS

MCIDPQTHKPSSDANGLMWTMPTTPSARHMAQWESARLEAEARLSRESLHLVPSPTTVGSETDFFLRMWNSEIGESFRKL

KKGEKADCQSPTSETSSSMKCGSASGTTEVDPSLTVHSAADCNPNKDPEQKSCKSYIEYPLSEMSCTDELDDSSESALQL

LLDFPTHNDMSFLEHTDAYTIHPTPFSPKIL

>g9635

MGRSPCCEKMGLKKGPWTKEEDEILVDYISRHGHGNWRALPKHAGLLRCGKSCRLRWINYLRPDIKRGNFSHEEEDAIIK

LHQALGNRWSVIAARLPGRTDNEIKNIWHTRLKKRLNDYDLVPPQPRLKSKSQPLKPFAMDLLITNGTLLTPSSPPHSST

TTSTDVHALSACSISSDCVVSDAVLQSDPPEVDESFWSQVFSLENSSDAGDLPATVDGENRFDSTENETYETKSSVEFWH

RLFSKAEDLPVLPEL

>g10520

MGRHSCCYKQKLRKGLWSPEEDEKLIKHITTYGHGCWSSVPKLAGLQRCGKSCRLRWINYLRPDLKRGTFSQEEENLIIE

LHAVLGNKWSQIAARLPGRTDNEIKNLWNSSIKKKLRQKGIDPNTHKPLSEVENEEKLVASLTNNNNEKLTLSEGSSELN

FVEAETCNNNNNQVPSLAVMDSYPMIDNPMSTNTPPTHHEFFLNKTPDLSGYLSFFNNNIGSSFPMQHPSNSSLFLNPKS

NVVLPMETNSSFFENNASFSWGPATDHHHHCGGKPEKQSADLDETKWSEYLQAPFYLQPPPPPNHQDLYGEGKPETQFAT

QGGSPLIPTTTSATAAATATATTTWLQNQAMYNNSKNFQRLHAAFGQFS

>g11082

MTSESEDGMTLKGSVSSPSIEEVTAGGKTGGSVLLKKGPWTSAEDAILVDYVTKHGEGNWNAVQKHSGLARCGKSCRLRW

ANHLRPDLKKGAFTPEEECRIIELHSKMGNKWARMAAELPGRTDNEIKNYWNTRIKRRQRAGLPIYPPDLCLRSINARQQ

NEDMNIFSIGDMQHPDLFQINSFEIPAVEFKNLELNQHSYPPPSLVDIPASSLLDIPSSSLVGQGLKSSCGNRYFHSTVH

PSKRLRGTETLFPGLSENGSDFLPACSRFQSGAYIAAQSFGVSSAYNQNLASDDPSLLSVIPGSHASLNGNSSYSSEPSW

EKKPELPSLQSQMGSWYSPSSPLPSLESIDTLIQSPPPTEHTESGSLSPRNSGLLDAVLYESQTLKHSKNNNTSSQQTTS

DASIIPGSIVDNSCPDLHDTEWEAYGDPISPLGHSSASVFSEYTPAVNGSCSLDEPQTVVAGCKVKQEEEGMELSPMQCD

TGRDDDEESFQNMFSRPDFLLASNCFGSQTEYSKNHCVLKEVFGAVLFDDFSSRDCKNMRTATSSGGSPCPWDHAMSTV

>g11443

MRKPEFSSGKNGANVGNSNIINSNNNSSNNVVVANNKLRKGLWSPEEDEKLMHYMLSNGQGCWSEVARNAGLQRCGKSCR

LRWINYLRPDLKRGAFSPQEEELIIHLHSLLGNRWSQIAARLPGRTDNEIKNGLKKRMKKRTTSNTSDSSSLEPCTGGLI

SSSSSMQDHHHHQALMSSIYMHDPSSSSSSSSSFSSSMPLSAAAADPLPMLGHSISAAASFMETGHALGGGGAYYRDHGI

FGGNIGAEEGPNHLFMVPPLESVKSEGFVSRMSIHNTTNPSHNNNNNNINTNNIDINNHFMNNNNSVKVENVGGGFGSYW

DNGGEWDLEELMKDVPNSFPFLDFQVE

>g13062

MMGTEQMGWGIMGGWRKGPWTAEEDRLLVEYVRFHGEGRWNSVARFAGSSNTVVDGTRSLLPNFTPVTRLKRNGKSCRLR

WVNYLRPDLKRGQITPHEERIIVELHARWGNRWSTIARSLPGRTDNEIKNYWRTHFKKKAAKSAATNNSDHPKARILRQQ

QQQQQQQQQKKKKQEESNHQMDMRNMMSSFDEIDENRLSVPQPQPPTDLLSDQEQGFLYSMINGCASVTEPSSTEDMIMC

DGLWNLDDFHGSFSANKACFLQPVAAPFY

>g13125

MGLKKGPWTPEEDQILVSYIQKNGHGNWRALPKQAGLLRCGKSCRLRWTNYLRPDIKRGNFTKEEEDTIIQLHEMLGNRW

SAIAARLPGRTDNEIKNVWHTHLKKKLKGYQPLKTPRNNFPARLPAPSPATLPEILVVVGVERDVVGDGGSGGGRAGGWG

EAGRRELVAGVLAGDRRELLDGRGGGGFAVGASDDEFPVVGASLANSDDVDRMMWHSTTLDDDMDFWYNVFVRSAGELPE

LPEF

>g13520

MDHVKGGGAYKSVAQQQAEDDADLRRGPWTVEEDFTLINYIAHHGEGRWNSLARCAGLKRTGKSCRLRWLNYLRPDVRRG

NITLEEQLLILELHSRWGNRWSKIAQHLPGRTDNEIKNYWRTRVQKHAKQLKCDVNSKQFKDTMRYLWMPRLMERIQAAA

ANAAVSSSTTTSANSDGAAYIHNPLDISATSDPAGMQQHPNNAAPTIPDYNAAAAAAVNFPSDNSSTAASSDSFSDLTDC

CGYNFHVTQGANHDYYQPNNNNHFGYGESLTSPTGFFNPNLDFQMMDVNNNNNNNNNNNHQWMDGADVVSDNLWNIEDMW

FSFQQQFNNNNNNSHP

>g13997

MMGWGVLDEGWRKGPWTAEEDRLLIQYVKLHGEGRWNSVAKLQGLKRNGKSCRLRWVNYLRPDLKRGQITPHEERIIIEL

HAKWGNRWSTIARSLPGRTDNEIKNYWRTHFKKKAKSSAADNTEKSRARLLRKQQFHLQQLQQQQQQQQQYNIQTDITKK

VIPLFDENNETRVAAPSVPQGQGGVFYPEQDQGFFFSLMNGYVPSVAEAPSNDQDSIMWDGLWNLDDFQGNFCAVSAANK

ICSLQPIAAPFY

>g14592

MRIMMKGGLWKNTEDEILKAAVMKYGNNQWARISSLLVRKSAEQCKARCNSILVECLQLALRFLTEWTREEDEKLLHLAK

LMPTQWRTIAPIVGRTPSQCLEHYEKLLDAACENYDDSRNWKLRPGEIDTNLESRPARPDPVDMDEDEKEMVSEARARFA

NTRGKKAKRKAREKQLREARRLASLQKRRELKAAGIDVDARHRNRKRKKGIIDYNAEIAFEKKPPPGFYDVTDENRTGEQ

PNKFPTSIEEVEGERRVDKEGRLRKQDIIARNKIAPSAILHDYPETVRKRTKLNLPAPQISDHELEAIASYQRRSASRF

>g14671

MGRYPCCKDSEEVKKGPWTPEEDQKLSDYVSKNGHGNWQMLPKKAGLNRCGKSCRLRWTNYLRPDIKRGGFSQEEEQIII

NLHSSLGNNPQLAPISSFFGRGEEKKSTAITGWSRIAAHLPGRTDNEIKNFWNTNLRKKLLRMGIDPKTHQPITDINLLL

NLSHQMMLSNTNNPLGSALRDLDDLEVMESNTQGDTTHEWKSGMKIESLSHKNEWAPAPKEKKKIVLNHRSIGVLDMENS

LPKEMATDKSSVEILGEKCANLTLGMEAEYELEVQEPDNGDGPKECINLRWAAVGRFLSDTAIKFEVMRQVLATVWRPVF

GLVIKELPGNRYIFQFFHEKDICRILEEGPWRFENSTLVLKRLTDDDQPSKVDLDRYLQTPDYYFGVWMRASLRRTVAPI

SERWLAIPEDSKTMGTPLQTPNFDSATTMHGDLSKEPSSSANARVSMDLDENGITVVDTKRRRGNDHDKKIGSEAQLVNH

SPRLNHLREASHELAASMSVFSWNCRGLGNPRAVQHADMVSKKRPLFRSSMEIKVVRTHVQRLKSNFGFNGVGAVILIVH

GGGFSQQSGIEVVDLGVLDQLSGMGIKFEDEVLGPWLLNTLPDSWETFRVSLTNSAPNGAVTMEYVKSGHIKKYCFKFKR

DKKQEQNDGDTENQVATVVRADLLVAFDENAINVACHETTWIVDSGAAYHVTPRKEFFSSYTQGDFGELRMGDDGQVKVT

GTGTVCLETSNGAILVLKDVKHAPDIRLNLISTGKLDDDGFCCFFGDGHWKITKGSLVVARGNKHSNLYSLQSSVSDDSV

NVVEKECASELWHKRLGHMSVKGIDYLAKKTREAVSFMSHPPSRKSEPLELIHSDVCGPMKVRSLGGAYYFVTFIDDYSR

KLWVYTLKHKSDVLGVFKEFHALVESKAGEEIEFLIKFGVERKFLTITLESSVVRHLCMFRDERSKLDSKTRECIFIGYG

FDEFGYGLERAESHASGSLVDIERLPRGTHDVDEVQENVQNGDLVPEYQDDAANVDGHVDDFVHRTGGGGIFNTRCRVAA

AMKSGGTTRGTRYGSRNGWIGWYQTHNGARIIARGENANIVTNAWSSNPCMIYLPVEFIAQMLSQLGGSYHNKFGKLKRI

RRALITSNRADRLEWMRDGNTRFFHQYASGRKRKRVSVTNRDATFLILTCTKNLLVIVSAVRFHKGLGKANIVLLPKKQT

PEIVAELRPIALCNTIYKVIAKLVANRMKGMLRGLIAESQSAFVPNRLITDNILITAEAGHFLRRKVGGVQGWSGLKLDM

AKAYNRMEWDYVRFMPTKFGFADEWVNLIMLCVESAEYDIRINEGSVWTGLSALIRDAEMKGTIHGCRIARGAPAVTHLM

FADDCLLFFKANSEEVEAIKGCLTGYEQASGQSINYNKSTVCFSSNTPTETCAMISNCIAVPIAADFGKYLGLPSVIGRN

KMEVFKYIDQKIRQRVGSWQKKLLSRARKETLIKSVAQSLPTYSMSVYLLPDSLCDTAQKVMNKYFWESGGGNSRGVHWL

SWDRLAFPKGQGGLGFKKLRDFNRALLANVLAGREVIKIGLARRIGNGRQTKIWSVPWLHDRNNPELITPINALHGDQWV

SSLLLLDNERWNVELIRDLFDPMDVNRIMKTPVNREMEDGWYWRHELRGGYPVRSGYRLLSNSPQENNNFNSWGKLWKLD

VAPKIRNLLWRCVKEILPFRTTLRRRKIDTALECPLCNNEEEMVAHIFLECPVVAGVWVAAGIPINLTAPNFSRWLESVL

AKANALIWLTDWYDLIKRKVMTKVRTESPQPTAALRCFVDAALFENVDLAGFGVIAVNNEDFIVAACSGTHCSSLDPYHA

ELWAIKEALSWIKKENWSGVVVCSDCLNACNALNKPSDDRSYAGIIIRQCHDIMETLHQTSIMHVARGRNMRAHELAKSS

ILYRNSRYWRFEPPVCTLEFSIT

>g15127

MSSRKSGGARMHVHKGAWTAEEDKKLTQYIENHGAKKWKTVAIKSGLNRCGKSCRLRWLNYLRPNIKRGNIAEDEADLIL

RLHKLLGNRWSLIAGRLPGRTDNEIKNYWNTHLSKKVTQVGKSSLSATQNQPPKANAEQVGGNKESEEEDPELNFDVDEF

FDFSVEGTYGTEWVNKFLEVEN

>g15640

MGRQPCCDKLGVKKGPWTAEEDKKLISFILTNGQCCWRAAGSPARKSCRLRWTNYLRPDLKRGLLSDAEEQLVIDLHAHL

GNRWSKIASRLPGRTDNEIKNHWNTHIKKKLLKMGINPVTHEPLNKEETKPTSDDQSSTLPESDQKNGQQQVQVVPQSTT

QVTTSEEQSSSCSPTENSSSSTATTTEVVVVLDAVGGGEDDEDPLLSSLLENHAPILPDAAQWDLPLPPDNQPPLIFDEN

HDLGMPTLLDDNFGWLFNCQDFGIQDFGFDCLNDPEMHIFDTVDSAGNDHNK

>g16830

MGRKPCCDKVGLKKGPWTTEEDKKLINFILKNGLLRCGKSCRLRWTNYLRPDLKRGLLSDYEEKMVIDLHAQLGNRWSKI

ASYLPGRTDNEIKNHWNTHIKKKLKKMGIDPVTHRPLSSATIDDDNKETAKQRESGQEMEVVETPLMDQSTITDAFEEDN

KNMETSPTLVDPHQVNNNNGFFFSSPDNNEILATSLSSSSSSSTSSLNSHGGFVSSVEDNNNNNNNNVGGLFEDLVDFLP

GFDWHNDYINDVGLWVEDDDFFKELCFVDIHDGYLRVGYPVERAMVARVHSVGALSRRWVEQIVWQLTQWQSGCGARLGT

FR

>g17105

MADSCSSEPPSGVKKGAWTEQEDNLLRKCIHKYGEGKWHLVPVRAVVLNRYCNCVERELQLRGTYVVSSPGNRSLSLNRC

RKSCRLRWLNYLRPDIKRGDFNLDEIDLIMRLHKLLGNRWSLIAGRIPGRTANDVKNLWNTRLQKKTIANNTPSSGQEKW

KDKAPKTTENTAVIRPRPRRFVMTSSSRTLPITGKTTTIVTSEVVQLQGHNKPPEAAESTSAPRLIENVDPNNSIIDLAG

GAETSDDLGQWLDDFLLDMEFDGDGMACMQEGQIEWCDFHIDSDLLDLLS

>g17106

MVNSLSSAWPSPSGLMRKGAWTEEEDNLLRKCIQKYGEGKWHLVPLRAVCSSKWSNPTPVRRRSKWSSPASCLSKCDTWS

LIAGRIPGRTANDVKNYWNTHIQKKVFAMAAASSRMQDNWKGKAPEISKNTVVKPQPRRFLNTSSISRTSITGKATAVTY

DAQIQAHTLPQPETTTTSDLVMENVQKNDTIASFPSELETTTFDDRVQWWEELLFDKELNDEGTACMHEGQVGWSHLPTD

IDLLELLS

>g17108

MAAASSDNWKGKAPEMRENTVVRPRPRRLSHRTPLTGKATAVICDAQIQGHKIPTSELVMENLQENNTITSELETTTSND

KVQWWEDFLFDNEGSTCVNQGQVGWANFTIDMDLSQLLS

>g17110

MQKKLQTKMAELSPWSLIAGRIPGRTANDVKNYWNTHIQKKVFAMAAASSRIMQDNWKGKAPEIRENNVVRPRPRRLSRT

PLTGKFTAVTYDAQIQGHKLPTSDLVMENLQENNTITSELETTTLNDRVQWWEDFLFDNEGSTCMNQGQVGWANFPTDMD

LSQLLS

>g17137

MAAASSRIMQDNWKGKAPEIRENNVVRPRPRRLSRTPLTGKFTAVTYDAQIQGHKLPTSDLVMENLQENNTITSELETTT

LNDRVQWWEDFLFDNEGSTCMNQGQVGWANFPTDMDLSQLLS

>g17139

MAAASSDNWKGKAPEMRENTVVRPRPRRLSHRTPLTGKATAVICDAQIQGHKIPTSELVMENLQENNTITSELETTTSND

KVQWWEDFLFDNEGSTCVNQGQVGWANFTIDMDLSQLLS

>g17159

MGRAPCCDKANVKKGPWSPDEDAKLKAYIHQHGTGGNWIALPHKIGLKRCGKSCRLRWLNYLRPNIKHGEFTREEDNIIC

ALYLSIGSRWSIIAAQLPGRTDNDIKNYWNTKLKKKLLGVKQRRDHTVEMMSSKEREKLQGEQTAMNVCPPPVAFPAPEI

LCEYPMYTTMGCITNSYSSSFATNNNYSPLVMSDSLATNDVFRQAFSFPTAELNHHDFLYGYNSPQQLADRYSSTITSGS

SSEGTSSSWEDISSLVYPPPKMVSTNHHLDEICPPTMFSNPNLL

>g17283

MVGAVEKAGWRKGPWTPEEDKLLGDYVSLHGEGRWSSVARCAGLNRNGKSCRLRWVNYLRPGLKRGHISPQEEGIIIELH

ALWGNKWSTIARYLPGRTDNEIKNYWRTHFKKKPAAGKTSEKQDRRKNRRKRNEEKDEVNDTKPQETMSNNDSSCITAAA

APMGDNNQLGGTSATTTTTFYHEDIESWVDSFAMDMDGLWGGGLWNLDDDDSYPEAALLEQGHVIQNPCGFGADHAVNLW

NGGFIF

>g17403

MGRSPCCYKLGLKKGPWTPEEDHKLLAYIQEHGHGSWRALPVKAGLQRCGKSCRLRWTNYLRPDIKRGKFSLQEEQSIIQ

LHALLGNRWSAIATHLPKRTDNEIKNYWNTHLKKRLAKMGIDPVTHKPKNDVTVFPRDAAAATLSHMAQWESARLEAETR

LARRSTLTCSENVAQISTAAAKIGNSSASDFHGGIFPDNAAWTTEDSVRTANTQHIPTLTGNFVDTFTDLLLSRASADRR

FCDGGSTDSINGGGSNYYEDNKNYWNSILNLVNSSPSDSPMF

>g17420

MGRPPCCDKQGVKKGPWTPEEDIMLVSYVQQHGPANWRTVPAHTGLRRCSKSCRLRWTNYLRPGIKRGCFTDQEEKMIIQ

LQALLGNKFSSSSFSSHSSSSRGQWERTLQADINTAKQALNDALSLEKPTRHRPLDFIPQPPAPVQYASSADNIARLLKG

WMESPAKSYSTPSTSNSNGGVATTTVTDTSSSSSDDAPGGICLSEAFESLFGFDSDQFSQSYESKPQVPLSVMLESWLLD

DDI

>g17487

MDKKPCNSQDVEIRKGPWTMEEDLILINYIANHGEGVWNSLARSAGLKRTGKSCRLRWLNYLRPDVRRGNITPEEQLLIM

ELHAKWGNRWSKIAKHLPGRTDNEIKNYWRTRIQKHMKPQQGGENAATRQSSSSCCGGEQNEQASTSQASAPDTVETYSP

TSYNNNMDATFQGTFPTESNVDNMWSMEDLWSMHLLNGD

>g17691

MVGASEKVGWRKGPWTPEEDKLLGDYVSLHGEGRWSSVARCAGLNRNGKSCRLRWVNYLRPGLKRGHISPQEEGIIIELH

ALWGNKWSTIARYLPGRTDNEIKNYWRTHFKKKPAAGKTSEKQDRRKNRRKRNEEKVINDTKPQETMSNNDSSCITAAAA

PMGDNNQLGGTSANTTTSYHEDIESWVDSFAMDMDGLWGGGLWNLDDDDSYPEAALLEQGHVIQNPCGFGADHAVNLWNG

GFIF

>g17983

MAPPSKSGGGGGTGEEDDEVVVLEDGSGNSDQMSDKKAAADRVKGPWSPEEDAILSRLVSNFGPRNWSLIARGISGRSGK

SCRLRWCNQLDPAVKRKPFTEEEDRIILQAHAVHGNKWASIAKLLPGRTDNAIKNHWNSTLRRHYAALGKLKGEFGNMGE

DVSPERSKASSEDTQSCGDVNSLKASEGKDFFSVENPNDVPSEDKNQDAVQSIEEPREPPSLFALWRCGSSSSHLKSKYR

ISKLLEGAFGEHLVPRQCGHGCCGNSPQGNHNSSLLGPEFVDYSESPSFPIHELAALATDISNVAWRNSGLENGSINLFS

NSASSVMCGASHAQGESLEEMRKSDHHHSQLERGKALMADPASTPISRQPLQGQSNSSLNSLSHFLALLLQEGSLTSFPL

LPAFILTPYTTSHTPKQHTWCKSVPPFTFTPSLAPSVGNATKCRVPLINRMRPTTIKISDPAPSLPPTADVSYSFITIDN

NNKKKDQIRTGNNHRCRSREAATTTSTSAAEVASWKERDELTNRRGHYTPATAAATTVTPLPPPTAASTCDLQRLVCAPS

SDASGSPPPTPTSSMVSPYLSLAGEQ

>g18115

MELDTAELEDAPVSAGDATVESGGGGGTGEEDDEVVVLEDGSGNSDQMSDKKAAADRVKGPWSPEEDAILSRLVSNFGPR

NWSLIARGISGRSGKSCRLRWCNQLDPAVKRKPFTEEEDRIILQAHAVHGNKWASIAKLLPGRTDNAIKNHWNSTLRRHY

AALGKLKGEFGNMGEDVSPERSKASSEDTQSCGDVNSLKASEGKDFFSVENPNDVPSEDKNQDAVQSIEEPREPPTLFRP

VARVSAFSVYNSLDGPEAFFSVPRVTPLQGSSLQATSNPNIGISKLLEGAFGEHLVPRQCGHGCCGNSPQGNHNSSLLGP

EFVDYSESPSFPIHELAALATDISNVAWRNSGLENGSINLFSNSASSVMCGASHAQGESLEEMRKSDHHSQLEKGKALMA

DPASTPISRQPLQVNTKT

>g19448

MGRAPCCSKEGLKKGPWSTKEDLLLTNYIQQHGEGQWRSLPKKAGLLRCGKSCRLRWMNYLRPGIKRGNFSPEEEDLIVR

LHSLLGNRWSLIAGRLPGRTDNEIKNYWNTHLLKKLKSSGIEPRPPRKIVTSKKKATIPKIVTSKKPANNNSRNKKLQRK

EISDDNQRYKVYAPKAIRLSSRNNSVEDIAGSVSSSSGEVENKGIIDGSSSFIPWNLYELRDDFCAEVLTAAGDDLSPQC

ALPVGSDDCLLDKVYDEYLQLLSENCFLEDDPFGANL

>g19644

MGHHSCCNQQKVKRGLWSPEEDEKLIRYITNHGYGCWSEVPEKAGLQRCGKSCRLRWINYLRPDIRRGRFSPEEEKLIIS

LHGVVGNRFHNKAPPPTPTNPFSFPNTNPNPFFPFDAKAAALEGVNGDYTDRAAAAIDQPFQDSPAFWQQELQAAAAFSM

GMDSGYLPPLMENIMAPPPPSVELPAPPCNVALEAQMARDESDHVSEWAAVDTQQCPGSFLFWDQTDGEELALPTTSNIG

AILSSSFPSSI

>g20464

MVNLKKGAWSPQEDRRLISYITKYGIWNWSQTPKFAGLARTGKSCRLRWINYLRPDVKRGPFSVEEVEVIVRMYLSLGNS

VPVSRRAAAAAENGRKRTNKSKNNDNAPPPVMVQKHPQVVAEKPRHDDDQVLLMNSSPGGFGNPNVAHREQEVHRRFSSL

SSEDDYDEDRSFWYNVLKEADDLKF

>g20734

MRRPSSPTLSGSSGGRGDENAGGVKKGPWTPEEDKKLVDYIRKHGHGSWRAVPKLAGLNRCGKSCRLRWTNYLRPDIKRG

KFEEEEEQLIIKLHSVLGNKWSAIAMRLPGRTDNEIKNHWNTHLRKRLLQMGIDPVTHRPRTDINFIDALANLPQLLVAA

ANMGNNSNVANPLWDSINALRLCSDAAQIANELQLLQNFMALQLQLRGSVNNTTNEAQNQIPELATQFGSCNQLLDHLAL

LNPQLQGGLCNLGSSYNFSRLPPNISCSGSVATSSTSQNSEIQIHHPGIISNETNQGQTINSNVSRINDDSNKLMTNAFT

VSSSSPLNVPSGEDIPSNPIFPTLIPASPFPENPSSSIDWETNKEKYTISANLKHDIPNHVPNATTTFEAWRDIKVDDDE

ATDSYWQDIL

>g20840

MGRSPCCEKEHTNKGAWTKEEDERLIRYIKKHGEGCWRTLPKAAGLLRCGKSCRLRWINYLRPDLKRGNFTEEEDELIIN

LHSLLGNKWSLIAARLPGRTDNEIKNYWNTHIKRKLLSRGIDPQTHRPVSSAQNTAAADTAAVPLANNLDQQHETAAGGC

LPWMRNTKAENSNNTSSTTEDSNSSNGLSSEEIVLHPAAPLINLELSISLPQSPPAPSTTKLTGKEFNDQTLKNRLFFSQ

RAVCLCYNLGFQNSNACNCDKMMTTSSINAEAGMHSFYRPLSL

>g23112

MVSGDGDIASLRLLPVRFTAHDRDREPLGFRLSISTRYHTDLTIQIPQLCSDNYKVWKERILLNLGWKELDYAFNNNKPQ

IPTNSSTPDEIALYERWERSNRLSVILIKSNVSDSVRGIVDAYTDVKLLLEALDAQYASSEERLSMEMGESVMLSIPQGK

GKSRSTTKAKGKGQPQADIKKMRRCFFVKKKGHFKVDCIKFKKWLENKGNLSSFGLQALRKPGKWHYLIPGNKMGSQVEA

IGTYCIKGKQTNKSKKGATRSSTILEIIHTDICCPDIDMIGQKYFITFIDDYSRFTYLYLLSNKYEALDAFKIFKAEVEN

QCGKQIQIVRSDRGGEYYGRYTENGQTPGPFAKFLQEHGIVAQYTMPGSPDQNGVAERRNRTLLDMVRSWKPSLQHMRVW

GCPSEVRIYNPQEKKLDPRTISGFFVGYAQSSKGYRFYCPSHSTRFVESRNVKFLEDDLISGRDRFKDLIPTQEHNELHP

STSLDRLIIDQNTPQDHTEEVEPSTLQREGSPALRRSTQIRKSAIPSDYIVYLQESNVGETNDPETFSQAISCKESDLWY

EAMKDEMSSMRSNDVWDLVELPNDAKAIGCKWVYKTKRDSLGNIERYKARLVAKGFTQQEGIDYTETFSPVSKKDSLRII

LALVAHFNFELQQMDVKTAFLNGDLEEEVYMKQPEGFSSSKGEHLVCKLKKSIYGLKQASRQWYIKFDGIISSYGFVESP

IDNCIYQKGDKLSLDQCPKNDFERESMKDIPYASVVGSLGYVQVCTRPDIAFAVGMLGRYQTSGSLDVIGYSDSDFAGCV

DSRKSTSGYIFIMAGGAISWRSVKQTLIATYTMEAEFVSCFEATSQGVWLKNFISGLRIMDSISKPLKVYYDNSAAVFLA

KNNKSGSRSKHIDIKFLAIRERIKEKIVVIQHISPELMLADPLTKGMPPFKFKDLVGKMGLSLEDISLWKVDPLAENMDP

LSLKIRDSLSH

>g24292

MDQARTEDCWVENKQSPAASSSSISENSGSTNFKSPEISSPITSSPLNQRASGPIRRAKGGWTPEEDDMLKKAVAAYNGK

CWKKIAEFFPSRSEVQCLHRWQKVLNPDLVKGPWTQEEDDRIIELVGEYGPTKWSIIAKSLSGRIGKQCRERWHNHLNPN

IKKDAWTLEEELALINAHHAHGNKWAKIAKVLPGRPTRITFHFFISTEITILIPXNLPPAAKNGHQNGCKDITRTVTKNK

RFQCSKKQSDSTGLEPSATTDVCIFQVDGKVHEEPRTPVTVMNMGASTSVQQNKPANSESVQYEVQSSVCPQRIDTIHAI

SSTDLKFESCRVSDENGQHKLAFSGIPTYITVYYDSPQLHSYVQLERDHQNLPWAHHESQQSPSLPPATFLTPPSIKGRS

LYGQTPESILKIAAQSFPNTPSILRKRKTDVRLITPSKVVDGKKPRTSSEERRTDVSENSGLKDGSPSRNFSYNNSGLSS

TQVYNASPPYRLKSKRTSVLKSVEKQLEFAFEKEQSDDTTSSVKQISQATKDLPHKTKMANLD

>g24634

MGRPPCCDKIGIKKGPWTPEEDIILVSYIQEHGPGNWRSVPTNTGLLRCSKSCRLRWTNYLRPGIKRGNFTPHEEGMIIH

LQALLGNKWAAIASYLPQRTDNDIKNYWNTHLKKKLKKLQSPGSENTAQMGSENSPTSATSYHLVSKNSFTDRPQIATSS

SSLYASSAENISRLLEGWMRASSSSSSSKGFTSNSHTNNTSHGGDMEEEEDQTRPEPEGAATGSNDNNGVIPKEEYLDSI

LTFGSLMNGMESESCSGNNNKQKVNEGNDSDNPPPLSFLEKWLLDESAAQVEEEEGEDNQGAVMELPTIFS

>g25226

MGRAPCCDKANVKKGPWSPEEDAKLKDYIEKQGTGGNWIALPQKAGLRRCGKSCRLRWLNYLRPNIKHGEFSDEEDRVIC

SLYASIGSRWSIIAAQLPGRTDNDIKNYWNTKLKKKLMGLVSSSSSSQKISRPHQTPYPPPSILSSSPYSSPSISSPPPL

IKSTNYFPSPTFHALSIPPQHSSVAGAYVGPIHQNHQHHMVMTDRVLNFGGAAASCSSSDGSCTMMSSHGKDFDYSTESY

GGLQSFVYDNGDVKPSVVESGGCYGGNNPLDYSSLEEIKQLITTNNMCNNFFVDENKAQENVVLYY

>g25359

MGRQPCCDKLGVKKGPWTAEEDRKLISFILTNGHCCWRAVPKLAGLRRCGKSCRLRWTNYLRPDLKRGLLTQDEENLVID

LHARLGNRWAKIAARLPGRTDNEIKNHWNTHIKKKLIKMGIDPLTHEPLQKEDEEMLPPSSSSHSGDHQKHKPAENDEEE

EVPKMEEHKQCSSKEAAMFAAAPQPQVEADYYDPWMSQLWPDTFFNDLSWSFGGDECSDFGMSSSSHGSSEWLMDLQDFG

EDQSWALLPGNPPPKNMDMEIHTQDIITWKF

>g25413

MAEVKRGAWSPEEDTKLISYIRNHGIWNWTQMPKFAGLARTGKSCRLRWVNYLRPGVKRGPFSREEVEIIIKMYESMGNR

WSAMAAQLPGRSDNEIKNFFHTHLKKHLRRQEPKHAATDHHRISKADNTTSDDAKINIIADDCKLLGSSSSVVILESSRS

CSSRVVENNDGSSGGNNGTAVVAPPFSSDTDDDVNGFWYDVLMEAEYLNF

>g25699

MVRAPCCEKMGMKKGPWTPEEDQILTSFIQRYGHENWRALPRQAGLLRCGKSCRLRWINYLRPDIKRGNFSKDEEETIIQ

LHQTLGNRWSAIASRLPGRTDNEIKNFYNTHLKKRLQHHGSPYYSPNNVNSTYSISSPMTTKMEEEESMQESYQNLGTTS

NDDSGIVYLPSSSSVLPMELGGCETSSSISNDAVFWYNLLINAGNTS

>g25936

MGRAPCCDKTKVKRGPWSPEEDTTLKSYLQKHGTGGNWIALPQKADIKHGGFTDEEDQIILTLYTNIGSRWSVIASHLPG

RTDNDVKNYWNTKLKKKSSPPPSPPQTIIPTNGRIATASVPLPNLMEVQENCTTSTANNTANVSSYNPQVSAPCGGYSTS

FAYNNNNNENSFGTSWFGNGGIEEDVFRMDMMSSSTGLSSSSSPSSSSPSCFDHLMMNYGFDFQDNFAPPNITHNFTNHP

SNGFNISNLAHLTLLEKPSEWVPFRLDRAQFKLYLFVDIKK

>g26176

MKAKEVKTGLKRGFWTPEEDLTLKKCVETHGEGNWATISKKSGLMRSGKSCRLRWKNYLRPNIKRGMMSEDEKDLIIRMH

KLLGNRWSLIAGRLPGRTDNEVKNFWNTHLNKRSRRGKRMKITPKDDDSISASIPTQSMENTNLVEGSIKQEDEMDSIMN

SWMEHMGIENCNINSSISTNNLPWIFEDVPLIPILDDVLLDAFQRTGDETLLDGIHPFLL

>g27049

MGRSPCCDKNGLKKGPWTPEEDLKLIHYIQLHGPGNWRTLPKNAGLERCGKSCRLRWTNYLRPDIKRGRFSFEEEETIIQ

LHSVLGNKWSAIAARLPGRTDNEIKNYWNTHIRKRLLRSGIDPVTHSPRLDLLDLTSLLNLTQLNLSGLLGLQALASPEV

LRLLCTLMATQNENNSPQILLQKLQESQLNNNNQVENENPLLLLQKLQENQSPNAPIQSHQTAAFQPCDQFLDTVPSNNV

CASSSLPMQPTNLTTGQVIQESLIPSNGGNLMNGLHNNYYGVFEPEQSLSDWSNQCNFGLDSVLSTPLSSPGSTTFVNSG

SCTEDEKESDCIISNMMKFEIPSASLDFEDLL

>g28924

MASISPNGRRKDMDRVKGPWSPEEDELLQQLVQKHGPRNWSLISKSIPGRSGKSCRLRWCNQLSPQVEHRAFTPEEDDTI

IRAHARFGNKWATIARLLAGRTDNAIKNHWNSTLKRKCSSMSADEGNDLADRLQQQPLKRSVSAGAAVTLSGLHFNPGSP

SGSDVSESSLPVMSPSHVFKPIARTGGVLPPPVETPPPPPPVNDPPTSLSLSLPGVDSSSDVSPRLTESTQPISPIQLFS

SAIHSPPPPPPLPVPFQQPLEKFDLGGGAPPPMACPIPPKEAVPAPAQQDRVFLPFSQELLAVMQDMIKTEVRNYMMGVE

QQQPQPSQQQRYHQHQHQQQQQFQFQQQQQLQNGIGRGMCLQRATSNDGLRYAAAATVNRVGVNRLE

>g28993

MASISPNGRRKDMDRVKGPWSPEEDELLQQLVQKHGPRNWSLISKSIPGRSGKSCRLRWCNQLSPQVEHRAFTPEEDDTI

IRAHARFGNKWATIARLLAGRTDNAIKNHWNSTLKRKCSSMSADEGNDLADRLQQQPLKRSVSAGAAVTLSGLHFNPGSP

SGSDVSESSLPVMSPSHVFKPIARTGGVLPPPVETPPPPPPVNDPPTSLSLSLPGVDSSSDVSPRLTESTQPISPIQLFS

SAIHSPPPSTAPAGAVSTATRKIRSWRRRRAAADGLSNTAKGGGAGSSSARPSFSPVQPGAPGGDAGHDKDGGAELHDGR

RTTTTATIAAAALSPTPTSTTTAVSVSTATAIAEWDWARDVLAASHQQRRIEICSGGDRESRRS

>g29760

MGRQPCCDKVGLKKGPWTAEEDKKLITFILNNGQCCWRAVPKLAGLLRCGKSCRLRWINYLRPDLKRGLLSEFEEKMVID

LHAQLGNRWSKIASHLPGRTDNEIKNHWNTHIKKKLRKMGIDPVTHRPLSATDHEEEEEDDDDDQKTNKTKPQTENGTSA

DQESPVPGTSNDQSSITDLMVEEDNKSMETSQQSPVFESSIMEVSNGFCTDEVPLIEPHEILTCSSSSSDNYTAAGGDFV

SSNNVNINNNNINNVVEDMEFLPSLDWQCDSIDDMGFWGDDFITTTLDSLFNSDSNNNIINNNNNLNQVPLMVTDEESWK

LDQLL

>g29886

MGRSPCCDESTGLKKGPWTPEEDQKLINYINKNGHGSWRALPKHAGLNRCGKSCRLRWTNYLRPDIKRGKFSQEEEQTIL

NLHAILGNKWSAIATHLPGRTDNEIKNFWNTHLKKKLIQMGYDPMTHRPRTDIFSSLPHLIALANLKELVDQHQSSWEEQ

AAMARLQSEMAKFQYLQYLLQTSPAPAGPAFSPNNLNNTVTANNNTNNVSDMEAYSHLLSTVMGTSSSTQMESSVLPLSS

LGQAIQDSAIPFSHLPDLQPPCAIFQQSSDHNKDNNMAHQAQGISGFTVLSDGENSPTSPWLPSSLSPSPPPAVGPEPPT

VVDGSGGASSYGGAPPSSVVWPDLLLDDDPLFPDL

>g30119

MMGRPPCCDKANVKRGPWTPEEDAKILAYVASHGIGNWTLVPQKAGLNRCGKSCRLRWTNYLRPDLKHDNFTPEEEACIL

ELHKTIGSRWSLIAKHLPGRTDNDVKNYWNTKLKKKLKNMGIDPLTHKPFAQVFAEFGKLSGLPSPSNQNALLKNTIKNE

AVFEPEPRSFPTNAQNSRFVSPEMHEQLQIQNTPLVHNFPREPIQPPHSSPDTSFPHFASSQYCSSSYEQPLSQFLTSSS

STPWNEFILQDPCPMPDTELPRQDSKFPGTFSSDDPMTPLVQGEAGPICGFTNEDITEGDHGEASSSMAEDSFVENILAR

DRQMQLEYPQLLDGYFD

>g30889

MGRSPCCDKNGLKKGPWTPEEDLKLIHYIQLHGPGNWRTLPKNAGLERCGKSCRLRWTNYLRPDIKRGRFSFEEEETIIQ

LHSVLGNKWSAIAARLPGRTDNEIKNYWNTHIRKRLLRSGIDPVTHSPRLDLLDLTSLLNLTQLNLSGLLGLQALASPEV

LRLLCTLMATQNENNTPQILLQKLQESQLNNNNQVENENPLLLLQKLQENQSPNAPIQNHQTAAFQPCDQFLDTVPSNNS

LSDWSNQCNFGLDSVLSTTPLSSPGSTTFVNSGSCTEDEKESDCIISNMMKFEIPSASLDFEDLL

>g30895

MEGEEGQTWVTPNQETDEDSDEDLDFEEYSDITNIFFNRIIEWSAIAARLPGRTDNEIKNYWNTHIRKKASPERYRSGLQ

ALASPEVLRLLCTLMATQNENNTPQILLQKLQESQLLNNNNQVENENPLLLLQKLQENQSPNAPIQNHQTAAFQPCDQFL

DTVPSNNVCASSSLPMQPTNLSTGQVIQESLIPSNGGNLMNGLQNNYYGVFEPEQSLSDWSNQCNFGLDSVLSTTPLSSP

GVDNVCEQRELY

>g30900

MVRGPCVDKNGLKKGAWSEEEDDKLRAYVLRYGHWNWRQLPRFAGLSRCGKSCRLRWLNYLKPGVKRGSFSQEEDEMILK

LHKELGNKWSAIAAKLSGRSDNEIKNHWHTNLKKRLGLALHKKQDPGPSDADRLDLQPSKNSQEPQATQDQKHQINTAII

CGAVPAEISSHSQETSCSTTEGSSTFTSSQSFEEPLESFWNELLFSDPIYGGNGNTYYSSSESEEGLIMSSNSSTVEEDF

TLPYSLFCDDINFLSNFMQ

>g30901

MVRGPCVDKNGLKKGAWSEEEDDKLRAYVLRYGHWNWRQLPRFAGLSRCGKSCRLRWLNYLKPGVRRGSFSQEEDEMILE

LHKELGNKWSAIAAKLSGRSDNEIKNHWHTNLKKRLGLALHNKQDPEPSDADQLDLQPSKNSQEPQATQDQKHQNNTAII

CDAVLAKMSSHSQETSCSTEVSSTFTSSSSQSFEEPLESFWNELLFSDPIYGGNGNSYYSSSESEEGLIMSSNSSTVEED

FTLPYSLFXAGDFVDPDLTWKTPPVLVAPPPSSLFLVLLWCDLSAATIISTFGLLIWRILGAGTALVFSVACSQCWHHFS

GFGCGVVTVLPPSLVFQAVDLVDYQCWHHLCMLKVGVVSMMLNFLKVSAAAKALRSKIWRLQHLEMVEGNEVIKSSQAQI

IQMLGHMEQGFEDAVIFNLNVRNRQESRHLPK

>g30903

MVKTPSVDKNGIKKGLWSKEEDDKLKTFIESHGHKNWRQLPKIAGLSRCGKSCRLRWMNYLRPGLKKGAFSVQEDEIIIR

LHNKLGNKWSAMAELLPGRSDNEIKNHWHTHLKNRAKQTQKPEESMEELLPPSAETSEFSDFEFKPPQDDIFNILNLDEV

SLPQNSQTLDIPLSQEVSAASSGGGISSSSSSSSISDWILDDISTISLESFMDPLESFWTEPFVSDTIYPKNVGYPVTLF

EGENFPVQTSPLEGNFVPPYFEDAAWY

>g30904

MVRTPCCDVSGLKKGTWTVEEDKKLAAYISQYGCWNWRQLPKYAYRLPLLQVFVHIIYIIGLSRCGKSCRLRWMNYLRPN

IKRGNYTQEEDQLILKMHRQLGNKWSAIAAHLPGRTDNEIKNHWHTSLKKLTEQGYASSSSSPTQQPRKKPSAGRTKRSR

KQQPIPSASAPYSSSSTVSAHEILESSQWSSSEQAFSSSHSSSPSIGTSSPNGGNTPEPCADQNQTPPQVVGSSEDESFW

NEPFLLDNAFASGDDFLDYRPASPFSQYGQFSSSCNLYDELVNELMDYL

>g32142

MGRPPCCDKVGVKKGPWTPEEDIILVSYIQEHGPGNWRAVPTNTGLLRCSKSCRLRWTNYLRPGIKRGNFTDQEEKMIVH

LQALLGNRKTHTGKSPFVTLTFKTVSVSVAYRWAAIASYLPQRTDNDIKNYWNTHLKKKLKKVEGSSEDGQDGNSSSSSH

HSISKGQWEKRLQTDIHMAKQALCDALSIHKPATPEPFQPVHTLNLPVQPSSGSTYASSTENIARLLQTWTKPVQSRSNS

ETTIQSSLNNNPYLGPGSSSSPSEGTALSSAAFDQTVFGFNSNMDENNPWVFPVESKPQTVAPQNGNTSNNLGTQLPLTF

LEKWLLDDANNVPAPDDLMDMGMGIDLF

>g32158

MGRPPCCDKVGVKKGPWTPEEDIILVSYIQEHGPGNWRAVPTNTGLLRCSKSCRLRWTNYLRPGIKRGNFTDQEEKMIVH

LQALLGNRRKLKKVEGSSEDGQDGNSSSSSHQSISKGQWEKRLQTDIHMAKQALCDALSIHKPATPEPFQPVHALNPPVQ

PSSGSTYASSTENIARLLQTWTKPVQSRSNSETTIQSSLNNNPYLGPGSSSSPSEGTALSSTAFDQTVFGFNSNMDENNP

WVFPVESKPQTVAPQNGGGLGIGNQQRRGIAIT

>g32296

MGRPPCCDKVGVKKGPWTPEEDIILVSYIQEHGPGNWRAVPTNTGLLRCSKSCRLRWTNYLRPGIKRGNFTDQEEKMIVH

LQALLGNRWAAIASYLPQRTDNDIKNYWNTHLKKKLKKVEGSSEDGQDGNSSSSSHQSISKGQWEKRLQTDIHMAKQALC

DALSIHKPATPEPFQPVHALNPPVQPSSGSTYASSTENIARLLQTWTKPVQSRSNSETTIQSSLNNNPYLGPGSSSSPSE

GTALSSAAFDQTVFGFNSNMDENNPWVFPVESKPQTVAPQNGNTSNNLGTQLPLTFLEKWLLDDANNVPAPDDLMDMGMG

IDLF

>g33619

MGRHSCMVKQKLRKGLWSPEEDEKLYNYITNFGVGCWSSVPKHAGLQRCGKSCRLRWINYLRPDLKRGMFSQEEEDLILS

LHENQLRLKGGFESGPSCLYAREGHFEHNKCGHRLQQNCQGRTDNEIKNFWNSCLKKKLIKQGIDPNTHKPMAETQDDPK

NPTLPTPPNELPTNFPTTPQMEPSKLPFITTKQIFDPLFLYEPQENLNFPSTYLNPTYAFTSLPGLMNFDTNGQITETDY

FSDGSNSRMGSSNSSNNIGAAQMNNNMENGSRFSWEVGNRMESLFDQYGFSNNGEMIIKPEEEEEERQLIEAHCHDYTLT

SLPQDLGGANLDVFHQL

>g33626

MGRHSCMVKQKLRKGLWSPEEDEKLYNYITNFGVGCWSSVPKHAGLQRCGKSCRLRWINYLRPDLKRGMFSQEEEDLILS

LHETEQLQFDIKANLTQEVGSNLNLHALAPNPSYKRWAQIAAKLPGRTDNEIKNFWNSCLKKKLIKQGIDPNTHKPMAET

QDDPKNPTLPTPPNELPTNFPTTPQMEPSKLPFITTKQIFDPLFLYEPQENLNFPSTYLNPSYAFTSLPGLMNFDTNGQI

TETDYFSDGSNSRMGSSNSSNNIGAAQMNNNMENGSRFSSWEVGNRMESLFDQYGFSNNGEMIIKPEEEEEERQLIEAHC

HDYTLTSLPQDLGGANLDVFHQL

>g33699

MNGFDPFDPFSNDNSPSHHQNLNLFKPSQENSEIAHNNNNGGVVGGYLSYPNPKILSFHHDLKPTNVVVPDESSCVSANP

GFRKETGGRKRSNRAHQTTDNNGASASMKKHSKGRKKTKSSKGQWTVEEDRLLIQLVEKHGVRKWSHIAQLLKGRIGKQC

RERWHNHLRPNIKKDVWSEEEDEILIKAHVEVGNKWAEIAKRLPGRTENSIKNHWNATKRRQFSRRKCRTKWPRPSSLLQ

NYIKTVLNSEKNGGGTSRRNSSTNAEASTVVAPPPPPPPKAEPMEYCGGDNLVPDYDFALDEKLFAGNGIESFIEDIPGG

PLVLDEKYMEEMAYDMPPMMFGEPKELDLMDMISHVNL

>g34645

MGRSPCCEKVGLKKGPWTLEEDKQLLAYIQQYGHGSWRALPAKAGLQRCGKSCRLRWTNYLRPDIKRGNFSLQEEQSIIQ

LHALLGNRWSAIAAHLPKRTDNEIKNYWNTHLKKKLSKMGIDPMTHRPKINSSFGSAANLSHMDQWETARLEAEARLVRP

SKFIASSLISPRHFRNNPPPPPPPPKVPPPLDVLKAWQETWTKPPRTRVSSDGAFVSTATLNQSPTTLNFSDQILNFSDQ

NLCNMETQSSIGNPNNTAGDGIIPHVAMDPLTELPTFIHGLSYLSPEALTGYLDDDNVVGNCGTADVEDNSRYWNSILNN

LVASPVGSPVF

>g34994

MGHHCCSKQKVKRGLWSPEEDEKLIQHITSHGHGCWSAVPKLAGLQRCGKSCRLRWINYLRPDLKRGSFTEQEERTIIDV

HRILGNRWAQIAKHLPGRTDNEVKNFWNSCIKKKLIAQGLDPNTHNLLSPNQSKNNIHKPPTKNSYHHHHQLSPPSTFTI

DTTAPHTIKEVIPMGMKAALSSITPPFHQPVDYTNSMIPTSNDQNFDYQNPTTIMDFGSCSSSMESTTSSLIAAGFGIMA

GNDGAVFGTDTFEAAAQGFEPLRTQEEPQEQEDQVYKVNSDEFNNGGQNMFDAASNFDFEFMDAALMPCGVYSNVNPIDQ

LAWDC

>g35048

MGRAPCCSKVGLKKGPWSAKEDSLLTTYIQQHGEGHWRSLPKNAGLLRCGKSCRLRWMNYLRPGIKRGNFTPEEDDLIVR

LHSLLGNRWSLIAGRLPGRTDNEIKNYWNTHLLKKLKSEGMEPKPRKASRTIPKKKKTAKPDKTGSEKRKKAQARNNKAE

DNNNTPLNHNGNNNKAEESTGFPWDLYEVGDDLFDDYMDGCDLTARYSLPTSDTLLEKVYDEYLQLLSEDCCLQTCSSC

>g35053

MGRAPCCSKDGLKKGPWSTKEDSLLTTYIQQHGEGHWRSLPKNAGLLRCGKSCRLRWMNYLRPGIKRGNFAPEEDDLIVR

LHSLLGNRWSLIAGRLPGRTDNEIKNYWNTHLLKKLKSEGIQPKPARTSRAIPKNKKTAKPDKTGRDEKKKKHRHVSKPD

VINGVKDRPEMIKVYAPRPVRVSTGFARNYSFDDLAVDSASHGSIASDNNNNNNNNNNNTVEGTSFVPWNMYEVGDDLFN

DFMDGCDLSSNYLLPNPDDALLEKVYDEYLELLSEDSYLQTCLPS

>g35056

MGRAPCCSKDGLKKGPWSTKEDSLLTTYIQQHGEGHWRSLPKNAGVSSSVRDSILRLLRCGKSCRLRWMNYLRPGIKRGN

FAPEEDDLIVRLHSLLGNRWSLIAGRLPVGPITRSRTTGTSPPQEAQEREEKTAKRKCGSKPDAGNRDPDQPEMTTTKVK

VYAPRPVRVSTGFARNSSFDLRNCIATGQ

>g35179

MGHHSCCNQQKVKRGLWSPEEDEKLIRYISTHGYGCWSEVPDKAGLQRCGKSCRLRWINYLRPDIRRGRFTPEEEKLIIN

LHGAVGNRWAHIASHLPGRTDNEIKNYWNSWIKKKLKKPQSTLSHHHHQTTTTIIDQTTTSFGSGMGSGYLLPPLMDDLG

PLDVAPPCSLFDAARDNQVSEWGVDTTQQQQQRQQQQQCPGSNYLLWDEVTEGNLGGEEEEELIGVGVPTTSPMAAMLSS

FPPSL

>g35476

MTFFLKLPQPSITLIQFLPVSLSLSQTLSLFLANSHYIIISSFLPPTTGNHLSIHTSPQLLSFCSDNNTTLICTRHSCCY

KQKLRKGLWSPEEDEKLIKHINKYGHGCWSSVPKLAGLQRCGKSCRLRWINYLRPDLKRGTFSQEEESLIVELHAVLGNK

WSQIAARLPGRTDNEIKNLWNSSIKKKLRQKGIDPNTHKPLSELENDQENAGKKNGIASQESGELSLMESEHNNLISPPE

IAPDNNNKLSAMPIDQFSAPPQQSTQEFFLNNSPKIPDLTGYLCFQQMNNNNNNIGLSMNTNNSNLFFNPNNNVVPGSVL

PPSISSSHNNASCSSFFEANNGGLAWDCGKAEKESEEIKWSDYLQTPFLLGNTIHNIQAPHHHHDLFNETKPETQFASPQ

PSLPTTWLQNDQQPSLYNNTKHFQRLSAAFGQFS

>g36718

MGCIHVIAVTSADKHRNGSIAVHTPLVAWEVPALRATEVWEELSIEVDQLLETGSEEGDPSAWIQILTISSLLAKPRRPN

TTTPILVTKSHPPSAFTIETCSSSHKDVVPMEIKATLAAAFPPNTTPPNDKLLYHKNTITLSHERNNNPVMDYASCSSME

ITSNHNHLLPHPPIFGFGILNSDNCMWGGTTNLELLFGGSRQEEEDHIQEEMGAGTETQTRANQVYKLNTDDEFNNNNGQ

SIQINLFDNSNIGIDFVESSLMPCAMYCNANSMDQLPWDC

>g36720

MEINSIAGLQRCGKSCRLRWINYLRPDLRRGSFSEQEERTIIDVHRIIGNKWAQIAKHLPGRTDNEVKNFWNSCIKKKLI

AQGLDPNTHNLISPSQAKTTKHNNSHTRHQKSSPSAFTIETCSSSHKDVVPMEIKATLAAAFPPNTTPPNDKLLYHKNTI

TLSHERNNNPVMDYASCSSMEITSNHNNLSSSSSSNLSGFGILNSDNCMWGGTTNLELLFGGSRQEEEEDHIQEEKGGWD

>g37934

MVRTPCCDKSGFKKGTWTPEEDRKLAAYVTRYGCWNWRQLPKYAGLARCGKSCRLRWMNYLRPNIKRGNYTKEEDEIIMR

MHEQLGNKWSAIAAHLPGRTDNEIKNHWHTSLKKNSRQLPSSPAQPKKKSSSSRTKKQDRTNSFPENPIISAHEILESSQ

WSPQQSSSEACSSSPSSSSTTTTTTKSDEIIHDDQTVQGEFGHMECDESFWSEPFIMDNLWSKNEFDVPSIDFGLLSPPS

PFRDYDYLCSFDHFPGVNNLNW

>g37936

MLRAYVLKHGHQNWRKLPNLAGLSRCGKSCRLRWVNYLKPGIKRGNFTRDEDQLILNLNKQLGNKWSAIAARLPGRSDNE

IKNHWHTCLKKFDNVGVVESSKTHSSSHADDAQSVQSSDKSPQQHLAPFGLQAAMETLPFSPKTEPFSSFMDSAVLMSSE

EGTSNDYDDDDSRELLEQEFFIPDFADEEIQSLLNDFP

>g38712

MATMMSQKKDVDRIRGPWSPEEDELLTRLVEKHGPRNWSLISKSIPGRSGKSCRLRWCNQLSPQVEHRAFTAEEDETIIR

AHAKFGNKWATIARLLNGRTDNAIKNHWNSTLKRKCGSGSEDSGFEPPRKRSASVGAGVNVSGFYVSPGSPSGSDLSDSS

LPQVFRPVARTGGVFPTPVPQIETDSDPPTSLTLSLPGSDSSETPTSQLDSPHIVQSHAPPSQSHASQPQSHAPPPPPPP

PPISFSPVVAPSPSQPPPPPPQPQSFEFLPAPPPQTAEKPLFSPEFLAVLQDMIRKEVRTYMSGVEKNGLCMPTEAIRNA

VVKRISLSRIE

>g38942

MGRSPCCDKNNVKKGPWSAEEDAKLKAFIEQHGTGGNWIALPQKIGQQHQVTTRIAGRRLKRYKSSVLSIKNVTGLKRCG

KSCRLRWLNYLRPNLKHGGFSEEEDRIICSLYISIGSRWSIIAAQLPGRTDNDIKNYWNTKLKKKLFGKQVNDHGHSLRA

NQRQPEMVSCGPASSPNNINIYQNPSCPELVTVAMQPVPYPKEEPGFKDPTHLTDTLPVVNSILSSTGQYTMEDCLINQM

VYNVNPQNLEGVEFLCDNIFYNWKDGSSSSSAGGLDWNEINEIQSLVPSPPSPLASSYEGLEPAGNSVQLPAFE

>g39202

MGHHCCGKHKVKRGLWSPEEDEKLIKHLATHGHGCWSSVPKLAGLQRCGKSCRLRWINYLRPDLKRGSFTEQEERTIIDV

HRILGNRWAQIAKHLPGRTDNEVKNFWNSCIKKKLLAQGLDPNTHNLLSSNQAKINNNKNNKPINSQHFTVDQNSSPPKD

EPHIPLDLKASIAALTCIPNNEPHKYPFKFKYGPTGGYQYQNPSQNQLVTGFGTSCSSGESTSNNLSSSPSSSLNPVPGL

GAMNAENSMWGAGLFEPQFGPDHHQQQQQELYKVNTEFCSNNVQNMEMVKMFDDSF

>g39254

MGRAPCCSKEGLRKGPWSAKEDLLLTNHIQEHGEGQWRSLPKKAGLLRCGKSCRLRWMNYLRPGIKRGNFSQEEEDLIVR

LHSLLGNRWSLIAGRLPGRTDNEIKNYWNTHLLKKLKSAGIEPKPRQAKKKPTKPRPDSQNLNPNKKKKQKARNDEQPLR

QADQTTPPEKTQNRTKVYAPKPIRLTPPGISRINSLEDVAGSVSSSSGEVDNKAAVTADPPPFIPWHLYELGGGGDVDFC

DQILDGCDLSSPKCSGPTTDGLLEKVYDEYLHLLSENCFESLTDDYLCDYPFVDDNVAPTTSSNNSDLN

>g39379

MGRQPCCDKLGVKKGPWTAEEDKKLITFILSNGQCCWRALPKLAGLRRCGKSCRLRWTNYLRPDLKTLAELQWGQWSKIA

SKLPGRTDNEIKNHWNTHIKKKLIKMGIDPVTHEPLIKDTTNPTSDKGNGQLQVQVVPEGTPPTAPNLLTSEDLSSSCST

SENSSITSTNNDESQLVLDTMSDNDPLLSSLLENNAPPVDFTWSLSMIR

>g39784

MVTPATGAVGGRWCRDEMTATRLGIQQWKHCFHLFWLLLSSLSCLAAQMKNRAASGNYKVWKERILLNLGWKELDYAVNN

NKPQIPTDSSTLDEIALYERWERSNRLSVILIKSNVSDSVRGIVDAYTNVKPLLEALNAQYASSMKSLTSTLIMKFSSLR

LNTVRGTLPHHYEPFKISYNKHKENWSIVDLMTMCVEEEERLSMEMGESVMLSIPQGKVKSQSTTTKAKGKSQPQADMKK

MRRCFFCKKKGHFKIYCIKFKKWLENNGNLSSFGLQALRKPVGSERTILSRNKMGSQVEAIGTCELHEALDAFKTFKAEV

ENQCGKRIQIVRSDRGGEYYGRYTENGQTPGLFAKFLQEHGIVAQYTVPGFPDQNGVAERRNRTLLDMARSWKPNLQHMR

VWGCPSEVRIYNQQEKKLDPRTISGFFVGYAQSSKGYRFHCPSHSTRFVESRNAKFLKDNLISGRDRLKGLIPIQEHDEF

HHSTSLDRLIIDQNTPQDHTEEVEPPAPQGEGSPALRRSIRIRRSPIPSDYIVYFQESNVGETNGPETFSQAISCKESDL

WYEAMQDEMNSMRSNEVWDLVELPNDAKAIGCKWVYKTKRDSLGNIKRYKARLVAKGFTQQEGIDYTETFSPVSKKDSLR

IILALVAHFNFELQQMDVKTSFLNGDLEEEVYMKQPEGFSSSKERFRMKDCSPSVAPIAKGDKLSLDQCPKNDFERESMK

EIPYASVVGSLGYVQVCNRPDIAFAVGMLGRYQSNPGLDHWRAAKKVMRYLKGTKDHIFLAKNNKSGSRNPLTKGMPPFK

FKDLVGKMGLSSTLGMLAKHSNLTISMNSAHDTDSHGTHTSSTTVGDHVGGAALFCYTTGVATSIAPKARVAMYKALWDE

SSVKKRYCVGVDSSKDIYDKTESISGRSERPAVIALYEKPIAIAAFAAMENNFFISTSVGNQGPYFASLRGRPLSVVRLV

DQEFRGTLTLDNGVSVSLVSISWDKT

>g41253

MQNMKKKGSNGNNGNSNEAPKPKERHIVSWSQEEDDILREQIRVHGTENWTIIASKFKDKTTRQCRRRWFTYLNSDFKKG

GWSPEEDVLLCEAQRIFGNRWTEIAKVVSGRTDNAVKNRFTTLCKKRAKNEALAKENNNSFINLNNKRVIFPSALNIDRI

SGPGSPFKKLRRSHISDLPENCNSGEKSLVTCNAVNQLQRSPFSVLPNVGSSLPTNQNVSIKDSPTNASINKTEGTFLKK

DDPKILALMQQAELLSSLALKVNSENSYQSLENAWKVLQDFLNQKEDDVLKGRISEMDIQLEDFKGLAEDLKSINVPSQS

WRQPALSEDSAGSSEYSSGSTLLSHAPGDKKEQCEAEVSALHQDIESGLQSTELGDQTTINAFSGGTPANATCTEADILP

TCDKVSANDEAACEYSAEECSSPLHVTPLFRTLAAAIPSPKFSESIDIEMSQLTRWHCELRLAFMRNPK

>g41923

MASSARRKDTDRIKGPWSPEEDELLQKLVEKYGARNWSLISKSICGRSGKSCRLRWCNQLSPQVEHRGFTAEEDEIIIRA

HSKFGNKWATISRLLHGRTDNAIKNHWNSTLKRKCASKAAKTLVKPPQQPQPVIGSSGVLFSAPDSPPGSDLSSSSLSGA

VPPHVYRPVARAAGVVPPPKETVSSTTNPITSLSLCVPGSDSSEIPHSVIQPSPPPPAPVSLPVPSFLAQTYGSFQFASP

PTAEKRLFSPEFLAMLQDVIRKEVRDYMSGIEHGGLCLQTEAVQNAIVNRIGISKIDS

>g41977

MGRAPCCDKNNVKKGPWSPEEDAKLKAYIEEHGTGGNWIALPQKIGLKRCGKSCRLRWLNYLRPNIKHGGFSEEEDNIIC

SLFISIGSRWSIIAAQLPGRTDNDIKNYWNTRLKKKLFGKQRREHGGLKAGNVARQKQAEMRKAAVAAAARENPMMMMMA

PLANTNNNTPPWPELPVLDPIRFAPDNQPAFNDHHSIRNLLIKLGGKFGDDNDDDLKHKNVALYPTENPPAVLLPPLYQQ

SPMNQISSAPTTETLNSPFSINEYNMEAEIRSFPGENCFYTDNTNIPQKMDGLEFLYDNMLNNNNGRLGSSSGGAMMDWS

EMMSYCSLAFAPPLSSYTSSAVQPPAAQPHSAAALFDGEELISYSGTPPQ

>g42274

MVETTGGGGAAASASSDDSSRTCPRGHWRPAEDEKLRQLVEQYGPQNWNSIAEKLQGRSGKSCRLRWFNQLDPRINRRPF

SEEEEERLVAAHRIHGNKWALISRLFPGRTDNAVKNHWHVLMARRQREQSKLSVKRTYQQVFNNVNDTNVFRRRYKSPDP

DPYPYPSNKIISFLEFQNNPADSRPNTFSLSKYTPQAPPLRPTASSSDYSLLRHHKNLGFSLVGSHGNGIRSDPHNLIKT

GNSFLNYSSTFNDGGRGSGAKNHERDYLFTEKRERNDPQQELRQDKEDGGEDKRERRAVTFIDFLGVGSSS

>g42408

MVETTGGGTAASASSDDSSRTCPRGHWRPAEDEKLRQLVEQYGPQNWNSIAEKLQGRSGKSCRLRWFNQLDPRINRRPFS

EEEEERLVAAHRIHGNKWALISRLFPGRTDNAVKNHWHVLMARRQREQSKLSVKRTYQQVFNNVNINDTNVFRRRYKSPE

PYPYPSNKIISFLEFQNNPDNRPNTFSLSKYTPQAPYSSPPLRPTTSSSDYSLLRHHKNNTNKNLGFSLVGSHGNGIRLN

PPNLKPGNNFLNYSSTFNDGDRGAKNHEFRSEKRERNEQKDKEDDEGEDKRGRREDVTFIDFLGVGSSS

>g42847

MGRSPCCEKAHTNKGAWTKEEDERLIAYIKSHGEGCWRSLPKAAGLLRCGKSCRLRWINYLRPDLKRGNFTHDEDELIIK

LHSLLGNKWSLIAGRLPGRTDNEIKNYWNTHIRRKLLSRGIDPTTHRPINGGAEPPKETTISFGAVKPEDAENNNSITGK

DSEPKKEENKEETLLFKSEEPQVAEACPDLNLELRISPPSSQETRPPPLPLEAAKSGGGGRVNGLCFACILGIPNSIDCT

CNNNEDYSSGSSSSN

>g44150

MGVFVMLCLIPSTCGATSSLPEVTPPDDSRATIKAADEAPEINRRSLGDRRYERRAASLRLLGHTHRFCLKRELPDRPIP

VWSIFCGNAGAEGIATIGLRWRYAARGWRMVLLELAIQRFCYNAEVELWLQPRTKRRRGVRGGVRRGWWGYNMIEVSKTY

GGFSHTTIPHERYPAFSKNHVDVEVSIANLGIWRMTGFYGFPEHFNDLLFRTREGNPHHTISFSGFGEVVEECALARLPM

LGYRFTWERERDFDWMEERDEILHNICKNHLSLRWPGSTMRGAEWKRHGRRVEGLVCLTVFGYVVGSIPIILASIVLKSN

YMPIRAGRCVRKTRAQHWLRGADANTKFFHRDDGMNPGFYQHYWDVVGNDVSAFVIKCLDEGTIRDGLNNTNVVLIPKKS

SPEKVADLRPIALCNVIYKIMAKMIANRMKPLLGDVISESQSAFIPNRPGHSYSVFGREILPPRIFYNDDSLLFFKATLR

NTSRSIDTEVAALWMWFSSNLDLRAFNPASKSWRFLTRPHSLVARIYKARRIGDGKSTLIGTSPSLDDRTDDSHHYAREL

AGVSGLIDRLRSWIKTLSEIFSTMSDVQRILRIPISPHYEDAWFWLGDPGATIPSTLSHLESKECGGLGWMFARPDECVR

RKHISGSYIERGFSRSRRPSLSRSAISARLVLSAVSPLDSTPCHGGALLSRQWCFLGGVQRYSSTMFVTTHGGISCMHSC

CYKQKLRKGLWSPEEDEKLINHIEKFGHGCWSSVPKLAGLERCGKSCRLRWINYLRPDLKRGTFSQEEENLIIELHALLG

NKWSQIAARLPGRTDNEIKNLWNSSIKKKLRQKGIDPNTHKPLSQVENAEKAQVFNNNSGFLSSNRNNLVEFNNPKSSQM

ISGFNPNPNSNSNSNTILDHHCSSSIKLQGSPVFFNSSGFPWLVSGSEKSEKLTDPEDIKWSEYLLGNSIPNQPEPQFTV

TEEGSFSTTESTWLQNQHPSLQAANLCTAKSGYGFEFESQQAEQKLYS

>g44355

MASDSDEEFDGDGGFGRHGSPNEGCVLARAPTTVAMICRRAAAIVSHPPETDGVDDVELVRDIERFALSTDVRCLGYEAY

MLNIPPRVGMSLKMILKRSVLLNDDLRHIMTVDTTKEGLVKELNNTEQVGVTNITSEEESCNNFFLERTNAGEGFPTCVD

GNNSALQISKGAIPQGGGAENASSKSSGFPKSAQAFIDAIKKNRALQKLIRSKMIHVEARIEELKKLKDRVKILKDYQVS

CRKRTGHALAQKKDARVQLVLPRERVNSKLNEKKSSALYYAPPENSLVAGYRDALEKFPVSVNRERWSKEERENLLKGVK

QQFQETMFQRAIDLRDLDITPEMMRLFLPKVNWDCLASMYVPRHSSAECQTRKGACPGVFLRLSRWLNWEDPLINQEPWS

VVEDKNLLHIVQQKGLSNWIDIALSLGTNRTPFQCLARYQRSLNASIIKREWTEEEDNKLRAAVEVFGESNWQVVAASLE

GRTGTQCSNRWIKTLHPARQRVGKRTADEDKRLKVAVMLFGPKTWRKIAQYVPGRIGGQCRERWANSLDPSLNLNQWTEE

EDLKLEAALQEHGYSWSKVAACVAPRTDSQCRRRWKALFPHEVPLLREARKILKLAIIANFVDREKRKIASRYFGIKAGR

WMMGLGNMSSDDPTIAENCRYGITYQSRRSKRQPKRKICTNRRRRSPPFANPGMLSDGNELEGLEFSVAMNNRTSKLPPR

KKRKREPYVEVPEISASDEIETTNGEGIIFRRSTRGTEFDSGSDNDAKLDSFSSFPNSFSDARTLGGKAIKSRKRKRSIR

CPRKKCSDLYVSQGNHPFPTSRKREGPLREFPGSNLDADPSGDSSLSFEENSELESGAKQVEESHDRSSPLRCLAHNGLE

SNNFRTSSSCMQGKKKGLKCRDVDHSNKLAETEEDDSITLAAFIKKSLRLSSDHNSNENGAILERNIHTPTTSRGCQIQG

HGNGLGLGPSPPTSEAGIMDDMPLAHFLNTLKRRVKPATSK

>g46362

MGRAPCCDKDGLKRGPWTAEEDQKLIDYINKNGYGNWRTLPTNAGLQRCGKSCRLRWMNYLRPDIKRGRFSSEEEHVIIQ

LHSILGNKWSAIAARLPGRTDNEIKNYWNTHIRKKLLRMGIDPVTHRRRVDLLDLSSILNNPSLLYNSRILGAQNLYNVI

NSENANNVVQDTQQLHAPPPLIQDFPVYSPAMAAAQITQQPNVEFGVENYPANDFWLGTGLPESELTQDYLLPPLQNYGY

YEVAVDPQSAMDPPAPAADESCRFGFRQVWSTPSSSQVNSGSTTTTEDEREISYGSNLLNFDVANIFGVNEFIVMSRLRL

EPTVCPSLSLDELAKITILNELIDLVFDEHALVRVISRLRLDPLNELIDLVFDEHARVRVIFRLRRVILSLSPITPLLLS

TYVNKPAQSISSE

>g47242

MHQNMKKKSGSTNSSETTKPKERHIVSWSQEEDDILREQIRVHGTDNWTIIASKFKDKTTRQCRRRWFTYLNSDFKRGGW

SPEEDMLLCEAQRIFGNRWTEIAKVVSGRTDNAVKNRFTTLCKKRAKNEALAKENSNSSINRRVIFPSWLNSDSISESTV

PLKKQRRSHIPDPSESFSNGEKLVSCDATNQMLRSPFAVIGQNLYSPGSNLSSHQNDEDAKDSHANGSSNKTEGTFLKKD

DPKILALMQQAELLSSLALKVNSENTDQSLENACKVLEDFLNHTKDGDVTKCQIAEMEIQLENFKQSANELKNINECSQP

SWRQPALSEESAGSSEYSTGSTLLAHGVGDNGEKSDAELCALHQDIESGLQSTHIDDEFAKGISGNNASTSQVDTFPACD

KVNPVNETICEYSNEECCSPLQVTPMFRSLAAAIPSPKFSESVRETIPTKNAWNGIYVSESNHQSFSSSIVQEGPPP

>g47276

MASNRCIYPPPNYSPSPSSSSSSSSPFGASMGMVFADMGSLSIDPKNGGTQILQEASLPKNSEIGGGEGRGCWGFPSFLK

GGLEDHHHHNHNHHQNHQNQNNSDGDEGGKGSDCSDGFGENSEPIIDLNAAVLSGEDHHHHHHQPIMHVGGNNGKEAEST

GQSKLCARGHWRPAEDAKLKELVAIYGPQNWNLIAEKLEGRSGKSCRLRWFNQLDPRINRRAFSEEEEERLMAAHRLYGN

KWAMIARLFPGRTDNAVKNHWHVIMARKYREQSSAYRRRKMGQLVYRRMEEDDEIATPNNGGGGGPPPEIMAVIVVVVVV

AVEAIPTNNGHHHHHPPPPFSALCAPQQPPFDPFPGQCTNQMVGMLNQGRCWGKPHHHHHHDHHHILGHHPPPPPPPLMM

TPMQQQQQQYYNFPYSISPDFSSRPPPQPEPHPPPAAGGDDRKGGGTSSTTCDAISPPFIDFLGVGAT

>g47289

MRWTNYLRPDIKRGKFSSQEEQTIIQLHALLGNRWSAIAANLPKRTDNEIKNYWNSHLKKRLSEMGIDPITHKPKSNPFG

SRKPPTAWLGGRRLGSAGFALLATTPPCSILSRLSPPHHVSSSTSRQQPSATHIPPRRAKNNEDMCVSSSTAVALDRSPT

PLNFSDQNLVAFPAVGFGDDVLCYLEATSNMNTFGNPNTTGDDIMGPAMDPLSEFPTFILPENLSTHCSNGYLDNIAGNY

GAHSGXATIDRLVRAELTLSARSAVRPEITAIASFHRNYTSRRRSCRAGVTRTRHQVMSRSVETSLDVSNCRLVSGVYAD

ARDMAVAAASRHRGLERLRRDHLEGVARWIKSFGF

>g47290

MGRSPCCEKVGLKKGPWTPEEDKQLLAYIEQYGHGSWRALPAKAGLQRCGKSCRLRWTNYLRPDIKRGNFSLQEEQSIIQ

LHALLGNRWSAIAAHLPKRTDNEIKNYWNTHLKKKLSKMGIDPMTHRPKINSSFASAANLSHMAQWETARLEAEARLVRP

SKFIASSLISPRHFRNNPPPPPPPPKVPPPLDVLKAWQETWTKPPRTRVSSDGAFVSTATLNQSPTTLNFSDQILNFSDQ

NLCNMETQSSIGNPNNTAGDGIIPHVAMDPLTELPTFIHELTELSPETLTGYLDDDNVVGNCGTADVEDNSRYWNSILNN

LVASPVGSPVF

>g47294

MGRSPCCEKVGLKKGPWTLEEDKQLLAYIEQYGHGSWRALPAKAGLQRCGKSCRLRWTNYLRPDIKRGNFSLQEEQSIIQ

LHALLGNSCDERMNGLCSAAFFQHYQDTTQLQYHFNTTAISAICGPWSTMVAIAAHTPKRTDNEIKNYWNTHLKKKLLGD

RPARPARLVRPPSSSLLSYLTSPLTLHGRPLKSRRLGVLKAWQETWTKPPRTRVSSNVDGGAFVSNATTHQSRVVDFFGV

FEKKVMRLPSYQKKKKVKAKADTQEREGNPNNTTGDDIIPHVAMDPLSELPTFIHGFSELPPETLTGYLDDDVVGNYDYG

VEEQRLLEHYPI

>g47296

MGRSPCCEKVGLKKGPWTLEEDKQLLAYIEQYGHGSWRALPAKAGLQRCGKSCRLRWTNYLRPDIKRGNFSLQEEQSIIQ

LHALLGNSCDERMNGLCSAQLQFLSTLLQYQWSAIAAHLPKRTDNEIKNYWNTHLKKKLCKMGIDPMTHRPKINSSFGSA

ANLSHMAQWETARLEAEARLVRPSKFVTSSLISPHRLLLHSRPAAKVPPSRRAKSLARTWTKPPDEGFIQCRRRRVCLKR

HYTPISGC

>g47299

MGRSPCCEKVGLKKGPWTPEEDKQLLAYIEQYGHGSWRALPAKAGLKRCGKSCRLRWTNYLRPDIKRGNISLQEEQSIIQ

LHALLGNRWSAIASHLPKRTDNEIKNYWNTHLKKKLSKMGIDPMTHRPKMGKAANLSHMAQWETARLEAEARLVRHSKFI

SSSLISAPHKPPPPPKVPPSLDVLKAWQETWTKPPRTRVSSNVDGGAFVPNATLHQSPTPLNFSDQNLTTLNFSDQNLTI

LNFSDQNLTTLNFSDQNLCYMETPYVHESTLNIGNPKTTGDNIIPHAMDPLSELPTFIHGFSELSQETLTGYLDDDNVVE

TAHGRHGRQQPLLEHHP

>g47304

MGRSPCCEKVGLKKGPWTPDEDKKLLAYIEQYGHGSWRALPAKAGLQRCGNSCRLRWNNYLRPDIKRGNFSQQEEQSIIQ

LHALLGNKWSAIASHLPKRTDNKIKNYWNTHLKKKLSKMGIDPMTHRPKMNPSFGSAANLNHMAQWETARLEAEARLVRP

SNFIASSLISPHHFRLPTNHPPPPKIPPSLDVLKAWQETWTKPPRTRVSSNLDGGAFLSNAALVEDKG

>g47308

MERSPCCEKVGLKKGPWTPEEDKHLLAYIQQYGHGSWQALPEKSGLQRCGKSCRLRWTNYLRPDIKRGNFSLQEEQSIIQ

LHAFLGNRWSAIASHLPKRTDNEIKNYWNTHLKKKLCKMGIDPMTHRPKINSSSAQPPISATWLSGDRLEAEASPGRHSK

FISSNLISPHHNPPPPPKVPPSLDVLKAWQETWTKLGKLLKINKIL

>g47310

MERSPCCEKVGLKKGPWTPEEDKHLLAYIQQYGHGSWQALPEKSGLQRCGKSCRLRWTNYLRPDIKRGNFSLQEEQSIIQ

LHAFLGNRWSAIASHLPKRTDNEIKNYWNTHLKKKLCKMGIDPMTHRPKINSSFGSAANLSHMAQWEIARLEAEARLVRH

SKFISSNLISPHHNPPPPPKVPPSLDVLKAWQETWTKPPRTRVSLSHVDHGAFVSNATPTTLNFPDQNLCYMETPYVHES

NIGNPNPTGDDIIPHVAMDPLSELPTFIHGFPELSPKPSQDIWTTTMSSETTARPTWKTTAITGTTSVIIRITYKYP

>g47311

MGRSPCCEKVGLKKGPWTPDEDKQLLAYIQQYGHGSWRALPAKAGLQRCGKSCRLRWSNYLRPDIKRGNFSLQEEQSIIQ

LHALLGNRWSAIASHLPKRTDNEIKNYWNTHLKKKLCKMGIDPMTHRPKINSSFGSAANISHMAQWETARLEAEARLVRP

SKFIGSSLISPHHKPPSPPPKVPPSLDVLKAWQETWTKPPRTRVSSDGAFLSNATTYQSPTTLNFSDQNLTTLNFSDQNL

CYMETPYVHESSSNIVNLNTTGDDLITHVAMDPLSELPTFIHGFPELSPETLTGYLADDNVVGNYGTGDLEDNSRYWNSI

LNNLVASPVGSPVF

>g47312

MGRSPCCEKVGLKKGPWTPEEDKQLLAYIEQYGHGSWRALPAKAGLQRCGKSCRLRWSNYLRPDIKIGNFSMQEEQSIIQ

LHALLGNRWSAIASHLPRRTDNEIKNYWNTHLKKKLSTWLSGNRRLEAGSPRPPLKVHRFLSYLTSPQAAAAKVPPSLDM

LKAWQETWTKPPRTRVSLSHVDDGAFLSNATPTTLNFPDQNLCYMETPYVHESTSNIGNPNPTGDDIIPHVAMDPLSDLP

TFIHGFSSELSQETLTGYLDDHNVVGKHGRHGRQQPLLEQHPS

>g47316

MGRSPCCEKVGLKKGPWTPEEDKHLLAYIQQYGYGSWRDLPAKAGLQRCGKSCRLRWSNYLRPGIKRGNFSLQEEQSIIQ

LHALLGNRWSAIASHLPKRTDNEIKNYWNTHLKKKLSKMGIDPMTHRPKINSSFGSAANLSHMAQWEIARLEAEARLVRH

SKFISSSLISPHHFRLPTNNPPPPPKVPPSRRAKSLARNVDQAAEDEGNPNPTRIIPHVALDPLSELPTFIHGFSELSPE

TLTLTGYLDDNSRYWNSILNNLVVSPVGSPY

>g47319

MGRSPCCEKVGLKKGPWTPEEDKQLLAYIEQYGHGSWRALPAKAGLQRCGKSCRLRWTNYLRPDIKRGNFSLQEEQSILQ

LHALLGNRWSAIAAHLPKRTDNEIKNYWNTHLKKKLSKMGIDPMTHRPKINSSFGSAANLSHMAQWEIARLEAEARHSKF

ISSAQNSSFRLPTNNPPPPPLDVLKAWQETWTKPPRTRVSSGGAFVPNATPHQSPTTLNNVSDQNLNFSHQNLCYVHESS

SNIVNPNSTGDAIIPHVAMDPLSDLPTFIHGSSELSPETLTGYFDDDNFVGNCGTADMEDNNSYWNIILNNLVASPVGSP

VF

>g47799

MGRAPCCDKANVKRGPWSPEEDAKLKDFIHKFGTGGNWIALPHKAGLRRCGKSCRLRWLNYLRPNIKHGEFSDEEDRVIC

TLYASIGSRWSIIAAQLPGRTDNDIKNYWNTKLKKKVMAMMLINNNPSSSSSLSSSRSSAMVFSSSSSSSIAPPHLLHPQ

QLSFSGLDEHQSLSAAQNPYFLYDNNNNGSDYQSYPYNFQTQDQGFSNLMQFGGENSNNNNLLMFGGTEASGSSSDGICS

YGNKPEIKQEADDHHHQPLLGATCFQSTNAHSWDHNNNGGFDHHHQDQMNNNTGYFAGNNDLDIVRKLVSSSGCNDNICN

NNNSRLMLFNDINKTDERGMLYQCYYY

>g47810

MGRAPCCDKANVKRGPWSPEEDAKLKDFIHKFGTGGNWIALPHKAGLRRCGKSCRLRWLNYLRPNIKHGEFSDEEDRVIC

TLYASIGSRWSIIAAQLPGRTDNDIKNYWNTKLKKKVMAMMLINNNPSSSSSLSSSRSSPMVFSSSSSSSIAPPHLLHPQ

QLSFSGLDEHQSLSAAQNPYFLYDNNNNGSDYQSYPYNFQTQDQGFSNLMQFGGENSNNNNLLMFGGTEASGSSSDGICS

YGNKPEIKQEADDHHHHQPLLGATCFQPTNGHSWDNNNGGFDHHHQDQMNNNNTGYFAGNNDLDIVRKLVSSSGCNDNIC

NNNNSRLMLFNDINKTDERGMLYQCYYY

>g48266

MAQEEMMKMMMMRRGPWTEQEDVQLVLYVNMFGDRRWDFLAKVSGLKRSGKSCRLRWVNYLQPGLKRGKITPQEQRLILQ

LHSKWGNRWSKIAQKLPGRTDNEIKNYWRTHMRKKANEDRANKFGSSSSPSSSSSLSNNSSSSSGGSSSPPAVDSRPIPD

TNERNFYDTGGIIFEDETTPQQQQQQEKKIMDDHHQQQQQVCSSSMDDIWKDMELWAEQEKGGCTMWNYWSDWVWTTCSS

SNNNRMCPPPPPPSSSTDRLCFFDNQDYAQYN

>g49846

MGRTPCCEKVGLKRGRWTAEEDRILTDYIHANGEGSWRSLPKNAGLLRCGKSCRLRWINYLRSDLKRGKFSPQEEEIIIK

SHAILGNRWSLIATQLPGRTDNEIKNYWNSHLSRKFYSFRRAGSEKTIENLETDLVKAAEQTKRRRGKVSRSAMKKNKTT

GYKHYSSNNNQAPEFPHFQTHHTHKDTIPSLCTDPVNNNVAASAVQISSTPPILMDKEDIDTSSFLCMSGDYFSLDDIML

ALEEEMDPAGIILSTSLNGSRENSVKEFGLQVVQSQHDLAKSGNSSVDIYSGLIPNDHHQFGGENNESTATSSSFPVEHH

CSLAQNISDWDDLQYYWDDSGNNLCNTQNLMPQQNEDDVMLSSP

>g49847

MGRTPCCEKVGLKRGRWTAEEDRILTDYIHANGEGSWRSLPKNAGLLRCGKSCRLRWINYLRSDLKRGKFSPQEEEIIIK

SHAILGNRWSLIATQLPGRTDNEIKNYWNSHLSRKFYSYRRAGSEKTIENLETDLVKAAEQTKRRRGKTHHTHKDTIPSL

CTDPVNNNVAASALGRHNASIGGRDGSCRDNFEHFVKREPRCEKFGLQVVQSQHDLAKAVIAALIFIAGYPMITINLVGE

QRKHHNIIIIPVEHHSLAQNISDWDDLQYYWDDSETTSATPESNA

>g49849

MGRTPCCEKVGLKRGRWTAEEDRILTDYIHANGEGSWRSLPKNAGLLRCGKSCRLRWINYLRSDLKRGKFSPQEEEIIIK

SHAILGNRWSMIAAQLPGRTDNEIKNYWNSHLSRKFYSFRRAGSEKTIENLKTDLAKAAEQTKRRRGKVSRSAMKKNKTT

GYKHFSSNINHPPELPHFQTLCTEPVNNNAATSAVQIPSMPPILMEKEDDIDTSSFLCMAGSEYFSLDDIMPILVEEMDP

GTILSTSLGNSVKEFGVVAQSQHDDLAKKHESTATSSSFPVEHHCSLTQNIIDWDNWQYYWDDDSGNNLCNTQNLMPQHN

DEDVMLSSPWPWDDTFYT

>g49916

MGRAPCCDKASVKKGPWSPEEDAKLKAYIEKHGTGGNWIALPQKIGLKRCGKSCRLRWLNYLRPNIKHGGFTEEEDNIIC

SLYISIGSRWSIIAAQLPGRTDNDIKNYWNTRLKKKLLGKRKQSQMNRLLLAGHQESDHKETNGLGVTEENPFLQNLSNS

ALERLQLHMQLQGLQNPFSLYNNNPNNPPPLWPNKITPTPFQPKIADDEPIMASNLNIPEYSKMNELENCMNNNSGSSLG

QENNNNNNSGEIQTIPGFTQSEIDSLLINGGENHQISDFDCFKQMEGSKERLEWWNTEFDANSEIENLLINGGGTVMLPH

PHQSHQEGIMYEEYASLGYNM

>g49997

MMVSKSGEIQSLSNNNNEEERFGELRRGPWTLEEDTLLIKYIAAHGEGRWNALAKCAGLRRTGKSCRLRWLNYLKPDIKR

GNLTPQEQILILELHSKWGNRWSKIAQHLPGRTDNEIKNYWRTRVQKQARQLKIDSNSKKFIEAVKRFWMPRLIEKMEAA

DFYFSSSFISSPSSSSSISTMEKQSPINSLPSPITIPEPPKTDNPENKNLNICSTDSFQLHDDDDCYHVEIMGNSCYTGE

EEGFGHPAMSAFESRDISMLECQLAPDDWFGNDVADSLWNIDETWQYRKLEDFGDLS

>g50106

MRKASCDHSHHHHEINKGAWSKQEDQKLLDYIRKHGEGGWRDLPKAAGLLRCSKSCRLRWMNHLKQTAKRGNFGDDEEDL

IIKLHALLGDRWSLIAGRLPGRTDEEVKNYWNSHIKKKLLDMGIDPNNHRLSCTHNTAAQTSAGKSRVTSPEKQRVESDG

EVSDAGSSNVR

>g50509

MGRQPCCDKVGLKRGPWTIEEDHKLMTFILNNGIQCWRLVPKLAGLMRCGKSCRLRWINYLRPDLKRGALTEAEEEMIIK

LHSQLGNRWSKIAAHFPGRTDNEIKNHWNTRIKKKLKLRGLDPTTHKPLDQPGSSIAKINGVDDHNQQQSSSSGEEEMMI

MKNKDYPIPNYEYALQQTLNSSLDESLELESNIIPAGSEDMDNLSMDMYNPDDQRQEPYQTWIGSPVQWDLFNNLDGNFL

>g51043

MNIEEKSTIHGEWFLYILFLKLQSFYLDLESIQRERKFSPKREKTLRNFRCKTILWLLGLQRCGKSCRLRWINYLRPDLR

RGSFSEQEERTIIDVHRIIGNRWAQIAKHLPGRTDNEVKNFWNSCIKKKLIAQGLDPNTHNLISPSQAKTTKHNNSHTRH

QKSSPSAFTIETCSSSHKDVVPMEIKATLAAAFPPNTTPPNDKLLYHKNTITLSHERNNNPVMDYASCSSMEITSNHNNL

SSSSSSNLSGFGILNSDNCMWGGTTNLELLFGGSRQEEEDHIQEEMGAGTETQTRANQVYKLNTDDEFNNNNGQSIQINL

FDNSNIGIDFVESSLMPCAMYCNANSMDQLPWDC

>g51726

MRIMIKGGVWKNTEDEILKAAVMKYGKNQWARISSLLVRKSAKQCKARWYEWLDPSIKKASTEWTREEDEKLLHLAKLMP

TQWRTIAPIVGRTPSQCLERYEKLLDAACAKDENYEAGDDPRKLRPGEIDPNPESKPARPDPVDMDEDEKEMLSEARARL

ANTRGKKAKRKAREKQLEEARRLASLQKRRELKAAGIDVRHRKRKRKGIDYNAEIPFEKKPPPGFYDVTDEDRTVEQPKF

PTTIEELEGERRVDKEARLRKQDIARNKIAQRQDAPSAILHANKLNDPETVRKRTKLNLPAPQISDHELEAIAKFGIASD

LIGSEELLEGNAATRALVANYTQTPRQGMTPLRTPQRTPANKQDAIMMEAENQRRLSQSQTPLLGGENPMLHPSDFSGVT

PKKKEIQTPNPLLTPSATPGGTGLTPRIGMTPSSDGYSFGMTPKGTPMRDELHINEEMDMDGGKVARSDSRLELRSRLGS

LPNPKNEYQIVMQPLPEESEEPEEKIEEDMSERIAREKAEEEARQQALLRKRSKALQRDLPRPPAASLDLIKSSLIRADE

DKSSFVPPTLIEQADELIRRELLSLLEHDNVKYPIDEKSEKEKKKGTKRKSVSVPVIEDFEEDELKEAEDLIKDEAQFLR

VAMGHETESIDEFVEAHKTCSSDIMYFPTRNAYGLSSVAGNMEKLSALQFEFENVKKKMDDDTKKAQKLEQKVKVLTNGY

QFRAGKIWSQIEATFKQMDTAGTELECFKVLQKQELLAASNRINNIWEEVQKQKDLGALQKRYGDLLGDAADNKMIVAPS

NEDPESVAYVNEHDPAQESPNKQAEDGSSMAVDPAQESPNKQAEDGSSMAVAPAQESPNKQTEHEGSMADDPAQENPNEP

TDNAQEQPSGSPKLGMDIDEVGSTTDTNELSQSTPAARESSLTDEVHAENACNESESGVTSGSPKLMNADENPTSGIGGE

ASADAFVSPITEDQVS

>g52884

MASSPSKSSTSSCLTVEDMDGTELRRGPWTPEEDTLLIHYIACHGEGRWNLLAKSSGLRRTGKSCRLRWLNYLKPDVKRG

NLSPQEQLLILELHSKWGNRWSKIAQFLPGRTDNEIKNYWRTRVQKQARHLKIDSNSAAFQQLIRGFWMPRLIQKIQASS

IQHSDEILNTQSISQQVPAPAPAPHDCHQTTIINSGLENTPNLKLSECPKTALSNDPKFEPCPNGHHQYYATHVMDGFSP

SPFPETGEFKDVIDYDLLGTGGGNSLIMDEFLDDSLWSMDKF

>g52971

MGKAPCCDENGLKKGPWTPEEDKKLSDYIEKHGHGSWRALPKLAGLNRCGKSCRLRWTNYLRPDIKRGKFSQEEEQTILH

LHSILGNKWSTIATHLPGRTDNEIKNFWNTHLKKKLIQMGYDPMTHRPRTDLFSNLPNLLALATLLQSHPLEEHAAAAAR

LQAQAAAQNMAAKIQYLQMLFQSSSSSSSMTTTSSSSYDNNGDFWDFNLPNLSNKETDNNNPLLSSLSQMENQTLFSIEN

SGASQLLHNQVPFNFQTHLNNDNNNKSLNSDSILPPLTDCF

>g53401

MTWLPNGGCLGLLPVSHQDKLKLSMDRERKVGSRLASARWAAIASYLPQRTDNDIKNYWNTHLKKKLKKMQAEDGAESSG

GQDGNSSSSSHHSISKGQWERRLQTDIHLAKQALCEALSIDKSKPVLSAGGSSNPAPVPVQTGPYASSAENIARLLESWV

KNHGPTRSNSETTVTQTGFAGSTSSPSEATFDHSLFSFNSTCNNSEALSAVESKPVFARAAVAPPTFLTQPKPNNYNNAP

PETQMPLTLLENWLFDDAANVHPQEGIMGMPVPLAGTAELF

>g53402

MGRPPCCDKVGVKKGPWTPEEDIILVSYIQEHGPGNWRAVPTNTGLLRCSKSCRLRWTNYLRPGIKRGNFTEQEEKMIIH

LQALLGNRWAAIASYLPQRTDNDIKNYWNTHLKKKLKKMQGEDGAESSGGQDGNSSSSSHHSISKGQWERRLQTDIHLAK

QALCEALSIDKSKPVLSAGGSSNPAPVPVQTGPYASSAENIARLLESWVKNHGPTRSNSETTVTQTGFAGSTSSPSEATF

DHSLFSFNSTCNNSEALSAVESKPVFSRAAVAPPAFLTQPKPNNYNNAPPETQMPLTLLENWLFDDAANVHPQEGIMGMP

VPLAGTAELF

>g53830

MSVMRWIRWAQIAAKLPGRTDNEIKNFWNSCLKKKLIKRGIDPNTHKPIINTSSSSSDAPKSFAEDHNNINNSSSTIPKP

TLPWPLNLSELEQPQPFDPLFLYDFQQSLNNNNSEQYFHGFSTLPSLANFENMSSITVNNNGPTFPNWETCDEINKTDSM

LAQFPFSAKSEDVNGCSWQGHSQDDFSEYSLPALPQDLSGENNLEFFMSCTKFNL

>g54394

MGRSPCCVDKSGLKKGPWTPEEDQKLIHYIQANGPGNWRTLPKNAGLQRCGKSCRLRWTNYLRPDIKRGRFSFEEEETII

QLHSVLGNKWSAIAARLPGRTDNEIKNYWNTHIRKRLLRMGIDPVTHTPRLDLLDLSSILGTSTHLNFPSLLGLQAILNP

ELLRLATNLFASQNDNNINPDLLSNPHLQNQNPQMLLRKLQESQLLINPQTSRLTVMIIISSNIHSHLNNGQVFQESLLQ

SLENYGGCFGSQQSLSNSSNQSGLENFSFDSSVLSTPLNSSSTLVNSGGANTEDEKESFCSNLMRFDIPDSCLDFEDLL

>g55216

MGHHSCCNKQKVKRGLWSPEEDEKLINYISTYGHGCWSSVPRLAGLQRCGKSCRLRWINYLRPDLKRGSFSPQEAALIIE

LHLILGNRWAQIAKYLPGRTDNEVKNFWNSSIKKKIISRACFSDHLSAAISSNIPADPNTAPFDHQTLYSLNPNYNNLIL

HTTHHHQVLQDQAAGLISTAGTTSSSSPSSYLQAAAHQMIDQNGNLVLPMMPSDPAWFLGQQPQNLEHNFSIFSAAANDH

IAPDFDMMVFPAMPKLCEMIKAGGEGENMPISSSSSSSSSAVVAAAVQGGSDLVVPGSSLSCYPSGYNARDLHVPTGYDE

MEQTDTIILPSFPLLPPPPPPLSSLSPPSSFSGQSGNQIITYQS

>g55769

MGRYPCCKESSELKKGPWSTDEDKKLADYIHENGIGNWQLVPKGAGLNRCGKSCRLRWTNYLRPDIKRGCFSEEEEDMII

QLHKQLGNRWSKIAAKLPAGRTDNEVKNYYNTHIKKKLMRLGIDPITHKPLPPNLNHLLNNLPHNYYNSFNNNPIISPLE

SILRLQANLTQMANAQLLQNIAQFLNNNNNNIPLPLAQNNNNVPMLFNNNVTTFDNFNVGSNESPLFDLPLSTTPNSYSI

ESVLDQPSPMSNSLGFVPGNDDLVPRVLTSNNILSLPPLVSATPETSNSEQMFPTGAMQCGGGGGGDDDDFSALEKIFCD

EGNSSLLESIFQ

>g55803

MGRYPCCKESSELKKGPWSTDEDKKLADYIHENGIGNWQLVPKGAGLNRCGKSCRLRWTNYLRPDIKRGCFSEEEEDMII

QLHKQLGNRWSKIAAKLPAGRTDNEVKNYYNTHIKKKLMRLGIDPITHKPLPPNLNHLLNNLPNNYYNSFNNNPIISPLE

SILRLQANLTQMANAQLLQNIAQFLNNNNNPLPLAQNNNNVPMLFNNNVTTFDNFNVGSNESPPLFDHLLSTTPNSYSIE

SVLDQPSPMSNSLGFVPGNGDLVPRVLTSNNILSLPPLVSATPETSNSDQMFPTGAMQCGGGGGGDDDDDFSALEKIFCD

EGNSSLLESIFQ

>g57206

MGRTPCCDKKGLKKGPWTPEEDEKLIDFIKKNGQGSWRSLPKLAGLLRCGKSCRLRWTNYLRPDIKRGPFSSEEEKLVIQ

LHGILGNRWAAIASQLPGRTDNEIKNLWNTHLKKRLLSMGIDPQTHEPSSESNGPQRRLPTTPSARHMAQWESARLEAEA

RLSRESKLLVPSTIGGSEADFFLRIWNSEIGEAFRKLKKGVKFACQSPISQASSSTKYGSASGTTTEMDITLAGSADAGG

NPNEDPEGKSNRSYTDDPLQGSETSCSNELEDSSESALQLLLDFPTYNDMSFLGHTDTYSIYPAFLTESTLNCSSAEH

>g58953

MKERQRWRAEEDALLRAYVKQYGPKEWHLVSQRMNTPLNRDAKSCLERWKNYLKPGIKKGSLTEEEQRLVIHLQAKHGNK

WKKIAAEVPGRTAKRLGKWWEVFKEKQQREQKENNKIVEPVEEGKYDHILETFAEKIVKERSVPGLLMASSKGGFLHADP

PAPTPPNLLPPWLSNTTTTSAVRPPSPSVTLSLSPSTVPPSPVIPWLQSDRGSDKTPLTLSGFPPHHGISPPCGENPMVT

ELVECCKDLEEGHRAWAAHKKEATWRLRRVELQLESEKSCKIREKREEFDSKMKALMEEQKTSLDRIESDYKEQLAGLRR

DAEAKEQKLAEQWASKHLRLTKFLEQMGCPPRLAEPNGQ

>g58956

MDEERIEDCCPENKQSIAASSSSVSENSGSVNLKSPGVSSPAPSSPVHRRTSGPIRRAKGGWTPEEVSGGPEISDDTLKK

AVAAFKGKCWKKIAEFFPDRSEVQCLHRWQKVLNPELVKGPWTQEEDDKIIELVEKYGPTKWSIIAKSLPGRIGKQCRER

WHNHLNPNIKKDAWILEEELALINAHRVHGNKWAEIAKVLPGRTDNAIKNHWNSSLKKKLDFYLATGNLPPLAKNVSQNT

CKDISRTTPTEKGLECLSKRSDSTELASSGTTDVCKIVDGKIHLEGRSAIVDMGASTSSPQIESADSEDAPCEVKSSVCF

SPTFPTPPSVKHSNLCVQTPESILKIAAQSFRNTPSILRKRKTESQSSTPSNKIGKADDDGLDDIQLSNTKPFNASPPYR

LRSKRTSVLKSVEKQLDFTFSKEQDDNCPTTSGGSTVKQISQATKAMSDE

>g59241

MGRAPCCDKDGLKKGPWTPEEDQKLVDYIQKHGYGNWRTLPKNAGLQRCGKSCRLRWTNYLRPDIKRGRFSFEEEETIIQ

LHSILGNKWSAIAARLPGRTDNEIKNYWNTHIRKRLLRMGIDPVTHSPRLDLLDLSSILNTPSPLYNYPCQQINLSRLLG

LQQNLLNPHVLRLASSLFSPPVPRHGLNPEFGLQNNANQMCNMENPGFQGVQDNVQFSCPQSVTQEQTEVPVQNVNNVTF

SPVTQQPNNVDQFQPQFSSFPIGNCQENEWQNNGIPSSLTEDYLPLQNYGCYQPAVDPQSIMDPPPSDASTFCRSFQSVL

STPSSSPAPLNSNSTYINSCSTTTMDDEIDISYSSNLIDFDYSSILEVNEFM

>g59810

MGRSPCCEKAHTNKGAWTKEEDERLIAYIRAHGEGCWRSLPKAAGLLRCGKSCRLRWINYLRPDLKRGNFTEEEDELIIK

LHSLLGNKWSLIAGRLPGRTDNEIKNYWNTHIRRKLLSRGIDPTTHRPVDDPKEKVTTISFGPAKAAQEDDVNVVDEKKM

MMIRQDSLPTTTTTTTTVKQESTSPVRDDDERCPDLNLELRISPPYHPNQQPPLTLTPGSLVNVNNTAVCFACSLGIQNS

KDCTCSTNANAITSIAGYDFLGLRKNTLLDYRNLETMMN

>g60377

MEAADRIKGPWSPEEDEMLERLVEKYGARNWSLIGNSIPGRSGKSCRLRWCNQLSPEVEHRPFTAEEDETIIRAHAKFGN

KWATIARLLSGRTDNAIKNHWNSTLKRKCGSLSADLSFNPPLKRSSSAGSASDSDFSDIYKSIARTTGNGKLPLPLPLPP

LPSVFPAMETPSFSAADPVTSLSLSLPGSNEAPPLINRRNHLVPIPQLPPMSPPAKVYLPPQSFQFAAPPPPQAAEKPFF

SPEFLAELQEMIRKEVRNYMDGVEQNGSVRRLKQFKMRRRRRRRSEFGIGKIELTVSDEFHLCL

>g60983

MGHHNCCNKQKVKRGLWSPEEDEKLINYISNYGHGCWSTLPKLAGLQRCGKSCRLRWINYLRPDLKRGSFSPQEAALIIE

LHRILGNRWAQIAKHLPGRTDNEVKNFWNSSIKKKLISHGGRLSHLPSSPAPILSNVSSNPNFQTFYSQLPSIMPSVPLP

QLETTTSIDPTWFLGYPQSQNLVLDPQHYQISSYNNNNFDNEMMVPLLHHEDEPIIIPKLLPGNLLLSSSSLCSGIAQQD

DFIVPNPIPYSHHDLQIPSYEIMLPPMPALSLSSSSSSSSFSAVPCSQLLMNPSSWVP

>g61376

MGKGRAPCCDKSKVKKGPWSPAEDLRLISFIQKHGHPNWRALPSRLAHKIGFIPCTFLNSDYEFPRLLRCGKSCRLRWIN

YLRPDVKRGNFTPEEEETIIKLHNSLGNKWSKIAAHFPGRTDNEIKNVWNTHLKKRLTNKKGKGRSNNTDDDEDDDESPQ

NDTVSPDTPSPKDASLDASPTSSSSSSSTKHNQTPPEAPPEPPTPGDHDSVIDMEIWDMLDTLDNPSPQDPEIVVDLKPA

GSSCGGEIDRQVEWLRYLENELGLEDSPNNNNHAQPQKDNNDSNHNLVHLNSFDDNDDFNDMVASLYFPTWPPCSPQHFG

I

>g61550

MTNKSVCFRQRNLVEVNNVLIRFQQNASGCGTHVTVPLTSTLEVYLLPCQSYDCMYVVVIDELDHYTAYSGFREFVLTTS

AFPLPQPFLVLCASGTVTKPAEHALWVCINSWLCLTVNRWGGISIRHSGGPSREGIHVSRIGSDGEEFIANIQPSISDLD

NATLTAPITIQEIKEAVWALDPDSAARPDGYSGSFFRHCWHIIENDLYMAVLDFFAGVPVPQSIGSAQIMLLPKNSNPDS

FANFRPICLCTFISKVFTRIIMTRIKNLLPKLISKEQIGFVSGRSIQDNVLLAHELIHYIDKKCRGSNVAVKMDMMKAFD

RVAWPFLRAILKRFGFDQRIVNLILNNLASTRLSILVNGVSCGYFSPTRGVKQGDPLSPILFIIVSEALSSALIREVGSG

LISPYYTCPSMPTISHLAFADDLLIFTNGGKTSLKRLSTVLSRYQLASGQPINYHKSFFVTAKCCPANRLRTMERNLGMK

SSTLPFRYLGVNLFKGRNLAIYYQFILEKFDHSLQERRLIFGAPMITATSIIGLLGVRHKYPRPIFESRPSDSHIWKRMV

KIADEVEDCMMESSNGPIWTPSTFDEALLRLGLNTSRGMPLNSQISMEHCFFSCDVTLAVWHNFALIFDITRFPFGCIID

TRGETFALDFALRWCDLTPALLRFIEVDSKYLASLISSPSSKIPWRIRDALARIKEFLAFNNSITTESETPRPGALHCGP

LPERRTIAGFRDPDRKKRDEEFDGDDGFEADMEALKRACVLAGASPGHADDDFSGRAVSNSVPSSPDTDDGVDDVELVRD

IQKRFALSTDVQVPLDMRPICSIFPIEGGNESEDDMETLRAIERRFASYYDDTTKEGLDKELHNTEQVGVTNITSEEESC

NNFFLERTNAGEGFPTCVDGNNSALQISEGAIPQDGGAENASSESSGFPKSAQAFVDAIKKNRAFQKLIRSKMIHVEARI

EELKKLKERVKILKDYQVSCRKRTGHALAQKKDARVQLVLPRERGNSKLNEKKSSALYYAPPENSLVASYRDALEKFPVS

VNREKWSKEERENLLKGVKQQFQETMFQRAIDLSDMDGSFGDMANIDSNILSIRDLDITPGMMRQFLPKVNWDRLASMYV

PRHSGAECQTRWLNWEDPLINHEPWSVVQDKNLLHIVQQKGLSNWIDIALSLGTNRTPFQCLARYQRSLNASIIKREWTE

EEDNKLRAAVEVFGESNWQVVAASLEGRTGTQCSNRWIKTLHPARKRVGKWTADEDKRLKVAVMLFGPKTWRKIAQYVPG

RTHVQCRERWANSLDPSLNLNGWTEEEDLKLEAAIQEHGYSWSKVAACVAPRTDNQCRRRWKALFPHEVPLLRQARKIQK

AAFIANFVDRESERPSLKPDDFVPAPLLLQPSGSEPSRKRKIASRYFGIKAGRWMMGLGLVTTSLGNLFWIIKFGVFFFL

GGRRGWGMALVVMSNMSSDDPTIAENWRYGITYQSRKSKRQPKRKICTNRKRRRPANPGMLSNGNDLGGPEFSVAMNNRT

SKLPPRKKRKREPYVEVPEISASDEIETTNGDGIIFKRSTRGPEFESGSDNDAKVDTSSSFPNSFSDARTLGGKAIKSRK

RSIRRPRKNCSDLYVSQIYHPFPTSRKRKGPLREFLGSNLDADPSGELYDSSLSFEENSELESGAKQNQTTSELPAVACK

VKKKGLKCRDVDHSNKLAETEEDDSITLAAFIKKSVRLSDHNSNQSGAIPEHNIHTPTTSRGCEIQGHGNGLGFGPSPPT

SEAGIMDDDMPLAHFLNRLKRRVKPATSK

>g63073

MAQRSGPPLHNIYRGRIGKQCRERWHNHLNPSINKEAWTQEEELALIRAHQIYGNKWAELTKFLPGRTDNAIKNHWNSSV

KKKLDSYLASGLLSQFPALPNVNSANQILPTTSSKFQQSSEDRSVCREGKEVDEVSECSQGSALVGCSQSTSDRGNTFVH

TREERGVKDESSHEKDPSLTVTAMESPPLQTAMDLGTISHMGSIAVGSDNQDDMFNLEDGHCGIIYTGPENHQCFPSGNG

IRGVEESADSVILQPSNYQIPEAGMLAPQSCNTISSDNSGPSSYQAFSVPIMFGVDSDQLIENPRQEIITCGDFVYASEP

GSSTCGNRMDGQGVSTCNGMDDQGLKDQMDQYIQENECVKLVPANDFGPVQPDSVQTYMQQEYSPLGIRQLMMSSMNCLT

PFRLWDSPSRDSSPDAVLKSAAKTFTCTPSILKKRHRDLVSPLSEKRSEKKLESDIKHEFSDLARNFSRLEVMVGDAGNE

KATRSSPLPNQEFISESSIEDKENLNPEFEGTTKEGMEGTRFSGRRDLGRESNKGDADDTIQVKRTVGVLVERGKDDPLF

FSPDRFGSKSDRAVTQTTKVLGNSCLNRLETAPNQGTVSSSSEAPCLSVCSPRICAIKEGNNLVVATSVQSVPASENAGE

GSGNGVGLDNNISIFGETPFKRSIESPSAWKSPWFINSFLSSPRVDTEITLEDFGFLFSPGDRSYDAIGLMKQLSEQTAA

TFADAQEVLGGETPESILRGRCSKNQKADENTSQFTSNVLPSVTSSNPSSLGLNRSAMGNLVWFTSLWSSAAIAARVKAV

LLSHTCRTERRMLDFSECGTPGKKAESGKFPGGSSSSISSPSSYLLKECR

>g63810

MGRAPCCDKEKVKRGPWSPEEDEKLKEYIEKNGPGGNWIALPQKAGLRRCGKSCRLRWLNYLRPNIKHGDFSDQEDRIIC

SLYATIGSRWSIIAAQLPGRTDNDIKNYWNTKLKKKLMGIMLSSSSSSPHQKTRPNPLPPSSSSPFNYNNYPITDPTNFP

PQYAAATTFLNNPSSSSSAASNPVGPTIQTHHLLVKGDHHHHHHQGSCSSSDGSWSNNNNQISQDKEYGYGESLGLQSFG

YNNGVGENQQAVNNGGNPLDYSSLEEIKQLISTNNVSQQNSFFVEENKYEEKVMMYY
